# Global Health Research on Refugees and Other Forcibly Displaced Populations: A Bibliometric Analysis from 2000 to 2024

**DOI:** 10.3390/ijerph23070864

**Published:** 2026-07-01

**Authors:** Zaid Ahmed Shaik, Noor Fathima Shaik, Aba Barden-Maja

**Affiliations:** 1 Department of Engineering, Drexel University, Philadelphia, PA 19104, USA; zas48@drexel.edu; 2 Department of Neurology, Hospital of the University of Pennsylvania, Philadelphia, PA 19104, USA; 3 Department of Medicine, Hospital of the University of Pennsylvania, Philadelphia, PA 19104, USA; aba.barden-maja@pennmedicine.upenn.edu

**Keywords:** refugees, asylum seekers, migrants, forcibly displaced persons, global health

## Abstract

**Highlights:**

**Public health relevance—How does this work relate to a public health issue?**

- Forced displacement is a major global public health challenge affecting more than 120 million people, with significant implications for their physical, mental, and social well-being.
- This study maps how health research has responded to displacement-related needs, highlighting patterns in attention to mental health, infectious disease, maternal health, and other core public health domains.

**Public health significance—Why is this work of significance to public health?**

- By analyzing global trends over two decades, it helps public health professionals understand how humanitarian crises shape research priorities and reveals areas needing urgent scientific attention (such as non-communicable diseases and environmental health), guiding more equitable and effective resource allocation.

**Public health implications—What are the key implications or messages for practitioners, policy makers and/or researchers in public health?**

- Practitioners and policymakers can use these findings to design policies and care models that better address gaps in how health systems respond to displacement, especially around chronic disease and climate-related risks, while also supporting the current needs in mental health and infectious disease.
- Researchers can use this work to help prioritize underexplored domains, diversify study designs (including increasing longitudinal and interventional research), and expand geographic representation.

**Abstract:**

The global population of forcibly displaced persons (FDPs), including refugees and asylum seekers, surpassed 120 million in 2024. Understanding research trends on FDP health is essential for addressing their complex needs. To characterize trends in health research focused on these populations, we conducted a bibliometric analysis of PubMed-indexed publications from 2000 to 2024 using the title and abstract-based search terms “refugee,” “asylum seeker,” “asylee,” and “forcibly displaced.” A total of 1590 relevant publications were included. Research output grew modestly from 2000 to 2013, surged between 2013 and 2017, and slowed from 2017 to 2024. Review articles dominated (64%), followed by comparative studies (13%) and randomized controlled trials (8%). Mental and psychosocial health was the most common theme (31%), followed by health policy (26%) and children/youth (22%). Non-communicable diseases (9%) and climate-related health (1.5%) were underrepresented. Geographic focus shifted from Southeast Asia and East Africa in the early 2000s, to Syria post-2016, then with emerging attention to Rohingya and Ukrainian refugees in the early 2020s. This study provides an updated overview of Pubmed-indexed research trends, and highlights thematic and geographic gaps that warrant further investigation.

## 1. Introduction

According to the United Nations High Commissioner for Refugees (UNHCR), there were over 120 million forcibly displaced persons (FDPs) worldwide in 2024, including refugees, asylum seekers, and other displaced groups. This number has nearly doubled over the past decade and continues to rise in response to ongoing global and humanitarian crises [1]. Growing recognition of the unique health challenges faced by FDPs has led to an expansion of research on their medical, psychological, and social needs. Bibliometric analysis offers a structured approach to understanding how this body of research has evolved over time. By examining patterns in publication volume, thematic focus, authorship and geography, such analyses can highlight research priorities as well as gaps that require further study. Prior bibliometric work in this field has often been limited in scope to voluntary migration. For example, some studies have focused on migrants who were not forcibly displaced such as Arab migrants [2] or international migrant workers [3], while others have evaluated research within host countries, like Ireland [4] or Scotland [5]. Additional publications have examined very specialized topics, including mental health or the perinatal experiences of displaced refugee and asylee women [6,7], who would fall into the category of FDPs.

The most recent global bibliometric study assessing health-related research on FDPs covered only the period up to 2015, predating several major displacement events and global crises of the past decade [8]. These include the Syrian civil war, the Rohingya crisis, the COVID-19 pandemic, and the mass displacement associated with the conflict in Ukraine; events that have undoubtedly reshaped scholarly output. For instance, COVID-19 led to a major shift in research publications related to the disease along with cross-field collaboration [9]. Thus, given the differences in scope of previous work, we sought to perform a bibliometric analysis of literature regarding refugees and other FDPs published globally from 2000 to 2024, aiming to identify thematic concentrations, geographic trends, and gaps to inform future research. This work is not encompassing of all FDPs but attempts to focus on persons who were forcibly displaced instead of those who have voluntarily migrated. The conceptual framework for this study is based on bibliometric analysis, which systematically evaluates patterns in scholarly output to understand the trends, focus, and gaps in research [10].

## 2. Materials and Methods

We reviewed articles on refugees, asylum seekers, and other forcibly displaced populations in the PubMed database, using a title and abstract-based search strategy with key terms, as used by others [8]. This search strategy was selected to prioritize studies that explicitly highlighted displaced populations as their primary focus. While this approach increases precision, it excludes studies in which displaced populations are addressed secondarily or described only in the text, as discussed in the limitation section. The words “refugee” OR “asylum seeker” OR “asylee” OR “forcibly displaced” were entered as the main search into PubMed’s search engine. The publication date range was limited to 1 January 2000, through 31 December 2024. Finally, article types were restricted to articles categorized as “Classical Article, Clinical Study, Clinical Trial, Comparative Study, Meta-Analysis, Observational Study, Randomized Controlled Trial, Review, Scoping Review, Systematic Review”. The complete search string is as follows: (“refugee s” [All Fields] OR “refugees” [MeSH Terms] OR “refugees” [All Fields] OR “refugee” [All Fields] OR (“refugees” [MeSH Terms] OR “refugees” [All Fields] OR (“asylum” [All Fields] AND “seeker” [All Fields]) OR “asylum seeker” [All Fields]) OR (“asylee” [All Fields] OR “asylees” [All Fields]) OR (“forcibly” [All Fields] AND (“displace” [All Fields] OR “displaced” [All Fields] OR “displacement, psychological” [MeSH Terms] OR (“displacement” [All Fields] AND “psychological” [All Fields]) OR “psychological displacement” [All Fields] OR “displacement” [All Fields] OR “displacements” [All Fields] OR “displaces” [All Fields] OR “displacing” [All Fields]))) AND ((classicalarticle [Filter] OR clinicalstudy [Filter] OR clinicaltrial [Filter] OR comparativestudy [Filter] OR meta-analysis [Filter] OR observationalstudy [Filter] OR randomizedcontrolledtrial [Filter] OR review [Filter] OR scopingreview [Filter] OR systematicreview [Filter]) AND (2000:2024 [pdat])). The retrieved data were exported to Microsoft Excel for further analysis. The search was conducted on 14 June 2025, and all analyses were based on the results of this search to preclude changes in citation metrics.

Exported data included PubMed ID (PMID), title, authors, journal, publication year, type of paper, and keywords. Affiliation of the authors (both country and institution) were additionally recorded for each paper. Each paper’s titles and abstracts were individually reviewed to exclude papers that were not relevant to FDPs, such as molecular studies. Each included article was then assigned one or more thematic categories derived from prior bibliometric literature: (1) mental/psychosocial health; (2) health policy and systems; (3) children and youth; (4) infectious diseases; (5) maternal and reproductive health; (6) non-communicable diseases; and (7) environmental or climate-related health [3,5,6,7].

Thematic coding was conducted by two independent reviewers using a framework developed iteratively based on title, abstract, and keyword review. Articles were permitted to receive multiple thematic assignments when relevant, with a primary theme and additional secondary themes if applicable. There was a high level of agreement (over 98%), and for any articles for which there was uncertainty or disagreement, the reviewers would re-review these articles, discuss amongst themselves, and come to an accord.

Measures studied in this analysis included journal of publication, publication year, authors’ country of institutional affiliation and institutional affiliation, type of paper, number of citations, title, keywords, thematic category as described above, and geographic focus if applicable. Geographic focus was determined using explicit country or regional references in article titles and abstract reviews when applicable.

For the trend of papers published between 2000 and 2024, the Joinpoint Regression Program (Version 5.4.0; Bethesda, MD, USA) from the National Cancer Institute was used to calculate the average annual percentage change (AAPC), with final model selection for the number of joinpoints determined using the weighted Bayesian information criterion [11]. GraphPad Prism (version 10.6.1; Boston, MA, USA) was used to visualize the data. Due to the nature of this publicly available database, this research is considered nonhuman research under US regulation (45 CFR §46.102 [d]) and IRB review was not required.

## 3. Results

**Figure 1:**
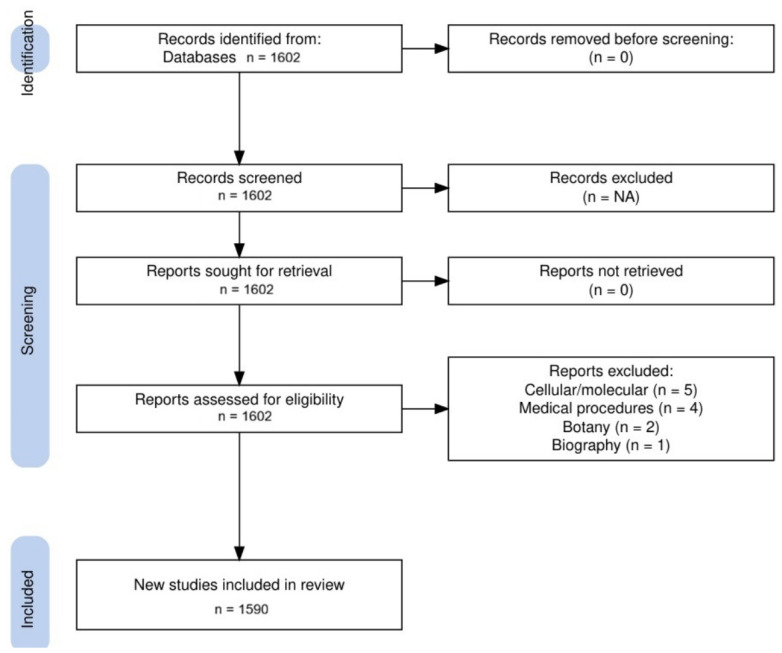
Flow chart delineates how 1602 records were identified and all screened to assess eligibility. Twelve papers were removed for lack of relevance to this topic, and thus 1590 studies were ultimately included in this analysis. This flowchart was made using an R-package version 1.1.3 developed by others [12].

Using the aforementioned search terms and as delineated in the flowchart of Figure 1, we retrieved 1602 publications. On further review, 12 of these papers did not involve our study population (i.e., five papers were cellular/molecular papers involving topics like refugee proteins, four papers were regarding medical/surgical procedures detailing approach to injuries such as displaced joints, two studies about the migration of plants, and one paper was a biography in memoriam); thus, these and were excluded from analysis. Therefore, we ultimately analyzed 1590 papers that studied the health of FDPs between 2000 and 2024.

**Figure 2:**
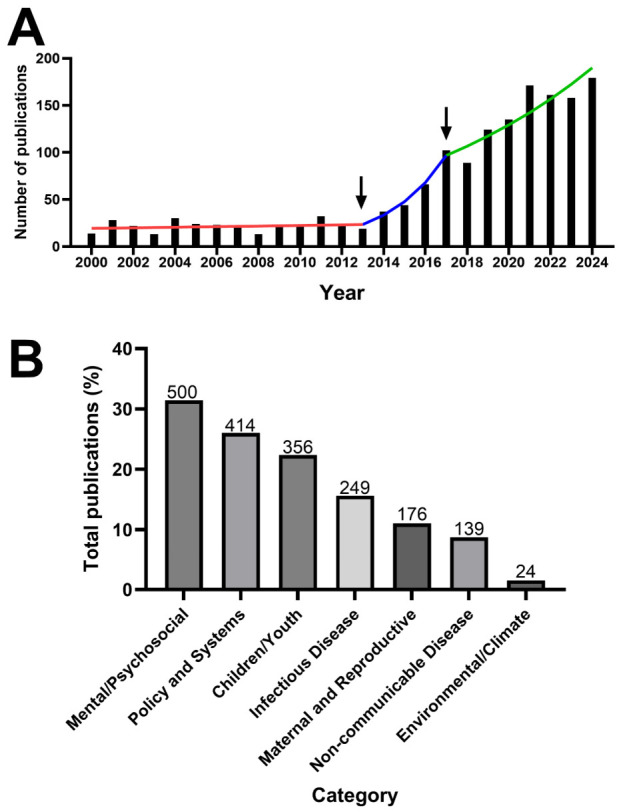
(**A**) Number of publications per year. The number of publications is displayed as vertical bars with superimposed joinpoint analysis highlighting joinpoints at 2013 and 2017 (delineated by black arrows) separating the three colored line segments in red, blue, and green (reflecting changes in the rate of publications per year). (**B**) The thematic categories of the publications. The bars represent total publications within the thematic category as a percentage, and the respective *n* value of papers is included on top of each bar. Mental/psychosocial health was the most common category.

For the number of papers published annually, Joinpoint analysis identified two joinpoints with three segments as the final model (Figure 2A). The first segment (in red) from 2000 to 2013 had an AAPC of 1.49%, and this jumped to an AAPC of 42.49% between 2013 and 2017 (segment in blue), indicating a large spike in the number of publications between 2013 and 2014. The second joinpoint is between the segments connecting 2013–2017 and the final segment (in green) between 2017 and 2024, the latter of which has an AAPC of 10.10% indicating slower growth in this final sector.

Looking at thematic categories (Figure 2B), nearly one in three (31.45%, *n* = 500) looked at mental/psychosocial health, followed by health policy and systems (26.04%, *n* = 414), children/youth (22.39%, *n* = 356), infectious disease (15.66%, *n* = 249), maternal and reproductive health (11.07%, *n* = 176), non-communicable diseases (8.74%, *n* = 139), and environmental/climate (1.51%, *n* = 24). Papers that have multiple thematic categories were counted for each of their respective themes (i.e., a paper on mental health in children was included in both the mental/psychological health group and the children/youth group. Some papers, *n* = 256, involved multiple themes, and the most common overlapping themes were mental/psychosocial health in children/youth (*n* = 134).

These themes are further detailed with common subtopics below in Table 1. Please see supplemental data (Table S1) for accompanying reviewed references.

The majority of papers were review articles (64.15%, *n* = 1020), followed by comparative studies (13.14%, *n* = 209), randomized controlled trials (8.05%, *n* = 128), meta-analyses (5.41%, *n* = 86), observational studies (3.46%, *n* = 55), with the remaining 92 papers (5.79%) consisting of study protocols, non-randomized clinical trials, feasibility/pilot studies, and case reports. When looking at specifically clinical trials, comparative studies, observational studies, feasibility/pilot studies, and randomized controlled trials, these encompassed approximately 3,012,698 patients. While all the titles, abstracts, and keywords were reviewed in English, we did not filter by language. The vast majority of these papers were in written in English (95%), with German and French being the next most common article languages. Leading journals that published literature involving people who have been forcibly displaced were the Journal of Immigrant and Minority Health (*n* = 55), the International Journal of Environmental Research and Public Health (*n* = 48), PLOS ONE (*n* = 42), and BMC Public Health, BMJ Open, and Trials (each of which had 24 publications). Authors typically came from the United States (*n* = 563), United Kingdom (*n* = 252), Australia (*n* = 232), Canada (*n* = 167), and Germany (*n* = 147), and leading institutional affiliations were Johns Hopkins University (*n* = 78), the University of New South Wales (*n* = 63), Harvard University (*n* = 51), the University of Toronto (*n* = 48), and Columbia University (*n* = 44). The range of citations per publication was from 0 to 788 citations, with a median of six citations per paper.

Finally, the top keywords were “refugee” (46.98%, *n* = 747), “health” (41.45%, *n* = 659), “mental” (13.60%, *n* = 214), “migrant” (8.87%, *n* = 141), and “immigrant” (8.55%, *n* = 136). Notably papers did not regularly start incorporating keywords until 2013. For looking at geographic focus of countries and regions most frequently mentioned explicitly in titles, publications between 2000 and 2008 most often mentioned people from Southeast Asian countries like Vietnam and Cambodia, followed by East African nations like Tanzania and Uganda, and finally Balkan countries such as Kosovo, Serbia, and Bosnia and Herzegovina. From 2009 to 2015, while Southeast Asian and East African countries were still frequently mentioned, there was more discussion of people from Middle Eastern countries, especially Afghanistan. In 2016, there was a major demographic shift with research increasingly focusing on people displaced from Syria. Finally, while Syrian refugees are still a large area of research, the early 2020s saw additional studies published about the Rohingya people and Ukrainian refugees.

## 4. Discussion

This bibliometric analysis of 1590 publications describes how health research focused on refugees and other forcibly displaced populations has evolved over the past two decades, revealing evolving scholarly attention shaped by global crises and shifting research priorities. The sharp increase in publications between 2013 and 2017 aligns with major humanitarian crises, most notably the Syrian civil war. While others have described temporal patterns of research output [8], our study uniquely utilizes Joinpoint analysis to reveal where there are statistically significant shifts in the research output. Geographic focus similarly shifted over time; earlier work concentrated on Southeast Asia, East Africa, and the Balkans, whereas more recent studies increasingly examined displaced groups from Syria, the Rohingyas, and in the early 2020s, Ukrainian refugees. These patterns suggest how global crises may influence the trajectory of research on displaced populations. However, given the descriptive nature of this analysis, these patterns should be interpreted cautiously as temporal associations rather than evidence of causality. Alternative explanations including logistical publication lags, evolving funding cycles, and variable humanitarian policies and research agendas may also contribute and were not studied in this analysis.

Prior studies that have performed thematic analysis have primarily focused on migrant workers and other voluntary migrating groups like immigrants. Our work expands this research to populations who have been forcibly displaced. Mental/psychosocial health emerged as the most frequently studied theme in our investigation, consistent with prior work on the psychological effects of international migrant workers and immigrants [3,6]. Our study thus expands the relevance of mental and psychosocial health to people who have been forcibly displaced, underscoring the need for screening and treatment. Health policy and systems research also featured prominently, reflecting interest in structural responses to both forced displacement and to voluntary migration [4,5]. The attention given to research on children and maternal health suggests special needs to vulnerable subgroups, as with a previous review on perinatal experience among refugee and asylum-seeking women [7]. Notably, our work highlights how non-communicable diseases such as heart disease and diabetes, along with the effects of environmental health are particularly understudied despite their impact on forcibly displaced populations.

The predominance of review articles (64%) suggests a continued emphasis on synthesizing existing knowledge, likely reflecting a combination of PubMed article-type indexing practices and the methodological burden of conducting primary research among displaced populations, including ethical constraints, population mobility, and data access challenges. Meta-analyses composed only 5% of studies, often limited by heterogeneity in study populations and methodologies. Comparative studies and randomized controlled trials together accounted for nearly one-quarter of the literature, possibly indicating growing interest in intervention-based research. Taken together, these findings underscore the need for more rigorous designs to guide evidence-based policy.

Consequently, future research areas include underrepresented health domains such as chronic disease and environmental health, and potential for studies with more longitudinal designs for displaced people who have been in their host countries for several years. Additionally, there is limited research for people who have been forcibly displaced from South and Central America, highlighting a gap in studies involving this geographic region.

This study has several important limitations. Firstly, while the search terms we employed focus on forcibly displaced persons, including refugees and asylum seekers as defined by the UNHCR, it does not definitively encompass everyone who has been forcibly displaced such as those who are stateless or Palestinian refugees under the United Nations Relief and Works Agency for Palestine Refugees in the Near East. Second, reliance on the single database of PubMed may exclude literature indexed in social science, regional, or humanitarian databases and likely overrepresents biomedical research conducted in high-income countries. Third, while the title- and abstract-based search strategy we employed is based on other published work in this field and increases specificity, this possibly excludes studies that address displaced populations that were not named explicitly. Fourth, while we did not filter studies by language, the vast majority of the studies (95%) were written in English, introducing language bias. Fifth, though thematic classification was conducted by two independent reviewers with high inter-rater agreement (over 98%), this strategy may still introduce subjectivity. Finally, while we attempted to analyze temporal trends of geographic focus, these patterns are only associations and do not signify causality. Collectively, these limitations constrain generalizability and emphasize that findings reflect patterns in PubMed-indexed literature captured using a conservative retrieval strategy, rather than the entirety of global research on displaced populations.

## 5. Conclusions

This study analyzed health-related research on refugees, asylum seekers, and other forcibly displaced persons from 2000 to 2024, highlighting thematic and geographic trends as well as potential gaps. By extending prior bibliometric work through 2024 and incorporating major recent displacement events and global crises, including the Syrian civil war, the Rohingya crisis, and the war in Ukraine, this study provides an updated global assessment of health-related research on FDPs.

## Figures and Tables

**Table 1:** Common subtopics are listed within each of the seven themes used for categorization.

Theme	Common Subtopics
Mental/Psychosocial	Post-traumatic stress, depression, drug use, Treatments and interventions
Health Policy and Systems	Access to resources, insurance, legal barriers, integration
Children/Youth	Education, childhood mortality, malnutrition, child trafficking
Infectious Disease	Tuberculosis, COVID-19, vaccination, malaria
Maternal and Reproductive	Antenatal and post-natal care, contraception, menstruation
Non-communicable Disease	Diabetes, hypertension, dental care, cancer
Environmental/Climate	Air quality, heat extremes, wildfires

## Data Availability

Data is from a publicly available database with the search parameters as described above.

## Supplementary Materials

The following supporting information can be downloaded at https://www.mdpi.com/article/10.3390/ijerph23070864/s1. Table S1. Common subtopics are listed within each of the seven themes used for categorization, along with relevant references.

## Abbreviations

The following abbreviations are used in this manuscript:

FDP	forcibly displaced persons
PMID	PubMed ID
AAPC	average annual percentage change
UNHCR	United Nations High Commissioner for Refugees

## References

[1] United Nations High Commissioner for Refugees (2025). Global Trends: Forced Displacement in 2024.

[2] Sweileh W.M. (2018). Global research output in the health of international Arab migrants (1988–2017). BMC Public Health.

[3] Sweileh W.M. (2018). Global output of research on the health of international migrant workers from 2000 to 2017. Glob. Health.

[4] Villarroel N., Hannigan A., Severoni S., Puthoopparambil S., MacFarlane A. (2019). Migrant health research in the Republic of Ireland: A scoping review. BMC Public Health.

[5] Petrie G., Angus K., O’Donnell R. (2024). A scoping review of academic and grey literature on migrant health research conducted in Scotland. BMC Public Health.

[6] Khosa M., Bhulani N., Ali A.A., Singh J., Khosa F., Nasrullah M. (2019). Bibliometrics of Fifty Most-Cited Articles on the Mental Health of Immigrants Living in the United States. J. Immigr. Minor. Health.

[7] Ramadan M., Rukh E.Q.H., Yang S., Vang Z.M. (2023). Fifty years of evidence on perinatal experience among refugee and asylum-seeking women in Organization for Economic Co-operation and Development (OECD) countries: A scoping review. PLoS ONE.

[8] Sweileh W.M. (2017). Bibliometric analysis of medicine—Related publications on refugees, asylum-seekers, and internally displaced people: 2000–2015. BMC Int. Health Hum. Rights.

[9] Dang Z., Li L., Peng H., Zhang J. (2021). Impact of Sudden Global Events on Cross-Field Research Cooperation. Information.

[10] Fraser H.S.F., Zahiri K., Kim N., Kim C., Craig S. (2023). The Global Health Informatics landscape and JAMIA. J. Am. Med. Inform. Assoc..

[11] Kim H.J., Chen H.S., Byrne J., Wheeler B., Feuer E.J. (2022). Twenty years since Joinpoint 1.0: Two major enhancements, their justification, and impact. Stat. Med..

[12] Haddaway N.R., Page M.J., Pritchard C.C., McGuinness L.A. (2022). PRISMA2020: An R package and Shiny app for producing PRISMA 2020-compliant flow diagrams, with interactivity for optimised digital transparency and Open Synthesis. Campbell Syst. Rev..

