# Supplementary material for: Global Health Research on Refugees and Other Forcibly Displaced Populations: A Bibliometric Analysis from 2000 to 2024
Source: Int J Environ Res Public Health. 2026 Jul 1;23(7):864. doi: 10.3390/ijerph23070864 (PMC13410235; doi:10.3390/ijerph23070864)
Supplement: Supplementary file 1 [file ijerph-23-00864-s001.zip › ijerph-4256460-supplementary.pdf]

Supplemental References for Table S1:

Table S1. Common subtopics are listed within each of the seven themes used for categorization, along with relevant references.

Theme	Common Subtopics	References
Mental/Psychosocial	Post-traumatic stress, depression, drug use, Treatments and interventions	13-481
Health Policy and Systems	Access to resources, insurance, legal barriers, integration	482-869
Children/Youth	Education, childhood mortality, malnutrition, child trafficking	870-1060
Infectious Disease	Tuberculosis, COVID-19, vaccination, malaria	1061-1296
Maternal and Reproductive	Antenatal and post-natal care, contraception, menstruation	1297-1461
Non-communicable Disease	Diabetes, hypertension, dental care, cancer	1462-1583
Environmental/Climate	Air quality, heat extremes, wildfires	1584-1602

List of all reviewed references

13. Chowdhury AN. Torture and mental health. J Indian Med Assoc. 2000 Jun;98(6):320-6.
14. Keyes EF. Mental health status in refugees: an integrative review of current research. Issues Ment Health Nurs. 2000 Jun;21(4):397-410. https://doi.org/10.1080/016128400248013
15. Fox PG, Burns KR, Popovich JM, Ilg MM. Depression among immigrant Mexican women and Southeast Asian refugee women in the U. S. Int J Psychiatr Nurs Res. 2001 Jun;7(1):778-92.
16. Weinstein CS, Fucetola R, Mollica R. Neuropsychological issues in the assessment of refugees and victims of mass violence. Neuropsychol Rev. 2001 Sep;11(3):131-41. https://doi.org/10.1023/a:1016650623996
17. Terheggen MA, Stroebe MS, Kleber RJ. Western conceptualizations and Eastern experience: a cross-cultural study of traumatic stress reactions among Tibetan refugees in India. J Trauma Stress. 2001 Apr;14(2):391-403. https://doi.org/10.1023/A:1011177204593
18. Levine J. Working with victims of persecution: lessons from Holocaust survivors. Soc Work. 2001 Oct;46(4):350-60. https://doi.org/10.1093/sw/46.4.350
19. Smajkic A, Weine S, Djuric-Bijedic Z, Boskailo E, Lewis J, Pavkovic I. Sertraline, paroxetine, and venlafaxine in refugee posttraumatic stress disorder with depression symptoms. J Trauma Stress. 2001 Jul;14(3):445-52. https://doi.org/10.1023/A:1011177420069
20. Nørregaard C. [Reactions to torture and persecution. Traumatized refugees in the Danish health service]. Ugeskr Laeger. 2001 Apr 2;163(14):1992-6.
21. Smajkić A, Weine S, Durić-Bijedić Z, Boskailo E, Lewis J, Pavković I. Sertralilne, paroxetine and venlafaxine in refugee post traumatic stress disorder with depression symptoms. Med Arh. 2001;55(1 Suppl 1):35-8.
22. Watters C. Emerging paradigms in the mental health care of refugees. Soc Sci Med. 2001 Jun;52(11):1709-18. https://doi.org/10.1016/s0277-9536(00)00284-7
23. Bolton P. Cross-cultural validity and reliability testing of a standard psychiatric assessment instrument without a gold standard. J Nerv Ment Dis. 2001 Apr;189(4):238-42. https://doi.org/10.1097/00005053-200104000-00005
24. Bolton P. Local perceptions of the mental health effects of the Rwandan genocide. J Nerv Ment Dis. 2001 Apr;189(4):243-8. https://doi.org/10.1097/00005053-200104000-00006

25. Hermansson AC, Timpka T, Thyberg M. The mental health of war-wounded refugees: an 8-year follow-up. J Nerv Ment Dis. 2002 Jun;190(6):374-80. https://doi.org/10.1097/00005053-200206000-00005
26. Bolton P, Neugebauer R, Ndogoni L. Prevalence of depression in rural Rwanda based on symptom and functional criteria. J Nerv Ment Dis. 2002 Sep;190(9):631-7. https://doi.org/10.1097/00005053-200209000-00009
27. Bolton P, Bass J, Neugebauer R, Verdeli H, Clougherty KF, Wickramaratne P, Speelman L, Ndogoni L, Weissman M. Group interpersonal psychotherapy for depression in rural Uganda: a randomized controlled trial. JAMA. 2003 Jun 18;289(23):3117-24. https://doi.org/10.1001/jama.289.23.3117
28. Procter N. Emergency mental health nursing for refugees and asylum seekers. Aust Nurs J. 2004 Dec-2005 Jan;12(6):21-3.
29. Neuner F, Schauer M, Klaschik C, Karunakara U, Elbert T. A comparison of narrative exposure therapy, supportive counseling, and psychoeducation for treating posttraumatic stress disorder in an african refugee settlement. J Consult Clin Psychol. 2004 Aug;72(4):579-87. https://doi.org/10.1037/0022-006X.72.4.579
30. Fenta H, Hyman I, Noh S. Determinants of depression among Ethiopian immigrants and refugees in Toronto. J Nerv Ment Dis. 2004 May;192(5):363-72. https://doi.org/10.1097/01.nmd.0000126729.08179.07
31. Rousseau C, Drapeau A. Premigration exposure to political violence among independent immigrants and its association with emotional distress. J Nerv Ment Dis. 2004 Dec;192(12):852-6. https://doi.org/10.1097/01.nmd.0000146740.66351.23
32. Lopes Cardozo B, Talley L, Burton A, Crawford C. Karenni refugees living in Thai-Burmese border camps: traumatic experiences, mental health outcomes, and social functioning. Soc Sci Med. 2004 Jun;58(12):2637-44. https://doi.org/10.1016/j.socscimed.2003.09.024
33. Silove D. The challenges facing mental health programs for post-conflict and refugee communities. Prehosp Disaster Med. 2004 Jan-Mar;19(1):90-6. https://doi.org/10.1017/s1049023x00001539
34. Boehnlein JK, Kinzie JD, Sekiya U, Riley C, Pou K, Rosborough B. A ten-year treatment outcome study of traumatized Cambodian refugees. J Nerv Ment Dis. 2004 Oct;192(10):658-63. https://doi.org/10.1097/01.nmd.0000142033.79043.9d
35. Procter NG. Emergency mental health nursing for self-harming refugees and asylum seekers. Int J Ment Health Nurs. 2005 Sep;14(3):196-201. https://doi.org/10.1111/j.1440-0979.2005.00381.x
36. Porter M, Haslam N. Predisplacement and postdisplacement factors associated with mental health of refugees and internally displaced persons: a meta-analysis. JAMA. 2005 Aug 3;294(5):602-12. https://doi.org/10.1001/jama.294.5.602
37. Fazel M, Wheeler J, Danesh J. Prevalence of serious mental disorder in 7000 refugees resettled in western countries: a systematic review. Lancet. 2005 Apr 9-15;365(9467):1309-14. https://doi.org/10.1016/S0140-6736(05)61027-6
38. Mills EJ, Singh S, Holtz TH, Chase RM, Dolma S, Santa-Barbara J, Orbinski JJ. Prevalence of mental disorders and torture among Tibetan refugees: a systematic review. BMC Int Health Hum Rights. 2005 Nov 9;5:7. https://doi.org/10.1186/1472-698X-5-7
39. Nguyen NL, Hunt DD, Scott CS. Screening for depression in a primary care setting in Vietnam. J Nerv Ment Dis. 2005 Feb;193(2):144-7. https://doi.org/10.1097/01.nmd.0000152811.72059.d3

40. Steel Z, Silove D, Chey T, Bauman A, Phan T, Phan T. Mental disorders, disability and health service use amongst Vietnamese refugees and the host Australian population. Acta Psychiatr Scand. 2005 Apr;111(4):300-9. https://doi.org/10.1111/j.1600-0447.2004.00458.x
41. Henderson DC, Mollica RF, Tor S, Lavelle J, Culhane MA, Hayden D. Building primary care practitioners' attitudes and confidence in mental health skills in a post-conflict society: a Cambodian example. J Nerv Ment Dis. 2005 Aug;193(8):551-9. https://doi.org/10.1097/01.nmd.0000172869.01711.33
42. Procter NG. 'They first killed his heart (then) he took his own life'. Part 2: Practice implications. Int J Nurs Pract. 2006 Feb;12(1):42-8. https://doi.org/10.1111/j.1440-172X.2006.00548.x
43. Kucukalić A, Bravo-Mehmedbasić A, Fadilpasić S, Priebe S, Gavrilović-Janković J, McCrone P, Schützwoh M, Ljubotina D, Francisković T, Lecić-Tosevski D. [Comparison of socio-demographic characteristics of patients with PTSD in the four countries of former Yugoslavia following the war]. Med Arh. 2006;60(4):230-3.
44. Keller A, Lhewa D, Rosenfeld B, Sachs E, Aladjem A, Cohen I, Smith H, Porterfield K. Traumatic experiences and psychological distress in an urban refugee population seeking treatment services. J Nerv Ment Dis. 2006 Mar;194(3):188-94. https://doi.org/10.1097/01.nmd.0000202494.75723.83
45. Sabin M, Sabin K, Kim HY, Vergara M, Varese L. The mental health status of Mayan refugees after repatriation to Guatemala. Rev Panam Salud Publica. 2006 Mar;19(3):163-71. https://doi.org/10.1590/s1020-49892006000300004
46. Silove D, Steel Z, Susljik I, Frommer N, Loneragan C, Chey T, Brooks R, le Touze D, Ceollo M, Smith M, Harris E, Bryant R. The impact of the refugee decision on the trajectory of PTSD, anxiety, and depressive symptoms among asylum seekers: a longitudinal study. Am J Disaster Med. 2007 Nov-Dec;2(6):321-9.
47. Alexander A, Blake S, Bernstein MA. The staying power of pain. A comparison of torture survivors from Bosnia and Colombia and their rates of anxiety, depression and PTSD. Torture. 2007;17(1):1-10.
48. Piwowarczyk L. Asylum seekers seeking mental health services in the United States: clinical and legal implications. J Nerv Ment Dis. 2007 Sep;195(9):715-22. https://doi.org/10.1097/NMD.0b013e318142ca0b
49. Silove D, Manicavasagar V, Mollica R, Thai M, Khiek D, Lavelle J, Tor S. Screening for depression and PTSD in a Cambodian population unaffected by war: comparing the Hopkins Symptom Checklist and Harvard Trauma Questionnaire with the structured clinical interview. J Nerv Ment Dis. 2007 Feb;195(2):152-7. https://doi.org/10.1097/01.nmd.0000254747.03333.70
50. Mollica RF, Caridad KR, Massagli MP. Longitudinal study of posttraumatic stress disorder, depression, and changes in traumatic memories over time in Bosnian refugees. J Nerv Ment Dis. 2007 Jul;195(7):572-9. https://doi.org/10.1097/NMD.0b013e318093ed2c
51. Grodin MA, Piwowarczyk L, Fulker D, Bazazi AR, Saper RB. Treating survivors of torture and refugee trauma: a preliminary case series using qigong and t'ai chi. J Altern Complement Med. 2008 Sep;14(7):801-6. https://doi.org/10.1089/acm.2007.0736
52. Neuner F, Onyut PL, Ertl V, Odenwald M, Schauer E, Elbert T. Treatment of posttraumatic stress disorder by trained lay counselors in an African refugee settlement: a randomized controlled trial. J Consult Clin Psychol. 2008 Aug;76(4):686-94. https://doi.org/10.1037/0022-006X.76.4.686
53. Weine S, Kulauzovic Y, Klebic A, Besic S, Mujagic A, Muzurovic J, Spahovic D, Sclove S, Pavkovic I, Feetham S, Rolland J. Evaluating a multiple-family group access intervention for

refugees with PTSD. J Marital Fam Ther. 2008 Apr;34(2):149-64. https://doi.org/10.1111/j.1752-0606.2008.00061.x
54. Johnson H, Thompson A. The development and maintenance of post-traumatic stress disorder (PTSD) in civilian adult survivors of war trauma and torture: a review. Clin Psychol Rev. 2008 Jan;28(1):36-47. Epub 2007 Feb 22. https://doi.org/10.1016/j.cpr.2007.01.017
55. Raphael B, Taylor M, McAndrew V. Women, catastrophe and mental health. Aust N Z J Psychiatry. 2008 Jan;42(1):13-23. https://doi.org/10.1080/00048670701732707
56. Norredam M, Garcia-Lopez A, Keiding N, Krasnik A. Risk of mental disorders in refugees and native Danes: a register-based retrospective cohort study. Soc Psychiatry Psychiatr Epidemiol. 2009 Dec;44(12):1023-9. Epub 2009 Mar 18. https://doi.org/10.1007/s00127-009-0024-6
57. Boynton L, Bentley J, Strachan E, Barbato A, Raskind M. Preliminary findings concerning the use of prazosin for the treatment of posttraumatic nightmares in a refugee population. J Psychiatr Pract. 2009 Nov;15(6):454-9. https://doi.org/10.1097/01.pra.0000364287.63210.92
58. Hollifield M, Warner TD, Krakow B, Jenkins J, Westermeyer J. The range of symptoms in refugees of war: the New Mexico Refugee Symptom Checklist-121. J Nerv Ment Dis. 2009 Feb;197(2):117-25. https://doi.org/10.1097/NMD.0b013e31819642dc
59. Cloitre M. Effective psychotherapies for posttraumatic stress disorder: a review and critique. CNS Spectr. 2009 Jan;14(1 Suppl 1):32-43.
60. Steel Z, Chey T, Silove D, Marnane C, Bryant RA, van Ommeren M. Association of torture and other potentially traumatic events with mental health outcomes among populations exposed to mass conflict and displacement: a systematic review and meta-analysis. JAMA. 2009 Aug 5;302(5):537-49. https://doi.org/10.1001/jama.2009.1132
61. Al-Krenawi A, Graham JR, Kanat-Maymon Y. Analysis of trauma exposure, symptomatology and functioning in Jewish Israeli and Palestinian adolescents. Br J Psychiatry. 2009 Nov;195(5):427-32. https://doi.org/10.1192/bjp.bp.108.050393
62. Mollica RF, Lyoo IK, Chernoff MC, Bui HX, Lavelle J, Yoon SJ, Kim JE, Renshaw PF. Brain structural abnormalities and mental health sequelae in South Vietnamese ex-political detainees who survived traumatic head injury and torture. Arch Gen Psychiatry. 2009 Nov;66(11):1221-32. https://doi.org/10.1001/archgenpsychiatry.2009.127
63. Murray KE, Davidson GR, Schweitzer RD. Review of refugee mental health interventions following resettlement: best practices and recommendations. Am J Orthopsychiatry. 2010 Oct;80(4):576-85. https://doi.org/10.1111/j.1939-0025.2010.01062.x
64. Halvorsen JØ, Stenmark H. Narrative exposure therapy for posttraumatic stress disorder in tortured refugees: a preliminary uncontrolled trial. Scand J Psychol. 2010 Dec;51(6):495-502. https://doi.org/10.1111/j.1467-9450.2010.00821.x
65. Norredam M, Garcia-Lopez A, Keiding N, Krasnik A. Excess use of coercive measures in psychiatry among migrants compared with native Danes. Acta Psychiatr Scand. 2010 Feb;121(2):143-51. Epub 2009 Jul 9. https://doi.org/10.1111/j.1600-0447.2009.01418.x
66. Dobricki M, Komproe IH, de Jong JT, Maercker A. Adjustment disorders after severe life-events in four postconflict settings. Soc Psychiatry Psychiatr Epidemiol. 2010 Jan;45(1):39-46. Epub 2009 Mar 31. https://doi.org/10.1007/s00127-009-0039-z
67. Hollander AC, Bruce D, Burström B, Ekblad S. Gender-related mental health differences between refugees and non-refugee immigrants--a cross-sectional register-based study. BMC Public Health. 2011 Mar 24;11:180. https://doi.org/10.1186/1471-2458-11-180
68. Williams ME, Thompson SC. The use of community-based interventions in reducing morbidity from the psychological impact of conflict-related trauma among refugee

populations: a systematic review of the literature. J Immigr Minor Health. 2011 Aug;13(4):780-94. https://doi.org/10.1007/s10903-010-9417-6

69. Kirmayer LJ, Narasiah L, Munoz M, Rashid M, Ryder AG, Guzder J, Hassan G, Rousseau C, Pottie K; Canadian Collaboration for Immigrant and Refugee Health (CCIRH). Common mental health problems in immigrants and refugees: general approach in primary care. CMAJ. 2011 Sep 6;183(12):E959-67. Epub 2010 Jul 5. https://doi.org/10.1503/cmaj.090292
70. Nickerson A, Bryant RA, Silove D, Steel Z. A critical review of psychological treatments of posttraumatic stress disorder in refugees. Clin Psychol Rev. 2011 Apr;31(3):399-417. Epub 2010 Nov 6. https://doi.org/10.1016/j.cpr.2010.10.004
71. Mueller J, Schmidt M, Staeheli A, Maier T. Mental health of failed asylum seekers as compared with pending and temporarily accepted asylum seekers. Eur J Public Health. 2011 Apr;21(2):184-9. Epub 2010 Jul 14. https://doi.org/10.1093/eurpub/ckq016
72. Mollica RF. Medical best practices for the treatment of torture survivors. Torture. 2011;21(1):8-17.
73. Palic S, Elklit A. Psychosocial treatment of posttraumatic stress disorder in adult refugees: a systematic review of prospective treatment outcome studies and a critique. J Affect Disord. 2011 Jun;131(1-3):8-23. Epub 2010 Aug 13. https://doi.org/10.1016/j.jad.2010.07.005
74. Benedek DM. Posttraumatic stress disorder from Vietnam to today: the evolution of understanding during Eugene Brody's tenure at the journal of nervous and mental disease. J Nerv Ment Dis. 2011 Aug;199(8):544-52. https://doi.org/10.1097/NMD.0b013e318225f0e9
75. Kroll J, Yusuf AI, Fujiwara K. Psychoses, PTSD, and depression in Somali refugees in Minnesota. Soc Psychiatry Psychiatr Epidemiol. 2011 Jun;46(6):481-93. Epub 2010 Mar 31. https://doi.org/10.1007/s00127-010-0216-0
76. Hansson EK, Tuck A, Lurie S, McKenzie K. Rates of mental illness and suicidality in immigrant, refugee, ethnocultural, and racialized groups in Canada: a review of the literature. Can J Psychiatry. 2012 Feb;57(2):111-21. https://doi.org/10.1177/070674371205700208
77. Slewa-Younan S, Chippendale K, Heriseanu A, Lujic S, Atto J, Raphael B. Measures of psychophysiological arousal among resettled traumatized Iraqi refugees seeking psychological treatment. J Trauma Stress. 2012 Jun;25(3):348-52. Epub 2012 Jun 8. https://doi.org/10.1002/jts.21694
78. Kalantari M, Yule W, Dyregrov A, Neshatdoost H, Ahmadi SJ. Efficacy of writing for recovery on traumatic grief symptoms of Afghani refugee bereaved adolescents: a randomized control trial. Omega (Westport). 2012;65(2):139-50. https://doi.org/10.2190/OM.65.2.d
79. Akinyemi OO, Owoaje ET, Ige OK, Popoola OA. Comparative study of mental health and quality of life in long-term refugees and host populations in Oru-Ijebu, Southwest Nigeria. BMC Res Notes. 2012 Jul 31;5:394. https://doi.org/10.1186/1756-0500-5-394
80. Stenmark H, Catani C, Neuner F, Elbert T, Holen A. Treating PTSD in refugees and asylum seekers within the general health care system. A randomized controlled multicenter study. Behav Res Ther. 2013 Oct;51(10):641-7. Epub 2013 Jul 8. https://doi.org/10.1016/j.brat.2013.07.002
81. Morina N, Ehring T, Priebe S. Diagnostic utility of the impact of event scale-revised in two samples of survivors of war. PLoS One. 2013 Dec 31;8(12):e83916. eCollection 2013. https://doi.org/10.1371/journal.pone.0083916
82. Gwozdziewycz N, Mehl-Madrona L. Meta-analysis of the use of narrative exposure therapy for the effects of trauma among refugee populations. Perm J. 2013 Winter;17(1):70-6. https://doi.org/10.7812/TPP/12-058

83. Arnetz J, Rofa Y, Arnetz B, Ventimiglia M, Jamil H. Resilience as a protective factor against the development of psychopathology among refugees. J Nerv Ment Dis. 2013 Mar;201(3):167-72. https://doi.org/10.1097/NMD.0b013e3182848afe
84. Patel N, Kellezi B, Williams AC. Psychological, social and welfare interventions for psychological health and well-being of torture survivors. Cochrane Database Syst Rev. 2014 Nov 11;2014(11):CD009317. https://doi.org/10.1002/14651858.CD009317.pub2
85. Siriwardhana C, Ali SS, Roberts B, Stewart R. A systematic review of resilience and mental health outcomes of conflict-driven adult forced migrants. Confl Health. 2014 Aug 20;8:13. eCollection 2014. https://doi.org/10.1186/1752-1505-8-13
86. Rohlof HG, Knipscheer JW, Kleber RJ. Somatization in refugees: a review. Soc Psychiatry Psychiatr Epidemiol. 2014 Nov;49(11):1793-804. Epub 2014 May 11. https://doi.org/10.1007/s00127-014-0877-1
87. Al-Modallal H, Hamaideh S, Mudallal R. Mental health status of women in Jordan: a comparative study between attendees of governmental and UN relief and works agency's health care centers. Issues Ment Health Nurs. 2014 May;35(5):386-94. https://doi.org/10.3109/01612840.2013.807449
88. Palic S, Kappel ML, Nielsen MS, Carlsson J, Bech P. Comparison of psychiatric disability on the health of nation outcome scales (HoNOS) in resettled traumatized refugee outpatients and Danish inpatients. BMC Psychiatry. 2014 Dec 18;14:330. https://doi.org/10.1186/s12888-014-0330-8
89. Borneman T. Spiritual Assessment in a Patient With Lung Cancer. J Adv Pract Oncol. 2014 Nov-Dec;5(6):448-53.
90. Park YS, Park SM, Jun JY, Kim SJ. Psychiatry in former socialist countries: implications for north korean psychiatry. Psychiatry Investig. 2014 Oct;11(4):363-70. Epub 2014 Oct 20. https://doi.org/10.4306/pi.2014.11.4.363
91. Piwowarczyk L, Bishop H, Yusuf A, Mudymba F, Raj A. Congolese and Somali beliefs about mental health services. J Nerv Ment Dis. 2014 Mar;202(3):209-16. https://doi.org/10.1097/NMD.0000000000000087
92. Bolton P, Bass JK, Zangana GA, Kamal T, Murray SM, Kaysen D, Lejuez CW, Lindgren K, Pagoto S, Murray LK, Van Wyk SS, Ahmed AM, Amin NM, Rosenblum M. A randomized controlled trial of mental health interventions for survivors of systematic violence in Kurdistan, Northern Iraq. BMC Psychiatry. 2014 Dec 31;14:360. https://doi.org/10.1186/s12888-014-0360-2
93. Amawi N, Mollica RF, Lavelle J, Osman O, Nasir L. Overview of research on the mental health impact of violence in the Middle East in light of the Arab Spring. J Nerv Ment Dis. 2014 Sep;202(9):625-9. https://doi.org/10.1097/NMD.0000000000000174
94. Mollica RF, Brooks R, Tor S, Lopes-Cardozo B, Silove D. The enduring mental health impact of mass violence: a community comparison study of Cambodian civilians living in Cambodia and Thailand. Int J Soc Psychiatry. 2014 Feb;60(1):6-20. Epub 2013 Feb 7. https://doi.org/10.1177/0020764012471597
95. Dalgaard NT, Montgomery E. Disclosure and silencing: A systematic review of the literature on patterns of trauma communication in refugee families. Transcult Psychiatry. 2015 Oct;52(5):579-93. Epub 2015 Feb 5. https://doi.org/10.1177/1363461514568442
96. Bell SA, Lori J, Redman R, Seng J. Psychometric Validation and Comparison of the Self-Reporting Questionnaire-20 and Self-Reporting Questionnaire-Suicidal Ideation and Behavior Among Congolese Refugee Women. J Nurs Meas. 2015;23(3):393-408. https://doi.org/10.1891/1061-3749.23.3.393

97. Rasmussen A, Verkuilen J, Ho E, Fan Y. Posttraumatic stress disorder among refugees: Measurement invariance of Harvard Trauma Questionnaire scores across global regions and response patterns. Psychol Assess. 2015 Dec;27(4):1160-70. Epub 2015 Apr 20. https://doi.org/10.1037/pas0000115
98. Akinyemi OO, Atilola O, Soyannwo T. Suicidal ideation: Are refugees more at risk compared to host population? Findings from a preliminary assessment in a refugee community in Nigeria. Asian J Psychiatr. 2015 Dec;18:81-5. Epub 2015 Sep 21. https://doi.org/10.1016/j.ajp.2015.09.001
99. Schnyder U, Müller J, Morina N, Schick M, Bryant RA, Nickerson A. A Comparison of DSM-5 and DSM-IV Diagnostic Criteria for Posttraumatic Stress Disorder in Traumatized Refugees. J Trauma Stress. 2015 Aug;28(4):267-74. Epub 2015 Jul 20. https://doi.org/10.1002/jts.22023
100. Jha B, Seavy J, Young D, Bonner A. Positive Mental Health Outcomes in Individuals with Dementia: The Essential Role of Cultural Competence. Online J Issues Nurs. 2015 Jan 31;20(1):5.
101. Richter K, Lehfeld H, Niklewski G. [Waiting for Asylum: Psychiatric Diagnosis in Bavarian Admission Center]. Gesundheitswesen. 2015 Nov;77(11):834-8. Epub 2015 Sep 25. https://doi.org/10.1055/s-0035-1564075
102. Tierney D, Bolton P, Matanu B, Garasu L, Barnabas E, Silove D. The aftermath of the Bougainville Crisis: Mental health and psychosocial impacts and the need for services. Aust N Z J Psychiatry. 2015 May;49(5):481-2. Epub 2015 Mar 10. https://doi.org/10.1177/0004867415575381
103. Sigvardsdotter E, Malm A, Tinghög P, Vaez M, Saboonchi F. Refugee trauma measurement: a review of existing checklists. Public Health Rev. 2016 Sep 9;37:10. eCollection 2016. https://doi.org/10.1186/s40985-016-0024-5
104. Li SS, Liddell BJ, Nickerson A. The Relationship Between Post-Migration Stress and Psychological Disorders in Refugees and Asylum Seekers. Curr Psychiatry Rep. 2016 Sep;18(9):82. https://doi.org/10.1007/s11920-016-0723-0
105. Acarturk C, Konuk E, Cetinkaya M, Senay I, Sijbrandij M, Gulen B, Cuijpers P. The efficacy of eye movement desensitization and reprocessing for post-traumatic stress disorder and depression among Syrian refugees: results of a randomized controlled trial. Psychol Med. 2016 Sep;46(12):2583-93. Epub 2016 Jun 29. https://doi.org/10.1017/S0033291716001070
106. Sonne C, Carlsson J, Bech P, Elklit A, Mortensen EL. Treatment of trauma-affected refugees with venlafaxine versus sertraline combined with psychotherapy - a randomised study. BMC Psychiatry. 2016 Nov 8;16(1):383. https://doi.org/10.1186/s12888-016-1081-5
107. Horyniak D, Melo JS, Farrell RM, Ojeda VD, Strathdee SA. Epidemiology of Substance Use among Forced Migrants: A Global Systematic Review. PLoS One. 2016 Jul 13;11(7):e0159134. eCollection 2016. https://doi.org/10.1371/journal.pone.0159134
108. Murthy RS. Psychosocial and behavioral aspects of populations affected by humanitarian emergencies: recent developments. Curr Opin Psychiatry. 2016 Sep;29(5):280-5. https://doi.org/10.1097/YCO.0000000000000266
109. Stompe T, Ritter K, Holzer D, Topitz A, Wenzel T. [Alcohol- and substance abuse among mentally ill patients with migration background in Austria]. Neuropsychiatr. 2016 Sep;30(3):138-144. Epub 2016 Sep 28. https://doi.org/10.1007/s40211-016-0192-z
110. Ter Heide FJ, Mooren TM, van de Schoot R, de Jongh A, Kleber RJ. Eye movement desensitisation and reprocessing therapy v. stabilisation as usual for refugees: randomised

controlled trial. Br J Psychiatry. 2016 Oct;209(4):311-318. Epub 2016 Feb 18. https://doi.org/10.1192/bjp.bp.115.167775
111. Tierney D, Bolton P, Matanu B, Garasu L, Barnabas E, Silove D. The mental health and psychosocial impact of the Bougainville Crisis: a synthesis of available information. Int J Ment Health Syst. 2016 Mar 3;10:18. eCollection 2016. https://doi.org/10.1186/s13033-016-0054-x
112. Bass J, Murray SM, Mohammed TA, Bunn M, Gorman W, Ahmed AM, Murray L, Bolton P. A Randomized Controlled Trial of a Trauma-Informed Support, Skills, and Psychoeducation Intervention for Survivors of Torture and Related Trauma in Kurdistan, Northern Iraq. Glob Health Sci Pract. 2016 Sep 29;4(3):452-66. Print 2016 Sep 28. https://doi.org/10.9745/GHSP-D-16-00017
113. Dapunt J, Kluge U, Heinz A. Risk of psychosis in refugees: a literature review. Transl Psychiatry. 2017 Jun 13;7(6):e1149. https://doi.org/10.1038/tp.2017.119
114. Timshel I, Montgomery E, Dalgaard NT. A systematic review of risk and protective factors associated with family related violence in refugee families. Child Abuse Negl. 2017 Aug;70:315-330. Epub 2017 Jul 3. https://doi.org/10.1016/j.chiabu.2017.06.023
115. Nejad RM, Klöhn-Saghatolislam F, Hasan A, Pogarell O. [Mental disorders and problems in afghan refugees: The clinical perspective]. MMW Fortschr Med. 2017 May;159(9):64-66. https://doi.org/10.1007/s15006-017-9653-y
116. Kaur G. Chronic pain in refugee torture survivors. J Glob Health. 2017 Dec;7(2):010303. https://doi.org/10.7189/jogh.07.020303
117. Haagen JF, Ter Heide FJ, Mooren TM, Knipscheer JW, Kleber RJ. Predicting post-traumatic stress disorder treatment response in refugees: Multilevel analysis. Br J Clin Psychol. 2017 Mar;56(1):69-83. Epub 2016 Nov 30. https://doi.org/10.1111/bjc.12121
118. Anagnostopoulos DC, Giannakopoulos G, Christodoulou NG. The synergy of the refugee crisis and the financial crisis in Greece: Impact on mental health. Int J Soc Psychiatry. 2017 Jun;63(4):352-358. Epub 2017 Mar 29. https://doi.org/10.1177/0020764017700444
119. Ter Heide FJJ, Sleijpen M, van der Aa N. Posttraumatic world assumptions among treatment-seeking refugees. Transcult Psychiatry. 2017 Oct-Dec;54(5-6):824-839. https://doi.org/10.1177/1363461517741811
120. Lee Y, Lee M, Park S. Mental health status of North Korean refugees in South Korea and risk and protective factors: a 10-year review of the literature. Eur J Psychotraumatol. 2017 Sep 4;8(sup2):1369833. eCollection 2017. https://doi.org/10.1080/20008198.2017.1369833
121. Sandahl H, Vindbjerg E, Carlsson J. Treatment of sleep disturbances in refugees suffering from post-traumatic stress disorder. Transcult Psychiatry. 2017 Oct-Dec;54(5-6):806-823. https://doi.org/10.1177/1363461517746314
122. Roura M. Unravelling migrants' health paradoxes: a transdisciplinary research agenda. J Epidemiol Community Health. 2017 Jul 24:jech-2016-208439. Online ahead of print. https://doi.org/10.1136/jech-2016-208439
123. Coulter L, Ibrahimi M, Patel R, Agius M. Linking the psychosocial aetiology and neurobiology of unipolar depression. Psychiatr Danub. 2017 Sep;29(Suppl 3):441-446.
124. Adorjan K, Mulugeta S, Odenwald M, Ndetei DM, Osman AH, Hautzinger M, Wolf S, Othman M, Kizilhan JI, Pogarell O, Schulze TG. [Psychiatric care of refugees in Africa and the Middle East : Challenges and solutions]. Nervenarzt. 2017 Sep;88(9):974-982. https://doi.org/10.1007/s00115-017-0365-4
125. Nocon A, Eberle-Sejari R, Unterhitzenberger J, Rosner R. The effectiveness of psychosocial interventions in war-traumatized refugee and internally displaced minors:

systematic review and meta-analysis. Eur J Psychotraumatol. 2017 Nov 7;8(sup2):1388709. eCollection 2017. https://doi.org/10.1080/20008198.2017.1388709
126. Kahl F, Frewer A. [Medical Treatment of Newly Arrived Refugees in Erlangen: A Study of Drug Prescription Rates Focused on Psychotropic Drugs]. Psychother Psychosom Med Psychol. 2017 Apr;67(3-04):119-125. Epub 2016 Oct 17. https://doi.org/10.1055/s-0042-116325
127. Sandahl H, Jennum P, Baandrup L, Poschmann IS, Carlsson J. Treatment of sleep disturbances in trauma-affected refugees: Study protocol for a randomised controlled trial. Trials. 2017 Nov 6;18(1):520. https://doi.org/10.1186/s13063-017-2260-5
128. Sijbrandij M, Acarturk C, Bird M, Bryant RA, Burchert S, Carswell K, de Jong J, Dinesen C, Dawson KS, El Chammay R, van Ittersum L, Jordans M, Knaevelsrud C, McDaid D, Miller K, Morina N, Park AL, Roberts B, van Son Y, Sondorp E, Pfaltz MC, Ruttenberg L, Schick M, Schnyder U, van Ommeren M, Ventevogel P, Weissbecker I, Weitz E, Wiedemann N, Whitney C, Cuijpers P. Strengthening mental health care systems for Syrian refugees in Europe and the Middle East: integrating scalable psychological interventions in eight countries. Eur J Psychotraumatol. 2017 Nov 7;8(sup2):1388102. eCollection 2017. https://doi.org/10.1080/20008198.2017.1388102
129. Thompson CT, Vidgen A, Roberts NP. Psychological interventions for post-traumatic stress disorder in refugees and asylum seekers: A systematic review and meta-analysis. Clin Psychol Rev. 2018 Jul;63:66-79. Epub 2018 Jun 15. https://doi.org/10.1016/j.cpr.2018.06.006
130. Tuomisto MT, Roche JE. Beyond PTSD and Fear-Based Conditioning: Anger-Related Responses Following Experiences of Forced Migration-A Systematic Review. Front Psychol. 2018 Dec 19;9:2592. eCollection 2018. https://doi.org/10.3389/fpsyg.2018.02592
131. Morina N, Akhtar A, Barth J, Schnyder U. Psychiatric Disorders in Refugees and Internally Displaced Persons After Forced Displacement: A Systematic Review. Front Psychiatry. 2018 Sep 21;9:433. eCollection 2018. https://doi.org/10.3389/fpsyt.2018.00433
132. Hameed S, Sadiq A, Din AU. The Increased Vulnerability of Refugee Population to Mental Health Disorders. Kans J Med. 2018 Feb 28;11(1):1-12. eCollection 2018 Feb.
133. Kronick R. Mental Health of Refugees and Asylum Seekers: Assessment and Intervention. Can J Psychiatry. 2018 May;63(5):290-296. Epub 2017 Dec 5. https://doi.org/10.1177/0706743717746665
134. Miller A, Hess JM, Bybee D, Goodkind JR. Understanding the mental health consequences of family separation for refugees: Implications for policy and practice. Am J Orthopsychiatry. 2018;88(1):26-37. Epub 2017 Jun 15. https://doi.org/10.1037/ort0000272
135. Killikelly C, Bauer S, Maercker A. The Assessment of Grief in Refugees and Post-conflict Survivors: A Narrative Review of Etic and Emic Research. Front Psychol. 2018 Oct 22;9:1957. eCollection 2018. https://doi.org/10.3389/fpsyg.2018.01957
136. Wylie L, Van Meyel R, Harder H, Sukhera J, Luc C, Ganjavi H, Elfakhani M, Wardrop N. Assessing trauma in a transcultural context: challenges in mental health care with immigrants and refugees. Public Health Rev. 2018 Aug 22;39:22. eCollection 2018. https://doi.org/10.1186/s40985-018-0102-y
137. Bäärnhielm S, Sundvall M. Clinical challenges in cultural psychiatry - searching for meaning, searching for methods(†). Nord J Psychiatry. 2018 Sep;72(sup1):S9-S12. https://doi.org/10.1080/08039488.2018.1525648

138. Koesters M, Barbui C, Purgato M. Recent approaches to provision of mental healthcare in refugee populations. Curr Opin Psychiatry. 2018 Jul;31(4):368-372. https://doi.org/10.1097/YCO.0000000000000428
139. Lindert J, von Ehrenstein OS, Wehrwein A, Brähler E, Schäfer I. [Anxiety, Depression and Posttraumatic Stress Disorder in Refugees - A Systematic Review]. Psychother Psychosom Med Psychol. 2018 Jan;68(1):22-29. Epub 2017 May 3. https://doi.org/10.1055/s-0043-103344
140. Greene MC, Kane JC, Khoshnood K, Ventevogel P, Tol WA. Challenges and opportunities for implementation of substance misuse interventions in conflict-affected populations. Harm Reduct J. 2018 Nov 28;15(1):58. https://doi.org/10.1186/s12954-018-0267-1
141. Beck BD, Lund ST, Søgaard U, Simonsen E, Tellier TC, Cordtz TO, Laier GH, Moe T. Music therapy versus treatment as usual for refugees diagnosed with posttraumatic stress disorder (PTSD): study protocol for a randomized controlled trial. Trials. 2018 May 30;19(1):301. https://doi.org/10.1186/s13063-018-2662-z
142. Panter-Brick C, Dajani R, Eggerman M, Hermosilla S, Sancilio A, Ager A. Insecurity, distress and mental health: experimental and randomized controlled trials of a psychosocial intervention for youth affected by the Syrian crisis. J Child Psychol Psychiatry. 2018 May;59(5):523-541. Epub 2017 Oct 2. https://doi.org/10.1111/jcpp.12832
143. Kohrt BA, Asher L, Bhardwaj A, Fazel M, Jordans MJD, Mutamba BB, Nadkarni A, Pedersen GA, Singla DR, Patel V. The Role of Communities in Mental Health Care in Low- and Middle-Income Countries: A Meta-Review of Components and Competencies. Int J Environ Res Public Health. 2018 Jun 16;15(6):1279. https://doi.org/10.3390/ijerph15061279
144. Frost CJ, Morgan NJ, Allkhenfr H, Dearden S, Ess R, Albalawi WF, Berri A, Benson LS, Gren LH. Determining Physical and Mental Health Conditions Present in Older Adult Refugees: A Mini-Review. Gerontology. 2019;65(3):209-215. Epub 2018 Aug 21. https://doi.org/10.1159/000491695
145. Loomis AM, Berthold SM, Buckley T, Wagner J, Kuoch T. Integrated Health Care and mHealth: A Model of Care for Refugees with Complex Health Conditions. Soc Work Public Health. 2019;34(2):189-200. Epub 2019 Feb 18. https://doi.org/10.1080/19371918.2019.1575311
146. Khosa M, Bhulani N, Ali AA, Singh J, Khosa F, Nasrullah M. Bibliometrics of Fifty Most-Cited Articles on the Mental Health of Immigrants Living in the United States. J Immigr Minor Health. 2019 Apr;21(2):414-429. https://doi.org/10.1007/s10903-018-0778-6
147. Brandt L, Henssler J, Müller M, Wall S, Gabel D, Heinz A. Risk of Psychosis Among Refugees: A Systematic Review and Meta-analysis. JAMA Psychiatry. 2019 Nov 1;76(11):1133-1140. https://doi.org/10.1001/jamapsychiatry.2019.1937
148. Shaw SA, Ward KP, Pillai V, Hinton DE. A group mental health randomized controlled trial for female refugees in Malaysia. Am J Orthopsychiatry. 2019;89(6):665-674. Epub 2018 Jul 23. https://doi.org/10.1037/ort0000346
149. Bayne M, Sokoloff L, Rinehart R, Epie A, Hirt L, Katz C. Assessing the efficacy and experience of in-person versus telephonic psychiatric evaluations for asylum seekers in the U.S. Psychiatry Res. 2019 Dec;282:112612. Epub 2019 Oct 11. https://doi.org/10.1016/j.psychres.2019.112612
150. Derlet O, Deschietere G. Providing psychiatric healthcare to asylum seekers: reflections and challenges. Psychiatr Danub. 2019 Sep;31(Suppl 3):395-399.
151. Wulfes N, Del Pozo MA, Buhr-Riehm B, Heinrichs N, Kröger C. Screening for Posttraumatic Stress Disorder in Refugees: Comparison of the Diagnostic Efficiency of Two

Self-Rating Measures of Posttraumatic Stress Disorder. J Trauma Stress. 2019 Feb;32(1):148-155. Epub 2019 Jan 29. https://doi.org/10.1002/jts.22358

152. Hotzy F, Hengartner MP, Hoff P, Jaeger M, Theodoridou A. Clinical and socio-demographic characteristics associated with involuntary admissions in Switzerland between 2008 and 2016: An observational cohort study before and after implementation of the new legislation. Eur Psychiatry. 2019 Jun;59:70-76. Epub 2019 May 10. https://doi.org/10.1016/j.eurpsy.2019.04.004

153. Kluge U, Rapp MA, Mehran N, Jumaa J, Aichberger MC. [Poverty, migration and mental health]. Nervenarzt. 2019 Nov;90(11):1103-1108. https://doi.org/10.1007/s00115-019-00790-2

154. Newnham EA, Pearman A, Olinga-Shannon S, Nickerson A. The mental health effects of visa insecurity for refugees and people seeking asylum: a latent class analysis. Int J Public Health. 2019 Jun;64(5):763-772. Epub 2019 May 28. https://doi.org/10.1007/s00038-019-01249-6

155. Nygren T, Brohede D, Koshnaw K, Osman SS, Johansson R, Andersson G. Internet-based treatment of depressive symptoms in a Kurdish population: A randomized controlled trial. J Clin Psychol. 2019 Jun;75(6):985-998. Epub 2019 Jan 31. https://doi.org/10.1002/jclp.22753

156. Sangalang CC, Becerra D, Mitchell FM, Lechuga-Peña S, Lopez K, Kim I. Trauma, Post-Migration Stress, and Mental Health: A Comparative Analysis of Refugees and Immigrants in the United States. J Immigr Minor Health. 2019 Oct;21(5):909-919. https://doi.org/10.1007/s10903-018-0826-2

157. Hasha W, Fadnes LT, Igland J, Vårdal R, Giusti LM, Strømme EM, Haj-Younes J, Heltne U, Kumar BN, Diaz E. Two interventions to treat pain disorders and post-traumatic symptoms among Syrian refugees: protocol for a randomized controlled trial. Trials. 2019 Dec 27;20(1):784. https://doi.org/10.1186/s13063-019-3919-x

158. Hassan A, Sharif K. Efficacy of Telepsychiatry in Refugee Populations: A Systematic Review of the Evidence. Cureus. 2019 Jan 30;11(1):e3984. https://doi.org/10.7759/cureus.3984

159. Tay AK, Riley A, Islam R, Welton-Mitchell C, Duchesne B, Waters V, Varner A, Moussa B, Mahmudul Alam ANM, Elshazly MA, Silove D, Ventevogel P. The culture, mental health and psychosocial wellbeing of Rohingya refugees: a systematic review. Epidemiol Psychiatr Sci. 2019 Oct;28(5):489-494. Epub 2019 Apr 22. https://doi.org/10.1017/S2045796019000192

160. Rousseau C, Frounfelker RL. Mental health needs and services for migrants: an overview for primary care providers. J Travel Med. 2019 Feb 1;26(2):tay150. https://doi.org/10.1093/jtm/tay150

161. Turrini G, Purgato M, Acarturk C, Anttila M, Au T, Ballette F, Bird M, Carswell K, Churchill R, Cuijpers P, Hall J, Hansen LJ, Kösters M, Lantta T, Nosè M, Ostuzzi G, Sijbrandij M, Tedeschi F, Valimaki M, Wancata J, White R, van Ommeren M, Barbui C. Efficacy and acceptability of psychosocial interventions in asylum seekers and refugees: systematic review and meta-analysis. Epidemiol Psychiatr Sci. 2019 Aug;28(4):376-388. Epub 2019 Feb 11. https://doi.org/10.1017/S2045796019000027

162. Golchert J, Roehr S, Berg F, Grochtdreis T, Hoffmann R, Jung F, Nagl M, Plexnies A, Renner A, König HH, Kersting A, Riedel-Heller SG. HELP@APP: development and evaluation of a self-help app for traumatized Syrian refugees in Germany - a study protocol of a randomized controlled trial. BMC Psychiatry. 2019 Apr 30;19(1):131. https://doi.org/10.1186/s12888-019-2110-y

163. Wirth T, Mette J, Prill J, Harth V, Nienhaus A. Working conditions, mental health and coping of staff in social work with refugees and homeless individuals: A scoping review. Health Soc Care Community. 2019 Jul;27(4):e257-e269. Epub 2019 Mar 1. https://doi.org/10.1111/hsc.12730
164. Edwards J, Hu M, Thind A, Stranges S, Chiu M, Anderson KK. Gaps in Understanding of the Epidemiology of Mood and Anxiety Disorders among Migrant Groups in Canada: A Systematic Review. Can J Psychiatry. 2019 Sep;64(9):595-606. Epub 2019 May 26. https://doi.org/10.1177/0706743719839313
165. Richter-Levin G, Stork O, Schmidt MV. Animal models of PTSD: a challenge to be met. Mol Psychiatry. 2019 Aug;24(8):1135-1156. Epub 2018 Oct 19. https://doi.org/10.1038/s41380-018-0272-5
166. Araujo JO, Souza FM, Proença R, Bastos ML, Trajman A, Faerstein E. Prevalence of sexual violence among refugees: a systematic review. Rev Saude Publica. 2019 Sep 23;53:78. eCollection 2019. https://doi.org/10.11606/s1518-8787.2019053001081
167. McLaughlin KA, Alvarez K, Fillbrunn M, Green JG, Jackson JS, Kessler RC, Sadikova E, Sampson NA, Vilsaint CL, Williams DR, Alegría M. Racial/ethnic variation in trauma-related psychopathology in the United States: a population-based study. Psychol Med. 2019 Oct;49(13):2215-2226. Epub 2018 Oct 31. https://doi.org/10.1017/S0033291718003082
168. Byrow Y, Pajak R, Specker P, Nickerson A. Perceptions of mental health and perceived barriers to mental health help-seeking amongst refugees: A systematic review. Clin Psychol Rev. 2020 Feb;75:101812. Epub 2019 Dec 24. https://doi.org/10.1016/j.cpr.2019.101812
169. Flanagan N, Travers A, Vallières F, Hansen M, Halpin R, Sheaf G, Rottmann N, Johnsen AT. Crossing borders: a systematic review identifying potential mechanisms of intergenerational trauma transmission in asylum-seeking and refugee families. Eur J Psychotraumatol. 2020 Sep 23;11(1):1790283. https://doi.org/10.1080/20008198.2020.1790283
170. Peconga EK, Høgh Thøgersen M. Post-traumatic stress disorder, depression, and anxiety in adult Syrian refugees: What do we know?. Scand J Public Health. 2020 Nov;48(7):677-687. Epub 2019 Dec 8. https://doi.org/10.1177/1403494819882137
171. Jannesari S, Hatch S, Prina M, Oram S. Post-migration Social-Environmental Factors Associated with Mental Health Problems Among Asylum Seekers: A Systematic Review. J Immigr Minor Health. 2020 Oct;22(5):1055-1064. https://doi.org/10.1007/s10903-020-01025-2
172. Sambucini D, Aceto P, Begotaraj E, Lai C. Efficacy of Psychological Interventions on Depression Anxiety and Somatization in Migrants: A Meta-analysis. J Immigr Minor Health. 2020 Dec;22(6):1320-1346. https://doi.org/10.1007/s10903-020-01055-w
173. Kokou-Kpolou CK, Moukouta CS, Masson J, Bernoussi A, Cénat JM, Bacqué MF. Correlates of grief-related disorders and mental health outcomes among adult refugees exposed to trauma and bereavement: A systematic review and future research directions. J Affect Disord. 2020 Apr 15;267:171-184. Epub 2020 Feb 11. https://doi.org/10.1016/j.jad.2020.02.026
174. Hedrick K, Armstrong G, Coffey G, Borschmann R. Temporal variations in the distribution of self-harm episodes and methods across the Australian asylum seeker population: An observational study. PLoS Med. 2020 Aug 6;17(8):e1003235. eCollection 2020 Aug. https://doi.org/10.1371/journal.pmed.1003235
175. Peterson C, Poudel-Tandukar K, Sanger K, Jacelon CS. Improving Mental Health in Refugee Populations: A Review of Intervention Studies Conducted in the United States.

Issues Ment Health Nurs. 2020 Apr;41(4):271-282. Epub 2020 Jan 30. https://doi.org/10.1080/01612840.2019.1669748

176. Jumaa JA, Kluge U, Weigold S, Heinz E, Mehran N. [Peer-to-Peer Self-help Interventions for Refugees: A Pilot Study]. Fortschr Neurol Psychiatr. 2020 Feb;88(2):89-94. Epub 2020 Feb 26. https://doi.org/10.1055/a-1011-4232

177. Gutknecht S, Kaiser F, Leiding DV, Schneider F, Habel U, Schulte Holthausen B. [Evaluation of a peer-helper project for mental health stabilization of refugees]. Fortschr Neurol Psychiatr. 2020 Feb;88(2):82-88. Epub 2020 Feb 26. https://doi.org/10.1055/a-1090-7375

178. Haroz EE, Decker E, Lee C, Bolton P, Spiegel P, Ventevogel P. Evidence for suicide prevention strategies with populations in displacement: a systematic review. Intervention (Amstelveen). 2020 Jan-Jun;18(1):37-44. Epub 2019 May 29.

179. Akhtar A, Giardinelli L, Bawaneh A, Awwad M, Naser H, Whitney C, Jordans MJD, Sijbrandij M, Bryant RA; STRENGTHS Consortium. Group problem management plus (gPM+) in the treatment of common mental disorders in Syrian refugees in a Jordanian camp: study protocol for a randomized controlled trial. BMC Public Health. 2020 Mar 26;20(1):390. https://doi.org/10.1186/s12889-020-08463-5

180. Slewa-Younan S, McKenzie M, Thomson R, Smith M, Mohammad Y, Mond J. Improving the mental wellbeing of Arabic speaking refugees: an evaluation of a mental health promotion program. BMC Psychiatry. 2020 Jun 18;20(1):314. https://doi.org/10.1186/s12888-020-02732-8

181. Nickerson A, Byrow Y, Pajak R, McMahon T, Bryant RA, Christensen H, Liddell BJ. 'Tell Your Story': a randomized controlled trial of an online intervention to reduce mental health stigma and increase help-seeking in refugee men with posttraumatic stress. Psychol Med. 2020 Apr;50(5):781-792. Epub 2019 Apr 11. https://doi.org/10.1017/S0033291719000606

182. Gleeson C, Frost R, Sherwood L, Shevlin M, Hyland P, Halpin R, Murphy J, Silove D. Post-migration factors and mental health outcomes in asylum-seeking and refugee populations: a systematic review. Eur J Psychotraumatol. 2020 Dec 1;11(1):1793567. https://doi.org/10.1080/20008198.2020.1793567

183. Henkelmann JR, de Best S, Deckers C, Jensen K, Shahab M, Elzinga B, Molendijk M. Anxiety, depression and post-traumatic stress disorder in refugees resettling in high-income countries: systematic review and meta-analysis. BJPsych Open. 2020 Jul 2;6(4):e68. https://doi.org/10.1192/bjo.2020.54

184. Solberg MA, Peters RM. Adverse Childhood Experiences in Non-Westernized Nations: Implications for Immigrant and Refugee Health. J Immigr Minor Health. 2020 Feb;22(1):145-155. https://doi.org/10.1007/s10903-019-00953-y

185. Miller KE, Koppenol-Gonzalez GV, Arnous M, Tossyeh F, Chen A, Nahas N, Jordans MJD. Supporting Syrian families displaced by armed conflict: A pilot randomized controlled trial of the Caregiver Support Intervention. Child Abuse Negl. 2020 Aug;106:104512. Epub 2020 May 11. https://doi.org/10.1016/j.chiabu.2020.104512

186. Northwood AK, Vukovich MM, Beckman A, Walter JP, Josiah N, Hudak L, O'Donnell Burrows K, Letts JP, Danner CC. Intensive psychotherapy and case management for Karen refugees with major depression in primary care: a pragmatic randomized control trial. BMC Fam Pract. 2020 Jan 28;21(1):17. https://doi.org/10.1186/s12875-020-1090-9

187. Betancourt TS, Berent JM, Freeman J, Frounfelker RL, Brennan RT, Abdi S, Maalim A, Abdi A, Mishra T, Gautam B, Creswell JW, Beardslee WR. Family-Based Mental Health Promotion for Somali Bantu and Bhutanese Refugees: Feasibility and Acceptability Trial. J

Adolesc Health. 2020 Mar;66(3):336-344. Epub 2019 Nov 5. https://doi.org/10.1016/j.jadohealth.2019.08.023

188. Ng E, Zhang H. The mental health of immigrants and refugees: Canadian evidence from a nationally linked database. Health Rep. 2020 Aug 19;31(8):3-12. https://doi.org/10.25318/82-003-x202000800001-eng

189. Jobst S, Windeisen M, Wuensch A, Meng M, Kugler C. Supporting migrants and refugees with posttraumatic stress disorder: development, pilot implementation, and pilot evaluation of a continuing interprofessional education for healthcare providers. BMC Med Educ. 2020 Sep 16;20(1):311. https://doi.org/10.1186/s12909-020-02220-3

190. Heeke C, O'Donald A, Stammel N, Böttche M. Same same but different? DSM-5 versus ICD-11 PTSD among traumatized refugees in Germany. J Psychosom Res. 2020 Jul;134:110129. Epub 2020 May 5. https://doi.org/10.1016/j.jpsychores.2020.110129

191. Matheson K, Asokumar A, Anisman H. Resilience: Safety in the Aftermath of Traumatic Stressor Experiences. Front Behav Neurosci. 2020 Dec 21;14:596919. eCollection 2020. https://doi.org/10.3389/fnbeh.2020.596919

192. Blackmore R, Boyle JA, Fazel M, Ranasinha S, Gray KM, Fitzgerald G, Misso M, Gibson-Helm M. The prevalence of mental illness in refugees and asylum seekers: A systematic review and meta-analysis. PLoS Med. 2020 Sep 21;17(9):e1003337. eCollection 2020 Sep. https://doi.org/10.1371/journal.pmed.1003337

193. van der Boor CF, White R. Barriers to Accessing and Negotiating Mental Health Services in Asylum Seeking and Refugee Populations: The Application of the Candidacy Framework. J Immigr Minor Health. 2020 Feb;22(1):156-174. https://doi.org/10.1007/s10903-019-00929-y

194. Silveira K, Garcia-Barrera MA, Smart CM. Neuropsychological Impact of Trauma-Related Mental Illnesses: A Systematic Review of Clinically Meaningful Results. Neuropsychol Rev. 2020 Sep;30(3):310-344. Epub 2020 Jul 23. https://doi.org/10.1007/s11065-020-09444-6

195. Lee W, Lee YR, Yoon JH, Lee HJ, Kang MY. Occupational post-traumatic stress disorder: an updated systematic review. BMC Public Health. 2020 May 24;20(1):768. https://doi.org/10.1186/s12889-020-08903-2

196. Tol WA, Leku MR, Lakin DP, Carswell K, Augustinavicius J, Adaku A, Au TM, Brown FL, Bryant RA, Garcia-Moreno C, Musci RJ, Ventevogel P, White RG, van Ommeren M. Guided self-help to reduce psychological distress in South Sudanese female refugees in Uganda: a cluster randomised trial. Lancet Glob Health. 2020 Feb;8(2):e254-e263. https://doi.org/10.1016/S2214-109X(19)30504-2

197. Husby SR, Carlsson J, Mathilde Scotte Jensen A, Glahder Lindberg L, Sonne C. Prevention of trauma-related mental health problems among refugees: A mixed-methods evaluation of the MindSpring group programme in Denmark. J Community Psychol. 2020 Apr;48(3):1028-1039. Epub 2020 Feb 6. https://doi.org/10.1002/jcop.22323

198. Michalopoulos LM, Meinhart M, Yung J, Barton SM, Wang X, Chakrabarti U, Ritchey M, Haroz E, Joseph N, Bass J, Bolton P. Global Posttrauma Symptoms: A Systematic Review of Qualitative Literature. Trauma Violence Abuse. 2020 Apr;21(2):406-420. Epub 2018 Apr 26. https://doi.org/10.1177/1524838018772293

199. Tay AK, Mung HK, Miah MAA, Balasundaram S, Ventevogel P, Badrudduza M, Khan S, Morgan K, Rees S, Mohsin M, Silove D. An Integrative Adapt Therapy for common mental health symptoms and adaptive stress amongst Rohingya, Chin, and Kachin refugees living in Malaysia: A randomized controlled trial. PLoS Med. 2020 Mar 31;17(3):e1003073. eCollection 2020 Mar. https://doi.org/10.1371/journal.pmed.1003073

200. Wilker S, Catani C, Wittmann J, Preusse M, Schmidt T, May T, Ertl V, Doering B, Rosner R, Zindler A, Neuner F. The efficacy of Narrative Exposure Therapy for Children (KIDNET) as a treatment for traumatized young refugees versus treatment as usual: study protocol for a multi-center randomized controlled trial (YOURTREAT). Trials. 2020 Feb 14;21(1):185. https://doi.org/10.1186/s13063-020-4127-4
201. Coventry PA, Meader N, Melton H, Temple M, Dale H, Wright K, Cloitre M, Karatzias T, Bisson J, Roberts NP, Brown JVE, Barbui C, Churchill R, Lovell K, McMillan D, Gilbody S. Psychological and pharmacological interventions for posttraumatic stress disorder and comorbid mental health problems following complex traumatic events: Systematic review and component network meta-analysis. PLoS Med. 2020 Aug 19;17(8):e1003262. eCollection 2020 Aug. https://doi.org/10.1371/journal.pmed.1003262
202. Rawlinson R, Aslam RW, Burnside G, Chiumento A, Eriksson-Lee M, Humphreys A, Khan N, Lawrence D, McCluskey R, Mackinnon A, Orton L, Rahman A, Roberts E, Rosala-Hallas A, Edwards RT, Uwamaliya P, White RG, Winrow E, Dowrick C. Lay-therapist-delivered, low-intensity, psychosocial intervention for refugees and asylum seekers (PROSPER): protocol for a pilot randomised controlled trial. Trials. 2020 Apr 28;21(1):367. https://doi.org/10.1186/s13063-020-04310-5
203. Khan S, Kuhn SK, Haque S. A Systematic Review of Autobiographical Memory and Mental Health Research on Refugees and Asylum Seekers. Front Psychiatry. 2021 Jun 4;12:658700. eCollection 2021. https://doi.org/10.3389/fpsyt.2021.658700
204. Scoglio AAJ, Salhi C. Violence Exposure and Mental Health Among Resettled Refugees: A Systematic Review. Trauma Violence Abuse. 2021 Dec;22(5):1192-1208. Epub 2020 Apr 2. https://doi.org/10.1177/1524838020915584
205. Vallejo-Martín M, Sánchez Sancha A, Canto JM. Refugee Women with a History of Trauma: Gender Vulnerability in Relation to Post-Traumatic Stress Disorder. Int J Environ Res Public Health. 2021 Apr 30;18(9):4806. https://doi.org/10.3390/ijerph18094806
206. van Es CM, Boelen PA, Zwaanswijk M, Te Brake H, Mooren T. Family Empowerment (FAME): A feasibility trial of preventive multifamily groups for asylum seeker families in the Netherlands. J Marital Fam Ther. 2021 Oct;47(4):864-881. Epub 2021 Jul 20. https://doi.org/10.1111/jmft.12539
207. Hawkes C, Norris K, Joyce J, Paton D. Individuals of refugee background resettled in regional and rural Australia: A systematic review of mental health research. Aust J Rural Health. 2021 Dec;29(6):850-864. Epub 2021 Oct 19. https://doi.org/10.1111/ajr.12785
208. Nissen A, Cauley P, Saboonchi F, J Andersen A, Solberg Ø. Mental health in adult refugees from Syria resettled in Norway between 2015 and 2017: a nationwide, questionnaire-based, cross-sectional prevalence study. Eur J Psychotraumatol. 2021 Dec 6;12(1):1994218. eCollection 2021. https://doi.org/10.1080/20008198.2021.1994218
209. Greene MC, Likindikoki S, Rees S, Bonz A, Kaysen D, Misinzo L, Njau T, Kiluwa S, Turner R, Ventevogel P, Mbwambo JKK, Tol WA. Evaluation of an integrated intervention to reduce psychological distress and intimate partner violence in refugees: Results from the Nguvu cluster randomized feasibility trial. PLoS One. 2021 Jun 18;16(6):e0252982. eCollection 2021. https://doi.org/10.1371/journal.pone.0252982
210. Akhtar A, Giardinelli L, Bawaneh A, Awwad M, Al-Hayek H, Whitney C, Jordans MJD, Sijbrandij M, Cuijpers P, Dawson K, Bryant R. Feasibility trial of a scalable transdiagnostic group psychological intervention for Syrians residing in a refugee camp. Eur J Psychotraumatol. 2021 Jun 30;12(1):1932295. eCollection 2021. https://doi.org/10.1080/20008198.2021.1932295

211. Hoell A, Kourmpeli E, Salize HJ, Heinz A, Padberg F, Habel U, Kamp-Becker I, Höhne E, Böge K, Bajbouj M. Prevalence of depressive symptoms and symptoms of post-traumatic stress disorder among newly arrived refugees and asylum seekers in Germany: systematic review and meta-analysis. BJPsych Open. 2021 May 3;7(3):e93. https://doi.org/10.1192/bjo.2021.54
212. Cohen F, Hermosilla S, Knox J, Agaba GS, Obalim G, Kajungu R, Mangen PO, Stark L. Protocol for a caregiver psychosocial support intervention for populations affected by displacement in Uganda. BMC Public Health. 2021 May 17;21(1):932. https://doi.org/10.1186/s12889-021-10921-7
213. Shi M, Stey A, Tatebe LC. Recognizing and Breaking the Cycle of Trauma and Violence Among Resettled Refugees. Curr Trauma Rep. 2021;7(4):83-91. Epub 2021 Nov 13. https://doi.org/10.1007/s40719-021-00217-x
214. Der Sarkissian A, Sharkey JD. Transgenerational Trauma and Mental Health Needs among Armenian Genocide Descendants. Int J Environ Res Public Health. 2021 Oct 8;18(19):10554. https://doi.org/10.3390/ijerph181910554
215. Rashki Kemmak A, Nargesi S, Saniee N. Social Determinant of Mental Health in Immigrants and Refugees: A Systematic Review. Med J Islam Repub Iran. 2021 Dec 31;35:196. eCollection 2021. https://doi.org/10.47176/mjiri.35.196
216. Gerber M, Colledge F, de Quervain D, Filippou K, Havas E, Knappe F, Ludyga S, Meier M, Morres ID, Panagos A, Pühse U, Ramadan K, Seelig H, Theodorakis Y, von Känel R, Hatzigeorgiadis A. Effects of an exercise and sport intervention among refugees living in a Greek refugee camp on mental health, physical fitness and cardiovascular risk markers: study protocol for the SALEEM pragmatic randomized controlled trial. Trials. 2021 Nov 21;22(1):827. https://doi.org/10.1186/s13063-021-05808-2
217. Fennig M, Denov M. Interpreters working in mental health settings with refugees: An interdisciplinary scoping review. Am J Orthopsychiatry. 2021;91(1):50-65. Epub 2020 Oct 15. https://doi.org/10.1037/ort0000518
218. Sonne C, Mortensen EL, Silove D, Palic S, Carlsson J. Predictors of treatment outcomes for trauma-affected refugees - results from two randomised trials. J Affect Disord. 2021 Mar 1;282:194-202. Epub 2020 Dec 28. https://doi.org/10.1016/j.jad.2020.12.095
219. de Silva U, Glover N, Katona C. Prevalence of complex post-traumatic stress disorder in refugees and asylum seekers: systematic review. BJPsych Open. 2021 Oct 15;7(6):e194. https://doi.org/10.1192/bjo.2021.1013
220. Akhtar A, Bawaneh A, Awwad M, Al-Hayek H, Sijbrandij M, Cuijpers P, Bryant RA. A longitudinal study of mental health before and during the COVID-19 pandemic in Syrian refugees. Eur J Psychotraumatol. 2021 Nov 10;12(1):1991651. eCollection 2021. https://doi.org/10.1080/20008198.2021.1991651
221. Abbas Z, Eiden C, Salameh P, Peyriere H. Substance use among refugees in three Lebanese camps: A cross-sectional study. Int J Drug Policy. 2021 Aug;94:103204. Epub 2021 Apr 8. https://doi.org/10.1016/j.drugpo.2021.103204
222. Geiling A, Knaevelsrud C, Böttche M, Stammel N. Mental Health and Work Experiences of Interpreters in the Mental Health Care of Refugees: A Systematic Review. Front Psychiatry. 2021 Oct 18;12:710789. eCollection 2021. https://doi.org/10.3389/fpsyt.2021.710789
223. Arundell LL, Barnett P, Buckman JEJ, Saunders R, Pilling S. The effectiveness of adapted psychological interventions for people from ethnic minority groups: A systematic review and conceptual typology. Clin Psychol Rev. 2021 Aug;88:102063. Epub 2021 Jul 7. https://doi.org/10.1016/j.cpr.2021.102063

224. Jing GP, Katz CL. An update on psychotic spectrum disorders and disasters. Curr Opin Psychiatry. 2021 May 1;34(3):211-215. https://doi.org/10.1097/YCO.0000000000000700
225. Beck BD, Meyer SL, Simonsen E, Søgaard U, Petersen I, Arnfred SMH, Tellier T, Moe T. Music therapy was noninferior to verbal standard treatment of traumatized refugees in mental health care: Results from a randomized clinical trial. Eur J Psychotraumatol. 2021 Jul 6;12(1):1930960. eCollection 2021. https://doi.org/10.1080/20008198.2021.1930960
226. Sudheer N, Banerjee D. The Rohingya refugees: a conceptual framework of their psychosocial adversities, cultural idioms of distress and social suffering. Glob Ment Health (Camb). 2021 Dec 17;8:e46. eCollection 2021. https://doi.org/10.1017/gmh.2021.43
227. Bajbouj M, Panneck P, Winter SM, Ajami C, Alabdullah J, Benedikt Burger M, Haberlandner A, Hahn E, Heinz A, Heuser I, Hoyer A, Kluge U, Aichberger M, Repantis D, Schreiter S, Seybold J, Sutej I. A Central Clearing Clinic to Provide Mental Health Services for Refugees in Germany. Front Public Health. 2021 Feb 1;9:635474. eCollection 2021. https://doi.org/10.3389/fpubh.2021.635474
228. Hajak VL, Sardana S, Verdeli H, Grimm S. A Systematic Review of Factors Affecting Mental Health and Well-Being of Asylum Seekers and Refugees in Germany. Front Psychiatry. 2021 Mar 18;12:643704. eCollection 2021. https://doi.org/10.3389/fpsyt.2021.643704
229. Delgado JR, Diaz LD, LaHuffman-Jackson R, Quion N, Walts K. Community-Based Trauma-Informed Care Following Immigrant Family Reunification: A Narrative Review. Acad Pediatr. 2021 May-Jun;21(4):600-604. Epub 2021 Feb 11. https://doi.org/10.1016/j.acap.2021.02.005
230. Lindegaard T, Seaton F, Halaj A, Berg M, Kashoush F, Barchini R, Ludvigsson M, Sarkohi A, Andersson G. Internet-based cognitive behavioural therapy for depression and anxiety among Arabic-speaking individuals in Sweden: a pilot randomized controlled trial. Cogn Behav Ther. 2021 Jan;50(1):47-66. Epub 2020 Jun 30. https://doi.org/10.1080/16506073.2020.1771414
231. Stein J, Niemeyer H, Meyer C, Wirz C, Eiling A, Gruzman R, Heeke C, Stammel N, Knaevelsrud C. Posttraumatic stress in adult civilians exposed to violent conflict, war and associated human rights abuses in the Eastern Mediterranean Region: A systematic review and meta-analysis. J Affect Disord. 2021 Nov 1;294:605-627. Epub 2021 Jun 26. https://doi.org/10.1016/j.jad.2021.06.042
232. Raghuraman S, Stuttard N, Hunt N. Evaluating narrative exposure therapy for post-traumatic stress disorder and depression symptoms: A meta-analysis of the evidence base. Clin Psychol Psychother. 2021 Jan;28(1):1-23. Epub 2020 Jul 15. https://doi.org/10.1002/cpp.2486
233. Semmlinger V, Takano K, Schumm H, Ehring T. Dropout from psychological interventions for refugees and asylum seekers: A meta-analysis. J Consult Clin Psychol. 2021 Sep;89(9):717-730. https://doi.org/10.1037/ccp0000681
234. Sevinc M, Hasbal NB, Sakaci T, Basturk T, Ahbap E, Ortaboz M, Mazi EE, Pirdogan E, Ling J, Unsal A. Frequency of depressive symptoms in Syrian refugees and Turkish maintenance hemodialysis patients during COVID-19 pandemic. PLoS One. 2021 Jan 4;16(1):e0244347. eCollection 2021. https://doi.org/10.1371/journal.pone.0244347
235. Renner A, Jäckle D, Nagl M, Plexnies A, Röhr S, Löbner M, Grochtdreis T, Dams J, König HH, Riedel-Heller S, Kersting A. Traumatized Syrian Refugees with Ambiguous Loss: Predictors of Mental Distress. Int J Environ Res Public Health. 2021 Apr 7;18(8):3865. https://doi.org/10.3390/ijerph18083865

236. Turrini G, Tedeschi F, Cuijpers P, Del Giovane C, Kip A, Morina N, Nosè M, Ostuzzi G, Purgato M, Ricciardi C, Sijbrandij M, Tol W, Barbui C. A network meta-analysis of psychosocial interventions for refugees and asylum seekers with PTSD. BMJ Glob Health. 2021 Jun;6(6):e005029. https://doi.org/10.1136/bmjgh-2021-005029
237. Naeem F, Tuck A, Mutta B, Dhillon P, Thandi G, Kassam A, Farah N, Ashraf A, Husain MI, Husain MO, Vasiliadis HM, Sanches M, Munshi T, Abbott M, Watters N, Kidd SA, Ayub M, McKenzie K. Protocol for a multi-phase, mixed methods study to develop and evaluate culturally adapted CBT to improve community mental health services for Canadians of south Asian origin. Trials. 2021 Sep 6;22(1):600. https://doi.org/10.1186/s13063-021-05547-4
238. Ciaramella M, Monacelli N, Cocimano LCE. Promotion of Resilience in Migrants: A Systematic Review of Study and Psychosocial Intervention. J Immigr Minor Health. 2022 Oct;24(5):1328-1344. Epub 2021 Jul 29. https://doi.org/10.1007/s10903-021-01247-y
239. Magwood O, Kassam A, Mavedatnia D, Mendonca O, Saad A, Hasan H, Madana M, Ranger D, Tan Y, Pottie K. Mental Health Screening Approaches for Resettling Refugees and Asylum Seekers: A Scoping Review. Int J Environ Res Public Health. 2022 Mar 16;19(6):3549. https://doi.org/10.3390/ijerph19063549
240. Magwood O, Bellai-Dussault K, Fox G, McCutcheon C, Adams O, Saad A, Kassam A. Diagnostic test accuracy of screening tools for post-traumatic stress disorder among refugees and asylum seekers: A systematic review and meta-analysis. J Migr Health. 2022 Dec 10;7:100144. eCollection 2023. https://doi.org/10.1016/j.jmh.2022.100144
241. Semmlinger V, Ehring T. Predicting and preventing dropout in research, assessment and treatment with refugees. Clin Psychol Psychother. 2022 May;29(3):767-782. Epub 2021 Oct 11. https://doi.org/10.1002/cpp.2672
242. DeSa S, Gebremeskel AT, Omonaiye O, Yaya S. Barriers and facilitators to access mental health services among refugee women in high-income countries: a systematic review. Syst Rev. 2022 Apr 6;11(1):62. https://doi.org/10.1186/s13643-022-01936-1
243. Troya MI, Spittal MJ, Pendrous R, Crowley G, Gorton HC, Russell K, Byrne S, Musgrove R, Hannah-Swain S, Kapur N, Knipe D. Suicide rates amongst individuals from ethnic minority backgrounds: A systematic review and meta-analysis. EClinicalMedicine. 2022 Apr 28;47:101399. eCollection 2022 May. https://doi.org/10.1016/j.eclinm.2022.101399
244. Aizik-Reebs A, Yuval K, Beyene Kesete Y, Lurie I, Bernstein A. Prevalence and prevention of suicidal ideation among asylum seekers in a high-risk urban post-displacement setting. Epidemiol Psychiatr Sci. 2022 Oct 17;31:e76. https://doi.org/10.1017/S2045796022000579
245. Dowrick C, Rosala-Hallas A, Rawlinson R, Khan N, Winrow E, Chiumento A, Burnside G, Aslam R, Billows L, Eriksson-Lee M, Lawrence D, McCluskey R, Mackinnon A, Moitt T, Orton L, Roberts E, Rahman A, Smith G, Tudor Edwards R, Uwamaliya P, White R. The Problem Management Plus psychosocial intervention for distressed and functionally impaired asylum seekers and refugees: the PROSPER feasibility RCT. Southampton (UK): National Institute for Health and Care Research; 2022 Oct.
246. Bunn M, Zolman N, Smith CP, Khanna D, Hanneke R, Betancourt TS, Weine S. Family-based mental health interventions for refugees across the migration continuum: A systematic review. SSM Ment Health. 2022 Dec;2:100153. Epub 2022 Sep 2. https://doi.org/10.1016/j.ssmmh.2022.100153
247. Williams ACC, Hughes J. [Improving the assessment and treatment of pain in torture survivors : German version]. Schmerz. 2022 Feb;36(1):6-12. Epub 2021 Nov 26. https://doi.org/10.1007/s00482-021-00606-w

248. Jou YC, Pace-Schott EF. Call to action: Addressing sleep disturbances, a hallmark symptom of PTSD, for refugees, asylum seekers, and internally displaced persons. Sleep Health. 2022 Dec;8(6):593-600. Epub 2022 Oct 27. https://doi.org/10.1016/j.sleh.2022.09.003
249. Nissen A, Hynek KA, Scales D, Hilden PK, Straiton M. Chronic pain, mental health and functional impairment in adult refugees from Syria resettled in Norway: a cross-sectional study. BMC Psychiatry. 2022 Aug 24;22(1):571. https://doi.org/10.1186/s12888-022-04200-x
250. Kelstrup L, Carlsson J. Trauma-affected refugees and their non-exposed children: A review of risk and protective factors for trauma transmission. Psychiatry Res. 2022 Jul;313:114604. Epub 2022 May 4. https://doi.org/10.1016/j.psychres.2022.114604
251. Cogo E, Murray M, Villanueva G, Hamel C, Garner P, Senior SL, Henschke N. Suicide rates and suicidal behaviour in displaced people: A systematic review. PLoS One. 2022 Mar 10;17(3):e0263797. eCollection 2022. https://doi.org/10.1371/journal.pone.0263797
252. Nguyen TP, Guajardo MGU, Sahle BW, Renzaho AMN, Slewa-Younan S. Prevalence of common mental disorders in adult Syrian refugees resettled in high income Western countries: a systematic review and meta-analysis. BMC Psychiatry. 2022 Jan 5;22(1):15. https://doi.org/10.1186/s12888-021-03664-7
253. Hermaszewska S, Sweeney A, Camminga B, Botelle R, Elliott K, Sin J. Lived experiences of transgender forced migrants and their mental health outcomes: systematic review and meta-ethnography. BJPsych Open. 2022 May 10;8(3):e91. https://doi.org/10.1192/bjo.2022.51
254. Bruhn M, Laugesen H, Kromann-Larsen M, Trevino CS, Eplov L, Hjorthøj C, Carlsson J. The effect of an integrated care intervention of multidisciplinary mental health treatment and employment services for trauma-affected refugees: study protocol for a randomised controlled trial. Trials. 2022 Oct 8;23(1):859. https://doi.org/10.1186/s13063-022-06774-z
255. Bedaso A, Duko B. Epidemiology of depression among displaced people: A systematic review and meta-analysis. Psychiatry Res. 2022 May;311:114493. Epub 2022 Mar 8. https://doi.org/10.1016/j.psychres.2022.114493
256. Haase E, Schönfelder A, Nesterko Y, Glaesmer H. Prevalence of suicidal ideation and suicide attempts among refugees: a meta-analysis. BMC Public Health. 2022 Apr 1;22(1):635. https://doi.org/10.1186/s12889-022-13029-8
257. Kaggwa MM, Najjuka SM, Bongomin F, Mamun MA, Griffiths MD. Prevalence of depression in Uganda: A systematic review and meta-analysis. PLoS One. 2022 Oct 20;17(10):e0276552. eCollection 2022. https://doi.org/10.1371/journal.pone.0276552
258. Bryant RA, Bawaneh A, Awwad M, Al-Hayek H, Giardinelli L, Whitney C, Jordans MJD, Cuijpers P, Sijbrandij M, Ventevogel P, Dawson K, Akhtar A; STRENGTHS Consortium. Twelve-month follow-up of a randomised clinical trial of a brief group psychological intervention for common mental disorders in Syrian refugees in Jordan. Epidemiol Psychiatr Sci. 2022 Nov 15;31:e81. https://doi.org/10.1017/S2045796022000658
259. Bryant RA, Bawaneh A, Awwad M, Al-Hayek H, Giardinelli L, Whitney C, Jordans MJD, Cuijpers P, Sijbrandij M, Ventevogel P, Dawson K, Akhtar A; STRENGTHS Consortium. Effectiveness of a brief group behavioral intervention for common mental disorders in Syrian refugees in Jordan: A randomized controlled trial. PLoS Med. 2022 Mar 17;19(3):e1003949. eCollection 2022 Mar. https://doi.org/10.1371/journal.pmed.1003949
260. Chowdhury N, Kainth A, Godlu A, Farinas HA, Sikdar S, Turin TC. Mental Health and Well-Being Needs among Non-Health Essential Workers during Recent Epidemics and

Pandemics. Int J Environ Res Public Health. 2022 May 13;19(10):5961. https://doi.org/10.3390/ijerph19105961
261. Bryant RA, Malik A, Aqel IS, Ghatasheh M, Habashneh R, Dawson KS, Watts S, Jordans MJD, Brown FL, van Ommeren M, Akhtar A. Effectiveness of a brief group behavioural intervention on psychological distress in young adolescent Syrian refugees: A randomised controlled trial. PLoS Med. 2022 Aug 12;19(8):e1004046. eCollection 2022 Aug. https://doi.org/10.1371/journal.pmed.1004046
262. Phelps AJ, Lethbridge R, Brennan S, Bryant RA, Burns P, Cooper JA, Forbes D, Gardiner J, Gee G, Jones K, Kenardy J, Kulkarni J, McDermott B, McFarlane AC, Newman L, Varker T, Worth C, Silove D. Australian guidelines for the prevention and treatment of posttraumatic stress disorder: Updates in the third edition. Aust N Z J Psychiatry. 2022 Mar;56(3):230-247. Epub 2021 Aug 27. https://doi.org/10.1177/00048674211041917
263. Solera-Deuchar L. Reflections on mental healthcare for an asylum seeker population caught in limbo on the Greek island of Samos. BJPsych Int. 2023 Feb;20(1):23-25. https://doi.org/10.1192/bji.2022.3
264. Davoren N, McEleney A, Corcoran S, Tierney P, Fortune DG. Refugees and asylum seekers who have experienced trauma: Thematic synthesis of therapeutic boundary considerations. Clin Psychol Psychother. 2023 Sep 2. Online ahead of print. https://doi.org/10.1002/cpp.2894
265. Smyth E, Steel C, Ellett L. The prevalence of non-affective psychosis in refugee populations: A systematic review. Schizophr Res. 2023 Oct;260:99-112. Epub 2023 Aug 25. https://doi.org/10.1016/j.schres.2023.08.011
266. Hynie M, Oda A, Calaresu M, Kuo BCH, Ives N, Jaimes A, Bokore N, Beukeboom C, Ahmad F, Arya N, Samuel R, Farooqui S, Palmer-Dyer JL, McKenzie K. Access to Virtual Mental Healthcare and Support for Refugee and Immigrant Groups: A Scoping Review. J Immigr Minor Health. 2023 Oct;25(5):1171-1195. Epub 2023 Jul 5. https://doi.org/10.1007/s10903-023-01521-1
267. Purgato M, Turrini G, Tedeschi F, Serra R, Tarsitani L, Compri B, Muriago G, Cadorin C, Ostuzzi G, Nicaise P, Lorant V, Sijbrandij M, Witteveen AB, Ayuso-Mateos JL, Mediavilla R, Haro JM, Felez-Nobrega M, Figueiredo N, Pollice G, McDaid D, Park AL, Kalisch R, Petri-Romão P, Underhill J, Bryant RA, Nosè M, Barbui C. Effectiveness of a stepped-care programme of WHO psychological interventions in migrant populations resettled in Italy: Study protocol for the RESPOND randomized controlled trial. Front Public Health. 2023 Jan 25;11:1100546. eCollection 2023. https://doi.org/10.3389/fpubh.2023.1100546
268. Pérez-Vázquez S, Bonilla-Campos A. Women Refugee's Perceptions, Experiences and Coping Mechanisms in Situations of Sexual and Gender-Based Violence (SGBV): A Metasynthesis. Trauma Violence Abuse. 2023 Dec;24(5):3313-3327. Epub 2022 Oct 3. https://doi.org/10.1177/15248380221126470
269. DiClemente-Bosco K, Elizabeth Neville S, Berent JM, Farrar J, Mishra T, Abdi A, Beardslee WR, Creswell JW, Betancourt TS. Understanding mechanisms of change in a family-based preventive mental health intervention for refugees by refugees in New England. Transcult Psychiatry. 2023 Feb;60(1):142-155. Epub 2022 Aug 22. https://doi.org/10.1177/13634615221111627
270. Ali-Naqvi O, Alburak TA, Selvan K, Abdelmeguid H, Malvankar-Mehta MS. Exploring the Impact of Family Separation on Refugee Mental Health: A Systematic Review and Meta-narrative Analysis. Psychiatr Q. 2023 Mar;94(1):61-77. Epub 2023 Jan 24. https://doi.org/10.1007/s11126-022-10013-8

271. Carroll HA, Kvietok A, Pauschardt J, Freier LF, Bird M. Prevalence of common mental health disorders in forcibly displaced populations versus labor migrants by migration phase: A meta-analysis. J Affect Disord. 2023 Jan 15;321:279-289. Epub 2022 Oct 29. https://doi.org/10.1016/j.jad.2022.10.010
272. Bryant RA, Nickerson A, Morina N, Liddell B. Posttraumatic Stress Disorder in Refugees. Annu Rev Clin Psychol. 2023 May 9;19:413-436. Epub 2023 Feb 28. https://doi.org/10.1146/annurev-clinpsy-080921-080359
273. Oren-Schwartz R, Aizik-Reebs A, Yuval K, Hadash Y, Bernstein A. Effect of mindfulness-based trauma recovery for refugees on shame and guilt in trauma recovery among African asylum-seekers. Emotion. 2023 Apr;23(3):622-632. Epub 2022 Aug 4. https://doi.org/10.1037/emo0001126
274. Shaw SA, Lee C, Ahmadi M, Karim Shor Muluk H, Mohamed Jibril Z, Ahmadi L, Randall L, Yang C, Gilbert L. A randomized controlled trial testing the feasibility, acceptability, and preliminary effects of a mental health Screening, Brief Intervention, and Referral to Treatment among refugees in Malaysia. Int J Soc Psychiatry. 2023 Dec;69(8):1898-1908. Epub 2023 Jun 16. https://doi.org/10.1177/00207640231179323
275. Ferguson GM, Causadias JM, Simenec TS. Acculturation and Psychopathology. Annu Rev Clin Psychol. 2023 May 9;19:381-411. Epub 2023 Feb 28. https://doi.org/10.1146/annurev-clinpsy-080921-080622
276. Kim SH, Kim KA, Baek J, Choi J, Chu SH. e-Health for Traumatized Refugees: A Scoping Review. Telemed J E Health. 2023 May;29(5):635-645. Epub 2022 Sep 28. https://doi.org/10.1089/tmj.2022.0228
277. Eskici HS, Hinton DE, Jalal B, Yurtbakan T, Acarturk C. Culturally adapted cognitive behavioral therapy for Syrian refugee women in Turkey: A randomized controlled trial. Psychol Trauma. 2023 Feb;15(2):189-198. Epub 2021 Oct 7. https://doi.org/10.1037/tra0001138
278. Lardier DT, Hess J, Winter L, Goodkind JR. The impact of postresettlement stressors and access to health care on health outcomes in recently resettled refugees in the United States. Am J Orthopsychiatry. 2023;93(6):516-531. Epub 2023 Aug 31. https://doi.org/10.1037/ort0000697
279. Tay AK, Mohsin M, Foo CYS, Rees S, Silove D. Long-term efficacy of brief psychological treatments for common mental disorders in Myanmar refugees in Malaysia: 12-month follow-up of a randomized, active-controlled trial of integrative adapt therapy v. cognitive behavioral therapy. Psychol Med. 2023 Oct;53(13):6055-6067. Epub 2022 Nov 4. https://doi.org/10.1017/S0033291722003245
280. Von Arcosy C, Padilha M, Mello GL, Vilete L, Luz MP, Mendlowicz M, Serpa OD Jr, Berger W. A bright side of adversity? A systematic review on posttraumatic growth among refugees. Stress Health. 2023 Dec;39(5):956-976. Epub 2023 Mar 29. https://doi.org/10.1002/smi.3242
281. Reinhardt I, Schmidt L, Reske D, Zielasek J, Braun G, Böttche M, Boettcher J, Burchert S, Glaesmer H, Knaevelsrud C, Konnopka A, Muntendorf L, Nohr L, Paskuy S, Renneberg B, Sierau S, Stammel N, Wagner B, Wirz T, Gouzoulis-Mayfrank E. Blended-ALMAMAR app for inpatient mental health care for refugees: study protocol for a multicenter implementation study within the I-REACH consortium (Internet based REfugee mentAl healtH Care). BMC Health Serv Res. 2023 Dec 13;23(1):1409. https://doi.org/10.1186/s12913-023-10403-z
282. Mehjabeen D, Blignault I, Taha PH, Reavley N, Slewa-Younan S. A mixed methods systematic review of mental health self-care strategies for Arabic-speaking refugees and

migrants. BMC Public Health. 2023 Dec 20;23(1):2544. https://doi.org/10.1186/s12889-023-17395-9
283. Lüder CC, Michael T, Lass-Hennemann J, Schanz CG, Venhorst A, Meyer T, Equit M. Moderate-intensity aerobic exercise training as an adjunct to trauma-focused psychotherapy in traumatized refugees and asylum seekers: study protocol of a randomized controlled trial. Eur J Psychotraumatol. 2023;14(2):2251777. Epub 2023 Oct 20. https://doi.org/10.1080/20008066.2023.2251777
284. McDaid D, Park AL. Making an economic argument for investment in global mental health: The case of conflict-affected refugees and displaced people. Glob Ment Health (Camb). 2023 Mar 2;10:e10. eCollection 2023. https://doi.org/10.1017/gmh.2023.1
285. Wagner JA, Bermúdez-Millán A, Buckley TE, Buxton OM, Feinn RS, Kong S, Kuoch T, Master L, Scully MF. Secondary analysis of a randomized trial testing community health educator interventions for diabetes prevention among refugees with depression: effects on nutrition, physical activity and sleep. Int J Behav Nutr Phys Act. 2023 Sep 12;20(1):107. https://doi.org/10.1186/s12966-023-01509-y
286. Teixeira-Santos LM, Ventura FIQS, Santos JAO, Almeida IF, Abreu WCP. Mental health training programs for non-health professionals and volunteers working with asylum-seekers and refugees: scoping review. Rev Esc Enferm USP. 2023 Aug 25;57(spe):e20220447. eCollection 2023. https://doi.org/10.1590/1980-220X-REEUSP-2022-0447en
287. Wagner JA, Bermúdez-Millán A, Buckley TE, Buxton OM, Feinn RS, Kong S, Kuoch T, Scully MF. Community-based diabetes prevention randomized controlled trial in refugees with depression: effects on metabolic outcomes and depression. Sci Rep. 2023 May 30;13(1):8718. https://doi.org/10.1038/s41598-023-35738-9
288. Lakin DP, Cooper SE, Andersen L, Brown FL, Augustinavicius JLS, Carswell K, Leku M, Adaku A, Au T, Bryant R, Garcia-Moreno C, White RG, Tol WA. Psychological flexibility in South Sudanese female refugees in Uganda as a mechanism for change within a guided self-help intervention. J Consult Clin Psychol. 2023 Jan;91(1):6-13. https://doi.org/10.1037/ccp0000774
289. Schippert ACSP, Grov EK, Dahl-Michelsen T, Silvola J, Sparboe-Nilsen B, Danielsen SO, Lie I, Bjørnnes AK. Re-traumatization of torture survivors during treatment in somatic healthcare services: A mapping review and appraisal of literature presenting clinical guidelines and recommendations to prevent re-traumatization. Soc Sci Med. 2023 Apr;323:115775. Epub 2023 Feb 25. https://doi.org/10.1016/j.socscimed.2023.115775
290. Milewski A, Weinstein E, Lurie J, Lee A, Taki F, Pilato T, Jedlicka C, Kaur G. Reported Methods, Distributions, and Frequencies of Torture Globally: A Systematic Review and Meta-Analysis. JAMA Netw Open. 2023 Oct 2;6(10):e2336629. https://doi.org/10.1001/jamanetworkopen.2023.36629
291. Kagabo DM, Bangirana P, Burnside G, Chiumento A, Duarte R, Gishoma D, Girvan M, Jansen A, Jansen S, Kasujja R, Lubunga R, Nevitt S, Nzaramba L, Sarabwe E, Jackson C, Rahman A, Richters A, Robinson J, Rutayisire T, Ventevogel P, White RG. Community based sociotherapy for depressive symptomatology of Congolese refugees in Rwanda and Uganda (CoSTAR): a protocol for a cluster randomised controlled trial. Eur J Psychotraumatol. 2023;14(1):2151281. https://doi.org/10.1080/20008066.2022.2151281
292. Miller KE, Chen A, Koppenol-Gonzalez GV, Bakolis I, Arnous M, Tossyeh F, El Hassan A, Saleh A, Saade J, Nahas N, Abboud M, Jawad L, Jordans MJD. Supporting parenting among Syrian refugees in Lebanon: a randomized controlled trial of the caregiver support

intervention. J Child Psychol Psychiatry. 2023 Jan;64(1):71-82. Epub 2022 Jul 15. https://doi.org/10.1111/jcpp.13668
293. Alemi Q, Panter-Brick C, Oriya S, Ahmady M, Alimi AQ, Faiz H, Hakim N, Sami Hashemi SA, Manaly MA, Naseri R, Parwiz K, Sadat SJ, Sharifi MZ, Shinwari Z, Ahmadi SJ, Amin R, Azimi S, Hewad A, Musavi Z, Siddiqi AM, Bragin M, Kashino W, Lavdas M, Miller KE, Missmahl I, Omidian PA, Trani JF, van der Walt SK, Silove D, Ventevogel P. Afghan mental health and psychosocial well-being: thematic review of four decades of research and interventions. BJPsych Open. 2023 Jul 10;9(4):e125. https://doi.org/10.1192/bjo.2023.502
294. Zhao IY, Holroyd E, Garrett N, Wright-St Clair VA, Neville S. Chinese late-life immigrants' loneliness and social isolation in host countries: An integrative review. J Clin Nurs. 2023 May;32(9-10):1615-1624. Epub 2021 Nov 16. https://doi.org/10.1111/jocn.16134
295. Khraisha Q, Abujaber N, Carpenter S, Crossen RJ, Kappenberg J, Kelly R, Murphy C, Norton O, Put SM, Schnoebelen K, Warraitch A, Roney S, Hadfield K. Parenting and mental health in protracted refugee situations: a systematic review. Compr Psychiatry. 2024 Nov;135:152536. Epub 2024 Oct 4. https://doi.org/10.1016/j.comppsych.2024.152536
296. McDermott L, Hameed I, Lau-Zhu A. Cultural Adaptations, Efficacy, and Acceptability of Psychological Interventions for Mental Health in Adults with Refugees and Asylum-Seeker Status: A Systematic Review. Trauma Violence Abuse. 2024 Dec;25(5):3758-3776. Epub 2024 Aug 3. https://doi.org/10.1177/15248380241262262
297. Burgund Isakov A, Markovic V. Systematic Review of Trauma-Informed Approaches and Trauma-Informed Care for Forced Migrant Families: Concepts and Contexts. Trauma Violence Abuse. 2024 Dec;25(5):3999-4015. Epub 2024 Aug 9. https://doi.org/10.1177/15248380241266161
298. Taknint JT, Thomas FC, Gellatly R, Ameresekere M. Responding to Trauma: A Critical Review of Mental Health and Psychosocial Interventions for Refugee Women. Curr Psychiatry Rep. 2024 Dec;26(12):866-876. Epub 2024 Nov 29. https://doi.org/10.1007/s11920-024-01568-3
299. Daniel NA, Liu X, Thomas ET, Eraneva-Dibb E, Ahmad AM, Heneghan C. Brief CBT-based psychological interventions to improve mental health outcomes in refugee populations: a systematic review and meta-analysis. Eur J Psychotraumatol. 2024;15(1):2389702. Epub 2024 Aug 30. https://doi.org/10.1080/20008066.2024.2389702
300. Gasnier M, Aouizerat A, Chappell K, Baubet T, Corruble E. Psychotic and Somatic Symptoms Are Frequent in Refugees With Posttraumatic Stress Disorder: A Narrative Review. J Psychiatr Pract. 2024 Mar 1;30(2):104-118. https://doi.org/10.1097/PRA.0000000000000772
301. Santambrogio J, Cimminiello N, Wisidagamage Don P, Leon E, Miragliotta E, Capuzzi E, Colmegna F, Clerici M. Influence of post-migration living difficulties on mental health among refugees and asylum seekers: A scoping-review on clinical tools. Int J Soc Psychiatry. 2024 Nov;70(7):1191-1201. Epub 2024 Jul 24. https://doi.org/10.1177/00207640241251748
302. Sultani G, Heinsch M, Wilson J, Pallas P, Tickner C, Kay-Lambkin F. 'Now I Have Dreams in Place of the Nightmares': An Updated Systematic Review of Post-Traumatic Growth Among Refugee Populations. Trauma Violence Abuse. 2024 Jan;25(1):795-812. Epub 2023 Apr 25. https://doi.org/10.1177/15248380231163641
303. Abu-Ras W, Almoayad F, Bakry HM, Alammari D, Kelly PJ, Aboul-Enein BH. Interventions to promote mental health in the Occupied Palestinian Territories and

Palestinian refugees: A scoping review. Int J Soc Psychiatry. 2024 Sep;70(6):1037-1054. Epub 2024 Jun 18. https://doi.org/10.1177/00207640241259995
304. Giosan C, Popoviciu CM, Zhamaliyeva S, Zaborot I, Deac G. Evaluating the efficacy of support groups in the metaverse for Ukrainian refugees: a protocol for a randomized clinical trial. Trials. 2024 Oct 19;25(1):697. https://doi.org/10.1186/s13063-024-08543-6
305. Kirsch J, Kitchens K, Kerr K, Sivakumaran S. Group-Based Intervention Models in Treating Refugee Mental Health in High-Income Countries: A Systematic Review. Trauma Violence Abuse. 2024 Dec;25(5):4173-4187. Epub 2024 Aug 15. https://doi.org/10.1177/15248380241270039
306. Anisman H, Doubad D, Asokumar A, Matheson K. Psychosocial and neurobiological aspects of the worldwide refugee crisis: From vulnerability to resilience. Neurosci Biobehav Rev. 2024 Oct;165:105859. Epub 2024 Aug 17. https://doi.org/10.1016/j.neubiorev.2024.105859
307. Ramadan M, Hadfield K, Ryan M, Cai P, Bosqui T, Nolan A. The use of creative art therapy to address the mental health of refugee adolescents: a systematic review. Arts Health. 2024 Sep 18:1-19. Online ahead of print. https://doi.org/10.1080/17533015.2024.2395896
308. Nguyen TP, Al Asaad M, Sena M, Slewa-Younan S. Loneliness and social isolation amongst refugees resettled in high-income countries: A systematic review. Soc Sci Med. 2024 Nov;360:117340. Epub 2024 Sep 13. https://doi.org/10.1016/j.socscimed.2024.117340
309. Nohr L, Dumke L, Klein EM, Wilker S. [Current Outpatient Psychotherapeutic Care for People with Migration and Refugee Experience in Germany - An Overview]. Psychother Psychosom Med Psychol. 2024 Jun;74(6):205-213. Epub 2024 Jun 12. https://doi.org/10.1055/a-2304-8902
310. Amodu O, Janes CR, Pangan KTL. Psychosocial well-being and mental health of low- and middle-income countries' internally displaced persons and refugees during COVID-19: a systematic literature review. Glob Ment Health (Camb). 2024 Dec 10;11:e122. eCollection 2024. https://doi.org/10.1017/gmh.2024.110
311. Zoellner LA, Bentley JA, Musa K, Mohamed F, Ahmed LB, King KM, Feeny NC; Islamic Trauma Healing Clinical Team. Lay-Led Intervention for War and Refugee Trauma: A Randomized Clinical Trial. JAMA Netw Open. 2024 Aug 1;7(8):e2429661. https://doi.org/10.1001/jamanetworkopen.2024.29661
312. Trabsa A, Casanovas F, Pérez V, Moreno A, Amann B, Mané A. Comparison of male and female non-refugee immigrants with psychosis: clinical, sociodemographic, and migration-related differences and impact on stress. Arch Womens Ment Health. 2024 Oct;27(5):679-692. Epub 2024 Feb 19. https://doi.org/10.1007/s00737-024-01431-7
313. Côté-Olijnyk M, Perry JC, Paré MÈ, Kronick R. The mental health of migrants living in limbo: A mixed-methods systematic review with meta-analysis. Psychiatry Res. 2024 Jul;337:115931. Epub 2024 May 4. https://doi.org/10.1016/j.psychres.2024.115931
314. Karvela P, Papathanasiou C. [Posttraumatic stress disorder in refugees and therapeutic interventions based on cognitive behavioral approach: A systematic review]. Psychiatriki. 2024 Sep 18. Online ahead of print. https://doi.org/10.22365/jpsych.2024.011
315. Zagha K, Konietzny K, Brettschneider C, Chehadi O, Chehadi-Köster A, Chikhradze N, Dababneh N, Hegerath FM, Heller L, Dehnen A, Hessbruegge M, In der Schmitten J, König HH, Krasko J, Kumsta R, Luhmann M, Lukaschek K, Margraf J, Pflug V, Roesgen D, Sönnichsen A, Vollmar HC, Gensichen J, Schneider S. Improve Mental Health (Improve-MH) in refugee families using a culturally adapted, general practitioner-delivered psychotherapeutic intervention combined with Triple P Online parenting programme: study protocol of a

multicentre randomised controlled trial. BMJ Open. 2024 Sep 24;14(9):e084080. https://doi.org/10.1136/bmjopen-2024-084080
316. Dowllah IM, Melville C. Effectiveness of psychosocial interventions for post-traumatic stress disorder in refugees and asylum seekers resettled in low- and middle-income countries: A systematic review and meta-analysis. J Health Psychol. 2024 Nov;29(13):1463-1474. Epub 2023 Sep 20. https://doi.org/10.1177/13591053231199254
317. Asanov AM, Asanov I, Buenstorf G. A low-cost digital first aid tool to reduce psychological distress in refugees: A multi-country randomized controlled trial of self-help online in the first months after the invasion of Ukraine. Soc Sci Med. 2024 Dec;362:117442. Epub 2024 Oct 24. https://doi.org/10.1016/j.socscimed.2024.117442
318. Dumke L, Wilker S, Hecker T, Neuner F. Barriers to accessing mental health care for refugees and asylum seekers in high-income countries: A scoping review of reviews mapping demand and supply-side factors onto a conceptual framework. Clin Psychol Rev. 2024 Nov;113:102491. Epub 2024 Aug 22. https://doi.org/10.1016/j.cpr.2024.102491
319. Filges T, Bengtsen E, Montgomery E, Kildemoes MW. The impact of detention on the health of asylum seekers: An updated systematic review: A systematic review. Campbell Syst Rev. 2024 Jul 8;20(3):e1420. eCollection 2024 Sep. https://doi.org/10.1002/cl2.1420
320. Mabil-Atem JM, Gumuskaya O, Wilson RL. Digital mental health interventions for the mental health care of refugees and asylum seekers: Integrative literature review. Int J Ment Health Nurs. 2024 Aug;33(4):760-780. Epub 2024 Jan 30. https://doi.org/10.1111/inm.13283
321. Yılmaz T, Karakuş C. Psychotherapeutic Interventions Used in Psychological Treatment Studies With Syrian Refugees: A Systematic Review. Trauma Violence Abuse. 2024 Dec 24:15248380241306029. Online ahead of print. https://doi.org/10.1177/15248380241306029
322. Tucker S, Baldonado N, Ruina O, Ratmann O, Flaxman S, Bryn L, Lachman J, Taradaika E, Melendez-Torres GJ, Vallance I, Goldman P, Cluver L, Hillis S. Hope Groups: a protocol for a cluster randomized controlled trial of psychosocial, mental health, and parenting support groups for Ukrainian caregivers during war and conflict. Trials. 2024 Jul 17;25(1):486. https://doi.org/10.1186/s13063-024-08233-3
323. Gkintoni E, Nikolaou G. The Cross-Cultural Validation of Neuropsychological Assessments and Their Clinical Applications in Cognitive Behavioral Therapy: A Scoping Analysis. Int J Environ Res Public Health. 2024 Aug 22;21(8):1110. https://doi.org/10.3390/ijerph21081110
324. Miller-Graff LE, Paulson J, Hosny N, Ellis K. Trauma, resilience, and distress in post-revolution Egypt. Psychol Trauma. 2024 Jan;16(1):49-56. Epub 2022 Jun 9. https://doi.org/10.1037/tra0001239
325. Blackwell MA, Goodkind JR, Yeater EA, Van Horn ML. Predictors of mental health outcomes of three refugee groups in an advocacy-based intervention: A precision medicine perspective. J Consult Clin Psychol. 2024 Jan;92(1):16-25. Epub 2023 Sep 28. https://doi.org/10.1037/ccp0000847
326. Tutlam NT, Chang JJ, Byansi W, Flick LH, Ssewamala FM, Betancourt TS. War-Affected South Sudanese in Settings of Preflight, Flight, and Resettlement: a Systematic Review and Meta-analysis of Trauma-Associated Mental Disorders. Glob Soc Welf. 2024 Sep;11(3):193-210. Epub 2022 Jul 18. https://doi.org/10.1007/s40609-022-00227-w
327. Ojha S, Thapa S, Thapa SB. Mental health problems among Syrian refugees in Nordic countries: a systematic review. Nord J Psychiatry. 2024 Oct;78(7):561-569. Epub 2024 Sep 16. https://doi.org/10.1080/08039488.2024.2403600

328. Liddell BJ, Das P, Malhi GS, Jobson L, Lau W, Felmingham KL, Nickerson A, Askovic M, Aroche J, Coello M, Bryant RA. Self-construal modulates default mode network connectivity in refugees with PTSD. J Affect Disord. 2024 Sep 15;361:268-276. Epub 2024 Jun 10. https://doi.org/10.1016/j.jad.2024.06.009
329. Papola D, Prina E, Ceccarelli C, Cadorin C, Gastaldon C, Ferreira MC, Tol WA, van Ommeren M, Barbui C, Purgato M. Psychological and social interventions for the promotion of mental health in people living in low- and middle-income countries affected by humanitarian crises. Cochrane Database Syst Rev. 2024 May 21;5(5):CD014300. https://doi.org/10.1002/14651858.CD014300.pub2
330. Osman W, Ncube F, Shaaban K, Dafallah A. Prevalence, predictors, and economic burden of mental health disorders among asylum seekers, refugees and migrants from African countries: A scoping review. PLoS One. 2024 Jun 24;19(6):e0305495. eCollection 2024. https://doi.org/10.1371/journal.pone.0305495
331. Miller-Suchet L, Camargo N, Sangraula M, Castellar D, Diaz J, Meriño V, Chamorro Coneo AM, Chávez D, Venegas M, Cristobal M, Bonz AG, Ramirez C, Trejos Herrera AM, Ventevogel P, Brown AD, Schojan M, Greene MC. Comparing Mediators and Moderators of Mental Health Outcomes from the Implementation of Group Problem Management Plus (PM+) among Venezuelan Refugees and Migrants and Colombian Returnees in Northern Colombia. Int J Environ Res Public Health. 2024 Apr 24;21(5):527. https://doi.org/10.3390/ijerph21050527
332. Burchert S, Alkneme MS, Alsaod A, Cuijpers P, Heim E, Hessling J, Hosny N, Sijbrandij M, Van't Hof E, Ventevogel P, Knaevelsrud C; STRENGTHS Consortium. Effects of a self-guided digital mental health self-help intervention for Syrian refugees in Egypt: A pragmatic randomized controlled trial. PLoS Med. 2024 Sep 9;21(9):e1004460. eCollection 2024 Sep. https://doi.org/10.1371/journal.pmed.1004460
333. Kemna S, Bringmann M, Karnouk C, Hoell A, Tschorn M, Kamp-Becker I, Padberg F, Übleis A, Hasan A, Falkai P, Salize HJ, Meyer-Lindenberg A, Banaschewski T, Schneider F, Habel U, Plener P, Hahn E, Wiechers M, Strupf M, Jobst A, Millenet S, Hoehne E, Sukale T, Schuster M, Dinauer R, Mehran N, Kaiser F, Lieb K, Heinz A, Rapp M, Bajbouj M, Böge K. Predictors of symptom change in the mental health of refugees and asylum seekers (MEHIRA) study examining the effects of a stepped and collaborative care model - A multicentered rater-blinded randomized controlled trial. J Affect Disord. 2025 Feb 1;370:45-53. Epub 2024 Oct 28. https://doi.org/10.1016/j.jad.2024.10.103
334. Ashfaq A, Esmaili S, Najjar M, Batool F, Mukatash T, Al-Ani HA, Koga PM. Utilization of Mobile Mental Health Services among Syrian Refugees and Other Vulnerable Arab Populations-A Systematic Review. Int J Environ Res Public Health. 2020 Feb 18;17(4):1295. https://doi.org/10.3390/ijerph17041295
335. Ermansons G, Kienzler H, Asif Z, Schofield P. Refugee mental health and the role of place in the Global North countries: A scoping review. Health Place. 2023 Jan;79:102964. Epub 2023 Jan 8. https://doi.org/10.1016/j.healthplace.2023.102964
336. Sangalang CC, Vang C. Intergenerational Trauma in Refugee Families: A Systematic Review. J Immigr Minor Health. 2017 Jun;19(3):745-754. https://doi.org/10.1007/s10903-016-0499-7
337. Hynie M. The Social Determinants of Refugee Mental Health in the Post-Migration Context: A Critical Review. Can J Psychiatry. 2018 May;63(5):297-303. Epub 2017 Dec 4. https://doi.org/10.1177/0706743717746666
338. Due C, Green E, Ziersch A. Psychological trauma and access to primary healthcare for people from refugee and asylum-seeker backgrounds: a mixed methods systematic review.

Int J Ment Health Syst. 2020 Sep 11;14:71. eCollection 2020. https://doi.org/10.1186/s13033-020-00404-4
339. Griswold KS, Loomis DM, Pastore PA. Mental Health and Illness. Prim Care. 2021 Mar;48(1):131-145. Epub 2020 Dec 19. https://doi.org/10.1016/j.pop.2020.09.009
340. Mellor R, Werner A, Moussa B, Mohsin M, Jayasuriya R, Tay AK. Prevalence, predictors and associations of complex post-traumatic stress disorder with common mental disorders in refugees and forcibly displaced populations: a systematic review. Eur J Psychotraumatol. 2021 Feb 2;12(1):1863579. eCollection 2021. https://doi.org/10.1080/20008198.2020.1863579
341. Pluck F, Ettema R, Vermetten E. Threats and Interventions on Wellbeing in Asylum Seekers in the Netherlands: A Scoping Review. Front Psychiatry. 2022 Apr 1;13:829522. eCollection 2022. https://doi.org/10.3389/fpsyt.2022.829522
342. Mattar S, Gellatly R. Refugee mental health: Culturally relevant considerations. Curr Opin Psychol. 2022 Oct;47:101429. Epub 2022 Jul 20. https://doi.org/10.1016/j.copsyc.2022.101429
343. Gewirtz AH, Muldrew L, Sigmarsdóttir M. Mental health, risk and resilience among refugee families in Europe. Curr Opin Psychol. 2022 Oct;47:101428. Epub 2022 Jul 16. https://doi.org/10.1016/j.copsyc.2022.101428
344. Javanbakht A, Grasser LR. Biological Psychiatry in Displaced Populations: What We Know, and What We Need to Begin to Learn. Biol Psychiatry Cogn Neurosci Neuroimaging. 2022 Dec;7(12):1242-1250. Epub 2022 May 14. https://doi.org/10.1016/j.bpsc.2022.05.001
345. Grasser LR. Addressing Mental Health Concerns in Refugees and Displaced Populations: Is Enough Being Done?. Risk Manag Healthc Policy. 2022 May 6;15:909-922. eCollection 2022. https://doi.org/10.2147/RMHP.S270233
346. Schäfer SK, Kunzler AM, Lindner S, Broll J, Stoll M, Stoffers-Winterling J, Lieb K. Transdiagnostic psychosocial interventions to promote mental health in forcibly displaced persons: a systematic review and meta-analysis. Eur J Psychotraumatol. 2023;14(2):2196762. https://doi.org/10.1080/20008066.2023.2196762
347. Sukiasyan S. The Mental Health of Refugees and Forcibly Displaced People: A Narrative Review. Consort Psychiatr. 2024 Dec 19;5(4):78-92. eCollection 2024. https://doi.org/10.17816/CP15552
348. Schouler-Ocak M, Moran JK. Anxiety and mood disorders in forcibly displaced people across the world. Curr Opin Psychiatry. 2024 Jan 1;37(1):18-22. Epub 2023 Oct 27. https://doi.org/10.1097/YCO.0000000000000904
349. Sisenop F, Chatarajupalli P, Bain PA, Kaade H, Lindert J. Human rights violations are associated with forcibly displaced population's mental health-a systematic review and meta-analysis. Front Public Health. 2025 Jan 16;12:1454331. eCollection 2024. https://doi.org/10.3389/fpubh.2024.1454331
350. Fazel M, Reed RV, Panter-Brick C, Stein A. Mental health of displaced and refugee children resettled in high-income countries: risk and protective factors. Lancet. 2012 Jan 21;379(9812):266-82. Epub 2011 Aug 9. https://doi.org/10.1016/S0140-6736(11)60051-2
351. Tam SY, Houlihan S, Melendez-Torres GJ. A Systematic Review of Longitudinal Risk and Protective Factors and Correlates for Posttraumatic Stress and Its Natural History in Forcibly Displaced Children. Trauma Violence Abuse. 2017 Oct;18(4):377-395. Epub 2015 Dec 30. https://doi.org/10.1177/1524838015622437
352. Fabio M, Parker LD, Siddharth MB. Building on Resiliencies of Refugee Families. Pediatr Clin North Am. 2019 Jun;66(3):655-667. https://doi.org/10.1016/j.pcl.2019.02.011

353. Cayabyab CR, O'Reilly P, Murphy AM, O'Gorman C. Psychological morbidity among forcibly displaced children-a literature review. Ir J Med Sci. 2020 Aug;189(3):991-997. Epub 2020 Jan 28. https://doi.org/10.1007/s11845-020-02186-7
354. Schouler-Ocak M, Graef-Calliess IT, Bajbouj M, Plener PL. [Mental Disorders among Refugees]. Z Kinder Jugendpsychiatr Psychother. 2020 Nov;48(6):453-457. https://doi.org/10.1024/1422-4917/a000769
355. Khan F, Eskander N, Limbana T, Salman Z, Siddiqui PA, Hussaini S. Refugee and Migrant Children's Mental Healthcare: Serving the Voiceless, Invisible, and the Vulnerable Global Citizens. Cureus. 2020 Aug 22;12(8):e9944. https://doi.org/10.7759/cureus.9944
356. Bartlett R, Sarnyai Z, Momartin S, Ooi L, Schwab SG, Matosin N. Understanding the pathology of psychiatric disorders in refugees. Psychiatry Res. 2021 Feb;296:113661. Epub 2020 Dec 24. https://doi.org/10.1016/j.psychres.2020.113661
357. Dangmann C, Dybdahl R, Solberg Ø. Mental health in refugee children. Curr Opin Psychol. 2022 Dec;48:101460. Epub 2022 Aug 24. https://doi.org/10.1016/j.copsyc.2022.101460
358. Verhagen IL, Noom MJ, Lindauer RJL, Daams JG, Hein IM. Mental health screening and assessment tools for forcibly displaced children: a systematic review. Eur J Psychotraumatol. 2022 Sep 29;13(2):2126468. eCollection 2022. https://doi.org/10.1080/20008066.2022.2126468
359. Due C, Currie E. Practitioner competencies for working with refugee children and young people: A scoping review. Transcult Psychiatry. 2022 Apr;59(2):116-129. Epub 2021 Dec 3. https://doi.org/10.1177/13634615211043765
360. Taylor A, Radford G, Calia C. Review: Cultural adaptations to psychosocial interventions for families with refugee/asylum-seeker status in the United Kingdom - a systematic review. Child Adolesc Ment Health. 2023 May;28(2):241-257. Epub 2022 Feb 23. https://doi.org/10.1111/camh.12547
361. Thabet A, Ghandi S, Barker EK, Rutherford G, Malekinejad M. Interventions to enhance psychological resilience in forcibly displaced children: a systematic review. BMJ Glob Health. 2023 Feb;8(2):e007320. https://doi.org/10.1136/bmjgh-2021-007320
362. Cowling MM, Anderson JR. The effectiveness of therapeutic interventions on psychological distress in refugee children: A systematic review. J Clin Psychol. 2023 Aug;79(8):1857-1874. Epub 2023 Jan 12. https://doi.org/10.1002/jclp.23479
363. Oleimat AS, Jones C, Hayter M. Middle eastern refugee children and adolescents mental health: A systematic review. Int J Ment Health Nurs. 2023 Jun;32(3):687-703. Epub 2022 Nov 7. https://doi.org/10.1111/inm.13088
364. Di Nicola V, Leslie M, Haynes C, Nesbeth K. Clinical Considerations for Immigrant, Refugee, and Asylee Youth Populations. Child Adolesc Psychiatr Clin N Am. 2022 Oct;31(4):679-692. https://doi.org/10.1016/j.chc.2022.06.010
365. Schmidt KL, Rasmussen AL, Thomsen PH. [Refugee and immigrant children referred to a department of pediatric psychiatry during the years 1991-1994]. Ugeskr Laeger. 2000 Apr 17;162(16):2318-23.
366. Francisković T, Moro L, Torić I, Urlić I, Roncević-Grzeta I, Tić-Bacić T. The impact of traumatic experience on attitude towards future in refugee adolescents. Coll Antropol. 2000 Dec;24(2):579-84.
367. Loughry M, Flouri E. The behavioral and emotional problems of former unaccompanied refugee children 3-4 years after their return to Vietnam. Child Abuse Negl. 2001 Feb;25(2):249-63. https://doi.org/10.1016/s0145-2134(00)00240-4

368. Sundelin-Wahlsten V, Ahmad A, von Knorring AL. Traumatic experiences and post-traumatic stress reactions in children from Kurdistan and Sweden. Acta Paediatr. 2001 May;90(5):563-8.
369. Sundelin Wahlsten V, Ahmad A, von Knorring AL. Traumatic experiences and posttraumatic stress reactions in children and their parents from Kurdistan and Sweden. Nord J Psychiatry. 2001;55(6):395-400. https://doi.org/10.1080/08039480152693282
370. Fazel M, Stein A. Mental health of refugee children: comparative study. BMJ. 2003 Jul 19;327(7407):134. https://doi.org/10.1136/bmj.327.7407.134
371. Bolea PS, Grant G Jr, Burgess M, Plasa O. Trauma of children of the Sudan: a constructivist exploration. Child Welfare. 2003 Mar-Apr;82(2):219-33.
372. Goodman JH. Coping with trauma and hardship among unaccompanied refugee youths from Sudan. Qual Health Res. 2004 Nov;14(9):1177-96. https://doi.org/10.1177/1049732304265923
373. Lustig SL, Kia-Keating M, Knight WG, Geltman P, Ellis H, Kinzie JD, Keane T, Saxe GN. Review of child and adolescent refugee mental health. J Am Acad Child Adolesc Psychiatry. 2004 Jan;43(1):24-36. https://doi.org/10.1097/00004583-200401000-00012
374. Halcón LL, Robertson CL, Savik K, Johnson DR, Spring MA, Butcher JN, Westermeyer JJ, Jaranson JM. Trauma and coping in Somali and Oromo refugee youth. J Adolesc Health. 2004 Jul;35(1):17-25. https://doi.org/10.1016/j.jadohealth.2003.08.005
375. Basham K. Transforming the legacies of childhood trauma in couple and family therapy. Soc Work Health Care. 2004;39(3-4):263-85. https://doi.org/10.1300/j010v39n03_04
376. Nadeau L, Measham T. Caring for migrant and refugee children: challenges associated with mental health care in pediatrics. J Dev Behav Pediatr. 2006 Apr;27(2):145-54. https://doi.org/10.1097/00004703-200604000-00013
377. Kinzie JD, Cheng K, Tsai J, Riley C. Traumatized refugee children: the case for individualized diagnosis and treatment. J Nerv Ment Dis. 2006 Jul;194(7):534-7. https://doi.org/10.1097/01.nmd.0000224946.93376.51
378. Ehntholt KA, Yule W. Practitioner review: assessment and treatment of refugee children and adolescents who have experienced war-related trauma. J Child Psychol Psychiatry. 2006 Dec;47(12):1197-210. https://doi.org/10.1111/j.1469-7610.2006.01638.x
379. Bean T, Derluyn I, Eurelings-Bontekoe E, Broekaert E, Spinhoven P. Comparing psychological distress, traumatic stress reactions, and experiences of unaccompanied refugee minors with experiences of adolescents accompanied by parents. J Nerv Ment Dis. 2007 Apr;195(4):288-97. https://doi.org/10.1097/01.nmd.0000243751.49499.93
380. Murray LK, Cohen JA, Ellis BH, Mannarino A. Cognitive behavioral therapy for symptoms of trauma and traumatic grief in refugee youth. Child Adolesc Psychiatr Clin N Am. 2008 Jul;17(3):585-604, ix. https://doi.org/10.1016/j.chc.2008.02.003
381. Weine S. Family roles in refugee youth resettlement from a prevention perspective. Child Adolesc Psychiatr Clin N Am. 2008 Jul;17(3):515-32, vii-viii. https://doi.org/10.1016/j.chc.2008.02.006
382. Crowley C. The mental health needs of refugee children: a review of literature and implications for nurse practitioners. J Am Acad Nurse Pract. 2009 Jun;21(6):322-31. https://doi.org/10.1111/j.1745-7599.2009.00413.x
383. Derluyn I, Mels C, Broekaert E. Mental health problems in separated refugee adolescents. J Adolesc Health. 2009 Mar;44(3):291-7. Epub 2008 Nov 12. https://doi.org/10.1016/j.jadohealth.2008.07.016

384. Vostroknutov NV. [New approaches to the diagnosis of mental state of child victims of emergency situations]. Zh Nevrol Psikhiatr Im S S Korsakova. 2009;109(12):18-23.
385. Quiroga J. Torture in children. Torture. 2009;19(2):66-87.
386. Peltonen K, Punamäki RL. Preventive interventions among children exposed to trauma of armed conflict: a literature review. Aggress Behav. 2010 Mar-Apr;36(2):95-116. https://doi.org/10.1002/ab.20334
387. Bronstein I, Montgomery P. Psychological distress in refugee children: a systematic review. Clin Child Fam Psychol Rev. 2011 Mar;14(1):44-56. https://doi.org/10.1007/s10567-010-0081-0
388. Reed RV, Fazel M, Jones L, Panter-Brick C, Stein A. Mental health of displaced and refugee children resettled in low-income and middle-income countries: risk and protective factors. Lancet. 2012 Jan 21;379(9812):250-65. Epub 2011 Aug 9. https://doi.org/10.1016/S0140-6736(11)60050-0
389. Werner EE. Children and war: risk, resilience, and recovery. Dev Psychopathol. 2012 May;24(2):553-8. https://doi.org/10.1017/S0954579412000156
390. Dhossche DM, Ross CA, Stoppelbein L. The role of deprivation, abuse, and trauma in pediatric catatonia without a clear medical cause. Acta Psychiatr Scand. 2012 Jan;125(1):25-32. Epub 2011 Oct 24. https://doi.org/10.1111/j.1600-0447.2011.01779.x
391. Guruge S, Butt H. A scoping review of mental health issues and concerns among immigrant and refugee youth in Canada: Looking back, moving forward. Can J Public Health. 2015 Feb 3;106(2):e72-8. https://doi.org/10.17269/cjph.106.4588
392. Unterhitzenberger J, Eberle-Sejari R, Rassenhofer M, Sukale T, Rosner R, Goldbeck L. Trauma-focused cognitive behavioral therapy with unaccompanied refugee minors: a case series. BMC Psychiatry. 2015 Oct 23;15:260. https://doi.org/10.1186/s12888-015-0645-0
393. Pottie K, Dahal G, Georgiades K, Premji K, Hassan G. Do First Generation Immigrant Adolescents Face Higher Rates of Bullying, Violence and Suicidal Behaviours Than Do Third Generation and Native Born?. J Immigr Minor Health. 2015 Oct;17(5):1557-66. https://doi.org/10.1007/s10903-014-0108-6
394. Rassenhofer M, Fegert JM, Plener PL, Witt A. [Validated Instruments for the Psychological Assessment of Unaccompanied Refugee Minors - a Systematic Review]. Prax Kinderpsychol Kinderpsychiatr. 2016;65(2):97-112. https://doi.org/10.13109/prkk.2016.65.2.97
395. Reavell J, Fazil Q. The epidemiology of PTSD and depression in refugee minors who have resettled in developed countries. J Ment Health. 2017 Feb;26(1):74-83. Epub 2016 Sep 29. https://doi.org/10.1080/09638237.2016.1222065
396. Gadeberg AK, Montgomery E, Frederiksen HW, Norredam M. Assessing trauma and mental health in refugee children and youth: a systematic review of validated screening and measurement tools. Eur J Public Health. 2017 Jun 1;27(3):439-446. https://doi.org/10.1093/eurpub/ckx034
397. Betancourt TS, Newnham EA, Birman D, Lee R, Ellis BH, Layne CM. Comparing Trauma Exposure, Mental Health Needs, and Service Utilization Across Clinical Samples of Refugee, Immigrant, and U.S.-Origin Children. J Trauma Stress. 2017 Jun;30(3):209-218. Epub 2017 Jun 6. https://doi.org/10.1002/jts.22186
398. Fazel M, Betancourt TS. Preventive mental health interventions for refugee children and adolescents in high-income settings. Lancet Child Adolesc Health. 2018 Feb;2(2):121-132. Epub 2017 Nov 21. https://doi.org/10.1016/S2352-4642(17)30147-5

399. Vossoughi N, Jackson Y, Gusler S, Stone K. Mental Health Outcomes for Youth Living in Refugee Camps: A Review. Trauma Violence Abuse. 2018 Dec;19(5):528-542. Epub 2016 Oct 11. https://doi.org/10.1177/1524838016673602
400. Demazure G, Gaultier S, Pinsault N. Dealing with difference: a scoping review of psychotherapeutic interventions with unaccompanied refugee minors. Eur Child Adolesc Psychiatry. 2018 Apr;27(4):447-466. Epub 2017 Dec 6. https://doi.org/10.1007/s00787-017-1083-y
401. Eruyar S, Huemer J, Vostanis P. Review: How should child mental health services respond to the refugee crisis?. Child Adolesc Ment Health. 2018 Nov;23(4):303-312. Epub 2017 Nov 7. https://doi.org/10.1111/camh.12252
402. Mohwinkel LM, Nowak AC, Kasper A, Razum O. Gender differences in the mental health of unaccompanied refugee minors in Europe: a systematic review. BMJ Open. 2018 Jul 30;8(7):e022389. https://doi.org/10.1136/bmjopen-2018-022389
403. Murray JS. Toxic stress and child refugees. J Spec Pediatr Nurs. 2018 Jan;23(1). Epub 2017 Nov 20. https://doi.org/10.1111/jspn.12200
404. Myles P, Swenshon S, Haase K, Szeles T, Jung C, Jacobi F, Rath B. A comparative analysis of psychological trauma experienced by children and young adults in two scenarios: evacuation after a natural disaster vs forced migration to escape armed conflict. Public Health. 2018 May;158:163-175. Epub 2018 Apr 5. https://doi.org/10.1016/j.puhe.2018.03.012
405. Pfeiffer E, Sachser C, Rohlmann F, Goldbeck L. Effectiveness of a trauma-focused group intervention for young refugees: a randomized controlled trial. J Child Psychol Psychiatry. 2018 Nov;59(11):1171-1179. Epub 2018 Apr 6. https://doi.org/10.1111/jcpp.12908
406. Ismayilova L, Karimli L, Sanson J, Gaveras E, Nanema R, Tô-Camier A, Chaffin J. Improving mental health among ultra-poor children: Two-year outcomes of a cluster-randomized trial in Burkina Faso. Soc Sci Med. 2018 Jul;208:180-189. Epub 2018 May 7. https://doi.org/10.1016/j.socscimed.2018.04.022
407. Purgato M, Gross AL, Betancourt T, Bolton P, Bonetto C, Gastaldon C, Gordon J, O'Callaghan P, Papola D, Peltonen K, Punamaki RL, Richards J, Staples JK, Unterhitzenberger J, van Ommeren M, de Jong J, Jordans MJD, Tol WA, Barbui C. Focused psychosocial interventions for children in low-resource humanitarian settings: a systematic review and individual participant data meta-analysis. Lancet Glob Health. 2018 Apr;6(4):e390-e400. https://doi.org/10.1016/S2214-109X(18)30046-9
408. Hodes M. New developments in the mental health of refugee children and adolescents. Evid Based Ment Health. 2019 May;22(2):72-76. Epub 2019 Apr 3. https://doi.org/10.1136/ebmental-2018-300065
409. Kameg BN. Management of mental health conditions in refugee youth: An overview for the psychiatric-mental health nurse practitioner. J Child Adolesc Psychiatr Nurs. 2019 Nov;32(4):179-186. Epub 2019 Sep 15. https://doi.org/10.1111/jcap.12253
410. Marley C, Mauki B. Resilience and protective factors among refugee children post-migration to high-income countries: a systematic review. Eur J Public Health. 2019 Aug 1;29(4):706-713. https://doi.org/10.1093/eurpub/cky232
411. Kien C, Sommer I, Faustmann A, Gibson L, Schneider M, Krczal E, Jank R, Klerings I, Szelag M, Kerschner B, Brattström P, Gartlehner G. Prevalence of mental disorders in young refugees and asylum seekers in European Countries: a systematic review. Eur Child Adolesc Psychiatry. 2019 Oct;28(10):1295-1310. Epub 2018 Aug 27. https://doi.org/10.1007/s00787-018-1215-z

412. Concepcion Zayas MT, Fortuna LR, Cullins LM. Depression in Latino and Immigrant Refugee Youth: Clinical Opportunities and Considerations. Child Adolesc Psychiatr Clin N Am. 2019 Jul;28(3):483-495. Epub 2019 Apr 4. https://doi.org/10.1016/j.chc.2019.02.013
413. Miller KK, Brown CR, Shramko M, Svetaz MV. Applying Trauma-Informed Practices to the Care of Refugee and Immigrant Youth: 10 Clinical Pearls. Children (Basel). 2019 Aug 20;6(8):94. https://doi.org/10.3390/children6080094
414. von Werthern M, Grigorakis G, Vizard E. The mental health and wellbeing of Unaccompanied Refugee Minors (URMs). Child Abuse Negl. 2019 Dec;98:104146. Epub 2019 Sep 12. https://doi.org/10.1016/j.chiabu.2019.104146
415. Hodes M, Vostanis P. Practitioner Review: Mental health problems of refugee children and adolescents and their management. J Child Psychol Psychiatry. 2019 Jul;60(7):716-731. Epub 2018 Dec 13. https://doi.org/10.1111/jcpp.13002
416. Mitra R, Hodes M. Prevention of psychological distress and promotion of resilience amongst unaccompanied refugee minors in resettlement countries. Child Care Health Dev. 2019 Mar;45(2):198-215. https://doi.org/10.1111/cch.12640
417. Bennouna C, Khauli N, Basir M, Allaf C, Wessells M, Stark L. School-based programs for Supporting the mental health and psychosocial wellbeing of adolescent forced migrants in high-income countries: A scoping review. Soc Sci Med. 2019 Oct;239:112558. Epub 2019 Sep 14. https://doi.org/10.1016/j.socscimed.2019.112558
418. van Os ECCC, Zijlstra AEE, Knorth EJE, Post WJW, Kalverboer MEM. Finding Keys: A Systematic Review of Barriers and Facilitators for Refugee Children's Disclosure of Their Life Stories. Trauma Violence Abuse. 2020 Apr;21(2):242-260. Epub 2018 Feb 20. https://doi.org/10.1177/1524838018757748
419. Blackmore R, Gray KM, Boyle JA, Fazel M, Ranasinha S, Fitzgerald G, Misso M, Gibson-Helm M. Systematic Review and Meta-analysis: The Prevalence of Mental Illness in Child and Adolescent Refugees and Asylum Seekers. J Am Acad Child Adolesc Psychiatry. 2020 Jun;59(6):705-714. Epub 2019 Nov 26. https://doi.org/10.1016/j.jaac.2019.11.011
420. Sarkadi A, Warner G, Salari R, Fängström K, Durbeej N, Lampa E, Baghdasaryan Z, Osman F, Gupta Löfving S, Perez Aronsson A, Feldman I, Sampaio F, Ssegonja R, Calam R, Bjärtå A, Leiler A, Rondung E, Wasteson E, Oppedal B, Keeshin B. Evaluation of the Teaching Recovery Techniques community-based intervention for unaccompanied refugee youth experiencing post-traumatic stress symptoms (Swedish UnaccomPanied yOuth Refugee Trial; SUPpORT): study protocol for a randomised controlled trial. Trials. 2020 Jan 10;21(1):63. https://doi.org/10.1186/s13063-019-3814-5
421. McNeely CA, Sprecher K, Bates-Fredi D, Price OA, Allen CD. Identifying Essential Components of School-Linked Mental Health Services for Refugee and Immigrant Children: A Comparative Case Study. J Sch Health. 2020 Jan;90(1):3-14. Epub 2019 Nov 28. https://doi.org/10.1111/josh.12845
422. Miller KE, Arnous M, Tossyeh F, Chen A, Bakolis I, Koppenol-Gonzalez GV, Nahas N, Jordans MJD. Protocol for a randomized control trial of the caregiver support intervention with Syrian refugees in Lebanon. Trials. 2020 Mar 18;21(1):277. https://doi.org/10.1186/s13063-020-4175-9
423. Rosner R, Sachser C, Hornfeck F, Kilian R, Kindler H, Muche R, Müller LRF, Thielemann J, Waldmann T, Ziegenhain U, Unterhitzenberger J, Pfeiffer E. Improving mental health care for unaccompanied young refugees through a stepped-care approach versus usual care+: study protocol of a cluster randomized controlled hybrid effectiveness implementation trial. Trials. 2020 Dec 9;21(1):1013. https://doi.org/10.1186/s13063-020-04922-x

424. Mares S. Mental health consequences of detaining children and families who seek asylum: a scoping review. Eur Child Adolesc Psychiatry. 2021 Oct;30(10):1615-1639. Epub 2020 Sep 14. https://doi.org/10.1007/s00787-020-01629-x
425. Scharpf F, Kaltenbach E, Nickerson A, Hecker T. A systematic review of socio-ecological factors contributing to risk and protection of the mental health of refugee children and adolescents. Clin Psychol Rev. 2021 Feb;83:101930. Epub 2020 Oct 20. https://doi.org/10.1016/j.cpr.2020.101930
426. Cohodes EM, Kribakaran S, Odriozola P, Bakirci S, McCauley S, Hodges HR, Sisk LM, Zacharek SJ, Gee DG. Migration-related trauma and mental health among migrant children emigrating from Mexico and Central America to the United States: Effects on developmental neurobiology and implications for policy. Dev Psychobiol. 2021 Sep;63(6):e22158. Epub 2021 Jul 22. https://doi.org/10.1002/dev.22158
427. Bamford J, Fletcher M, Leavey G. Mental Health Outcomes of Unaccompanied Refugee Minors: a Rapid Review of Recent Research. Curr Psychiatry Rep. 2021 Jul 1;23(8):46. https://doi.org/10.1007/s11920-021-01262-8
428. Arakelyan S, Ager A. Annual Research Review: A multilevel bioecological analysis of factors influencing the mental health and psychosocial well-being of refugee children. J Child Psychol Psychiatry. 2021 May;62(5):484-509. Epub 2020 Dec 5. https://doi.org/10.1111/jcpp.13355
429. Ünver H, Çeri V, Perdahlı Fiş N. An overview of the mental and physical health status and post-migration psychosocial stressors of refugee toddlers and preschoolers. J Child Adolesc Psychiatr Nurs. 2021 Nov;34(4):335-342. Epub 2021 Jun 14. https://doi.org/10.1111/jcap.12340
430. Alozkan Sever C, Cuijpers P, Mittendorfer-Rutz E, Bryant RA, Dawson KS, Holmes EA, Mooren T, Norredam ML, Sijbrandij M. Feasibility and acceptability of Problem Management Plus with Emotional Processing (PM+EP) for refugee youth living in the Netherlands: study protocol. Eur J Psychotraumatol. 2021 Aug 5;12(1):1947003. eCollection 2021. https://doi.org/10.1080/20008198.2021.1947003
431. Foka S, Hadfield K, Pluess M, Mareschal I. Promoting well-being in refugee children: An exploratory controlled trial of a positive psychology intervention delivered in Greek refugee camps. Dev Psychopathol. 2021 Feb;33(1):87-95. https://doi.org/10.1017/S0954579419001585
432. Chipalo E. Is Trauma Focused-Cognitive Behavioral Therapy (TF-CBT) Effective in Reducing Trauma Symptoms among Traumatized Refugee Children? A Systematic Review. J Child Adolesc Trauma. 2021 Jun 21;14(4):545-558. eCollection 2021 Dec. https://doi.org/10.1007/s40653-021-00370-0
433. Durbeej N, McDiarmid S, Sarkadi A, Feldman I, Punamäki RL, Kankaanpää R, Andersen A, Hilden PK, Verelst A, Derluyn I, Osman F. Evaluation of a school-based intervention to promote mental health of refugee youth in Sweden (The RefugeesWellSchool Trial): study protocol for a cluster randomized controlled trial. Trials. 2021 Jan 28;22(1):98. https://doi.org/10.1186/s13063-020-04995-8
434. Fine SL, Malik A, Guimond MF, Nemiro A, Temu G, Likindikoki S, Annan J, Tol WA. Improving mental health in low-resource settings: A feasibility randomized controlled trial of a transdiagnostic psychological intervention among Burundian refugee adolescents and their caregivers. Behav Res Ther. 2021 Oct;145:103944. Epub 2021 Aug 5. https://doi.org/10.1016/j.brat.2021.103944
435. Fischer LC, Kölligan V, Wieland N, Klein M. Development and Evaluation of a Digital Health Intervention for Substance Use Reduction in Young Refugees With Problematic Use of

Alcohol and/or Cannabis-Study Protocol for a Single-Armed Feasibility Trial. Front Public Health. 2021 Mar 31;9:557431. eCollection 2021. https://doi.org/10.3389/fpubh.2021.557431

436. Akhtar A, Malik A, Ghatasheh M, Aqel IS, Habashneh R, Dawson KS, Watts S, Jordans MJD, Brown F, Sijbrandij M, Cuijpers P, Bryant R. Feasibility trial of a brief scalable psychological intervention for Syrian refugee adolescents in Jordan. Eur J Psychotraumatol. 2021 Nov 29;12(1):1901408. eCollection 2021. https://doi.org/10.1080/20008198.2021.1901408

437. Jin SS, Dolan TM, Cloutier AA, Bojdani E, DeLisi L. Systematic review of depression and suicidality in child and adolescent (CAP) refugees. Psychiatry Res. 2021 Aug;302:114025. Epub 2021 May 21. https://doi.org/10.1016/j.psychres.2021.114025

438. Klas J, Grzywacz A, Kulszo K, Grunwald A, Kluz N, Makaryczew M, Samardakiewicz M. Challenges in the Medical and Psychosocial Care of the Paediatric Refugee-A Systematic Review. Int J Environ Res Public Health. 2022 Aug 26;19(17):10656. https://doi.org/10.3390/ijerph191710656

439. Hutchinson R, King N, Majumder P. How effective is group intervention in the treatment for unaccompanied and accompanied refugee minors with mental health difficulties: A systematic review. Int J Soc Psychiatry. 2022 May;68(3):484-499. Epub 2021 Nov 25. https://doi.org/10.1177/00207640211057727

440. Annous N, Al-Hroub A, El Zein F. A Systematic Review of Empirical Evidence on Art Therapy With Traumatized Refugee Children and Youth. Front Psychol. 2022 May 18;13:811515. eCollection 2022. https://doi.org/10.3389/fpsyg.2022.811515

441. Gadermann AM, Gagné Petteni M, Janus M, Puyat JH, Guhn M, Georgiades K. Prevalence of Mental Health Disorders Among Immigrant, Refugee, and Nonimmigrant Children and Youth in British Columbia, Canada. JAMA Netw Open. 2022 Feb 1;5(2):e2144934. https://doi.org/10.1001/jamanetworkopen.2021.44934

442. Demazure G, Baeyens C, Pinsault N. Review: Unaccompanied refugee minors' perception of mental health services and professionals: a systematic review of qualitative studies. Child Adolesc Ment Health. 2022 Sep;27(3):268-280. Epub 2021 Jun 15. https://doi.org/10.1111/camh.12486

443. Ünver H, Perdahlı Fiş N. An Analysis of Admissions to a Refugee Child Mental Health Unit in the Context of the COVID-19 Pandemic. Clin Child Psychol Psychiatry. 2022 Jan;27(1):136-144. Epub 2021 Nov 25. https://doi.org/10.1177/13591045211058337

444. Daniel-Calveras A, Baldaquí N, Baeza I. Mental health of unaccompanied refugee minors in Europe: A systematic review. Child Abuse Negl. 2022 Nov;133:105865. Epub 2022 Sep 9. https://doi.org/10.1016/j.chiabu.2022.105865

445. Velu ME, Martens I, Shahab M, de Roos C, Jongedijk RA, Schok M, Mooren T. Trauma-focused treatments for refugee children: study protocol for a randomized controlled trial of the effectiveness of KIDNET versus EMDR therapy versus a waitlist control group (KIEM). Trials. 2022 Apr 23;23(1):347. https://doi.org/10.1186/s13063-022-06178-z

446. Mulligan CJ, Clukay CJ, Matarazzo A, Hadfield K, Nevell L, Dajani R, Panter-Brick C. Novel GxE effects and resilience: A case:control longitudinal study of psychosocial stress with war-affected youth. PLoS One. 2022 Apr 4;17(4):e0266509. eCollection 2022. https://doi.org/10.1371/journal.pone.0266509

447. Soltan F, Cristofalo D, Marshall D, Purgato M, Taddese H, Vanderbloemen L, Barbui C, Uphoff E. Community-based interventions for improving mental health in refugee children and adolescents in high-income countries. Cochrane Database Syst Rev. 2022 May 9;5(5):CD013657. https://doi.org/10.1002/14651858.CD013657.pub2

448. Peltonen K, Aalto S, Vänskä M, Lepistö R, Punamäki RL, Soye E, Watters C, de Wal Pastoor L, Derluyn I, Kankaanpää R. Effectiveness of Promotive and Preventive Psychosocial Interventions on Improving the Mental Health of Finnish-Born and Immigrant Adolescents. Int J Environ Res Public Health. 2022 Mar 20;19(6):3686. https://doi.org/10.3390/ijerph19063686
449. Wittmann J, Groß M, Catani C, Schmidt T, Neldner S, Wilker S, May T, Ertl V, Rosner R, Zindler A, Odenwald M, Neuner F. The efficacy of Narrative Exposure Therapy for Children (KIDNET) as a treatment for traumatized young refugees versus treatment as usual: update to the study protocol for the multi-center randomized controlled trial YOURTREAT. Trials. 2022 Apr 27;23(1):360. https://doi.org/10.1186/s13063-022-06288-8
450. Brown FL, Bosqui T, Elias J, Farah S, Mayya A, Abo Nakkoul D, Walsh B, Chreif S, Einein A, Meksassi B, Abi Saad R, Naal H, Ghossainy ME, Donnelly M, Betancourt TS, Carr A, Puffer E, El Chammay R, Jordans MJD. Family systemic psychosocial support for at-risk adolescents in Lebanon: study protocol for a multi-site randomised controlled trial. Trials. 2022 Apr 18;23(1):327. https://doi.org/10.1186/s13063-022-06284-y
451. Oberg C, Sharma H. Post-Traumatic Stress Disorder in Unaccompanied Refugee Minors: Prevalence, Contributing and Protective Factors, and Effective Interventions: A Scoping Review. Children (Basel). 2023 May 26;10(6):941. https://doi.org/10.3390/children10060941
452. Al-Hroub A. Art Therapy Interventions for Syrian Child and Adolescent Refugees: Enhancing Mental Well-being and Resilience. Curr Psychiatry Rep. 2023 Dec;25(12):857-863. Epub 2023 Nov 9. https://doi.org/10.1007/s11920-023-01474-0
453. Herati H, Meyer SB. Mental health interventions for immigrant-refugee children and youth living in Canada: a scoping review and way forward. J Ment Health. 2023 Feb;32(1):276-289. Epub 2020 Sep 11. https://doi.org/10.1080/09638237.2020.1818710
454. Spaas C, Said-Metwaly S, Skovdal M, Primdahl NL, Jervelund SS, Hilden PK, Andersen AJ, Opaas M, Soye E, Watters C, Verelst A, Derluyn I, Colpin H, Haene L. School-based Psychosocial Interventions' Effectiveness in Strengthening Refugee and Migrant Adolescents' Mental Health, Resilience, and Social Relations: A Four-country Cluster Randomized Study. Psychosoc Interv. 2023 Aug 7;32(3):177-189. eCollection 2023 Aug. https://doi.org/10.5093/pi2023a12
455. Metzler J, Saw T, Nono D, Kadondi A, Zhang Y, Leu CS, Gabriel A, Savage K, Landers C. Improving adolescent mental health and protection in humanitarian settings: longitudinal findings from a multi-arm randomized controlled trial of child-friendly spaces among South Sudanese refugees in Uganda. J Child Psychol Psychiatry. 2023 Jun;64(6):907-917. Epub 2023 Jan 2. https://doi.org/10.1111/jcpp.13746
456. Lee K, Kronick R, Miconi D, Rousseau C. Moving Forward in Mental Health Care for Refugee, Asylum-Seeking, and Undocumented Children: Social Determinants, Phased Approach to Care, and Advocacy. Child Adolesc Psychiatr Clin N Am. 2024 Apr;33(2):237-250. Epub 2023 Nov 3. https://doi.org/10.1016/j.chc.2023.09.007
457. Andersson J, Kankaanpää R, Peltonen K, Münger AC, Korhonen L. Examining heterogeneity: A systematic review of quantitative person-centered studies on adversity, mental health, and resilience in children and young adults with refugee backgrounds. Compr Psychiatry. 2024 Nov;135:152522. Epub 2024 Aug 8. https://doi.org/10.1016/j.comppsych.2024.152522
458. B Yonis O, Khader Y, Taha H, Al-Madhwahi A, Khudair SA, Tanaka E, Nsour MA. Psychosocial and emotional well-being of Syrian refugee children and adolescents in Jordan:

In-camp versus out-of-camp comparative analysis. Narra J. 2024 Aug;4(2):e849. Epub 2024 Aug 2. https://doi.org/10.52225/narra.v4i2.849
459. Otika D, Odongo G, Muzaki RM, Lamwaka BO, Bongomin F, Pebolo PF. Depression and suicidal ideation among adolescent girls in refugee settlements in northern Uganda. Medicine (Baltimore). 2024 May 10;103(19):e38077. https://doi.org/10.1097/MD.0000000000038077
460. Della Rocca B, Bello R, Carbone M, Pezzella P, Toni C, Sampogna G, Tarsitani L, Luciano M, Fiorillo A. Promoting mental health and preventing mental health problems in child and adolescent refugees and asylum seekers: A systematic review on psychosocial interventions. Int J Soc Psychiatry. 2024 Jun;70(4):653-666. Epub 2023 Dec 9. https://doi.org/10.1177/00207640231214964
461. Ali R, Brown FL, Stevenson K, Jordans M, Taha K, Amine ME, Steen F, Meksassi B, Elias J, Aoun M, Roberts B, Sijbrandij M, Cuijpers P, Akhtar A, Malik A, Woodward A, Fuhr DC; STRENGTHS Consortium. Implementing a Non-Specialist Delivered Psychological Intervention for Young Adolescents in a Protracted Refugee Setting: a Qualitative Process Evaluation in Lebanon. J Behav Health Serv Res. 2024 Jul;51(3):377-394. Epub 2023 Dec 12. https://doi.org/10.1007/s11414-023-09870-3
462. Giles CJ, Västhagen M, van Leuven L, Edenius A, Ghaderi A, Enebrink P. The efficacy of psychological prevention, and health promotion interventions targeting psychological health, wellbeing or resilience among forced migrant children and youth: a systematic review and meta-analysis. Eur Child Adolesc Psychiatry. 2025 Jan;34(1):123-140. Epub 2024 Apr 16. https://doi.org/10.1007/s00787-024-02424-8
463. Logie CH, Okumu M, Tailor L, MacKenzie F, Admassu Z, Hakiza R, Kibuuka Musoke D, Katisi B, Nakitende A, Kyambadde P, Mbuagbaw L. Tushirikiane-4-Uthabiti (Supporting Each Other For Resilience): study protocol of a mental health, HIV self-testing and livelihoods randomised controlled trial for advancing HIV prevention outcomes among urban refugee youth in Kampala, Uganda. BMJ Open. 2024 Nov 24;14(11):e087470. https://doi.org/10.1136/bmjopen-2024-087470
464. Alhasanat D, Giurgescu C. Acculturation and Postpartum Depressive Symptoms among Hispanic Women in the United States: Systematic Review. MCN Am J Matern Child Nurs. 2017 Jan/Feb;42(1):21-28. https://doi.org/10.1097/NMC.0000000000000298
465. Tobin CL, Di Napoli P, Beck CT. Refugee and Immigrant Women's Experience of Postpartum Depression: A Meta-Synthesis. J Transcult Nurs. 2018 Jan;29(1):84-100. Epub 2017 Jan 16. https://doi.org/10.1177/1043659616686167
466. Kassam S. Understanding Experiences of Social Support as Coping Resources among Immigrant and Refugee Women with Postpartum Depression: An Integrative Literature Review. Issues Ment Health Nurs. 2019 Dec;40(12):999-1011. Epub 2019 May 9. https://doi.org/10.1080/01612840.2019.1585493
467. Sabri B, Njie-Carr VPS, Messing JT, Glass N, Brockie T, Hanson G, Case J, Campbell JC. The weWomen and ourCircle randomized controlled trial protocol: A web-based intervention for immigrant, refugee and indigenous women with intimate partner violence experiences. Contemp Clin Trials. 2019 Jan;76:79-84. Epub 2018 Dec 2. https://doi.org/10.1016/j.cct.2018.11.013
468. Rees SJ, Fisher JR, Steel Z, Mohsin M, Nadar N, Moussa B, Hassoun F, Yousif M, Krishna Y, Khalil B, Mugo J, Tay AK, Klein L, Silove D. Prevalence and Risk Factors of Major Depressive Disorder Among Women at Public Antenatal Clinics From Refugee, Conflict-Affected, and Australian-Born Backgrounds. JAMA Netw Open. 2019 May 3;2(5):e193442. https://doi.org/10.1001/jamanetworkopen.2019.3442

469. Lai H, Due C, Ziersch A. The relationship between employment and health for people from refugee and asylum-seeking backgrounds: A systematic review of quantitative studies. SSM Popul Health. 2022 Mar 30;18:101075. eCollection 2022 Jun. https://doi.org/10.1016/j.ssmph.2022.101075
470. Gillespie S, Banegas J, Maxwell J, Chan ACY, Darawshy NA, Wasil AR, Marsalis S, Gewirtz A. Parenting Interventions for Refugees and Forcibly Displaced Families: A Systematic Review. Clin Child Fam Psychol Rev. 2022 Jun;25(2):395-412. Epub 2022 Jan 10. https://doi.org/10.1007/s10567-021-00375-z
471. Abdi S, Akinsulure-Smith AM, Sarkadi A, Fazel M, Ellis BH, Gillespie S, Juang LP, Betancourt TS. Promoting positive development among refugee adolescents. J Res Adolesc. 2023 Dec;33(4):1064-1084. Epub 2023 Oct 9. https://doi.org/10.1111/jora.12890
472. Gagnon AJ, Tuck J, Barkun L. A systematic review of questionnaires measuring the health of resettling refugee women. Health Care Women Int. 2004 Feb;25(2):111-49. https://doi.org/10.1080/07399330490267503
473. Roberts F, Teague B, Lee J, Rushworth I. The Prevalence of Burnout and Secondary Traumatic Stress in Professionals and Volunteers Working With Forcibly Displaced People: A Systematic Review and Two Meta-Analyses. J Trauma Stress. 2021 Aug;34(4):773-785. Epub 2021 Mar 27. https://doi.org/10.1002/jts.22659
474. Halcón LL, Robertson CL, Monsen KA, Claypatch CC. A theoretical framework for using health realization to reduce stress and improve coping in refugee communities. J Holist Nurs. 2007 Sep;25(3):186-94. https://doi.org/10.1177/0898010107303275
475. Mills E, Singh S, Roach B, Chong S. Prevalence of mental disorders and torture among Bhutanese refugees in Nepal: a systemic review and its policy implications. Med Confl Surviv. 2008 Jan-Mar;24(1):5-15. https://doi.org/10.1080/13623690701775171
476. Weinstein N, Khabbaz F, Legate N. Enhancing need satisfaction to reduce psychological distress in Syrian refugees. J Consult Clin Psychol. 2016 Jul;84(7):645-50. Epub 2016 Mar 28. https://doi.org/10.1037/ccp0000095
477. Posselt M, McIntyre H, Ngcanga M, Lines T, Procter N. The mental health status of asylum seekers in middle- to high-income countries: a synthesis of current global evidence. Br Med Bull. 2020 Jul 9;134(1):4-20. https://doi.org/10.1093/bmb/ldaa010
478. Lu J, Jamani S, Benjamen J, Agbata E, Magwood O, Pottie K. Global Mental Health and Services for Migrants in Primary Care Settings in High-Income Countries: A Scoping Review. Int J Environ Res Public Health. 2020 Nov 20;17(22):8627. https://doi.org/10.3390/ijerph17228627
479. Goodkind JR, Bybee D, Hess JM, Amer S, Ndayisenga M, Greene RN, Choe R, Isakson B, Baca B, Pannah M. Randomized Controlled Trial of a Multilevel Intervention to Address Social Determinants of Refugee Mental Health. Am J Community Psychol. 2020 Jun;65(3-4):272-289. Epub 2020 Feb 17. https://doi.org/10.1002/ajcp.12418
480. Taft A, Young F, Hegarty K, Yelland J, Mazza D, Boyle D, Norman R, Garcia-Moreno C, Nguyen CD, Li X, Pokharel B, Allen M, Feder G. HARMONY: a pragmatic cluster randomised controlled trial of a culturally competent systems intervention to prevent and reduce domestic violence among migrant and refugee families in general practice: study protocol. BMJ Open. 2021 Jul 29;11(7):e046431. https://doi.org/10.1136/bmjopen-2020-046431
481. Dualle MA, Robinette LM, Hatsu IE. Food Related Challenges and Mental Health Among U.S. African Migrants: A Narrative Review. J Immigr Minor Health. 2024 Apr;26(2):371-384. Epub 2023 Jul 4. https://doi.org/10.1007/s10903-023-01512-2

482. Cheng IH, Drillich A, Schattner P. Refugee experiences of general practice in countries of resettlement: a literature review. Br J Gen Pract. 2015 Mar;65(632):e171-6. https://doi.org/10.3399/bjgp15X683977
483. Waggoner-Fountain LA. Management of Refugees and International Adoptees. Pediatr Clin North Am. 2017 Aug;64(4):953-960. https://doi.org/10.1016/j.pcl.2017.03.011
484. Shishehgar S, Gholizadeh L, DiGiacomo M, Green A, Davidson PM. Health and Socio-Cultural Experiences of Refugee Women: An Integrative Review. J Immigr Minor Health. 2017 Aug;19(4):959-973. https://doi.org/10.1007/s10903-016-0379-1
485. Boylen S, Cherian S, Gill FJ, Leslie GD, Wilson S. Impact of professional interpreters on outcomes for hospitalized children from migrant and refugee families with limited English proficiency: a systematic review. JBI Evid Synth. 2020 Jul;18(7):1360-1388. https://doi.org/10.11124/JBISRIR-D-19-00300
486. Behnke NL, Cronk R, Shackelford BB, Cooper B, Tu R, Heller L, Bartram J. Environmental health conditions in protracted displacement: A systematic scoping review. Sci Total Environ. 2020 Jul 15;726:138234. Epub 2020 Apr 12. https://doi.org/10.1016/j.scitotenv.2020.138234
487. Rass E, Lokot M, Brown FL, Fuhr DC, Asmar MK, Smith J, McKee M, Orm IB, Yeretzian JS, Roberts B. Participation by conflict-affected and forcibly displaced communities in humanitarian healthcare responses: A systematic review. J Migr Health. 2020 Dec 9;1-2:100026. eCollection 2020. https://doi.org/10.1016/j.jmh.2020.100026
488. Walden J. Caring for the Forcibly Displaced. Prim Care. 2021 Mar;48(1):1-7. Epub 2020 Nov 26. https://doi.org/10.1016/j.pop.2020.11.001
489. Taki F, de Melo-Martin I. Conducting epigenetics research with refugees and asylum seekers: attending to the ethical challenges. Clin Epigenetics. 2021 May 8;13(1):105. https://doi.org/10.1186/s13148-021-01092-8
490. Makoni M. Insulin storage without refrigeration. Lancet Diabetes Endocrinol. 2021 Apr;9(4):202. https://doi.org/10.1016/S2213-8587(21)00057-7
491. Lau LS, Rodgers G. Cultural Competence in Refugee Service Settings: A Scoping Review. Health Equity. 2021 Mar 16;5(1):124-134. eCollection 2021. https://doi.org/10.1089/heq.2020.0094
492. Siddiq H, Rosenberg J. Clinicians as advocates amid refugee resettlement agency closures. J Public Health Policy. 2021 Sep;42(3):477-492. Epub 2021 Jul 21. https://doi.org/10.1057/s41271-021-00296-9
493. Corte-Real A, Nunes T, Rupino da Cunha P. Blockchain technology in migrant and refugee health: A scoping review. J Glob Health. 2022 May 14;12:04047. https://doi.org/10.7189/jogh.12.04047
494. Reißmann S, Flothow A, Harth V, Mache S. Exploring job demands and resources in psychotherapists treating displaced people-A scoping review. Psychother Res. 2022 Nov;32(8):1076-1089. Epub 2022 May 11. https://doi.org/10.1080/10503307.2022.2071653
495. Villalonga-Olives E, Wind TR, Armand AO, Yirefu M, Smith R, Aldrich DP. Social-capital-based mental health interventions for refugees: A systematic review. Soc Sci Med. 2022 May;301:114787. Epub 2022 Feb 19. https://doi.org/10.1016/j.socscimed.2022.114787
496. Sadana A, Gérard SE, Tang A, Pardee L, Sher O, Foote A, Ades V. Medical evidence in asylum applications: Medical versus legal approaches. J Forensic Leg Med. 2023 Jul;97:102553. Epub 2023 Jun 21. https://doi.org/10.1016/j.jflm.2023.102553

497. Jain R, Stone GS, Gartland MG. Medical Care for Newly Arrived Displaced Persons. NEJM Evid. 2023 Nov;2(11):EVIDra2200286. Epub 2023 Oct 24. https://doi.org/10.1056/EVIDra2200286
498. Farnham DDT, Goldstone R. A narrative review of refugee & asylum seekers' transitions into & experiences of working in the United Kingdom National Health Service. BMC Health Serv Res. 2023 Jun 13;23(1):622. https://doi.org/10.1186/s12913-023-09606-1
499. Davidson N, Hammarberg K, Fisher J. Ethical Considerations in Research With People From Refugee and Asylum Seeker Backgrounds: A Systematic Review of National and International Ethics Guidelines. J Bioeth Inq. 2024 Jun;21(2):261-284. Epub 2023 Oct 27. https://doi.org/10.1007/s11673-023-10297-w
500. Molyneux K, Singer E. Asylum seekers and the role of the acute care physician. J Am Coll Emerg Physicians Open. 2024 Jun 16;5(3):e13196. eCollection 2024 Jun. https://doi.org/10.1002/emp2.13196
501. Hosseini Z, Motamedi M. Home, School, and Community-based Services for Forcibly Displaced Youth and Their Families. Child Adolesc Psychiatr Clin N Am. 2024 Oct;33(4):677-692. Epub 2024 Apr 27. https://doi.org/10.1016/j.chc.2024.03.015
502. Ngo T, Hodes M. Pervasive refusal syndrome in asylum-seeking children: Review of the current evidence. Clin Child Psychol Psychiatry. 2020 Jan;25(1):227-241. Epub 2019 May 19. https://doi.org/10.1177/1359104519846580
503. Porignon D, Katulanya I, Elongo L, Ntalemwa N, Tonglet R, Dramaix M, Hennart P. The unseen face of humanitarian crisis in eastern Democratic Republic of Congo: was nutritional relief properly targeted?. J Epidemiol Community Health. 2000 Jan;54(1):6-9. https://doi.org/10.1136/jech.54.1.6
504. Roberts L, Chartier Y, Chartier O, Malenga G, Toole M, Rodka H. Keeping clean water clean in a Malawi refugee camp: a randomized intervention trial. Bull World Health Organ. 2001;79(4):280-7. Epub 2003 Jul 2.
505. National Research Council (US) Roundtable on the Demography of Forced Migration; Reed HE, Keely CB, editors. Forced Migration & Mortality. Washington (DC): National Academies Press (US); 2001.
506. Strumpf NE, Glicksman A, Goldberg-Glen RS, Fox RC, Logue EH. Caregiver and elder experiences of Cambodian, Vietnamese, Soviet Jewish, and Ukrainian refugees. Int J Aging Hum Dev. 2001;53(3):233-52. https://doi.org/10.2190/PXUG-J0T8-DGUK-08MD
507. Riner ME, Becklenberg A. Partnering with a sister city organization for an international service-learning experience. J Transcult Nurs. 2001 Jul;12(3):234-40. https://doi.org/10.1177/104365960101200308
508. Porter M, Haslam N. Forced displacement in Yugoslavia: a meta-analysis of psychological consequences and their moderators. J Trauma Stress. 2001 Oct;14(4):817-34. https://doi.org/10.1023/A:1013054524810
509. Burkle FM Jr, Hayden R. The concept of assisted management of large-scale disasters by horizontal organizations. Prehosp Disaster Med. 2001 Jul-Sep;16(3):128-37. https://doi.org/10.1017/s1049023x00025875
510. National Research Council (US) Roundtable on the Demography of Forced Migration. Demographic Assessment Techniques in Complex Humanitarian Emergencies: Summary of a Workshop. Washington (DC): National Academies Press (US); 2002.
511. Donev D, Onceva S, Gligorov I. Refugee crisis in Macedonia during the Kosovo conflict in 1999. Croat Med J. 2002 Apr;43(2):184-9.

512. Beyrer C, Kass NE. Human rights, politics, and reviews of research ethics. Lancet. 2002 Jul 20;360(9328):246-51. https://doi.org/10.1016/S0140-6736(02)09465-5
513. Kuhns DH. Globalizing the PA profession. JAAPA. 2002 Oct;15(10):45-8, 50.
514. VanRooyen MJ. Development of prehospital emergency medical services: strategies for system assessment and planning. Pac Health Dialog. 2002 Mar;9(1):86-92.
515. Duerr A, Posner SF, Gilbert M. Evidence in support of foster care during acute refugee crises. Am J Public Health. 2003 Nov;93(11):1904-9. https://doi.org/10.2105/ajph.93.11.1904
516. Bhatia R, Thorne-Lyman A. Food aid in emergencies and public health nutrition. Forum Nutr. 2003;56:391-4.
517. Weine SM, Raina D, Zhubi M, Delesi M, Huseni D, Feetham S, Kulauzovic Y, Mermelstein R, Campbell RT, Rolland J, Pavkovic I. The TAFES multi-family group intervention for Kosovar refugees: a feasibility study. J Nerv Ment Dis. 2003 Feb;191(2):100-7. https://doi.org/10.1097/01.NMD.0000050938.06620.D2
518. Kaiser R, Spiegel PB, Henderson AK, Gerber ML. The application of geographic information systems and global positioning systems in humanitarian emergencies: lessons learned, programme implications and future research. Disasters. 2003 Jun;27(2):127-40. https://doi.org/10.1111/1467-7717.00224
519. Bradt DA. Site management of health issues in the 2001 World Trade Center disaster. Acad Emerg Med. 2003 Jun;10(6):650-60. https://doi.org/10.1111/j.1553-2712.2003.tb00051.x
520. Nelson BD, Simic S, Beste L, Vukovic D, Bjegovic V, VanRooyen MJ. Multimodal assessment of the primary healthcare system of Serbia: a model for evaluating post-conflict health systems. Prehosp Disaster Med. 2003 Jan-Mar;18(1):6-13. https://doi.org/10.1017/s1049023x00000613
521. Guruge S, Khanlou N. Intersectionalities of influence: researching the health of immigrant and refugee women. Can J Nurs Res. 2004 Sep;36(3):32-47.
522. Adams KM, Gardiner LD, Assefi N. Healthcare challenges from the developing world: post-immigration refugee medicine. BMJ. 2004 Jun 26;328(7455):1548-52. https://doi.org/10.1136/bmj.328.7455.1548
523. Akiyama H. Market principles in health care and social security policy in Japan. World Hosp Health Serv. 2004;40(2):16-22, 40-2.
524. Halabi JO. Nursing research with refugee clients: a call for more qualitative approaches. Int Nurs Rev. 2005 Dec;52(4):270-5. https://doi.org/10.1111/j.1466-7657.2005.00440.x
525. Clarkin PF. Methodological issues in the anthropometric assessment of Hmong children in the United States. Am J Hum Biol. 2005 Nov-Dec;17(6):787-95. https://doi.org/10.1002/ajhb.20438
526. Boehnlein JK, Schaefer MN, Bloom JD. Cultural considerations in the criminal law: the sentencing process. J Am Acad Psychiatry Law. 2005;33(3):335-41.
527. VanRooyen M, Venugopal R, Greenough PG. International humanitarian assistance: where do emergency physicians belong?. Emerg Med Clin North Am. 2005 Feb;23(1):115-31. https://doi.org/10.1016/j.emc.2004.09.006
528. Hsu EB, Dey CC, Scheulen JJ, Bledsoe GH, VanRooyen MJ. Development of emergency medicine administration in the People's Republic of China. J Emerg Med. 2005 Feb;28(2):231-6. https://doi.org/10.1016/j.jemermed.2004.07.014
529. Herlihy J, Turner S. Should discrepant accounts given by asylum seekers be taken as proof of deceit?. Torture. 2006;16(2):81-92.

530. Pan A, Daley S, Rivera LM, Williams K, Lingle D, Reznik V. Understanding the role of culture in domestic violence: the Ahimsa Project for Safe Families. J Immigr Minor Health. 2006 Jan;8(1):35-43. https://doi.org/10.1007/s10903-006-6340-y
531. Dyhr L, Andersen JS. [Patterns of contact with general practice in the daytime by guest workers with immigrant and refugee background in Copenhagen municipality, 1998]. Ugeskr Laeger. 2006 Sep 18;168(38):3217-22.
532. Abdel-Khalek AM, Al-Arja NS, Abdalla T. Death obsession in Palestinians. Death Stud. 2006 Apr;30(3):203-15. https://doi.org/10.1080/07481180500493302
533. Willis MS, Nkwocha O. Health and related factors for Sudanese refugees in Nebraska. J Immigr Minor Health. 2006 Jan;8(1):19-33. https://doi.org/10.1007/s10903-006-6339-9
534. Hargreaves S, Friedland JS, Gothard P, Saxena S, Millington H, Eliahoo J, Le Feuvre P, Holmes A. Impact on and use of health services by international migrants: questionnaire survey of inner city London A&E attenders. BMC Health Serv Res. 2006 Nov 29;6:153. https://doi.org/10.1186/1472-6963-6-153
535. Andersen JS, Dyhr L. [Patterns of contact with the out-of-hours service and emergency rooms by guest workers with immigrant and refugee background in Copenhagen municipality, 1998]. Ugeskr Laeger. 2006 Sep 18;168(38):3222-7.
536. McMahon JD, Macfarlane A, Avalos GE, Cantillon P, Murphy AW. A survey of asylum seekers' general practice service utilisation and morbidity patterns. Ir Med J. 2007 May;100(5):461-4.
537. Carlock DM. Finding information on immigrant and refugee health. J Transcult Nurs. 2007 Oct;18(4):373-9. https://doi.org/10.1177/1043659607305196
538. Feldmann CT, Bensing JM, de Ruijter A. Worries are the mother of many diseases: general practitioners and refugees in the Netherlands on stress, being ill and prejudice. Patient Educ Couns. 2007 Mar;65(3):369-80. Epub 2006 Nov 20. https://doi.org/10.1016/j.pec.2006.09.005
539. Porter M. Global evidence for a biopsychosocial understanding of refugee adaptation. Transcult Psychiatry. 2007 Sep;44(3):418-39. https://doi.org/10.1177/1363461507081639
540. Griswold K, Zayas LE, Kernan JB, Wagner CM. Cultural awareness through medical student and refugee patient encounters. J Immigr Minor Health. 2007 Jan;9(1):55-60. https://doi.org/10.1007/s10903-006-9016-8
541. Tripathy V, Gupta R. Growth among Tibetans at high and low altitudes in India. Am J Hum Biol. 2007 Nov-Dec;19(6):789-800. https://doi.org/10.1002/ajhb.20638
542. Cohen Y, Haberfeld Y. Self-selection and earnings assimilation: immigrants from the former Soviet Union in Israel and the United States. Demography. 2007 Aug;44(3):649-68. https://doi.org/10.1353/dem.2007.0023
543. Todd EC, Greig JD, Bartleson CA, Michaels BS. Outbreaks where food workers have been implicated in the spread of foodborne disease. Part 2. Description of outbreaks by size, severity, and settings. J Food Prot. 2007 Aug;70(8):1975-93. https://doi.org/10.4315/0362-028x-70.8.1975
544. Stauffer WM, Weinberg M. Emerging clinical issues in refugees. Curr Opin Infect Dis. 2009 Oct;22(5):436-42. https://doi.org/10.1097/QCO.0b013e32832f14a4
545. Kushner AL, Groen RS, Kingham TP. Surgery and refugee populations. Scand J Surg. 2009;98(1):18-24. https://doi.org/10.1177/145749690909800104
546. Inter-Agency Field Manual on Reproductive Health in Humanitarian Settings: 2010 Revision for Field Review. Geneva: Inter-agency Working Group on Reproductive Health in Crises; 2010.

547. Williams N. Establishing the boundaries and building bridges: a literature review on ecological theory: implications for research into the refugee parenting experience. J Child Health Care. 2010 Mar;14(1):35-51. Epub 2009 Nov 20. https://doi.org/10.1177/1367493509347116
548. Perron NJ, Dao MD, Kossovsky MP, Miserez V, Chuard C, Calmy A, Gaspoz JM. Reduction of missed appointments at an urban primary care clinic: a randomised controlled study. BMC Fam Pract. 2010 Oct 25;11:79. https://doi.org/10.1186/1471-2296-11-79
549. Phillips C, Narayanasamy S. Proof of age required--estimating age in adults without birth records. Aust Fam Physician. 2010 Jul;39(7):518-21.
550. Vincent JE, Netek S, Parry A, Mladenovich D, Thein NN, Amendola PR. Reported wearing compliance of ready-made spectacles at 6 and 12 months. Optom Vis Sci. 2010 Dec;87(12):958-65. https://doi.org/10.1097/OPX.0b013e3181fef3a9
551. Wros P, Archer S. Comparing learning outcomes of international and local community partnerships for undergraduate nursing students. J Community Health Nurs. 2010 Oct;27(4):216-25. https://doi.org/10.1080/07370016.2010.515461
552. Khoo SE. Health and humanitarian migrants' economic participation. J Immigr Minor Health. 2010 Jun;12(3):327-39. https://doi.org/10.1007/s10903-007-9098-y
553. Mowafi H. Conflict, displacement and health in the Middle East. Glob Public Health. 2011;6(5):472-87. Epub 2011 May 16. https://doi.org/10.1080/17441692.2011.570358
554. Williams N. A critical review of the literature: engendering the discourse of masculinities matter for parenting African refugee men. Am J Mens Health. 2011 Mar;5(2):104-17. Epub 2009 Dec 29. https://doi.org/10.1177/1557988309346055
555. Meyer S, Tappis H, Weiss W, Spiegel P, Vu A. Refugee site health service utilization: more needs to be done. Am J Disaster Med. 2011 Jul-Aug;6(4):231-42. https://doi.org/10.5055/ajdm.2011.0062
556. Correa-Velez I, Barnett AG, Gifford SM, Sackey D. Health status and use of health services among recently arrived men with refugee backgrounds: a comparative analysis of urban and regional settlement in South-east Queensland. Aust J Prim Health. 2011;17(1):66-71. https://doi.org/10.1071/PY10051
557. Eckstein B. Primary care for refugees. Am Fam Physician. 2011 Feb 15;83(4):429-36.
558. Tugwell P, Pottie K, Welch V, Ueffing E, Chambers A, Feightner J; Canadian Collaboration for Immigrant and Refugee Health (CCIRH). Evaluation of evidence-based literature and formulation of recommendations for the clinical preventive guidelines for immigrants and refugees in Canada. CMAJ. 2011 Sep 6;183(12):E933-8. Epub 2010 Jun 23. https://doi.org/10.1503/cmaj.090289
559. Inhorn MC, Serour GI. Islam, medicine, and Arab-Muslim refugee health in America after 9/11. Lancet. 2011 Sep 3;378(9794):935-43. https://doi.org/10.1016/S0140-6736(11)61041-6
560. Norredam M, Kastrup M, Helweg-Larsen K. Register-based studies on migration, ethnicity, and health. Scand J Public Health. 2011 Jul;39(7 Suppl):201-5. https://doi.org/10.1177/1403494810396561
561. Gushulak BD, Pottie K, Hatcher Roberts J, Torres S, DesMeules M; Canadian Collaboration for Immigrant and Refugee Health. Migration and health in Canada: health in the global village. CMAJ. 2011 Sep 6;183(12):E952-8. Epub 2010 Jun 28. https://doi.org/10.1503/cmaj.090287

562. Jackson JC, Nguyen D, Hu N, Harris R, Terasaki GS. Alterations in medical interpretation during routine primary care. J Gen Intern Med. 2011 Mar;26(3):259-64. Epub 2010 Oct 5. https://doi.org/10.1007/s11606-010-1519-2
563. Longacre M, Silver-Highfield E, Lama P, Grodin M. Complementary and alternative medicine in the treatment of refugees and survivors of torture: a review and proposal for action. Torture. 2012;22(1):38-57.
564. Tappis H, Doocy S, Haskew C, Wilkinson C, Oman A, Spiegel P. United Nations High Commissioner for Refugees feeding program performance in Kenya and Tanzania: a retrospective analysis of routine Health Information System data. Food Nutr Bull. 2012 Jun;33(2):150-60. https://doi.org/10.1177/156482651203300209
565. Prabhu M, Baranoski M. Forensic mental health professionals in the immigration process. Psychiatr Clin North Am. 2012 Dec;35(4):929-46. https://doi.org/10.1016/j.psc.2012.08.012
566. Rah JH, dePee S, Kraemer K, Steiger G, Bloem MW, Spiegel P, Wilkinson C, Bilukha O. Program experience with micronutrient powders and current evidence. J Nutr. 2012 Jan;142(1):191S-6S. Epub 2011 Nov 30. https://doi.org/10.3945/jn.111.140004
567. Irfan FB, Irfan BB, Spiegel DA. Barriers to accessing surgical care in Pakistan: healthcare barrier model and quantitative systematic review. J Surg Res. 2012 Jul;176(1):84-94. Epub 2011 Aug 27. https://doi.org/10.1016/j.jss.2011.07.046
568. AlHeresh R, Bryant W, Holm M. Community-based rehabilitation in Jordan: challenges to achieving occupational justice. Disabil Rehabil. 2013 Oct;35(21):1848-52. Epub 2013 Jan 24. https://doi.org/10.3109/09638288.2012.756944
569. Leblanc Y, Bourgeault IL, Neiterman E. Comparing approaches to integrating refugee and asylum-seeking healthcare professionals in Canada and the UK. Healthc Policy. 2013 Oct;9(Spec Issue):126-38.
570. Ogilvie L, Higginbottom G, Burgess-Pinto E, Murray C. Fostering excellence: development of a course to prepare graduate students for research on migration and health. Nurs Inq. 2013 Sep;20(3):211-22. Epub 2012 May 25. https://doi.org/10.1111/j.1440-1800.2012.00605.x
571. Edge S, Newbold B. Discrimination and the health of immigrants and refugees: exploring Canada's evidence base and directions for future research in newcomer receiving countries. J Immigr Minor Health. 2013 Feb;15(1):141-8. https://doi.org/10.1007/s10903-012-9640-4
572. Sethi B. Service delivery on rusty health care wheels: implications for visible minority women. J Evid Based Soc Work. 2013 Oct;10(5):522-32. https://doi.org/10.1080/15433714.2012.760986
573. Kiss V, Pim C, Hemmelgarn BR, Quan H. Building knowledge about health services utilization by refugees. J Immigr Minor Health. 2013 Feb;15(1):57-67. https://doi.org/10.1007/s10903-011-9528-8
574. Joshi C, Russell G, Cheng IH, Kay M, Pottie K, Alston M, Smith M, Chan B, Vasi S, Lo W, Wahidi SS, Harris MF. A narrative synthesis of the impact of primary health care delivery models for refugees in resettlement countries on access, quality and coordination. Int J Equity Health. 2013 Nov 7;12:88. https://doi.org/10.1186/1475-9276-12-88
575. Rowley E, Burns L, Burnham G. Research review of nongovernmental organizations' security policies for humanitarian programs in war, conflict, and postconflict environments. Disaster Med Public Health Prep. 2013 Jun;7(3):241-50. https://doi.org/10.1001/dmp.2010.0723

576. Campbell RM, Klei AG, Hodges BD, Fisman D, Kitto S. A comparison of health access between permanent residents, undocumented immigrants and refugee claimants in Toronto, Canada. J Immigr Minor Health. 2014 Feb;16(1):165-76. https://doi.org/10.1007/s10903-012-9740-1
577. Highfield ES, Longacre M, Sager A, Grodin MA. A preliminary comparison of primary care use by refugees before and after acupuncture. J Complement Integr Med. 2014 Dec;11(4):289-95. https://doi.org/10.1515/jcim-2014-0001
578. Clifford V, Rhodes A, Paxton G. Learning difficulties or learning English difficulties? Additional language acquisition: an update for paediatricians. J Paediatr Child Health. 2014 Mar;50(3):175-81. Epub 2013 Oct 18. https://doi.org/10.1111/jpc.12396
579. Mpinga EK, Frey C, Chastonay P. Economic burden of torture for a refugee host country: development of a model and presentation of a country case study. Clinicoecon Outcomes Res. 2014 Apr 2;6:165-73. eCollection 2014. https://doi.org/10.2147/CEOR.S56431
580. Ehiri JE, Gunn JK, Center KE, Li Y, Rouhani M, Ezeanolue EE. Training and deployment of lay refugee/internally displaced persons to provide basic health services in camps: a systematic review. Glob Health Action. 2014 Oct 1;7:23902. eCollection 2014. https://doi.org/10.3402/gha.v7.23902
581. Pollard RQ Jr, Betts WR, Carroll JK, Waxmonsky JA, Barnett S, deGruy FV 3rd, Pickler LL, Kellar-Guenther Y. Integrating primary care and behavioral health with four special populations: Children with special needs, people with serious mental illness, refugees, and deaf people. Am Psychol. 2014 May-Jun;69(4):377-87. https://doi.org/10.1037/a0036220
582. Harstad I, Henriksen AH, Sagvik E. Collaboration between municipal and specialist public health care in tuberculosis screening in Norway. BMC Health Serv Res. 2014 May 27;14:238. https://doi.org/10.1186/1472-6963-14-238
583. Pimentel VM, Eckardt MJ. More than interpreters needed: the specialized care of the immigrant pregnant patient. Obstet Gynecol Surv. 2014 Aug;69(8):490-500. https://doi.org/10.1097/OGX.0000000000000099
584. Horvat L, Horey D, Romios P, Kis-Rigo J. Cultural competence education for health professionals. Cochrane Database Syst Rev. 2014 May 5;2014(5):CD009405. https://doi.org/10.1002/14651858.CD009405.pub2
585. Szajna A, Ward J. Access to health care by refugees: a dimensional analysis. Nurs Forum. 2015 Apr-Jun;50(2):83-9. Epub 2014 Jan 3. https://doi.org/10.1111/nuf.12051
586. Dharod JM. What changes upon resettlement: understanding difference in pre- and post-resettlement dietary habits among South-Asian refugees. Ecol Food Nutr. 2015;54(3):209-23. Epub 2014 Dec 31. https://doi.org/10.1080/03670244.2014.964800
587. Girard C. Immigrant use of public assistance and mode of entry: Demographics versus dependence. Soc Sci Res. 2015 Sep;53:1-18. Epub 2015 May 5. https://doi.org/10.1016/j.ssresearch.2015.04.007
588. Nørredam M. Migration and health: exploring the role of migrant status through register-based studies. Dan Med J. 2015 Apr;62(4):B5068.
589. Augusterfer EF, Mollica RF, Lavelle J. A review of telemental health in international and post-disaster settings. Int Rev Psychiatry. 2015;27(6):540-6. Epub 2015 Nov 17. https://doi.org/10.3109/09540261.2015.1082985
590. Morton MJ, DeAugustinis ML, Velasquez CA, Singh S, Kelen GD. Developments in Surge Research Priorities: A Systematic Review of the Literature Following the Academic Emergency Medicine Consensus Conference, 2007-2015. Acad Emerg Med. 2015 Nov;22(11):1235-52. Epub 2015 Nov 3. https://doi.org/10.1111/acem.12815

591. Durham J, Brolan CE, Lui CW, Whittaker M. The need for a rights-based public health approach to Australian asylum seeker health. Public Health Rev. 2016 Aug 22;37:6. eCollection 2016. https://doi.org/10.1186/s40985-016-0020-9
592. Daynes L. The health impacts of the refugee crisis: a medical charity perspective. Clin Med (Lond). 2016 Oct;16(5):437-440. https://doi.org/10.7861/clinmedicine.16-5-437
593. Gardemann J, Wilp T. [The Humanitarian Charter and minimum standards in humanitarian response are applicable in German refugee facilities]. Bundesgesundheitsblatt Gesundheitsforschung Gesundheitsschutz. 2016 May;59(5):556-60. https://doi.org/10.1007/s00103-016-2330-y
594. Hudson CC, Adams S, Lauderdale J. Cultural Expressions of Intergenerational Trauma and Mental Health Nursing Implications for U.S. Health Care Delivery Following Refugee Resettlement: An Integrative Review of the Literature. J Transcult Nurs. 2016 May;27(3):286-301. Epub 2015 May 25. https://doi.org/10.1177/1043659615587591
595. Kazerooni Y, Gyedu A, Burnham G, Nwomeh B, Charles A, Mishra B, Kuah SS, Kushner AL, Stewart BT. Fires in refugee and displaced persons settlements: The current situation and opportunities to improve fire prevention and control. Burns. 2016 Aug;42(5):1036-1046. Epub 2016 Jan 23. https://doi.org/10.1016/j.burns.2015.11.008
596. Hanza MM, Goodson M, Osman A, Porraz Capetillo MD, Hared A, Nigon JA, Meiers SJ, Weis JA, Wieland ML, Sia IG. Lessons Learned from Community-Led Recruitment of Immigrants and Refugee Participants for a Randomized, Community-Based Participatory Research Study. J Immigr Minor Health. 2016 Oct;18(5):1241-1245. https://doi.org/10.1007/s10903-016-0394-2
597. Kos V. To Accept or Refuse Patient's Gift in Money? and How? - Case Report with Review. Pril (Makedon Akad Nauk Umet Odd Med Nauki). 2016 Nov 1;37(2-3):127-131. https://doi.org/10.1515/prilozi-2016-0026
598. Huot S, Kelly E, Park SJ. Occupational experiences of forced migrants: A scoping review. Aust Occup Ther J. 2016 Jun;63(3):186-205. Epub 2016 May 2. https://doi.org/10.1111/1440-1630.12261
599. Wieland ML, Weis JA, Hanza MM, Meiers SJ, Patten CA, Clark MM, Sloan JA, Novotny PJ, Njeru JW, Abbenyi A, Levine JA, Goodson M, Porraz Capetillo MG, Osman A, Hared A, Nigon JA, Sia IG. Healthy immigrant families: Participatory development and baseline characteristics of a community-based physical activity and nutrition intervention. Contemp Clin Trials. 2016 Mar;47:22-31. Epub 2015 Dec 4. https://doi.org/10.1016/j.cct.2015.12.004
600. Rauscher C, Salzberger B. [Initial examination and screening of migrants : What makes sense and what is evidence-based?]. Internist (Berl). 2016 May;57(5):452-6. https://doi.org/10.1007/s00108-016-0056-4
601. Bazerghi C, McKay FH, Dunn M. The Role of Food Banks in Addressing Food Insecurity: A Systematic Review. J Community Health. 2016 Aug;41(4):732-40. https://doi.org/10.1007/s10900-015-0147-5
602. Salti N, Ghattas H. Food insufficiency and food insecurity as risk factors for physical disability among Palestinian refugees in Lebanon: Evidence from an observational study. Disabil Health J. 2016 Oct;9(4):655-62. Epub 2016 Mar 25. https://doi.org/10.1016/j.dhjo.2016.03.003
603. Falb KL, Tanner S, Ward L, Erksine D, Noble E, Assazenew A, Bakomere T, Graybill E, Lowry C, Mallinga P, Neiman A, Poulton C, Robinette K, Sommer M, Stark L. Creating opportunities through mentorship, parental involvement, and safe spaces (COMPASS) program: multi-country study protocol to protect girls from violence in humanitarian

settings. BMC Public Health. 2016 Mar 5;16:231. https://doi.org/10.1186/s12889-016-2894-3
604. Spiegel PB. The humanitarian system is not just broke, but broken: recommendations for future humanitarian action. Lancet. 2017 Jun 7:S0140-6736(17)31278-3. Online ahead of print. https://doi.org/10.1016/S0140-6736(17)31278-3
605. Milton AH, Rahman M, Hussain S, Jindal C, Choudhury S, Akter S, Ferdousi S, Mouly TA, Hall J, Efird JT. Trapped in Statelessness: Rohingya Refugees in Bangladesh. Int J Environ Res Public Health. 2017 Aug 21;14(8):942. https://doi.org/10.3390/ijerph14080942
606. Merry L, Pelaez S, Edwards NC. Refugees, asylum-seekers and undocumented migrants and the experience of parenthood: a synthesis of the qualitative literature. Global Health. 2017 Sep 19;13(1):75. https://doi.org/10.1186/s12992-017-0299-4
607. Hoare T, Vidgen A, Roberts N. In their own words: a synthesis of the qualitative research on the experiences of adults seeking asylum. A systematic review of qualitative findings in forced migration. Med Confl Surviv. 2017 Dec;33(4):273-298. https://doi.org/10.1080/13623699.2017.1419902
608. Dowling A, Enticott J, Russell G. Measuring self-rated health status among resettled adult refugee populations to inform practice and policy - a scoping review. BMC Health Serv Res. 2017 Dec 8;17(1):817. https://doi.org/10.1186/s12913-017-2771-5
609. Robertshaw L, Dhesi S, Jones LL. Challenges and facilitators for health professionals providing primary healthcare for refugees and asylum seekers in high-income countries: a systematic review and thematic synthesis of qualitative research. BMJ Open. 2017 Aug 4;7(8):e015981. https://doi.org/10.1136/bmjopen-2017-015981
610. Hong MK, Varghese RE, Jindal C, Efird JT. Refugee Policy Implications of U.S. Immigration Medical Screenings: A New Era of Inadmissibility on Health-Related Grounds. Int J Environ Res Public Health. 2017 Sep 24;14(10):1107. https://doi.org/10.3390/ijerph14101107
611. Nyoka R, Foote AM, Woods E, Lokey H, O'Reilly CE, Magumba F, Okello P, Mintz ED, Marano N, Morris JF. Sanitation practices and perceptions in Kakuma refugee camp, Kenya: Comparing the status quo with a novel service-based approach. PLoS One. 2017 Jul 13;12(7):e0180864. eCollection 2017. https://doi.org/10.1371/journal.pone.0180864
612. Herath JC, Pollanen MS. Clinical Examination and Reporting of a Victim of Torture. Acad Forensic Pathol. 2017 Sep;7(3):330-339. Epub 2017 Sep 1. https://doi.org/10.23907/2017.030
613. National Academies of Sciences, Engineering, and Medicine; Health and Medicine Division; Board on Population Health and Public Health Practice; Roundtable on Health Literacy. Facilitating Health Communication with Immigrant, Refugee, and Migrant Populations Through the Use of Health Literacy and Community Engagement Strategies: Proceedings of a Workshop. Washington (DC): National Academies Press (US); 2017 Nov 9.
614. Block W, Lee J, Vijayasingham K. Mercy for money: Torture's link to profit in Sri Lanka, a retrospective review. Torture. 2017;27(1):28-41. https://doi.org/10.7146/torture.v27i1.26532
615. Silberholz EA, Brodie N, Spector ND, Pattishall AE. Disparities in access to care in marginalized populations. Curr Opin Pediatr. 2017 Dec;29(6):718-727. https://doi.org/10.1097/MOP.0000000000000549
616. Killedar A, Harris P. Australia's refugee policies and their health impact: a review of the evidence and recommendations for the Australian Government. Aust N Z J Public Health. 2017 Aug;41(4):335-337. Epub 2017 Mar 28. https://doi.org/10.1111/1753-6405.12663

617. Schilling T, Rauscher S, Menzel C, Reichenauer S, Müller-Schilling M, Schmid S, Selgrad M. Migrants and Refugees in Europe: Challenges, Experiences and Contributions. Visc Med. 2017 Aug;33(4):295-300. Epub 2017 Jul 19. https://doi.org/10.1159/000478763
618. Health and Medicine Division; National Academies of Sciences, Engineering, and Medicine. Health Communication with Immigrants, Refugees, and Migrant Workers: Proceedings of a Workshop—in Brief. Washington (DC): National Academies Press (US); 2017 Jun 2.
619. Carrara VI, Stuetz W, Lee SJ, Sriprawat K, Po B, Hanboonkunupakarn B, Nosten FH, McGready R. Longer exposure to a new refugee food ration is associated with reduced prevalence of small for gestational age: results from 2 cross-sectional surveys on the Thailand-Myanmar border. Am J Clin Nutr. 2017 Jun;105(6):1382-1390. Epub 2017 May 10. https://doi.org/10.3945/ajcn.116.148262
620. Goodkind JR, Amer S, Christian C, Hess JM, Bybee D, Isakson BL, Baca B, Ndayisenga M, Greene RN, Shantzek C. Challenges and Innovations in a Community-Based Participatory Randomized Controlled Trial. Health Educ Behav. 2017 Feb;44(1):123-130. Epub 2016 Jul 10. https://doi.org/10.1177/1090198116639243
621. Febles C, Nies MA, Fanning K, Tavernier SS. Challenges and Strategies in Providing Home Based Primary Care for Refugees in the US. J Immigr Minor Health. 2017 Dec;19(6):1498-1505. https://doi.org/10.1007/s10903-016-0481-4
622. Mishori R, Aleinikoff S, Davis D. Primary Care for Refugees: Challenges and Opportunities. Am Fam Physician. 2017 Jul 15;96(2):112-120.
623. Greer SL, Wismar M, Pastorino G, Kosinska M, editors. Civil society and health: Contributions and potential. Copenhagen (Denmark): European Observatory on Health Systems and Policies; 2017.
624. Mahmood SS, Wroe E, Fuller A, Leaning J. The Rohingya people of Myanmar: health, human rights, and identity. Lancet. 2017 May 6;389(10081):1841-1850. Epub 2016 Dec 2. https://doi.org/10.1016/S0140-6736(16)00646-2
625. Shortall CK, Glazik R, Sornum A, Pritchard C. On the ferries: the unmet health care needs of transiting refugees in Greece. Int Health. 2017 Sep 1;9(5):272-280. https://doi.org/10.1093/inthealth/ihx032
626. Willen SS, Knipper M, Abadía-Barrero CE, Davidovitch N. Syndemic vulnerability and the right to health. Lancet. 2017 Mar 4;389(10072):964-977. https://doi.org/10.1016/S0140-6736(17)30261-1
627. Patel R, King J, Phelps L, Sanderson D. Discussion Informed by Recurrent Lessons from a Systematic Review on Targeting Practices in Urban Humanitarian Crises. PLoS Curr. 2017 Oct 13;9:ecurrents.dis.0d0be4b294b40f5e51ee7b58d9687ea0. https://doi.org/10.1371/currents.dis.0d0be4b294b40f5e51ee7b58d9687ea0
628. Hansoti B, Aluisio AR, Barry MA, Davey K, Lentz BA, Modi P, Newberry JA, Patel MH, Smith TA, Vinograd AM, Levine AC; Global Emergency Medicine Think Tank Clinical Research Working Group. Global Health and Emergency Care: Defining Clinical Research Priorities. Acad Emerg Med. 2017 Jun;24(6):742-753. Epub 2017 Mar 17. https://doi.org/10.1111/acem.13158
629. Ziersch A, Due C. A mixed methods systematic review of studies examining the relationship between housing and health for people from refugee and asylum seeking backgrounds. Soc Sci Med. 2018 Sep;213:199-219. Epub 2018 Jul 27. https://doi.org/10.1016/j.socscimed.2018.07.045
630. Antonipillai V, Baumann A, Hunter A, Wahoush O, O'Shea T. Health Inequity and "Restoring Fairness" Through the Canadian Refugee Health Policy Reforms: A Literature

Review. J Immigr Minor Health. 2018 Feb;20(1):203-213. https://doi.org/10.1007/s10903-016-0486-z
631. Bartelson AR, Sutherland MA. Experiences of Trauma and Implications for Nurses Caring for Undocumented Immigrant Women and Refugee Women. Nurs Womens Health. 2018 Oct;22(5):411-416. Epub 2018 Aug 23. https://doi.org/10.1016/j.nwh.2018.07.003
632. Abbas M, Aloudat T, Bartolomei J, Carballo M, Durieux-Paillard S, Gabus L, Jablonka A, Jackson Y, Kaojaroen K, Koch D, Martinez E, Mendelson M, Petrova-Benedict R, Tsiodras S, Christie D, Saam M, Hargreaves S, Pittet D. Migrant and refugee populations: a public health and policy perspective on a continuing global crisis. Antimicrob Resist Infect Control. 2018 Sep 20;7:113. eCollection 2018. https://doi.org/10.1186/s13756-018-0403-4
633. Gulati G, Kelly BD, Meagher D, Kennedy H, Dunne CP. Hunger strikes in prisons: a narrative systematic review of ethical considerations from a physician's perspective. Ir J Psychol Med. 2018 Jun;35(2):135-142. https://doi.org/10.1017/ipm.2017.33
634. O'Higgins A, Ott EM, Shea MW. What is the Impact of Placement Type on Educational and Health Outcomes of Unaccompanied Refugee Minors? A Systematic Review of the Evidence. Clin Child Fam Psychol Rev. 2018 Sep;21(3):354-365. https://doi.org/10.1007/s10567-018-0256-7
635. Kotsiou OS, Kotsios P, Srivastava DS, Kotsios V, Gourgoulianis KI, Exadaktylos AK. Impact of the Refugee Crisis on the Greek Healthcare System: A Long Road to Ithaca. Int J Environ Res Public Health. 2018 Aug 20;15(8):1790. https://doi.org/10.3390/ijerph15081790
636. Mirza MQ, Harrison EA, Chang HC, Salo CD, Birman D. Community perspectives on substance use among Bhutanese and Iraqi refugees resettled in the United States. J Prev Interv Community. 2018 Jan-Mar;46(1):43-60. https://doi.org/10.1080/10852352.2018.1385956
637. Bhayana A, Bhayana B. Approach to developmental disabilities in newcomer families. Can Fam Physician. 2018 Aug;64(8):567-573.
638. Lawlis T, Islam W, Upton P. Achieving the four dimensions of food security for resettled refugees in Australia: A systematic review. Nutr Diet. 2018 Apr;75(2):182-192. Epub 2017 Dec 20. https://doi.org/10.1111/1747-0080.12402
639. Islam MM, Nuzhath T. Health risks of Rohingya refugee population in Bangladesh: a call for global attention. J Glob Health. 2018 Dec;8(2):020309. https://doi.org/10.7189/jogh.08.020309
640. Sweileh WM. Global research output in the health of international Arab migrants (1988-2017). BMC Public Health. 2018 Jun 18;18(1):755. https://doi.org/10.1186/s12889-018-5690-4
641. Wilhelm TJ, Post S. [Globalization: challenges in abdominal surgery for migrants and refugees]. Chirurg. 2018 Mar;89(3):197-204. https://doi.org/10.1007/s00104-017-0584-z
642. El Arab R, Sagbakken M. Healthcare services for Syrian refugees in Jordan: a systematic review. Eur J Public Health. 2018 Dec 1;28(6):1079-1087. https://doi.org/10.1093/eurpub/cky103
643. Wali N, Chen W, Rawal LB, Amanullah ASM, Renzaho AMN. Integrating human rights approaches into public health practices and policies to address health needs amongst Rohingya refugees in Bangladesh: a systematic review and meta-ethnographic analysis. Arch Public Health. 2018 Oct 11;76:59. eCollection 2018. https://doi.org/10.1186/s13690-018-0305-1

644. Dator W, Abunab H, Dao-Ayen N. Health challenges and access to health care among Syrian refugees in Jordan: a review. East Mediterr Health J. 2018 Sep 6;24(7):680-686. https://doi.org/10.26719/2018.24.7.680
645. Wieland ML, Hanza MMM, Weis JA, Meiers SJ, Patten CA, Clark MM, Sloan JA, Novotny PJ, Njeru JW, Abbenyi A, Levine JA, Goodson M, Capetillo GDP, Osman A, Hared A, Nigon JA, Sia IG. Healthy Immigrant Families: Randomized Controlled Trial of a Family-Based Nutrition and Physical Activity Intervention. Am J Health Promot. 2018 Feb;32(2):473-484. Epub 2017 Nov 29. https://doi.org/10.1177/0890117117733342
646. Ng Chok H, Mannix J, Dickson C, Wilkes L. Experiences of registered nurses from a refugee background: A scoping review. J Clin Nurs. 2018 Apr;27(7-8):e1275-e1283. Epub 2018 Feb 11. https://doi.org/10.1111/jocn.14257
647. Robards F, Kang M, Usherwood T, Sanci L. How Marginalized Young People Access, Engage With, and Navigate Health-Care Systems in the Digital Age: Systematic Review. J Adolesc Health. 2018 Apr;62(4):365-381. Epub 2018 Feb 21. https://doi.org/10.1016/j.jadohealth.2017.10.018
648. Tribe RH, Sendt KV, Tracy DK. A systematic review of psychosocial interventions for adult refugees and asylum seekers. J Ment Health. 2019 Dec;28(6):662-676. Epub 2017 May 9. https://doi.org/10.1080/09638237.2017.1322182
649. Ongenaert D, Joye S. Selling displaced people? A multi-method study of the public communication strategies of international refugee organisations. Disasters. 2019 Jul;43(3):478-508. Epub 2019 May 9. https://doi.org/10.1111/disa.12353
650. Kavukcu N, Altıntaş KH. The Challenges of the Health Care Providers in Refugee Settings: A Systematic Review. Prehosp Disaster Med. 2019 Apr;34(2):188-196. Epub 2019 Apr 10. https://doi.org/10.1017/S1049023X19000190
651. Peterson P, Ali S, Kenneh A, Wakefield A. Community engagement with refugee-background communities around health: the experience of the Group of 11. Aust J Prim Health. 2019 Apr;25(2):113-117. https://doi.org/10.1071/PY18139
652. Spaaij R, Broerse J, Oxford S, Luguetti C, McLachlan F, McDonald B, Klepac B, Lymbery L, Bishara J, Pankowiak A. Sport, Refugees, and Forced Migration: A Critical Review of the Literature. Front Sports Act Living. 2019 Oct 11;1:47. eCollection 2019. https://doi.org/10.3389/fspor.2019.00047
653. Blundell H, Milligan R, Norris SL, Garner P. WHO guidance for refugees in camps: systematic review. BMJ Open. 2019 Sep 4;9(9):e027094. https://doi.org/10.1136/bmjopen-2018-027094
654. Haque MS. Sustainable use of plastic brick from waste PET plastic bottle as building block in Rohingya refugee camp: a review. Environ Sci Pollut Res Int. 2019 Dec;26(36):36163-36183. Epub 2019 Nov 26. https://doi.org/10.1007/s11356-019-06843-y
655. Bozorgmehr K, Biddle L, Rohleder S, Puthoopparambil SJ, Jahn R. What is the evidence on availability and integration of refugee and migrant health data in health information systems in the WHO European Region? Themed issues on migration and health, X. Copenhagen: WHO Regional Office for Europe; 2019.
656. Kim J, ElRayes W, Anthony RS, Dombrowski K, Watanabe-Galloway S. A Review of Health Survey Research for People with Refugee Background Resettled from Africa: Research Gaps and Methodological Issues. J Racial Ethn Health Disparities. 2019 Feb;6(1):160-181. Epub 2018 Jul 16. https://doi.org/10.1007/s40615-018-0511-0

657. Guess MA, Tanabe KO, Nelson AE, Nguyen S, Hauck FR, Scharf RJ. Emergency Department and Primary Care Use by Refugees Compared to Non-refugee Controls. J Immigr Minor Health. 2019 Aug;21(4):793-800. https://doi.org/10.1007/s10903-018-0795-5
658. Russell G, Gunatillaka N, Lewis V, Cheng IH, Enticott J, Marsh G, Vasi S, Advocat J, Song H, Saito S, Casey S, Smith M, Harris M. The OPTIMISE project: protocol for a mixed methods, pragmatic, quasi-experimental trial to improve primary care delivery to refugees in Australia. BMC Health Serv Res. 2019 Jun 19;19(1):396. https://doi.org/10.1186/s12913-019-4235-6
659. El Hechi MW, Khalifeh JM, Ramly EP, Elahad JA, Bonde A, Velmahos GC, Hoballah JJ, Kaafarani HMA. Refugee Access to Surgical Care in Lebanon: A Post Hoc Analysis of the SCAR Study. J Surg Res. 2019 Aug;240:175-181. Epub 2019 Apr 4. https://doi.org/10.1016/j.jss.2019.03.007
660. Toubøl J. From democratic participation to civic resistance: the loss of institutional trust as an outcome of activism in the refugee solidarity movement. Br J Sociol. 2019 Sep;70(4):1198-1224. Epub 2018 Dec 12. https://doi.org/10.1111/1468-4446.12622
661. Clarke SK, Jaffe J, Mutch R. Overcoming Communication Barriers in Refugee Health Care. Pediatr Clin North Am. 2019 Jun;66(3):669-686. https://doi.org/10.1016/j.pcl.2019.02.012
662. National Academies of Sciences, Engineering, and Medicine; Division of Behavioral and Social Sciences and Education; Committee on Population; Majmundar MK, Olson S, editors. Forced Migration Research: From Theory to Practice in Promoting Migrant Well-Being: Proceedings of a Workshop. Washington (DC): National Academies Press (US); 2019 Dec 10.
663. Assi R, Özger-İlhan S, İlhan MN. Health needs and access to health care: the case of Syrian refugees in Turkey. Public Health. 2019 Jul;172:146-152. Epub 2019 Jun 22. https://doi.org/10.1016/j.puhe.2019.05.004
664. Au M, Anandakumar AD, Preston R, Ray RA, Davis M. A model explaining refugee experiences of the Australian healthcare system: a systematic review of refugee perceptions. BMC Int Health Hum Rights. 2019 Jul 18;19(1):22. https://doi.org/10.1186/s12914-019-0206-6
665. El Arnaout N, Rutherford S, Zreik T, Nabulsi D, Yassin N, Saleh S. Assessment of the health needs of Syrian refugees in Lebanon and Syria's neighboring countries. Confl Health. 2019 Jun 27;13:31. eCollection 2019. https://doi.org/10.1186/s13031-019-0211-3
666. Hahn K, Steinhäuser J, Wilfling D, Goetz K. Quality of health care for refugees - a systematic review. BMC Int Health Hum Rights. 2019 Jun 13;19(1):20. https://doi.org/10.1186/s12914-019-0205-7
667. McAuley R. Clinical Tools for Working Abroad with Migrants. Pediatr Clin North Am. 2019 Jun;66(3):589-599. https://doi.org/10.1016/j.pcl.2019.02.007
668. Ho S, Javadi D, Causevic S, Langlois EV, Friberg P, Tomson G. Intersectoral and integrated approaches in achieving the right to health for refugees on resettlement: a scoping review. BMJ Open. 2019 Jul 1;9(7):e029407. https://doi.org/10.1136/bmjopen-2019-029407
669. Brandenberger J, Tylleskär T, Sontag K, Peterhans B, Ritz N. A systematic literature review of reported challenges in health care delivery to migrants and refugees in high-income countries - the 3C model. BMC Public Health. 2019 Jun 14;19(1):755. https://doi.org/10.1186/s12889-019-7049-x
670. Borsch AS, de Montgomery CJ, Gauffin K, Eide K, Heikkilä E, Smith Jervelund S. Health, Education and Employment Outcomes in Young Refugees in the Nordic Countries: A

Systematic Review. Scand J Public Health. 2019 Nov;47(7):735-747. Epub 2018 Aug 1. https://doi.org/10.1177/1403494818787099
671. Ardic A, Esin MN, Koc S, Bayraktar B, Sunal N. Using the Omaha System to determine health problems of urban Syrian immigrants. Public Health Nurs. 2019 Mar;36(2):126-133. Epub 2018 Nov 22. https://doi.org/10.1111/phn.12563
672. Grech H. Impact of Forced Migration on Communication and Social Adaptation. Folia Phoniatr Logop. 2019;71(4):137-145. Epub 2019 Apr 4. https://doi.org/10.1159/000497057
673. Alawa J, Zarei P, Khoshnood K. Evaluating the Provision of Health Services and Barriers to Treatment for Chronic Diseases among Syrian Refugees in Turkey: A Review of Literature and Stakeholder Interviews. Int J Environ Res Public Health. 2019 Jul 25;16(15):2660. https://doi.org/10.3390/ijerph16152660
674. Agbata EN, Padilla PF, Agbata IN, Armas LH, Solà I, Pottie K, Alonso-Coello P. Migrant Healthcare Guidelines: A Systematic Quality Assessment. J Immigr Minor Health. 2019 Apr;21(2):401-413. https://doi.org/10.1007/s10903-018-0759-9
675. El Arab R, Sagbakken M. Child marriage of female Syrian refugees in Jordan and Lebanon: a literature review. Glob Health Action. 2019;12(1):1585709. https://doi.org/10.1080/16549716.2019.1585709
676. Fry MW, Skinner AC, Wheeler SB. Understanding the Relationship Between Male Gender Socialization and Gender-Based Violence Among Refugees in Sub-Saharan Africa. Trauma Violence Abuse. 2019 Dec;20(5):638-652. Epub 2017 Aug 29. https://doi.org/10.1177/1524838017727009
677. Nöst S, Jahn R, Aluttis F, Drepper J, Preussler S, Qreini M, Breckenkamp J, Razum O, Bozorgmehr K. [Health and primary care surveillance among asylum seekers in reception centres in Germany: concept, development, and implementation]. Bundesgesundheitsblatt Gesundheitsforschung Gesundheitsschutz. 2019 Jul;62(7):881-892. https://doi.org/10.1007/s00103-019-02971-5
678. Iolascon A, De Franceschi L, Muckenthaler M, Taher A, Rees D, de Montalembert M, Rivella S, Eleftheriou A, Cappellini MD. EHA Research Roadmap on Hemoglobinopathies and Thalassemia: An Update. Hemasphere. 2019 Jun 4;3(3):e208. eCollection 2019 Jun. https://doi.org/10.1097/HS9.0000000000000208
679. Khan-Gökkaya S, Higgen S, Mösko M. Qualification programmes for immigrant health professionals: A systematic review. PLoS One. 2019 Nov 15;14(11):e0224933. eCollection 2019. https://doi.org/10.1371/journal.pone.0224933
680. Villarroel N, Hannigan A, Severoni S, Puthoopparambil S, MacFarlane A. Migrant health research in the Republic of Ireland: a scoping review. BMC Public Health. 2019 Mar 20;19(1):324. https://doi.org/10.1186/s12889-019-6651-2
681. Guerra JVV, Alves VH, Rachedi L, Pereira AV, Branco MBLR, Santos MVD, Schveitzer MC, Carvalho BF. Forced international migration for refugee food: a scoping review. Cien Saude Colet. 2019 Dec;24(12):4499-4508. Epub 2019 Aug 12. https://doi.org/10.1590/1413-812320182412.23382019
682. Sharif MZ, Biegler K, Mollica R, Sim SE, Nicholas E, Chandler M, Ngo-Metzger Q, Paigne K, Paigne S, Sorkin DH. A Health Profile and Overview of Healthcare Experiences of Cambodian American Refugees and Immigrants Residing in Southern California. J Immigr Minor Health. 2019 Apr;21(2):346-355. https://doi.org/10.1007/s10903-018-0736-3
683. Bou-Karroum L, Daou KN, Nomier M, El Arnaout N, Fouad FM, El-Jardali F, Akl EA. Health Care Workers in the setting of the "Arab Spring": a scoping review for the Lancet-AUB

Commission on Syria. J Glob Health. 2019 Jun;9(1):010402. https://doi.org/10.7189/jogh.09.010402
684. Shackelford BB, Cronk R, Behnke N, Cooper B, Tu R, D'Souza M, Bartram J, Schweitzer R, Jaff D. Environmental health in forced displacement: A systematic scoping review of the emergency phase. Sci Total Environ. 2020 Apr 20;714:136553. Epub 2020 Jan 13. https://doi.org/10.1016/j.scitotenv.2020.136553
685. Fregoso RL. Stolen lives: What the dead teach us. Death Stud. 2020;44(11):736-745. Epub 2020 Jun 13. https://doi.org/10.1080/07481187.2020.1771856
686. Parajuli J, Horey D. Barriers to and facilitators of health services utilisation by refugees in resettlement countries: an overview of systematic reviews. Aust Health Rev. 2020 Feb;44(1):132-142. https://doi.org/10.1071/AH18108
687. Rashid M, Cervantes AD, Goez H. Refugee Health Curriculum in Undergraduate Medical Education (UME): A Scoping Review. Teach Learn Med. 2020 Oct-Dec;32(5):476-485. Epub 2020 Jun 19. https://doi.org/10.1080/10401334.2020.1779071
688. Synovec CE, Aceituno L. Social justice considerations for occupational therapy: The role of addressing social determinants of health in unstably housed populations. Work. 2020;65(2):235-246. https://doi.org/10.3233/WOR-203074
689. Zivot C, Dewey C, Heasley C, Srinivasan S, Little M. Exploring the State of Gender-Centered Health Research in the Context of Refugee Resettlement in Canada: A Scoping Review. Int J Environ Res Public Health. 2020 Oct 15;17(20):7511. https://doi.org/10.3390/ijerph17207511
690. Oltrogge JH, Schäfer I, Schlichting D, Jahnke M, Rakebrandt A, Pruskil S, Wagner HO, Lühmann D, Scherer M. Episodes of care in a primary care walk-in clinic at a refugee camp in Germany - a retrospective data analysis. BMC Fam Pract. 2020 Sep 21;21(1):193. https://doi.org/10.1186/s12875-020-01253-3
691. Suphanchaimat R, Sinam P, Phaiyarom M, Pudpong N, Julchoo S, Kunpeuk W, Thammawijaya P. A cross sectional study of unmet need for health services amongst urban refugees and asylum seekers in Thailand in comparison with Thai population, 2019. Int J Equity Health. 2020 Nov 11;19(1):205. https://doi.org/10.1186/s12939-020-01316-y
692. McElrone M, Colby S, Fouts HN, Spence M, Kavanagh K, Franzen-Castle L, Olfert MD, Kattelmann KK, White AA. Feasibility and Acceptability of Implementing a Culturally Adapted Cooking Curriculum for Burundian and Congolese Refugee Families. Ecol Food Nutr. 2020 Nov 1;59(6):598-614. Epub 2020 May 12. https://doi.org/10.1080/03670244.2020.1759575
693. O'Mara B, Carey G, Weier M. Community-based health promotion about alcohol and other drugs in a multicultural Australia-what works? A review of evidence. Health Educ Res. 2020 Oct 1;35(5):437-449. https://doi.org/10.1093/her/cyaa027
694. Hahn K, Steinhäuser J, Goetz K. Equity in Health Care: A Qualitative Study with Refugees, Health Care Professionals, and Administrators in One Region in Germany. Biomed Res Int. 2020 Feb 24;2020:4647389. eCollection 2020. https://doi.org/10.1155/2020/4647389
695. Narla NP, Surmeli A, Kivlehan SM. Agile Application of Digital Health Interventions during the COVID-19 Refugee Response. Ann Glob Health. 2020 Oct 15;86(1):135. https://doi.org/10.5334/aogh.2995
696. Brown CM, Swaminathan L, Saif NT, Hauck FR. Health Care for Refugee and Immigrant Adolescents. Prim Care. 2020 Jun;47(2):291-306. Epub 2020 Feb 20. https://doi.org/10.1016/j.pop.2020.02.007

697. Berthold SM, Polatin P, Mollica R, Higson-Smith C, Streets FJ, Kelly CM, Lavelle J. The complex care of a torture survivor in the United States: The case of Joshua. Torture. 2020;30(1):23-39. https://doi.org/10.7146/torture.v30i1.113063
698. Van Hout MC, Lungu-Byrne C, Germain J. Migrant health situation when detained in European immigration detention centres: a synthesis of extant qualitative literature. Int J Prison Health. 2020 Jun 1;16(3):221-236. https://doi.org/10.1108/IJPH-12-2019-0074
699. Radl-Karimi C, Nicolaisen A, Sodemann M, Batalden P, von Plessen C. Under what circumstances can immigrant patients and healthcare professionals co-produce health? - an interpretive scoping review. Int J Qual Stud Health Well-being. 2020 Dec;15(1):1838052. https://doi.org/10.1080/17482631.2020.1838052
700. Kostareva U, Albright CL, Berens EM, Levin-Zamir D, Aringazina A, Lopatina M, Ivanov LL, Sentell TL. International Perspective on Health Literacy and Health Equity: Factors That Influence the Former Soviet Union Immigrants. Int J Environ Res Public Health. 2020 Mar 24;17(6):2155. https://doi.org/10.3390/ijerph17062155
701. McGowan E, Beamish N, Stokes E, Lowe R. Core competencies for physiotherapists working with refugees: a scoping review. Physiotherapy. 2020 Sep;108:10-21. Epub 2020 Apr 30. https://doi.org/10.1016/j.physio.2020.04.004
702. Clancy M, Taylor J, Bradbury-Jones C, Phillimore J. A systematic review exploring palliative care for families who are forced migrants. J Adv Nurs. 2020 Nov;76(11):2872-2884. Epub 2020 Aug 31. https://doi.org/10.1111/jan.14509
703. Uygun E, Ilkkursun Z, Sijbrandij M, Aker AT, Bryant R, Cuijpers P, Fuhr DC, de Graaff AM, de Jong J, McDaid D, Morina N, Park AL, Roberts B, Ventevogel P, Yurtbakan T, Acarturk C; STRENGHTS consortium. Protocol for a randomized controlled trial: peer-to-peer Group Problem Management Plus (PM+) for adult Syrian refugees in Turkey. Trials. 2020 Mar 20;21(1):283. https://doi.org/10.1186/s13063-020-4166-x
704. Helmboldt L, Nikendei C, Zehetmair C, Schließler C, Ditzen B, Kohl RM. [Evaluation of the Use of Interpreters in Medical and Psychosocial Health Care of Refugees in a Reception and Registration Centre of the German Federal State of Baden-Wuerttemberg]. Psychother Psychosom Med Psychol. 2020 Oct;70(9-10):378-385. Epub 2020 Mar 11. https://doi.org/10.1055/a-1101-9626
705. Willcox-Pidgeon SM, Franklin RC, Leggat PA, Devine S. Identifying a gap in drowning prevention: high-risk populations. Inj Prev. 2020 Jun;26(3):279-288. Epub 2020 Jan 6. https://doi.org/10.1136/injuryprev-2019-043432
706. Mansour R, Liamputtong P, Arora A. Prevalence, Determinants, and Effects of Food Insecurity among Middle Eastern and North African Migrants and Refugees in High-Income Countries: A Systematic Review. Int J Environ Res Public Health. 2020 Oct 4;17(19):7262. https://doi.org/10.3390/ijerph17197262
707. Riza E, Kalkman S, Coritsidis A, Koubardas S, Vassiliu S, Lazarou D, Karnaki P, Zota D, Kantzanou M, Psaltopoulou T, Linos A. Community-Based Healthcare for Migrants and Refugees: A Scoping Literature Review of Best Practices. Healthcare (Basel). 2020 Apr 28;8(2):115. https://doi.org/10.3390/healthcare8020115
708. Bonfiglio G, Rosal K, Henao-Martínez A, Franco-Paredes C, Poeschla EM, Moo-Young J, Seefeldt T, Dunlevy H, Haas M, Young J. The long journey inside immigration detention centres in the USA. J Travel Med. 2020 Nov 9;27(7):taaa083. https://doi.org/10.1093/jtm/taaa083
709. Filler T, Jameel B, Gagliardi AR. Barriers and facilitators of patient centered care for immigrant and refugee women: a scoping review. BMC Public Health. 2020 Jun 26;20(1):1013. https://doi.org/10.1186/s12889-020-09159-6

710. Patel P, Bernays S, Dolan H, Muscat DM, Trevena L. Communication Experiences in Primary Healthcare with Refugees and Asylum Seekers: A Literature Review and Narrative Synthesis. Int J Environ Res Public Health. 2021 Feb 4;18(4):1469. https://doi.org/10.3390/ijerph18041469
711. Chadwick S, Townes DA, Perrone LA. Utility of Point of Care and Rapid Diagnostics in Humanitarian Emergencies. J Appl Lab Med. 2021 Jan 12;6(1):236-246. https://doi.org/10.1093/jalm/jfaa180
712. Filler T, Benipal PK, Torabi N, Minhas RS. A chair at the table: a scoping review of the participation of refugees in community-based participatory research in healthcare. Global Health. 2021 Sep 6;17(1):103. https://doi.org/10.1186/s12992-021-00756-7
713. Hawkins MM, Schmitt ME, Adebayo CT, Weitzel J, Olukotun O, Christensen AM, Ruiz AM, Gilman K, Quigley K, Dressel A, Mkandawire-Valhmu L. Promoting the health of refugee women: a scoping literature review incorporating the social ecological model. Int J Equity Health. 2021 Jan 23;20(1):45. https://doi.org/10.1186/s12939-021-01387-5
714. Faruque ASG, Khan AI, Islam SMR, Nahar B, Hossain MN, Widiati Y, Hasan ASMM, Prajapati M, Kim M, Vandenent M, Ahmed T. Diarrhea treatment center (DTC) based diarrheal disease surveillance in settlements in the wake of the mass influx of forcibly displaced Myanmar national (FDMN) in Cox's Bazar, Bangladesh, 2018. PLoS One. 2021 Aug 2;16(8):e0254473. eCollection 2021. https://doi.org/10.1371/journal.pone.0254473
715. Albers T, Ariccio S, Weiss LA, Dessi F, Bonaiuto M. The Role of Place Attachment in Promoting Refugees' Well-Being and Resettlement: A Literature Review. Int J Environ Res Public Health. 2021 Oct 20;18(21):11021. https://doi.org/10.3390/ijerph182111021
716. Young RA, Nelson MJ, Castellon RE, Martin CM. Improving quality in a complex primary care system-An example of refugee care and literature review. J Eval Clin Pract. 2021 Oct;27(5):1018-1026. Epub 2020 Jun 29. https://doi.org/10.1111/jep.13430
717. Cohen F, Yaeger L. Task-shifting for refugee mental health and psychosocial support: A scoping review of services in humanitarian settings through the lens of RE-AIM. Implement Res Pract. 2021 Mar 17;2:2633489521998790. eCollection 2021 Jan-Dec. https://doi.org/10.1177/2633489521998790
718. Zhang CX, Wurie F, Browne A, Haworth S, Burns R, Aldridge R, Zenner D, Tran A, Campos-Matos I. Social prescribing for migrants in the United Kingdom: A systematic review and call for evidence. J Migr Health. 2021 Oct 9;4:100067. eCollection 2021. https://doi.org/10.1016/j.jmh.2021.100067
719. Russell GM, Long K, Lewis V, Enticott JC, Gunatillaka N, Cheng IH, Marsh G, Vasi S, Advocat J, Saito S, Song H, Casey S, Smith M, Harris MF. OPTIMISE: a pragmatic stepped wedge cluster randomised trial of an intervention to improve primary care for refugees in Australia. Med J Aust. 2021 Nov 1;215(9):420-426. Epub 2021 Sep 28. https://doi.org/10.5694/mja2.51278
720. Warner G, Baghdasaryan Z, Osman F, Lampa E, Sarkadi A. 'I felt like a human being'-An exploratory, multi-method study of refugee involvement in the development of mental health intervention research. Health Expect. 2021 May;24 Suppl 1(Suppl 1):30-39. Epub 2019 Nov 9. https://doi.org/10.1111/hex.12990
721. Gagliardi J, Brettschneider C, König HH. Health-related quality of life of refugees: a systematic review of studies using the WHOQOL-Bref instrument in general and clinical refugee populations in the community setting. Confl Health. 2021 Jun 2;15(1):44. https://doi.org/10.1186/s13031-021-00378-1
722. Hiensch RJ. Pulmonary Manifestations of Torture. Chest. 2021 Jun;159(6):2384-2391. Epub 2021 Feb 12. https://doi.org/10.1016/j.chest.2021.02.010

723. Reese K, Moyer B. Refugee Medical Screening. Prim Care. 2021 Mar;48(1):9-21. Epub 2020 Nov 26. https://doi.org/10.1016/j.pop.2020.09.003
724. Karadag Caman O. Photovoice for Health Promotion Research, Empowerment, and Advocacy: Young Refugee Stories from Turkey. 2021 Mar 30. In: Corbin JH, Sanmartino M, Hennessy EA, Urke HB, editors. Arts and Health Promotion: Tools and Bridges for Practice, Research, and Social Transformation [Internet]. Cham (CH): Springer; 2021. Chapter 10.
725. Nur HA, Atoloye AT, Wengreen H, Archuleta M, Savoie-Roskos MR, Wille C, Jewkes M. A Scoping Review and Assessing the Evidence for Nutrition Education Delivery Strategies for Refugees in High-Income Countries. Adv Nutr. 2021 Dec 1;12(6):2508-2524. https://doi.org/10.1093/advances/nmab080
726. Saito S, Harris MF, Long KM, Lewis V, Casey S, Hogg W, Cheng IH, Advocat J, Marsh G, Gunatillaka N, Russell G. Response to language barriers with patients from refugee background in general practice in Australia: findings from the OPTIMISE study. BMC Health Serv Res. 2021 Sep 6;21(1):921. https://doi.org/10.1186/s12913-021-06884-5
727. Wachter K, Bunn M, Schuster RC, Boateng GO, Cameli K, Johnson-Agbakwu CE. A Scoping Review of Social Support Research among Refugees in Resettlement: Implications for Conceptual and Empirical Research. J Refug Stud. 2021 Jun 10;35(1):368-395. eCollection 2022 Mar. https://doi.org/10.1093/jrs/feab040
728. Modesti C, Talamo A. Defining Adjustment to Address the Missing Link between Refugees and Their Resettlement Communities. Int J Environ Res Public Health. 2021 Sep 20;18(18):9902. https://doi.org/10.3390/ijerph18189902
729. El-Khani A, Cartwright K, Maalouf W, Haar K, Zehra N, Çokamay-Yılmaz G, Calam R. Enhancing Teaching Recovery Techniques (TRT) with Parenting Skills: RCT of TRT + Parenting with Trauma-Affected Syrian Refugees in Lebanon Utilising Remote Training with Implications for Insecure Contexts and COVID-19. Int J Environ Res Public Health. 2021 Aug 16;18(16):8652. https://doi.org/10.3390/ijerph18168652
730. Wood JM, Booth AO, Margerison C, Worsley A. What factors are associated with food security among recently arrived refugees resettling in high-income countries? A scoping review. Public Health Nutr. 2021 Sep;24(13):4313-4327. Epub 2021 Jul 12. https://doi.org/10.1017/S1368980021002925
731. Arya N, Redditt VJ, Talavlikar R, Holland T, Brindamour M, Wright V, Saad A, Beukeboom C, Coakley A, Rashid M, Pottie K. Caring for refugees and newcomers in the post-COVID-19 era: Evidence review and guidance for FPs and health providers. Can Fam Physician. 2021 Aug;67(8):575-581. https://doi.org/10.46747/cfp.6708575
732. Johnson K, Carpenter E, Walters T. Special Issues in Immigrant Medicine. Prim Care. 2021 Mar;48(1):147-161. Epub 2020 Nov 26. https://doi.org/10.1016/j.pop.2020.09.010
733. Matlin SA, Karadag O, Brando CR, Góis P, Karabey S, Khan MMH, Saleh S, Takian A, Saso L. COVID-19: Marking the Gaps in Migrant and Refugee Health in Some Massive Migration Areas. Int J Environ Res Public Health. 2021 Nov 30;18(23):12639. https://doi.org/10.3390/ijerph182312639
734. Refugees and migrants in times of COVID-19: mapping trends of public health and migration policies and practices. Geneva: World Health Organization; 2021.
735. Lupieri S. Refugee Health During the Covid-19 Pandemic: A Review of Global Policy Responses. Risk Manag Healthc Policy. 2021 Apr 6;14:1373-1378. eCollection 2021. https://doi.org/10.2147/RMHP.S259680
736. Tan JS, Allen CW. Cultural Considerations in Caring for Refugees and Immigrants. Prim Care. 2021 Mar;48(1):35-43. Epub 2020 Nov 26. https://doi.org/10.1016/j.pop.2020.09.005

737. Liem A, Natari RB, Jimmy, Hall BJ. Digital Health Applications in Mental Health Care for Immigrants and Refugees: A Rapid Review. Telemed J E Health. 2021 Jan;27(1):3-16. Epub 2020 Jun 4. https://doi.org/10.1089/tmj.2020.0012
738. League A, Donato KM, Sheth N, Selden E, Patel S, Cooper LB, Mendenhall E. A Systematic Review of Medical-Legal Partnerships Serving Immigrant Communities in the United States. J Immigr Minor Health. 2021 Feb;23(1):163-174. Epub 2020 Sep 25. https://doi.org/10.1007/s10903-020-01088-1
739. Kostareva U, Albright CL, Berens EM, Polansky P, Kadish DE, Ivanov LL, Sentell TL. A Multilingual Integrative Review of Health Literacy in Former Soviet Union, Russian-Speaking Immigrants. Int J Environ Res Public Health. 2021 Jan 14;18(2):657. https://doi.org/10.3390/ijerph18020657
740. Bowsher G, El Achi N, Augustin K, Meagher K, Ekzayez A, Roberts B, Patel P. eHealth for service delivery in conflict: a narrative review of the application of eHealth technologies in contemporary conflict settings. Health Policy Plan. 2021 Jun 25;36(6):974-981. https://doi.org/10.1093/heapol/czab042
741. Bernardini S, Pieri M, Ciotti M. How Could POCT be a Useful Tool for Migrant and Refugee Health?. EJIFCC. 2021 Jun 29;32(2):200-208. eCollection 2021 Jun.
742. Brown CM, Bland S, Saif N. Effective Communication with Refugees and Immigrants. Prim Care. 2021 Mar;48(1):23-34. Epub 2020 Nov 26. https://doi.org/10.1016/j.pop.2020.09.004
743. Biswas B, Ahsan MN, Mallick B. Analysis of residential satisfaction: An empirical evidence from neighbouring communities of Rohingya camps in Cox's Bazar, Bangladesh. PLoS One. 2021 Apr 29;16(4):e0250838. eCollection 2021. https://doi.org/10.1371/journal.pone.0250838
744. Jaung MS, Willis R, Sharma P, Aebischer Perone S, Frederiksen S, Truppa C, Roberts B, Perel P, Blanchet K, Ansbro É. Models of care for patients with hypertension and diabetes in humanitarian crises: a systematic review. Health Policy Plan. 2021 May 17;36(4):509-532. https://doi.org/10.1093/heapol/czab007
745. Alduraidi H, Abdulla Aqel A, Saleh Z, Almansour I, Darawad M. UNRWA's role in promoting health outcomes of Palestinian refugees in Jordan: A systematic literature review. Public Health Nurs. 2021 Jul;38(4):692-700. Epub 2021 Mar 14. https://doi.org/10.1111/phn.12889
746. Chowdhury N, Naeem I, Ferdous M, Chowdhury M, Goopy S, Rumana N, Turin TC. Unmet Healthcare Needs Among Migrant Populations in Canada: Exploring the Research Landscape Through a Systematic Integrative Review. J Immigr Minor Health. 2021 Apr;23(2):353-372. Epub 2020 Sep 26. https://doi.org/10.1007/s10903-020-01086-3
747. Kaya H, Sengoren Dikis O, Sezgin B, Demirci H, Haberal MA, Akar E, Yüksel M. Evaluation of Syrian refugees' emergency service admissions in the western region of Turkey. Pathog Glob Health. 2021 May;115(3):196-202. Epub 2021 Jan 24. https://doi.org/10.1080/20477724.2021.1878442
748. Pham Q, Gamble A, Hearn J, Cafazzo JA. The Need for Ethnoracial Equity in Artificial Intelligence for Diabetes Management: Review and Recommendations. J Med Internet Res. 2021 Feb 10;23(2):e22320. https://doi.org/10.2196/22320
749. Jesus TS, Kamalakannan S, Bhattacharjya S, Bogdanova Y, Arango-Lasprilla JC, Bentley J, Landry MD, Papadimitriou C; Refugee Empowerment Task Force and International Networking Group of the American Congress of Rehabilitation Medicine. PREparedness, REsponse and SySTemic transformation (PRE-RE-SyST): a model for disability-inclusive pandemic responses and systemic disparities reduction derived from a scoping review and

thematic analysis. Int J Equity Health. 2021 Sep 14;20(1):204. https://doi.org/10.1186/s12939-021-01526-y
750. Woolhandler S, Himmelstein DU, Ahmed S, Bailey Z, Bassett MT, Bird M, Bor J, Bor D, Carrasquillo O, Chowkwanyun M, Dickman SL, Fisher S, Gaffney A, Galea S, Gottfried RN, Grumbach K, Guyatt G, Hansen H, Landrigan PJ, Lighty M, McKee M, McCormick D, McGregor A, Mirza R, Morris JE, Mukherjee JS, Nestle M, Prine L, Saadi A, Schiff D, Shapiro M, Tesema L, Venkataramani A. Public policy and health in the Trump era. Lancet. 2021 Feb 20;397(10275):705-753. Epub 2021 Feb 11. https://doi.org/10.1016/S0140-6736(20)32545-9
751. Echterhoff G, Becker JC, Knausenberger J, Hellmann JH. Helping in the context of refugee immigration. Curr Opin Psychol. 2022 Apr;44:106-111. Epub 2021 Sep 9. https://doi.org/10.1016/j.copsyc.2021.08.035
752. Gower S, Jeemi Z, Forbes D, Kebble P, Dantas JAR. Peer Mentoring Programs for Culturally and Linguistically Diverse Refugee and Migrant Women: An Integrative Review. Int J Environ Res Public Health. 2022 Oct 7;19(19):12845. https://doi.org/10.3390/ijerph191912845
753. Lieberman Lawry L. Review of Humanitarian Guidelines to Ensure the Health and Well-being of Afghan Refugees on U.S. Military Bases. Mil Med. 2022 Oct 29;187(11-12):1299-1309. https://doi.org/10.1093/milmed/usac086
754. Ebren G, Demircioğlu M, Çırakoğlu OC. A neglected aspect of refugee relief works: Secondary and vicarious traumatic stress. J Trauma Stress. 2022 Jun;35(3):891-900. Epub 2022 Feb 24. https://doi.org/10.1002/jts.22796
755. Fox S, Kramer E, Agrawal P, Aniyizhai A. Refugee and Migrant Health Literacy Interventions in High-Income Countries: A Systematic Review. J Immigr Minor Health. 2022 Feb;24(1):207-236. Epub 2021 Feb 25. https://doi.org/10.1007/s10903-021-01152-4
756. Gruner D, Feinberg Y, Venables MJ, Shanza Hashmi S, Saad A, Archibald D, Pottie K. An undergraduate medical education framework for refugee and migrant health: Curriculum development and conceptual approaches. BMC Med Educ. 2022 May 16;22(1):374. https://doi.org/10.1186/s12909-022-03413-8
757. Griffin G, Ali M, Nau SZ, Riggs E, Dantas JAR. Accessing and navigating healthcare: A scoping review of the experiences of women of refugee background from Myanmar. Health Soc Care Community. 2022 Nov;30(6):e3459-e3476. Epub 2022 Aug 1. https://doi.org/10.1111/hsc.13955
758. Anwar A, Mondal PK, Yadav UN, Shamim AA, Rizwan AAM, Mistry SK. Implications of updated protocol for classification of childhood malnutrition and service delivery in world's largest refugee camp amid this COVID-19 pandemic. Public Health Nutr. 2022 Mar;25(3):538-542. Epub 2022 Jan 12. https://doi.org/10.1017/S1368980022000052
759. El-Moslemany R, Mellon L, Tully L, McConkey SJ. Factors Associated With Intimate Partner Violence Perpetration and Victimization in Asylum Seeking and Refugee Populations: A Systematic Review. Trauma Violence Abuse. 2022 Jul;23(3):827-839. Epub 2020 Dec 11. https://doi.org/10.1177/1524838020977147
760. South E, Rodgers M, Wright K, Whitehead M, Sowden A. Reducing lifestyle risk behaviours in disadvantaged groups in high-income countries: A scoping review of systematic reviews. Prev Med. 2022 Jan;154:106916. Epub 2021 Dec 16. https://doi.org/10.1016/j.ypmed.2021.106916
761. Elreichouni A, Aly S, Maciejewski K, Salem I, Ghossein N, Mankash MS, Dziura J, Mowafi H. Health services access, utilization, and barriers for Arabic-speaking refugees

resettled in Connecticut, USA. BMC Health Serv Res. 2022 Nov 11;22(1):1337. https://doi.org/10.1186/s12913-022-08733-5
762. Daniels D, Imdad A, Buscemi-Kimmins T, Vitale D, Rani U, Darabaner E, Shaw A, Shaw J. Vaccine hesitancy in the refugee, immigrant, and migrant population in the United States: A systematic review and meta-analysis. Hum Vaccin Immunother. 2022 Nov 30;18(6):2131168. Epub 2022 Nov 4. https://doi.org/10.1080/21645515.2022.2131168
763. Martin DE, Fadhil RAS, Więcek A. Ethical Aspects of Kidney Donation and Transplantation for Migrants. Semin Nephrol. 2022 Jul;42(4):151271. Epub 2022 Dec 27. https://doi.org/10.1016/j.semnephrol.2022.07.005
764. Lee IS, Kim E. Effects of parenting education programs for refugee and migrant parents: a systematic review and meta-analysis. Child Health Nurs Res. 2022 Jan;28(1):23-40. Epub 2022 Jan 28. https://doi.org/10.4094/chnr.2022.28.1.23
765. Su Z, McDonnell D, Cheshmehzangi A, Ahmad J, Šegalo S, Pereira da Veiga C, Xiang YT. Public health crises and Ukrainian refugees. Brain Behav Immun. 2022 Jul;103:243-245. Epub 2022 May 10. https://doi.org/10.1016/j.bbi.2022.05.004
766. Yeh CY, Tsai CC. Massive Distance Education: Barriers and Challenges in Shifting to a Complete Online Learning Environment. Front Psychol. 2022 Jun 23;13:928717. eCollection 2022. https://doi.org/10.3389/fpsyg.2022.928717
767. Ankomah A, Byaruhanga J, Woolley E, Boamah S, Akombi-Inyang B. Double burden of malnutrition among migrants and refugees in developed countries: A mixed-methods systematic review. PLoS One. 2022 Aug 18;17(8):e0273382. eCollection 2022. https://doi.org/10.1371/journal.pone.0273382
768. P Iqbal M, Walpola R, Harris-Roxas B, Li J, Mears S, Hall J, Harrison R. Improving primary health care quality for refugees and asylum seekers: A systematic review of interventional approaches. Health Expect. 2022 Oct;25(5):2065-2094. Epub 2021 Oct 15. https://doi.org/10.1111/hex.13365
769. Rhodes-Dicker L, Brown NJ, Currell M. Unpacking intersecting complexities for WASH in challenging contexts: A review. Water Res. 2022 Feb 1;209:117909. Epub 2021 Nov 30. https://doi.org/10.1016/j.watres.2021.117909
770. Selvan K, Leekha A, Abdelmeguid H, Malvankar-Mehta MS. Barriers adult refugees face to community health and patient engagement: a systematic review. Glob Public Health. 2022 Dec;17(12):3412-3425. Epub 2022 Sep 8. https://doi.org/10.1080/17441692.2022.2121846
771. Sagi OI, Ohana N, Appel R, Kogan L. The Impact of the Syrian Civil War on One Department in an Israeli Hospital. Semin Plast Surg. 2022 Aug 4;36(2):107-112. eCollection 2022 May. https://doi.org/10.1055/s-0042-1749105
772. Deps PD, Aborghetti HP, Zambon TL, Costa VC, Dos Santos JD, Collin SM, Charlier P. Assessing signs of torture: A review of clinical forensic dermatology. J Am Acad Dermatol. 2022 Aug;87(2):375-380. Epub 2020 Sep 16. https://doi.org/10.1016/j.jaad.2020.09.031
773. Butenop J, Brake T, Mauder S, Razum O. [Health Situation in Ukraine Before Onset of War and Its Relevance for Health Care for Ukrainian Refugees in Germany: Literature Review, Risk Analysis, and Priority Setting]. Gesundheitswesen. 2022 Aug;84(8-09):679-688. Epub 2022 Jul 15. https://doi.org/10.1055/a-1876-2423
774. Kumar BN, James R, Hargreaves S, Bozorgmehr K, Mosca D, Hosseinalipour SM, AlDeen KN, Tatsi C, Mussa R, Veizis A, Kállayová D, Blanchet K, Machado RS, Orcutt M, Severoni S. Meeting the health needs of displaced people fleeing Ukraine: Drawing on existing technical guidance and evidence. Lancet Reg Health Eur. 2022 May 7;17:100403. eCollection 2022 Jun. https://doi.org/10.1016/j.lanepe.2022.100403

775. Morassaei S, Irvin E, Smith PM, Wilson K, Ghahari S. The Role of Immigrant Admission Classes on the Health and Well-being of Immigrants and Refugees in Canada: A Scoping Review. J Immigr Minor Health. 2022 Aug;24(4):1045-1060. Epub 2022 Mar 18. https://doi.org/10.1007/s10903-022-01352-6
776. Nowak AC, Namer Y, Hornberg C. Health Care for Refugees in Europe: A Scoping Review. Int J Environ Res Public Health. 2022 Jan 24;19(3):1278. https://doi.org/10.3390/ijerph19031278
777. Nisbet C, Lestrat KE, Vatanparast H. Food Security Interventions among Refugees around the Globe: A Scoping Review. Nutrients. 2022 Jan 25;14(3):522. https://doi.org/10.3390/nu14030522
778. Razavi SD, Noorulhuda M, Marcela Velez C, Kapiriri L, Dreyse BA, Danis M, Essue B, Goold SD, Nouvet E, Williams I. Priority setting for pandemic preparedness and response: A comparative analysis of COVID-19 pandemic plans in 12 countries in the Eastern Mediterranean Region. Health Policy Open. 2022 Dec;3:100084. Epub 2022 Nov 18. https://doi.org/10.1016/j.hpopen.2022.100084
779. Daniel M, Ottemöller FPG. Salutogenesis and Migration. 2022 Jan 1. In: Mittelmark MB, Bauer GF, Vaandrager L, Pelikan JM, Sagy S, Eriksson M, Lindström B, Meier Magistretti C, editors. The Handbook of Salutogenesis [Internet]. 2nd edition. Cham (CH): Springer; 2022. Chapter 47.
780. Matsangos M, Ziaka L, Exadaktylos AK, Klukowska-Rötzler J, Ziaka M. Health Status of Afghan Refugees in Europe: Policy and Practice Implications for an Optimised Healthcare. Int J Environ Res Public Health. 2022 Jul 27;19(15):9157. https://doi.org/10.3390/ijerph19159157
781. Scarr JP, Jagnoor J. Identifying opportunities for multisectoral action for drowning prevention: a scoping review. Inj Prev. 2022 Dec;28(6):585-594. Epub 2022 Oct 21. https://doi.org/10.1136/ip-2022-044712
782. Ishizuka A, Chiba M, Iso H, Katsuma Y. Unfinished business: Lessons for future G20 meetings on a more inclusive understanding of universal health coverage. GHM Open. 2022 Aug 31;2(1):1-11. https://doi.org/10.35772/ghmo.2021.01006
783. Lima Junior LP, Lima KCO, Bertolozzi MR, França FOS. Vulnerabilities of Arab refugees in primary health care: a scoping review. Rev Saude Publica. 2022 Apr 1;56:15. eCollection 2022. https://doi.org/10.11606/s1518-8787.2022056003691
784. Lindmark M, Cherukumilli K, Crider YS, Marcenac P, Lozier M, Voth-Gaeddert L, Lantagne DS, Mihelcic JR, Zhang QM, Just C, Pickering AJ. Passive In-Line Chlorination for Drinking Water Disinfection: A Critical Review. Environ Sci Technol. 2022 Jul 5;56(13):9164-9181. Epub 2022 Jun 14. https://doi.org/10.1021/acs.est.1c08580
785. Valeriani G, Sarajlic Vukovic I, Bersani FS, Sadeghzadeh Diman A, Ghorbani A, Mollica R. Tackling Ethnic Health Disparities Through Community Health Worker Programs: A Scoping Review on Their Utilization During the COVID-19 Outbreak. Popul Health Manag. 2022 Aug;25(4):517-526. Epub 2022 Apr 12. https://doi.org/10.1089/pop.2021.0364
786. Führer A. [Determinants of asylum seekers' health and medical care in Germany]. Bundesgesundheitsblatt Gesundheitsforschung Gesundheitsschutz. 2023 Oct;66(10):1083-1091. Epub 2023 Sep 14. https://doi.org/10.1007/s00103-023-03762-9
787. Davis MV, Ishiwata E, Sethi J, Sobral B. The Hows of Resident-Driven Community Empowerment toward Health Equity. Prog Community Health Partnersh. 2023;17(4):583-593.
788. Awuah WA, Adebusoye FT, Ferreira T, Azeem S, Bharadwaj HR, Akpan AA, Wellington J, Zia MR, Kumar H, Khalid A, Abdul-Rahman T, Isik A. The unmet surgical needs

of global refugee populations: A perspective review. SAGE Open Med. 2023 Oct 10;11:20503121231204492. eCollection 2023. https://doi.org/10.1177/20503121231204492
789. Ho CH, Denton AH, Blackstone SR, Saif N, MacIntyre K, Ozkaynak M, Valdez RS, Hauck FR. Access to Healthcare Among US Adult Refugees: A Systematic Qualitative Review. J Immigr Minor Health. 2023 Dec;25(6):1426-1462. Epub 2023 Apr 2. https://doi.org/10.1007/s10903-023-01477-2
790. MacPherson M. Immigrant, Refugee, and Indigenous Canadians' Experiences With Virtual Health Care Services: Rapid Review. JMIR Hum Factors. 2023 Oct 9;10:e47288. https://doi.org/10.2196/47288
791. Lokot M, Hartman E, Hashmi I. Participatory approaches and methods in gender equality and gender-based violence research with refugees and internally displaced populations: a scoping review. Confl Health. 2023 Dec 8;17(1):58. https://doi.org/10.1186/s13031-023-00554-5
792. Müller TR. Corporate sector engagement in contemporary 'crises': the case of refugee integration in Germany. Disasters. 2023 Oct;47(4):972-994. Epub 2023 May 18. https://doi.org/10.1111/disa.12583
793. Allen-Leap M, Hooker L, Wild K, Wilson IM, Pokharel B, Taft A. Seeking Help From Primary Health-Care Providers in High-Income Countries: A Scoping Review of the Experiences of Migrant and Refugee Survivors of Domestic Violence. Trauma Violence Abuse. 2023 Dec;24(5):3715-3731. Epub 2022 Dec 13. https://doi.org/10.1177/15248380221137664
794. Fraser HSF, Zahiri K, Kim N, Kim C, Craig S. The Global Health Informatics landscape and JAMIA. J Am Med Inform Assoc. 2023 Mar 16;30(4):775-780. https://doi.org/10.1093/jamia/ocad024
795. Georgeou N, Schismenos S, Wali N, Mackay K, Moraitakis E. A Scoping Review of Aging Experiences Among Culturally and Linguistically Diverse People in Australia: Toward Better Aging Policy and Cultural Well-Being for Migrant and Refugee Adults. Gerontologist. 2023 Jan 24;63(1):182-199. https://doi.org/10.1093/geront/gnab191
796. Yashadhana A, Alloun E, Serova N, de Leeuw E, Mengesha Z. Place-making and its impact on health and wellbeing among recently resettled refugees in high income contexts: A scoping review. Health Place. 2023 May;81:103003. Epub 2023 Mar 24. https://doi.org/10.1016/j.healthplace.2023.103003
797. Prusaczyk A, Bogdan M, Vinker S, Gujski M, Żuk P, Kowalska-Bobko I, Karczmarz S, Oberska J, Lewtak K. Health Care Organization in Poland in Light of the Refugee Crisis Related to the Military Conflict in Ukraine. Int J Environ Res Public Health. 2023 Feb 21;20(5):3831. https://doi.org/10.3390/ijerph20053831
798. Kouritzin T, Spence JC, Lee K. Food Intake and Food Selection Following Physical Relocation: A Scoping Review. Public Health Rev. 2023 Feb 1;44:1605516. eCollection 2023. https://doi.org/10.3389/phrs.2023.1605516
799. Filmer T, Ray R, Glass BD. Barriers and facilitators experienced by migrants and refugees when accessing pharmaceutical care: A scoping review. Res Social Adm Pharm. 2023 Jul;19(7):977-988. Epub 2023 Feb 27. https://doi.org/10.1016/j.sapharm.2023.02.016
800. Cohen F. Cultural idioms of distress among displaced populations: A scoping review. Int J Soc Psychiatry. 2023 Feb;69(1):5-13. Epub 2022 Aug 9. https://doi.org/10.1177/00207640221114742

801. Worabo HJ, Salt R, Grubesic R, Farokhi MR. Promoting social justice through experiential learning at an interprofessional refugee clinic. Nurse Educ Today. 2023 Feb;121:105699. Epub 2022 Dec 17. https://doi.org/10.1016/j.nedt.2022.105699
802. Bozorgmehr K, McKee M, Azzopardi-Muscat N, Bartovic J, Campos-Matos I, Gerganova TI, Hannigan A, Janković J, Kállayová D, Kaplan J, Kayi I, Kondilis E, Lundberg L, Mata I, Medarević A, Suvada J, Wickramage K, Puthoopparambil SJ. Integration of migrant and refugee data in health information systems in Europe: advancing evidence, policy and practice. Lancet Reg Health Eur. 2023 Oct 27;34:100744. eCollection 2023 Nov. https://doi.org/10.1016/j.lanepe.2023.100744
803. Zimba O, Gasparyan AY. Refugee Health: A Global and Multidisciplinary Challenge. J Korean Med Sci. 2023 Feb 13;38(6):e60. https://doi.org/10.3346/jkms.2023.38.e60
804. Brake TM, Dudek V, Sauzet O, Razum O. Psychosocial Attributes of Housing and Their Relationship With Health Among Refugee and Asylum-Seeking Populations in High-Income Countries: Systematic Review. Public Health Rev. 2023 May 4;44:1605602. eCollection 2023. https://doi.org/10.3389/phrs.2023.1605602
805. Cabieses B, Velázquez B, Blukacz A, Farante S, Bojórquez I, Mezones-Holguín E. Intersections between gender approaches, migration and health in Latin America and the Caribbean: a discussion based on a scoping review. Lancet Reg Health Am. 2023 Jun 20;40:100538. eCollection 2024 Dec. https://doi.org/10.1016/j.lana.2023.100538
806. Senthanar S, Dali N, Khan TH. A scoping review of refugees' employment integration experience and outcomes in Canada. Work. 2023;75(4):1165-1178. https://doi.org/10.3233/WOR-220221
807. Beqiraj G, Ferrari L. Taking Action towards an Inclusive Career Counselling for Asylum Seekers and Refugees-A Literature Review Based on the PRISMA Model. Behav Sci (Basel). 2023 Nov 22;13(12):962. https://doi.org/10.3390/bs13120962
808. Casale LM, Gentles SJ, McLaughlin J, Schneider M. Service access experiences of immigrant and refugee caregivers of autistic children in Canada: A scoping review. PLoS One. 2023 Nov 9;18(11):e0293656. eCollection 2023. https://doi.org/10.1371/journal.pone.0293656
809. Jankowski M, Lazarus JV, Kuchyn I, Zemskov S, Gałązkowski R, Gujski M. One Year On: Poland's Public Health Initiatives and National Response to Millions of Refugees from Ukraine. Med Sci Monit. 2023 Mar 31;29:e940223. https://doi.org/10.12659/MSM.940223
810. Tejkl L, Tellez D, McLaughlin D, Savold J, Vasquez C, Abrahim O, Spiegel P. Evaluation of the US detention standards to protect the health and dignity of migrants: a systematic review of national health standards. BMJ Open. 2023 Apr 18;13(4):e069949. https://doi.org/10.1136/bmjopen-2022-069949
811. Biesiada A, Mastalerz-Migas A, Babicki M. Response to provide key health services to Ukrainian refugees: The overview and implementation studies. Soc Sci Med. 2023 Oct;334:116221. Epub 2023 Sep 4. https://doi.org/10.1016/j.socscimed.2023.116221
812. Machado S, Karsiem S, Lavergne MR, Goldenberg S, Wiedmeyer ML. Respectful community engagement in health research with diverse im/migrant communities. BMJ Open. 2023 Dec 13;13(12):e077391. https://doi.org/10.1136/bmjopen-2023-077391
813. Bozorgmehr K, Kühne S, Biddle L. Local political climate and spill-over effects on refugee and migrant health: a conceptual framework and call to advance the evidence. BMJ Glob Health. 2023 Mar;8(3):e011472. https://doi.org/10.1136/bmjgh-2022-011472
814. Bang SH, Huang YC, Kuo HJ, Cho ES, García AA. Health status and Healthcare Access of Southeast Asian refugees in the United States: An integrative review. Public Health Nurs. 2023 Mar;40(2):324-337. Epub 2023 Jan 20. https://doi.org/10.1111/phn.13167

815. Whitehead L, Talevski J, Fatehi F, Beauchamp A. Barriers to and Facilitators of Digital Health Among Culturally and Linguistically Diverse Populations: Qualitative Systematic Review. J Med Internet Res. 2023 Feb 28;25:e42719. https://doi.org/10.2196/42719
816. Oladosu AO, Khai TS, Asaduzzaman M. Factors affecting access to healthcare for young people in the informal sector in developing countries: a systematic review. Front Public Health. 2023 Jun 8;11:1168577. eCollection 2023. https://doi.org/10.3389/fpubh.2023.1168577
817. Biddle L, Hintermeier M, Costa D, Wasko Z, Bozorgmehr K. Context, health and migration: a systematic review of natural experiments. EClinicalMedicine. 2023 Sep 18;64:102206. eCollection 2023 Oct. https://doi.org/10.1016/j.eclinm.2023.102206
818. Coumans JVF, Wark S. A scoping review on the barriers to and facilitators of health services utilisation related to refugee settlement in regional or rural areas of the host country. BMC Public Health. 2024 Jan 17;24(1):199. https://doi.org/10.1186/s12889-024-17694-9
819. Adebayo CT, Olukotun OV, Olukotun M, Kirungi J, Gondwe KW, Crooks NK, Singer RB, Adams S, Alfaifi FY, Dressel A, Fahmy L, Kako P, Snethen J, Mkandawire-Valhmu L. Experiences of gender-based violence among Somali refugee women: a socio-ecological model approach. Cult Health Sex. 2024 May;26(5):654-670. Epub 2023 Jul 30. https://doi.org/10.1080/13691058.2023.2236163
820. Essex R, Govintharjah P, Issa R, Kalocsányiová E, Lakika D, Markowski M, Smith J, Thompson T. Health Related Quality of life Amongst Refugees: A meta Analysis of Studies Using the SF-36. J Immigr Minor Health. 2024 Oct;26(5):925-935. Epub 2024 Jul 3. https://doi.org/10.1007/s10903-024-01615-4
821. Smith SE, Livingston P, Carney E, Mardon J, Tallentire VR. Snakes and ladders: An integrative literature review of refugee doctors' workforce integration needs. Med Educ. 2024 Jul;58(7):782-796. Epub 2023 Dec 16. https://doi.org/10.1111/medu.15290
822. Al-Hamad A, Yasin YM, Metersky K. Predictors, barriers, and facilitators to refugee women's employment and economic inclusion: A mixed methods systematic review. PLoS One. 2024 Jul 17;19(7):e0305463. eCollection 2024. https://doi.org/10.1371/journal.pone.0305463
823. Tafesse W, Jemutai J, Mayora C, Margini F. Scoping Review of Health Economics Research on Refugee Health in Sub-Saharan Africa. Value Health Reg Issues. 2024 Jan;39:98-106. Epub 2023 Dec 7. https://doi.org/10.1016/j.vhri.2023.10.008
824. Hochstetler E, Taweh O, Mistry AJ, Metz P. Worcester Refugee Assistance Project: An Example of Strengths-Based, Community-Based, Culturally Sensitive Care. Child Adolesc Psychiatr Clin N Am. 2024 Apr;33(2):263-276. Epub 2023 Oct 20. https://doi.org/10.1016/j.chc.2023.10.003
825. Warrens H, Jeyapala J, Blakeway H, Craig A, Tol I. Proposing a curriculum framework for refugee and migrant health for UK medical students. Future Healthc J. 2024 Sep 27;11(4):100190. eCollection 2024 Dec. https://doi.org/10.1016/j.fhj.2024.100190
826. Stevens AJ, Boukari Y, English S, Kadir A, Kumar BN, Devakumar D. Discriminatory, racist and xenophobic policies and practice against child refugees, asylum seekers and undocumented migrants in European health systems. Lancet Reg Health Eur. 2024 May 28;41:100834. eCollection 2024 Jun. https://doi.org/10.1016/j.lanepe.2023.100834
827. Sammouri J, Khachfe HH, Fares MY, Salhab HA, Nassar AH, Chamsy D. Deliveries in Lebanon, the Country with the Highest Refugee Density in the World: A Descriptive Review.

Matern Child Health J. 2024 Apr;28(4):601-608. Epub 2023 Nov 18. https://doi.org/10.1007/s10995-023-03826-x
828. McCleary JS, Horn TL. Processes for culturally adapting behavioral health interventions for people with refugee backgrounds: A scoping review. Am J Community Psychol. 2024 Mar;73(1-2):250-266. Epub 2023 Oct 19. https://doi.org/10.1002/ajcp.12709
829. Mpakosi A, Cholevas V, Tzouvelekis I, Cholevas S, Gavrilis PR, Katsouda A, Passos ID, Kalampaliki O, Mironidou-Tzouveleki M. The History of the Greek Refugee Hospital of the Interwar Period in a General Hospital of Nikaia "Agios Panteleimon". Cureus. 2024 Feb 22;16(2):e54698. eCollection 2024 Feb. https://doi.org/10.7759/cureus.54698
830. Ghandour L, Brown G, Tleis M, Al Masri H, Fares M, Al Halabi F, Najjar Y, Louis B, Afifi RA, Nakkash R. Structural and political determinants of health among Syrian refugee young adults in the Bekaa, Lebanon: a prospective cohort study of the impact of security raids on perceived discrimination and well-being. BMJ Open. 2024 Dec 5;14(12):e087777. https://doi.org/10.1136/bmjopen-2024-087777
831. Gettler LT, Jankovic-Rankovic J, Gengo RG, Eick GN, Nash MP, Arumah EN, Boru AM, Ali SA, Urlacher SS, Meyer JS, Snodgrass JJ, Oka RC. Refugee health and physiological profiles in transitional settlements in Serbia and Kenya: Comparative evidence for effects of gender and social support. Psychoneuroendocrinology. 2024 Jul;165:107024. Epub 2024 Mar 12. https://doi.org/10.1016/j.psyneuen.2024.107024
832. Al-Hamad A, Yasin YM, Metersky K, Guruge S, Jung G, Mahsud K. Homestay Hosting Dynamics and Refugee Well-Being: Scoping Review. Interact J Med Res. 2024 Nov 25;13:e58613. https://doi.org/10.2196/58613
833. Kunnuji M, Kanaahe B, Roth C, Bukoye F, Atukunda D, Alayande S, Schaub E, Esiet A, Marlow H, Izugbara C. Transactional sex in humanitarian settings: A comparative analysis of livelihood and demographic predictors. Afr J Reprod Health. 2024 Aug 31;28(8s):62-73. https://doi.org/10.29063/ajrh2024/v28i8s.7
834. Santambrogio J, Ciscato V, Lorusso O, Wisidagamage Don P, Leon E, Miragliotta E, Capuzzi E, Colmegna F, Clerici M. The challenge of diagnosing cultural syndromes: A narrative review. Int J Soc Psychiatry. 2024 Sep;70(6):1016-1027. Epub 2024 Mar 12. https://doi.org/10.1177/00207640241232335
835. Rzepka I, Kindermann D, Friederich HC, Nikendei C. Secondary traumatization in refugee care-EMDR intervention for interpreters (STEIN): a study protocol for a quasi-randomized controlled trial. Trials. 2024 Oct 1;25(1):643. https://doi.org/10.1186/s13063-024-08480-4
836. Gantz L, Pak-Gorstein S, Gutierrez JR, Noor Z, Shah S. Immigrant and Refugee Health Curricula in US-Based Medical Training: A Scoping Review to Inform Integration Into Pediatric Residency Programs. Acad Pediatr. 2024 Jul;24(5S):103-111. https://doi.org/10.1016/j.acap.2023.05.022
837. Wagaba MT, Musoke D, Opio C, Bagonza A, Aweko J, Nakitende H, Mulyowa A, Ediau M, Waiswa P, Ekirapa-Kiracho E. Do cash or digital payment modalities affect community health worker performance? - a case study of a remote refugee settlement in Western Uganda. Glob Health Action. 2024 Dec 31;17(1):2375867. Epub 2024 Aug 23. https://doi.org/10.1080/16549716.2024.2375867
838. Matlin SA, Hanefeld J, Corte-Real A, da Cunha PR, de Gruchy T, Manji KN, Netto G, Nunes T, Şanlıer İ, Takian A, Zaman MH, Saso L. Digital solutions for migrant and refugee health: a framework for analysis and action. Lancet Reg Health Eur. 2024 Dec 26;50:101190. eCollection 2025 Mar. https://doi.org/10.1016/j.lanepe.2024.101190

839. Harris P, Twose O, Ni Chaoilte A, Cinardo P, Bradbeer L, Longley N, Eisen S, Ward A. Empowering professionals: a multidisciplinary approach to supporting people seeking asylum and refugees. BMJ Paediatr Open. 2024 Dec 24;8(1):e002869. https://doi.org/10.1136/bmjpo-2024-002869
840. MacFarlane A, Huschke S, Marques MJ, Gama A, Kinaan W, Hassan A, Papyan A, Phelan H, Severoni S, Kumar B, Dias S. Normalising participatory health research approaches in the WHO European region for refugee and migrant health: a paradigm shift. Lancet Reg Health Eur. 2024 May 28;41:100837. eCollection 2024 Jun. https://doi.org/10.1016/j.lanepe.2024.100837
841. Cronin A, Hannigan A, Ibrahim N, Seidler Y, Owoeye BO, Gasmalla W, Moyles T, MacFarlane A. An updated scoping review of migrant health research in Ireland. BMC Public Health. 2024 May 28;24(1):1425. https://doi.org/10.1186/s12889-024-18920-0
842. Boettcher J, Heinrich M, Boettche M, Burchert S, Glaesmer H, Gouzoulis-Mayfrank E, Heeke C, Hernek M, Knaevelsrud C, Konnopka A, Muntendorf L, Nilles H, Nohr L, Pohl S, Paskuy S, Reinhardt I, Sierau S, Stammel N, Wirz C, Renneberg B, Wagner B. Internet-based transdiagnostic treatment for emotional disorders in Arabic- and Farsi-speaking refugees: study protocol of a randomized controlled trial. Trials. 2024 Jan 2;25(1):13. https://doi.org/10.1186/s13063-023-07845-5
843. Metersky K, Guruge S, Wang L, Al-Hamad A, Yasin YM, Catallo C, Yang L, Salma J, Zhuang ZC, Chahine M, Kirkwood M, Al-Anani A. Transnational Healthcare Practices Among Migrants: A Concept Analysis. J Adv Nurs. 2024 Dec 25. Online ahead of print. https://doi.org/10.1111/jan.16693
844. Boateng GO, Wachter K, Schuster RC, Burgess TL, Bunn M. A Scoping Review of Instruments Used in Measuring Social Support among Refugees in Resettlement. Int J Environ Res Public Health. 2024 Jun 20;21(6):805. https://doi.org/10.3390/ijerph21060805
845. Oepen R, Gruber H. Art-based interventions and art therapy to promote health of migrant populations - a systematic literature review of current research. Arts Health. 2024 Oct;16(3):266-284. Epub 2023 Sep 4. https://doi.org/10.1080/17533015.2023.2252003
846. Iordache MA, Blanchard AE. Perceptions of Afghan and Ukrainian refugees in Europe. Acta Psychol (Amst). 2024 Sep;249:104439. Epub 2024 Aug 12. https://doi.org/10.1016/j.actpsy.2024.104439
847. Kanengoni-Nyatara B, Watson K, Galindo C, Charania NA, Mpofu C, Holroyd E. Barriers to and Recommendations for Equitable Access to Healthcare for Migrants and Refugees in Aotearoa, New Zealand: An Integrative Review. J Immigr Minor Health. 2024 Feb;26(1):164-180. Epub 2023 Sep 4. https://doi.org/10.1007/s10903-023-01528-8
848. van den Broek M, Agondeze S, Greene MC, Kasujja R, Guevara AF, Kisakye Tukahiirwa R, Kohrt BA, Jordans MJD. A community case detection tool to promote help-seeking for mental health care among children and adolescents in Ugandan refugee settlements: a stepped wedge cluster randomised trial. Lancet Child Adolesc Health. 2024 Aug;8(8):571-579. https://doi.org/10.1016/S2352-4642(24)00130-5
849. Wieland ML, Molina L, Goodson M, Capetillo GP, Osman A, Ahmed Y, Elmi H, Nur O, Iteghete SO, Torres-Herbeck G, Dirie H, Clark MM, Lohr AM, Smith K, Zeratsky K, Rieck T, Herrin J, Valente TW, Sia IG. Healthy immigrant community study protocol: A randomized controlled trial of a social network intervention for cardiovascular risk reduction among Hispanic and Somali adults. Contemp Clin Trials. 2024 Mar;138:107465. Epub 2024 Feb 2. https://doi.org/10.1016/j.cct.2024.107465

850. Krystallidou D, Temizöz Ö, Wang F, de Looper M, Di Maria E, Gattiglia N, Giani S, Hieke G, Morganti W, Pace CS, Schouten B, Braun S. Communication in refugee and migrant mental healthcare: A systematic rapid review on the needs, barriers and strategies of seekers and providers of mental health services. Health Policy. 2024 Jan;139:104949. Epub 2023 Nov 28. https://doi.org/10.1016/j.healthpol.2023.104949
851. Okon II, Rehman IU, Amir MA, Musharaf I, Lucero-Prisno Iii DE, Atallah O, Musa MK, Udokang EI, Chaurasia B. Addressing neurosurgical challenges in war conflict countries. Neurosurg Rev. 2024 Aug 1;47(1):390. https://doi.org/10.1007/s10143-024-02655-y
852. Petrie G, Angus K, O'Donnell R. A scoping review of academic and grey literature on migrant health research conducted in Scotland. BMC Public Health. 2024 Apr 25;24(1):1156. https://doi.org/10.1186/s12889-024-18628-1
853. Liu O, van Gelderen E, Giwa G, Biswas A, Nair S, Garcia AV, Chidiac C, Rhee DS. A Scoping Review of Limited English Proficiency and Immigration in Pediatric Surgery. J Surg Res. 2024 Oct;302:540-554. Epub 2024 Aug 22. https://doi.org/10.1016/j.jss.2024.07.097
854. Markey K, Msowoya U, Burduladze N, Salsberg J, MacFarlane A, Dore L, Gilfoyle M. Antecedents and Consequences of Health Literacy among Refugees and Migrants during the First Two Years of COVID-19: A Scoping Review. Trop Med Infect Dis. 2024 May 16;9(5):116. https://doi.org/10.3390/tropicalmed9050116
855. Hitch L, Masoud D, Moujabber M, Hobbs LA, Cravero K. COVID-19, migrants, and world large urban areas: a thematic policy brief. J Public Health Policy. 2024 Dec;45(4):757-770. Epub 2024 Nov 21. https://doi.org/10.1057/s41271-024-00519-9
856. Yim SH, Said G, King D. Practical recommendations for addressing the psychological needs of unaccompanied asylum-seeking children in England: A literature and service review. Clin Child Psychol Psychiatry. 2025 Apr;30(2):245-263. Epub 2024 Jun 13. https://doi.org/10.1177/13591045241252858
857. Barış M, Sert G, Önder O. Ethical challenges in accessing and providing healthcare for Syrian refugees in Türkiye. Bioethics. 2025 Jan;39(1):49-57. Epub 2023 Nov 8. https://doi.org/10.1111/bioe.13233
858. Bitterfeld L, Ozkaynak M, Denton AH, Normeshie CA, Valdez RS, Sharif N, Caldwell PA, Hauck FR. Interventions to Improve Health Among Refugees in the United States: A Systematic Review. J Community Health. 2025 Feb;50(1):130-151. Epub 2024 Sep 6. https://doi.org/10.1007/s10900-024-01400-2
859. Snyder CS, May JD, Zulcic NN, Gabbard WJ. Social work with Bosnian Muslim refugee children and families: a review of the literature. Child Welfare. 2005 Sep-Oct;84(5):607-30.
860. Abaya M, Lesley B, Williams C, Chaves-Gnecco D, Flores G. Forcible Displacement, Migration, and Violence Against Children and Families in Latin America. Pediatr Clin North Am. 2021 Apr;68(2):371-387. Epub 2021 Jan 26. https://doi.org/10.1016/j.pcl.2020.12.003
861. Wise PH. Advocacy for Unaccompanied Migrant Children in US Detention. Pediatr Clin North Am. 2023 Feb;70(1):103-116. https://doi.org/10.1016/j.pcl.2022.09.006
862. Welch L. Understanding the Legal Rights and Mental Health Needs of Unaccompanied Immigrant Children in US Government Custody and Beyond. Child Adolesc Psychiatr Clin N Am. 2024 Apr;33(2):151-161. Epub 2023 Nov 18. https://doi.org/10.1016/j.chc.2023.10.007
863. Bala A, Pierce J, Pierce K, Song S. Advocacy and Policy: A Focus on Migrant Youth. Child Adolesc Psychiatr Clin N Am. 2024 Apr;33(2):163-180. Epub 2023 Oct 28. https://doi.org/10.1016/j.chc.2023.09.004
864. Nasir S, Goto R, Kitamura A, Alafeef S, Ballout G, Hababeh M, Kiriya J, Seita A, Jimba M. Dissemination and implementation of the e-MCHHandbook, UNRWA's newly released

maternal and child health mobile application: a cross-sectional study. BMJ Open. 2020 Mar 9;10(3):e034885. https://doi.org/10.1136/bmjopen-2019-034885
865. Garfì M, Tondelli S, Bonoli A. Multi-criteria decision analysis for waste management in Saharawi refugee camps. Waste Manag. 2009 Oct;29(10):2729-39. Epub 2009 Jun 27. https://doi.org/10.1016/j.wasman.2009.05.019
866. Augusterfer EF, Mollica RF, Lavelle J. Leveraging Technology in Post-Disaster Settings: the Role of Digital Health/Telemental Health. Curr Psychiatry Rep. 2018 Aug 28;20(10):88. https://doi.org/10.1007/s11920-018-0953-4
867. Enticott JC, Shawyer F, Vasi S, Buck K, Cheng IH, Russell G, Kakuma R, Minas H, Meadows G. A systematic review of studies with a representative sample of refugees and asylum seekers living in the community for participation in mental health research. BMC Med Res Methodol. 2017 Mar 2;17(1):37. https://doi.org/10.1186/s12874-017-0312-x
868. de Anstiss H, Ziaian T, Procter N, Warland J, Baghurst P. Help-seeking for mental health problems in young refugees: a review of the literature with implications for policy, practice, and research. Transcult Psychiatry. 2009 Dec;46(4):584-607. https://doi.org/10.1177/1363461509351363
869. Bartovic J, Padovese V, Pahlman K. Addressing the challenges to skin health of refugees and migrants in the WHO European region. Trop Med Int Health. 2021 May;26(5):602-606. Epub 2021 Feb 15. https://doi.org/10.1111/tmi.13552
870. Rousseau C, Drapeau A, Platt R. Living conditions and emotional profiles of Cambodian, Central American, and Québécois youth. Can J Psychiatry. 2000 Dec;45(10):905-11. https://doi.org/10.1177/070674370004501005
871. Pedersen FK, Møller NE. [Diseases among refugee and immigrant children]. Ugeskr Laeger. 2000 Nov 13;162(46):6207-9.
872. Ahmad A, Sundelin-Wahlsten V, Sofi MA, Qahar JA, von Knorring AL. Reliability and validity of a child-specific cross-cultural instrument for assessing posttraumatic stress disorder. Eur Child Adolesc Psychiatry. 2000 Dec;9(4):285-94. https://doi.org/10.1007/s007870070032
873. Pearn J. Pediatric diseases and operational deployments. Mil Med. 2000 Apr;165(4):283-6.
874. van Ree CJ, Schulpen TW. [Ethical shortcomings of skeletal age determination to establish minority for single young asylum seekers]. Ned Tijdschr Geneeskd. 2001 Feb 3;145(5):229-33.
875. Berman H. Children and war: current understandings and future directions. Public Health Nurs. 2001 Jul-Aug;18(4):243-52. https://doi.org/10.1046/j.1525-1446.2001.00243.x
876. Wahlsten VS, Ahmad A, Von Knorring AL. Do Kurdistanian and Swedish parents and children differ in their rating of competence and behavioural problems?. Nord J Psychiatry. 2002;56(4):279-83. https://doi.org/10.1080/08039480260242778
877. Haataja L, McGready R, Arunjerdja R, Simpson JA, Mercuri E, Nosten F, Dubowitz L. A new approach for neurological evaluation of infants in resource-poor settings. Ann Trop Paediatr. 2002 Dec;22(4):355-68. https://doi.org/10.1179/027249302125002029
878. Seal A, McGrath M, Seal A, Taylor A. Infant feeding indicators for use in emergencies: an analysis of current recommendations and practice. Public Health Nutr. 2002 Jun;5(3):365-72. https://doi.org/10.1079/phn2001310
879. Woodruff BA, Duffield A. Anthropometric assessment of nutritional status in adolescent populations in humanitarian emergencies. Eur J Clin Nutr. 2002 Nov;56(11):1108-18. https://doi.org/10.1038/sj.ejcn.1601456

880. Goldin S, Levin L, Persson LA, Hägglöf B. Child war trauma: a comparison of clinician, parent and child assessments. Nord J Psychiatry. 2003;57(3):173-83. https://doi.org/10.1080/08039480310001319
881. Renzaho AM. Fat, rich and beautiful: changing socio-cultural paradigms associated with obesity risk, nutritional status and refugee children from sub-Saharan Africa. Health Place. 2004 Mar;10(1):105-13. https://doi.org/10.1016/s1353-8292(03)00051-0
882. Khawaja M. The extraordinary decline of infant and childhood mortality among Palestinian refugees. Soc Sci Med. 2004 Feb;58(3):463-70. https://doi.org/10.1016/s0277-9536(03)00212-0
883. Rousseau C, Drapeau A, Lacroix L, Bagilishya D, Heusch N. Evaluation of a classroom program of creative expression workshops for refugee and immigrant children. J Child Psychol Psychiatry. 2005 Feb;46(2):180-5. https://doi.org/10.1111/j.1469-7610.2004.00344.x
884. Mjönes S. Refugee children--a concern for European paediatricians. Eur J Pediatr. 2005 Sep;164(9):535-8. Epub 2005 May 24. https://doi.org/10.1007/s00431-005-1696-x
885. Thabet AA, Vostanis P, Karim K. Group crisis intervention for children during ongoing war conflict. Eur Child Adolesc Psychiatry. 2005 Aug;14(5):262-9. https://doi.org/10.1007/s00787-005-0466-7
886. Hieu NT, Gainsborough M, Simpson JA, Thuy NT, Hang NN, Taylor AM, Ghebremeskel K, Crawford M, Golfetto I, Dubowitz L, Farrar J. Neurological status of low-risk Vietnamese newborns: a comparison with a British newborn cohort. J Health Popul Nutr. 2006 Mar;24(1):57-63.
887. Garcia-Lascurain MC, Kicklighter JR, Jonnalagadda SS, Boudolf EA, Duchon D. Effect of a nutrition education program on nutrition-related knowledge of English-as-second-language elementary school students: a pilot study. J Immigr Minor Health. 2006 Jan;8(1):57-65. https://doi.org/10.1007/s10903-006-6342-9
888. Rousseau C, Guzder J. School-based prevention programs for refugee children. Child Adolesc Psychiatr Clin N Am. 2008 Jul;17(3):533-49, viii. https://doi.org/10.1016/j.chc.2008.02.002
889. Neuner F, Catani C, Ruf M, Schauer E, Schauer M, Elbert T. Narrative exposure therapy for the treatment of traumatized children and adolescents (KidNET): from neurocognitive theory to field intervention. Child Adolesc Psychiatr Clin N Am. 2008 Jul;17(3):641-64, x. https://doi.org/10.1016/j.chc.2008.03.001
890. Manual for the Health Care of Children in Humanitarian Emergencies. Geneva: World Health Organization; 2008.
891. Raman S, Wood N, Webber M, Taylor KA, Isaacs D. Matching health needs of refugee children with services: how big is the gap?. Aust N Z J Public Health. 2009 Oct;33(5):466-70. https://doi.org/10.1111/j.1753-6405.2009.00431.x
892. Schwekendiek D, Pak S. Recent growth of children in the two Koreas: a meta-analysis. Econ Hum Biol. 2009 Mar;7(1):109-12. Epub 2009 Jan 16. https://doi.org/10.1016/j.ehb.2009.01.001
893. Rousseau C, Benoit M, Lacroix L, Gauthier MF. Evaluation of a sandplay program for preschoolers in a multiethnic neighborhood. J Child Psychol Psychiatry. 2009 Jun;50(6):743-50. Epub 2008 Dec 17. https://doi.org/10.1111/j.1469-7610.2008.02003.x
894. Pak S. The growth status of North Korean refugee children and adolescents from 6 to 19 years of age. Econ Hum Biol. 2010 Dec;8(3):385-95. Epub 2010 May 19. https://doi.org/10.1016/j.ehb.2010.05.006

895. Carlson BE, Cacciatore J, Klimek B. A risk and resilience perspective on unaccompanied refugee minors. Soc Work. 2012 Jul;57(3):259-69. https://doi.org/10.1093/sw/sws003
896. Tyrer RA, Fazel M. School and community-based interventions for refugee and asylum seeking children: a systematic review. PLoS One. 2014 Feb 24;9(2):e89359. eCollection 2014. https://doi.org/10.1371/journal.pone.0089359
897. Gibbs L, Waters E, de Silva A, Riggs E, Moore L, Armit C, Johnson B, Morris M, Calache H, Gussy M, Young D, Tadic M, Christian B, Gondal I, Watt R, Pradel V, Truong M, Gold L. An exploratory trial implementing a community-based child oral health promotion intervention for Australian families from refugee and migrant backgrounds: a protocol paper for Teeth Tales. BMJ Open. 2014 Mar 12;4(3):e004260. https://doi.org/10.1136/bmjopen-2013-004260
898. Quach A, Laemmle-Ruff IL, Polizzi T, Paxton GA. Gaps in smiles and services: a cross-sectional study of dental caries in refugee-background children. BMC Oral Health. 2015 Jan 22;15:10. https://doi.org/10.1186/1472-6831-15-10
899. Seery T, Boswell H, Lara A. Caring for Refugee Children. Pediatr Rev. 2015 Aug;36(8):323-38, quiz 339-40. https://doi.org/10.1542/pir.36-8-323
900. Slobodin O, de Jong JT. Family interventions in traumatized immigrants and refugees: A systematic review. Transcult Psychiatry. 2015 Dec;52(6):723-42. Epub 2015 Jun 5. https://doi.org/10.1177/1363461515588855
901. Zwi K, Joshua P, Moran P, White L. Prioritizing vulnerable children: strategies to address inequity. Child Care Health Dev. 2015 Nov;41(6):827-35. Epub 2015 Jun 14. https://doi.org/10.1111/cch.12265
902. Stanley N, Ellis J, Farrelly N, Hollinghurst S, Bailey S, Downe S. Preventing domestic abuse for children and young people (PEACH): a mixed knowledge scoping review. Southampton (UK): NIHR Journals Library; 2015 Jun.
903. Veenema TG, Thornton CP, Corley A. The public health crisis of child sexual abuse in low and middle income countries: an integrative review of the literature. Int J Nurs Stud. 2015 Apr;52(4):864-81. Epub 2014 Nov 7. https://doi.org/10.1016/j.ijnurstu.2014.10.017
904. LeBrun A, Hassan G, Boivin M, Fraser SL, Dufour S, Lavergne C. Review of child maltreatment in immigrant and refugee families. Can J Public Health. 2016 Mar 14;106(7 Suppl 2):eS45-56. https://doi.org/10.17269/cjph.106.4838
905. Graham HR, Minhas RS, Paxton G. Learning Problems in Children of Refugee Background: A Systematic Review. Pediatrics. 2016 Jun;137(6):e20153994. https://doi.org/10.1542/peds.2015-3994
906. Kaplan I, Stolk Y, Valibhoy M, Tucker A, Baker J. Cognitive assessment of refugee children: Effects of trauma and new language acquisition. Transcult Psychiatry. 2016 Feb;53(1):81-109. Epub 2015 Nov 12. https://doi.org/10.1177/1363461515612933
907. Dawson-Hahn E, Pak-Gorstein S, Matheson J, Zhou C, Yun K, Scott K, Payton C, Stein E, Holland A, Grow HM, Mendoza JA. Growth Trajectories of Refugee and Nonrefugee Children in the United States. Pediatrics. 2016 Dec;138(6):e20160953. Epub 2016 Nov 10. https://doi.org/10.1542/peds.2016-0953
908. Dawson-Hahn EE, Pak-Gorstein S, Hoopes AJ, Matheson J. Comparison of the Nutritional Status of Overseas Refugee Children with Low Income Children in Washington State. PLoS One. 2016 Jan 25;11(1):e0147854. eCollection 2016. https://doi.org/10.1371/journal.pone.0147854
909. van Os EC, Kalverboer ME, Zijlstra AE, Post WJ, Knorth EJ. Knowledge of the Unknown Child: A Systematic Review of the Elements of the Best Interests of the Child

Assessment for Recently Arrived Refugee Children. Clin Child Fam Psychol Rev. 2016 Sep;19(3):185-203. https://doi.org/10.1007/s10567-016-0209-y
910. Yun K, Matheson J, Payton C, Scott KC, Stone BL, Song L, Stauffer WM, Urban K, Young J, Mamo B. Health Profiles of Newly Arrived Refugee Children in the United States, 2006-2012. Am J Public Health. 2016 Jan;106(1):128-35. Epub 2015 Nov 12. https://doi.org/10.2105/AJPH.2015.302873
911. Cowden JD, Kreisler K. Development in Children of Immigrant Families. Pediatr Clin North Am. 2016 Oct;63(5):775-93. https://doi.org/10.1016/j.pcl.2016.06.005
912. Hirani K, Payne D, Mutch R, Cherian S. Health of adolescent refugees resettling in high-income countries. Arch Dis Child. 2016 Jul;101(7):670-6. Epub 2015 Oct 15. https://doi.org/10.1136/archdischild-2014-307221
913. Forum on Investing in Young Children Globally; Board on Global Health; Board on Children, Youth, and Families; Health and Medicine Division; Division of Behavioral and Social Sciences and Education; National Academies of Sciences, Engineering, and Medicine. Reaching and Investing in Children at the Margins: Workshop in Brief. Washington (DC): National Academies Press (US); 2016 Mar 16.
914. Forum on Investing in Young Children Globally; Board on Global Health; Board on Children, Youth, and Families; Health and Medicine Division; Division of Behavioral and Social Sciences and Education; National Academies of Sciences, Engineering, and Medicine. Investing in Young Children for Peaceful Societies: Individual and Structural Transformation: Workshop in Brief. Washington (DC): National Academies Press (US); 2016 Jun 24.
915. Pitt MB, Gladding SP, Suchdev PS, Howard CR. Pediatric Global Health Education: Past, Present, and Future. JAMA Pediatr. 2016 Jan;170(1):78-84. https://doi.org/10.1001/jamapediatrics.2015.2368
916. Forum on Investing in Young Children Globally; Board on Global Health; Board on Children, Youth, and Families; Health and Medicine Division; Division of Behavioral and Social Sciences and Education; National Academies of Sciences, Engineering, and Medicine. Investing in Young Children for Peaceful Societies: Proceedings of a Joint Workshop. Washington (DC): National Academies Press (US); 2016 Oct 14.
917. Belen BF, Polat M, Özsevik SN, Soylu E. Frequency of neutropenia among Turkish and Syrian pediatric thalassemia patients under deferiprone monotherapy. Pediatr Hematol Oncol. 2016 Feb;33(1):51-8. Epub 2016 Feb 26. https://doi.org/10.3109/08880018.2015.1106627
918. Cyril S, Halliday J, Green J, Renzaho AM. Relationship between body mass index and family functioning, family communication, family type and parenting style among African migrant parents and children in Victoria, Australia: a parent-child dyad study. BMC Public Health. 2016 Aug 3;15:707. https://doi.org/10.1186/s12889-016-3394-1
919. Stolk Y, Kaplan I, Szwarc J. Review of the strengths and difficulties questionnaire translated into languages spoken by children and adolescents of refugee background. Int J Methods Psychiatr Res. 2017 Dec;26(4):e1568. Epub 2017 Apr 27. https://doi.org/10.1002/mpr.1568
920. Wong CWS, Schweitzer RD. Individual, premigration and postsettlement factors, and academic achievement in adolescents from refugee backgrounds: A systematic review and model. Transcult Psychiatry. 2017 Oct-Dec;54(5-6):756-782. Epub 2017 Nov 8. https://doi.org/10.1177/1363461517737015
921. Kvaal SI, Haugen M. Comparisons between skeletal and dental age assessment in unaccompanied asylum seeking children. J Forensic Odontostomatol. 2017 Dec 1;35(2):109-116.

922. Lichtl C, Lutz T, Szecsenyi J, Bozorgmehr K. Differences in the prevalence of hospitalizations and utilization of emergency outpatient services for ambulatory care sensitive conditions between asylum-seeking children and children of the general population: a cross-sectional medical records study (2015). BMC Health Serv Res. 2017 Nov 15;17(1):731. https://doi.org/10.1186/s12913-017-2672-7
923. Desai N, Romano ME. Pediatric and Adolescent Issues in Underserved Populations. Prim Care. 2017 Mar;44(1):33-45. Epub 2017 Jan 2. https://doi.org/10.1016/j.pop.2016.09.007
924. Hamiel D, Wolmer L, Pardo-Aviv L, Laor N. Addressing the Needs of Preschool Children in the Context of Disasters and Terrorism: Clinical Pictures and Moderating Factors. Curr Psychiatry Rep. 2017 Jul;19(7):38. https://doi.org/10.1007/s11920-017-0793-7
925. Carroll GJ, Lama SD, Martinez-Brockman JL, Pérez-Escamilla R. Evaluation of Nutrition Interventions in Children in Conflict Zones: A Narrative Review. Adv Nutr. 2017 Sep 15;8(5):770-779. Print 2017 Sep. https://doi.org/10.3945/an.117.016121
926. Wofford MC, Tibi S. A human right to literacy education: Implications for serving Syrian refugee children. Int J Speech Lang Pathol. 2018 Feb;20(1):182-190. Epub 2017 Nov 24. https://doi.org/10.1080/17549507.2017.1397746
927. Abuhaloob L, Carson S, Richards D, Freeman R. Community-based nutrition intervention to promote oral health and restore healthy body weight in refugee children: a scoping review. Community Dent Health. 2018 May 30;35(2):81-88. https://doi.org/10.1922/CDH_4188Abuhaloob08
928. Given-Wilson Z, Hodes M, Herlihy J. A review of adolescent autobiographical memory and the implications for assessment of unaccompanied minors' refugee determinations. Clin Child Psychol Psychiatry. 2018 Apr;23(2):209-222. Epub 2017 Dec 20. https://doi.org/10.1177/1359104517748697
929. Nakeyar C, Esses V, Reid GJ. The psychosocial needs of refugee children and youth and best practices for filling these needs: A systematic review. Clin Child Psychol Psychiatry. 2018 Apr;23(2):186-208. Epub 2017 Dec 5. https://doi.org/10.1177/1359104517742188
930. Lane G, Farag M, White J, Nisbet C, Vatanparast H. Chronic health disparities among refugee and immigrant children in Canada. Appl Physiol Nutr Metab. 2018 Oct;43(10):1043-1058. https://doi.org/10.1139/apnm-2017-0407
931. Teksoz E, Düzgüner V, Bilgin I, Ocakci AF. The Impact of a Nursing Coping Kit and a Nursing Coping Bouncy Castle on the Medical Fear Levels of Uzbek Refugee Children. J Pediatr Nurs. 2018 Mar-Apr;39:68-73. Epub 2018 Feb 1. https://doi.org/10.1016/j.pedn.2018.01.010
932. Kobylianskii A, Jegathesan T, Young E, Fung K, Huber J, Minhas RS. Experiences of Inner-City Fathers of Children With Chronic Illness. Clin Pediatr (Phila). 2018 Jun;57(7):792-801. Epub 2017 Oct 3. https://doi.org/10.1177/0009922817734361
933. ISSOP Migration Working Group. ISSOP position statement on migrant child health. Child Care Health Dev. 2018 Jan;44(1):161-170. Epub 2017 Jul 23. https://doi.org/10.1111/cch.12485
934. Richter LM, Lye SJ, Proulx K. Nurturing Care for Young Children under Conditions of Fragility and Conflict. New Dir Child Adolesc Dev. 2018 Mar;2018(159):13-26. https://doi.org/10.1002/cad.20232
935. Wadia U, Soon W, Chivers P, Thambiran A, Burgner D, Cherian S, Siafarikas A. Randomised Controlled Trial Comparing Daily Versus Depot Vitamin D3 Therapy in 0-16-Year-Old Newly Settled Refugees in Western Australia Over a Period of 40 Weeks. Nutrients. 2018 Mar 13;10(3):348. https://doi.org/10.3390/nu10030348

936. van den Berg MM, Khader A, Hababeh M, Zeidan W, Pivetta S, Abd El-Kader M, Al-Jadba G, Seita A. Stalled decline in infant mortality among Palestine refugees in the Gaza Strip since 2006. PLoS One. 2018 Jun 13;13(6):e0197314. eCollection 2018. https://doi.org/10.1371/journal.pone.0197314
937. Grijalva-Eternod CS, Jelle M, Haghparast-Bidgoli H, Colbourn T, Golden K, King S, Cox CL, Morrison J, Skordis-Worrall J, Fottrell E, Seal AJ. A cash-based intervention and the risk of acute malnutrition in children aged 6-59 months living in internally displaced persons camps in Mogadishu, Somalia: A non-randomised cluster trial. PLoS Med. 2018 Oct 29;15(10):e1002684. eCollection 2018 Oct. https://doi.org/10.1371/journal.pmed.1002684
938. Buyukbese Sarsu S, Budeyri A. Mortality risk factors in war-related pediatric burns: A comparative study among two distinct populations. Burns. 2018 Aug;44(5):1210-1227. Epub 2018 Mar 16. https://doi.org/10.1016/j.burns.2018.02.014
939. Kliem S, Sandner M, Lohmann A, Sierau S, Dähne V, Klein AM, Jungmann T. Follow-up study regarding the medium-term effectiveness of the home-visiting program "Pro Kind" at age 7 years: study protocol for a randomized controlled trial. Trials. 2018 Jun 20;19(1):323. https://doi.org/10.1186/s13063-018-2707-3
940. Devries KM, Fabbri C, Allen E, Barongo V, Shayo E, Greco G, Kaemingk M, Qiu M, Steinacher R, Tol W, Rodrigues K. Preventing violence against children in schools (PVACS): protocol for a cluster randomised controlled trial of the EmpaTeach behavioural intervention in Nyarugusu refugee camp. BMC Public Health. 2019 Oct 15;19(1):1295. https://doi.org/10.1186/s12889-019-7627-y
941. Joury E. Syria Profile of the Epidemiology and Management of Early Childhood Caries Before and During the Time of Crisis. Front Public Health. 2019 Sep 24;7:271. eCollection 2019. https://doi.org/10.3389/fpubh.2019.00271
942. Metzler J, Diaconu K, Hermosilla S, Kaijuka R, Ebulu G, Savage K, Ager A. Short- and longer-term impacts of Child Friendly Space Interventions in Rwamwanja Refugee Settlement, Uganda. J Child Psychol Psychiatry. 2019 Nov;60(11):1152-1163. Epub 2019 May 20. https://doi.org/10.1111/jcpp.13069
943. Christenson JC, Chehab H. Pediatric Travelers and Immigrant Children. Pediatr Ann. 2019 Sep 1;48(9):e360-e369. https://doi.org/10.3928/19382359-20190812-01
944. Taylor LK, Goldberg MG, Tran MD. Promoting Student Success: How Do We Best Support Child and Youth Survivors of Catastrophic Events?. Curr Psychiatry Rep. 2019 Aug 13;21(9):82. https://doi.org/10.1007/s11920-019-1067-3
945. Westbom L, Hägglund G. [Refugee/immigrant children with cerebral palsy in the Swedish health care organization]. Lakartidningen. 2019 Jul 1;116:FL9L.
946. Kimyon S, Çelemler P, Mete A, Güngör K. Comparison of retinopathy of prematurity incidence between Turkish citizens and Syrian refugees. Indian J Ophthalmol. 2019 Jun;67(6):811-815. https://doi.org/10.4103/ijo.IJO_1639_18
947. Watson J, Dreibelbis R, Aunger R, Deola C, King K, Long S, Chase RP, Cumming O. Child's play: Harnessing play and curiosity motives to improve child handwashing in a humanitarian setting. Int J Hyg Environ Health. 2019 Mar;222(2):177-182. Epub 2018 Sep 13. https://doi.org/10.1016/j.ijheh.2018.09.002
948. Hermosilla S, Metzler J, Savage K, Musa M, Ager A. Child friendly spaces impact across five humanitarian settings: a meta-analysis. BMC Public Health. 2019 May 15;19(1):576. https://doi.org/10.1186/s12889-019-6939-2
949. Stevens AJ. How can we meet the health needs of child refugees, asylum seekers and undocumented migrants?. Arch Dis Child. 2020 Feb;105(2):191-196. Epub 2019 Oct 11. https://doi.org/10.1136/archdischild-2018-316614

950. Pottie K, Ratnayake A, Ahmed R, Veronis L, Alghazali I. How refugee youth use social media: what does this mean for improving their health and welfare?. J Public Health Policy. 2020 Sep;41(3):268-278. https://doi.org/10.1057/s41271-020-00231-4
951. Weine S, Brahmbatt Z, Cardeli E, Ellis H. Rapid Review to Inform the Rehabilitation and Reintegration of Child Returnees from the Islamic State. Ann Glob Health. 2020 Jun 19;86(1):64. https://doi.org/10.5334/aogh.2835
952. Nazer D, Greenbaum J. Human Trafficking of Children. Pediatr Ann. 2020 May 1;49(5):e209-e214. https://doi.org/10.3928/19382359-20200417-01
953. Veale A. Conflict-driven social change: the case of Syrian children and youth. Curr Opin Psychol. 2020 Oct;35:114-118. Epub 2020 Jun 9. https://doi.org/10.1016/j.copsyc.2020.06.001
954. Sever MS, Sever L, Vanholder R. Disasters, children and the kidneys. Pediatr Nephrol. 2020 Aug;35(8):1381-1393. Epub 2019 Aug 17. https://doi.org/10.1007/s00467-019-04310-x
955. Amsalu R, Schulte-Hillen C, Garcia DM, Lafferty N, Morris CN, Gee S, Akseer N, Scudder E, Sami S, Barasa SO, Had H, Maalim MF, Moluh S, Berkelhamer S. Lessons Learned From Helping Babies Survive in Humanitarian Settings. Pediatrics. 2020 Oct;146(Suppl 2):S208-S217. https://doi.org/10.1542/peds.2020-016915L
956. Brandenberger J, Bozorgmehr K, Vogt F, Tylleskär T, Ritz N. Preventable admissions and emergency-department-visits in pediatric asylum-seeking and non-asylum-seeking patients. Int J Equity Health. 2020 May 1;19(1):58. https://doi.org/10.1186/s12939-020-01172-w
957. Bottorff JL, Huisken A, Hopkins M, Nesmith C. A RE-AIM evaluation of Healthy Together: a family-centred program to support children's healthy weights. BMC Public Health. 2020 Nov 23;20(1):1754. https://doi.org/10.1186/s12889-020-09737-8
958. Jones N, Pincock K, Baird S, Yadete W, Hamory Hicks J. Intersecting inequalities, gender and adolescent health in Ethiopia. Int J Equity Health. 2020 Jun 15;19(1):97. https://doi.org/10.1186/s12939-020-01214-3
959. Suchdev PS, Jefferds MED, Ota E, da Silva Lopes K, De-Regil LM. Home fortification of foods with multiple micronutrient powders for health and nutrition in children under two years of age. Cochrane Database Syst Rev. 2020 Feb 28;2(2):CD008959. https://doi.org/10.1002/14651858.CD008959.pub3
960. Salami B, Mogale S, Ojo F, Kariwo M, Thompson J, Okeke-Ihejirika P, Yohani S. Health of African Refugee Children Outside Africa: A Scoping Review. J Pediatr Nurs. 2021 Nov-Dec;61:199-206. Epub 2021 Jun 9. https://doi.org/10.1016/j.pedn.2021.06.001
961. Larran J, Schuster I, Hein S. The feasibility of implementing autism intervention methods in formal education settings welcoming refugee and asylum-seeking children: A systematic review of the literature. New Dir Child Adolesc Dev. 2021 Sep;2021(179):7-28. Epub 2021 Dec 19. https://doi.org/10.1002/cad.20449
962. Bajo Marcos E, Serrano I, Fernández García MM. The antecedents of well-being in first-generation migrant children: A systematic review. Appl Psychol Health Well Being. 2021 Aug;13(3):677-692. Epub 2021 May 20. https://doi.org/10.1111/aphw.12282
963. Sahin E, Dagli TE, Acarturk C, Sahin Dagli F. Vulnerabilities of Syrian refugee children in Turkey and actions taken for prevention and management in terms of health and wellbeing. Child Abuse Negl. 2021 Sep;119(Pt 1):104628. Epub 2020 Jul 29. https://doi.org/10.1016/j.chiabu.2020.104628

964. Shaw SA, Ward KP, Pillai V, Ali LM, Karim H. A Randomized Clinical Trial Testing a Parenting Intervention Among Afghan and Rohingya Refugees in Malaysia. Fam Process. 2021 Sep;60(3):788-805. Epub 2020 Sep 27. https://doi.org/10.1111/famp.12592
965. Donnelly O, Leavey G. Screening Tools for Mental Disorders Among Female Refugees: a Systematic Review. J Child Adolesc Trauma. 2021 Jul 30;15(2):209-219. eCollection 2022 Jun. https://doi.org/10.1007/s40653-021-00375-9
966. Patras J, Saus M, Douglas M, Bjørknes R, Gammelsæter S, Rasmussen LP, Halvorsen T, Haug IM, Risholm R, Øktedalen T, Jakobsen R, Neumer SP. Parenting interventions for families with refugee backgrounds: a randomized factorial, mixed-methods design study protocol. Trials. 2021 Nov 11;22(1):790. https://doi.org/10.1186/s13063-021-05766-9
967. Simha S, Brown AC. Preventive Care in Children and Adolescents. Prim Care. 2021 Mar;48(1):99-116. Epub 2020 Nov 27. https://doi.org/10.1016/j.pop.2020.09.007
968. Salami B, Fernandez-Sanchez H, Fouche C, Evans C, Sibeko L, Tulli M, Bulaong A, Kwankye SO, Ani-Amponsah M, Okeke-Ihejirika P, Gommaa H, Agbemenu K, Ndikom CM, Richter S. A Scoping Review of the Health of African Immigrant and Refugee Children. Int J Environ Res Public Health. 2021 Mar 28;18(7):3514. https://doi.org/10.3390/ijerph18073514
969. Shah S, Padhani ZA, Als D, Munyuzangabo M, Gaffey MF, Ahmed W, Siddiqui FJ, Meteke S, Kamali M, Jain RP, Radhakrishnan A, Ataullahjan A, Das JK, Bhutta ZA. Delivering nutrition interventions to women and children in conflict settings: a systematic review. BMJ Glob Health. 2021 Apr;6(4):e004897. https://doi.org/10.1136/bmjgh-2020-004897
970. Lawrence JA, Dodds AE, Kaplan I, Tucci MM. Ambivalence towards the Protection of Refugee Children: A Developmental Relational Approach. Int J Environ Res Public Health. 2022 Jan 30;19(3):1602. https://doi.org/10.3390/ijerph19031602
971. Balza JS, Bikomeye JC, Beyer KMM, Rublee C, Flynn KE. Elevated blood lead levels of refugee children in the United States: a systematic review of recent literature (2011-2021). Rev Environ Health. 2022 Apr 21;38(2):361-383. Print 2023 Jun 27. https://doi.org/10.1515/reveh-2022-0015
972. Lindsay K, Hanes G, Mutch R, McKinnon E, Cherian S. Looking beyond: complex holistic care needs of Syrian and Iraqi refugee children and adolescents. Arch Dis Child. 2022 May;107(5):461-467. Epub 2021 Oct 26. https://doi.org/10.1136/archdischild-2021-322718
973. Kevers R, Spaas C, Derluyn I, de Smet S, Van Den Noortgate W, Colpin H, De Haene L. The effect of a school-based creative expression program on immigrant and refugee children's mental health and classroom social relationships: A cluster randomized trial in elementary school. Am J Orthopsychiatry. 2022;92(5):599-615. Epub 2022 Jun 27. https://doi.org/10.1037/ort0000628
974. Kankaanpää R, Aalto S, Vänskä M, Lepistö R, Punamäki RL, Soye E, Watters C, Andersen A, Hilden PK, Derluyn I, Verelst A, Peltonen K. Effectiveness of psychosocial school interventions in Finnish schools for refugee and immigrant children, "Refugees Well School" in Finland (RWS-FI): a protocol for a cluster randomized controlled trial. Trials. 2022 Jan 27;23(1):79. https://doi.org/10.1186/s13063-021-05715-6
975. Abu-Shamsieh A, Maw S. Pediatric Care for Immigrant, Refugee, and Internationally Adopted Children. Pediatr Clin North Am. 2022 Feb;69(1):153-170. https://doi.org/10.1016/j.pcl.2021.09.006
976. Metzner F, Adedeji A, Wichmann ML, Zaheer Z, Schneider L, Schlachzig L, Richters J, Heumann S, Mays D. Experiences of Discrimination and Everyday Racism Among Children and Adolescents With an Immigrant Background - Results of a Systematic Literature Review

on the Impact of Discrimination on the Developmental Outcomes of Minors Worldwide. Front Psychol. 2022 May 9;13:805941. eCollection 2022. https://doi.org/10.3389/fpsyg.2022.805941
977. Hodgins M, Ostojic K, Hu N, Lawson KD, Samir N, Webster A, Rogers H, Henry A, Murphy E, Lingam R, Raman S, Mendoza Diaz A, Dadich A, Eapen V, Rimes T, Woolfenden S. Study protocol for a real-world evaluation of an integrated child and family health hub for migrant and refugee women. BMJ Open. 2022 Aug 30;12(8):e061002. https://doi.org/10.1136/bmjopen-2022-061002
978. Spielberger B, Jackel-Neusser K, Schimana W, Fressle R, Langer T. [Care of refugee children and adolescents with chronic diseases and disabilities]. Monatsschr Kinderheilkd. 2022;170(12):1085-1095. Epub 2022 Sep 28. https://doi.org/10.1007/s00112-022-01608-3
979. Kampalath V, MacLean S, AlAbdulhadi A, Congdon M. The delivery of essential newborn care in conflict settings: A systematic review. Front Pediatr. 2022 Nov 1;10:937751. eCollection 2022. https://doi.org/10.3389/fped.2022.937751
980. Mak C, Wieling E. A Systematic Review of Evidence-Based Family Interventions for Trauma-Affected Refugees. Int J Environ Res Public Health. 2022 Jul 30;19(15):9361. https://doi.org/10.3390/ijerph19159361
981. Garcia MF, Birman D. Understanding the migration experience of unaccompanied youth: A review of the literature. Am J Orthopsychiatry. 2022;92(1):79-102. Epub 2021 Dec 9. https://doi.org/10.1037/ort0000588
982. Salami B, Olukotun M, Vastani M, Amodu O, Tetreault B, Obegu PO, Plaquin J, Sanni O. Immigrant child health in Canada: a scoping review. BMJ Glob Health. 2022 Apr;7(4):e008189. https://doi.org/10.1136/bmjgh-2021-008189
983. Khan MS, Saeedullah A, Andrews SC, Iqbal K, Qadir SA, Shahzad B, Ahmed Z, Shahzad M. Adolescent Afghan Refugees Display a High Prevalence of Hyperhomocysteinemia and Associated Micronutrients Deficiencies Indicating an Enhanced Risk of Cardiovascular Disease in Later Life. Nutrients. 2022 Apr 22;14(9):1751. https://doi.org/10.3390/nu14091751
984. Neville SE, DiClemente-Bosco K, Chamlagai LK, Bunn M, Freeman J, Berent JM, Gautam B, Abdi A, Betancourt TS. Investigating Outcomes of a Family Strengthening Intervention for Resettled Somali Bantu and Bhutanese Refugees: An Explanatory Sequential Mixed Methods Study. Int J Environ Res Public Health. 2022 Sep 29;19(19):12415. https://doi.org/10.3390/ijerph191912415
985. Shohel MMC, Babu R, Ashrafuzzaman M, Azim F, Bayezid A. Learning Competency Framework and Approach for the Displaced Rohingya Children Living in Bangladesh: A Critical Review. Contin Educ. 2023 Mar 15;4(1):50-66. eCollection 2023. https://doi.org/10.5334/cie.57
986. Benjeddi H, Kwee D, Gruppen M, van der Kuip M, van Hensbroek MB, Furth MT. Nutritional status of refugee children living in temporary settlements in Europe and MENA region: a systematic review and meta-analysis. Eur J Pediatr. 2023 Aug;182(8):3397-3404. Epub 2023 May 23. https://doi.org/10.1007/s00431-023-04999-x
987. Palik J, Østby G. Interventions to improve refugee children's access to education and quality learning: A scoping review of existing impact evaluations. Int Rev Educ. 2023;69(1-2):227-247. Epub 2023 May 29. https://doi.org/10.1007/s11159-023-10004-2
988. Higgins C, Gartland D, Yelland J, Brown S, Szwarc J, Kaplan I, Paxton G, Riggs E. Refugee child health: a systematic review of health conditions in children aged 0-6 years

living in high-income countries. Glob Health Promot. 2023 Dec;30(4):45-55. Epub 2023 Jul 4. https://doi.org/10.1177/17579759231165309
989. Amarasena L, Zwi K, Hu N, Lingam R, Raman S. Changing landscape of paediatric refugee health in South Western Sydney, Australia: a retrospective observational study. BMJ Open. 2023 Oct 18;13(10):e064497. https://doi.org/10.1136/bmjopen-2022-064497
990. Choudhary P, Padhi BK, Mital AK, Gandhi AP, Mishra SK, Suri N, Baral SS, Satapathy P, Shamim MA, Thangavelu L, Rustagi S, Sah R, Khatib MN, Gaidhane S, Zahiruddin QS, Abd-Alrazaq A, Abu Serhan H. Prevalence of stunting among under-five children in refugee and internally displaced communities: a systematic review and meta-analysis. Front Public Health. 2023 Nov 29;11:1278343. eCollection 2023. https://doi.org/10.3389/fpubh.2023.1278343
991. Hock E, Blank L, Fairbrother H, Clowes M, Cuevas DC, Booth A, Goyder E. Exploring the impact of housing insecurity on the health and well-being of children and young people: a systematic review. Public Health Res (Southampt). 2023 Dec;11(13):1-71. https://doi.org/10.3310/TWWL4501
992. Chierici DK, Hamdan AC. Cognitive evaluation in unaccompanied refugee children: a systematic review. Rev Paul Pediatr. 2023 May 15;41:e2022079. eCollection 2023. https://doi.org/10.1590/1984-0462/2023/41/2022079
993. Kampalath V, Tarakji A, Hamze M, Loutfi R, Cohn K, Abbara A. The impacts of the Syrian conflict on child and adolescent health: a scoping review. J Public Health (Oxf). 2023 Aug 28;45(3):621-630. https://doi.org/10.1093/pubmed/fdac132
994. Watson J, Osman IM, Amon-Tanoh M, Deola C, MacDougall A, Cumming O. A cluster-randomised controlled equivalence trial of the Surprise Soap handwashing intervention among older children living in a refugee settlement in Sudan. BMJ Glob Health. 2023 Oct;8(10):e012633. https://doi.org/10.1136/bmjgh-2023-012633
995. Duke T. Randomised controlled trials in child and adolescent health in 2023. Arch Dis Child. 2023 Sep;108(9):709-714. Epub 2023 Jul 20. https://doi.org/10.1136/archdischild-2023-326046
996. Abu-Ras W, Ashraf AbuLaban A, Talat AlQaisi S, AlQaisi MTH, Decker E. Orphans in Syria and Iraq Juggling Balls: Wars, COVID-19, and the NGO's financial crisis. Int J Qual Stud Health Well-being. 2023 Dec;18(1):2170010. https://doi.org/10.1080/17482631.2023.2170010
997. Brown FL, Taha K, Steen F, Kane J, Gillman A, Aoun M, Malik A, Bryant R, Sijbrandij M, El Chammay R, Servili C, van Ommeren M, Akhtar A, Zoghbi E; EASE Intervention Development, Training Team; Jordans MJD; STRENGTHS Consortium. Feasibility randomised controlled trial of the Early Adolescent Skills for Emotions psychological intervention with young adolescents in Lebanon. BMC Psychiatry. 2023 Mar 1;23(1):131. https://doi.org/10.1186/s12888-023-04571-9
998. Bernhardt K, Le Beherec S, Uppendahl JR, Fleischmann M, Klosinski M, Rivera LM, Samaras G, Kenney M, Müller R, Nehring I, Mall V, Hahnefeld A. Young children's development after forced displacement: a systematic review. Child Adolesc Psychiatry Ment Health. 2024 Feb 1;18(1):20. https://doi.org/10.1186/s13034-024-00711-5
999. Huynh I, Li CKW. Protective and Promotive Factors in Migrant and Refugee Children Facing Violence: A Systematic Review. Trauma Violence Abuse. 2024 Oct 13:15248380241287157. Online ahead of print. https://doi.org/10.1177/15248380241287157

1000. Fortuna LR, Porche MV. Upholding the Human Rights and Well-Being of Refugee Children Through Effective Clinical Care. Child Adolesc Psychiatr Clin N Am. 2024 Apr;33(2):111-124. Epub 2023 Oct 29. https://doi.org/10.1016/j.chc.2023.09.003
1001. Abdelhamid S, Kraaijenvanger E, Fischer J, Steinisch M. Assessing adverse childhood experiences in young refugees: a systematic review of available questionnaires. Eur Child Adolesc Psychiatry. 2024 Dec;33(12):4043-4059. Epub 2024 Mar 7. https://doi.org/10.1007/s00787-023-02367-6
1002. Ibrahim A, Linton JM, Dawson-Hahn E. Providing Compassionate, Evidence-Based Care for Refugee, Immigrant, and Migrant Children. Adv Pediatr. 2024 Aug;71(1):1-16. Epub 2024 Feb 29. https://doi.org/10.1016/j.yapd.2024.01.001
1003. Aleer E, Alam K, Rashid A. A Systematic Literature Review of Substance-Use Prevention Programs Amongst Refugee Youth. Community Ment Health J. 2024 Aug;60(6):1151-1170. Epub 2024 Apr 9. https://doi.org/10.1007/s10597-024-01267-6
1004. Bajo Marcos E, Fabretti V, Ordóñez-Carabaño Á, Rodríguez-Ventosa Herrera E, Taviani S. A child-centred intercultural approach to the socio-educational inclusion of migrant and refugee children. Open Res Eur. 2024 Oct 17;3:220. eCollection 2023. https://doi.org/10.12688/openreseurope.16999.1
1005. Lembke EJ, Linderkamp F, Casale G. Trauma-sensitive school concepts for students with a refugee background: a review of international studies. Front Psychol. 2024 May 2;15:1321373. eCollection 2024. https://doi.org/10.3389/fpsyg.2024.1321373
1006. Mattelin E, Paidar K, Söderlind N, Fröberg F, Korhonen L. A systematic review of studies on resilience and risk and protective factors for health among refugee children in Nordic countries. Eur Child Adolesc Psychiatry. 2024 Mar;33(3):667-700. Epub 2022 Apr 20. https://doi.org/10.1007/s00787-022-01975-y
1007. Hadfield K, Al-Hamad M, Dajani R, El Kharouf A, Michalek J, Qtaishat L, von Stumm S, Mareschal I. Effectiveness of a community-led shared book reading intervention in Syrian refugee children: a randomised controlled trial. Sci Rep. 2024 Aug 1;14(1):17822. https://doi.org/10.1038/s41598-024-68903-9
1008. Demir E, Duzguner V, Atici A, Yengil E. Oxidative stress responses of virtual reality use in refugee children undergoing elective surgery: A randomized controlled trial. J Pediatr Nurs. 2024 Mar-Apr;75:80-88. Epub 2023 Dec 19. https://doi.org/10.1016/j.pedn.2023.11.004
1009. Agbonyitor M. Unaccompanied Children in the Office of Refugee Resettlement Care. Child Adolesc Psychiatr Clin N Am. 2024 Apr;33(2):141-149. Epub 2023 Oct 11. https://doi.org/10.1016/j.chc.2023.09.001
1010. Arifoglu AT, Artan T. A systematic review of the factors influencing the risky behaviors of syrian forced migrant children and adolescents in Turkey. Clin Child Psychol Psychiatry. 2024 Jul;29(3):1195-1212. Epub 2024 Feb 8. https://doi.org/10.1177/13591045241231336
1011. Talukdar R, Ravel V, Barman D, Kumar V, Dutta S, Kanungo S. Prevalence of undernutrition among migrant, refugee, internally displaced children and children of migrated parents in lower-middle-income countries: A meta-analysis of published studies from last twelve years. Diabetes Metab Syndr. 2024 Mar;18(3):102976. Epub 2024 Mar 15. https://doi.org/10.1016/j.dsx.2024.102976
1012. Young J, Binford W, Bochenek MG, Greenbaum J. Health Risks of Unaccompanied Immigrant Children in Federal Custody and in US Communities. Am J Public Health. 2024 Mar;114(3):340-346. Epub 2024 Feb 8. https://doi.org/10.2105/AJPH.2023.307570

1013. Höhne E, Böge K, Karnouk C, Tschorn M, Banaschewski T, Hoell A, Sukale T, Plener P, Schneider F, Padberg F, Hasan A, Rapp MA, Bajbouj M, Kamp-Becker I. Culturally sensitive stepped care for adolescent refugees: efficacy and cost-utility of a multicentric randomized controlled trial. Eur Child Adolesc Psychiatry. 2024 Feb;33(2):581-593. Epub 2023 Mar 16. https://doi.org/10.1007/s00787-023-02179-8
1014. Priest N, Doery K, Lim CK, Lawrence JA, Zoumboulis G, King G, Lamisa D, He F, Wijesuriya R, Mateo CM, Chong S, Truong M, Perry R, King PT, Paki NP, Joseph C, Pagram D, Lekamge RB, Mikolajczak G, Darnett E, Trenerry B, Jha S, Masunga JG, Paradies Y, Kelly Y, Karlsen S, Guo S. Racism and health and wellbeing among children and youth-An updated systematic review and meta-analysis. Soc Sci Med. 2024 Nov;361:117324. Epub 2024 Sep 21. https://doi.org/10.1016/j.socscimed.2024.117324
1015. Panchal P, Usman M, Longkumer T, Babu RS, Khatib MN, Razak SA, Menon K. The hidden crisis: double burden of malnutrition among refugee children in South Asia - a systematic review and meta-analysis from observational studies. Front Nutr. 2025 Feb 10;11:1480319. eCollection 2024. https://doi.org/10.3389/fnut.2024.1480319
1016. Bosqui T, McEwen FS, Chehade N, Moghames P, Skavenski S, Murray L, Karam E, Weierstall-Pust R, Pluess M. What drives change in children receiving telephone-delivered Common Elements Treatment Approach (t-CETA)? A multiple n = 1 study with Syrian refugee children and adolescents in Lebanon. Child Abuse Negl. 2025 Apr;162(Pt 2):106388. Epub 2023 Aug 21. https://doi.org/10.1016/j.chiabu.2023.106388
1017. Trajkovski S, Al-Dabbas MA, Raman S, Giannoutsos N, Langman M, Schmied V. Immigrant and minority parents' experiences in a neonatal intensive care unit: A meta-ethnography review. J Clin Nurs. 2025 Mar;34(3):737-753. Epub 2024 Aug 23. https://doi.org/10.1111/jocn.17402
1018. Catani C, Kohiladevy M, Ruf M, Schauer E, Elbert T, Neuner F. Treating children traumatized by war and Tsunami: a comparison between exposure therapy and meditation-relaxation in North-East Sri Lanka. BMC Psychiatry. 2009 May 13;9:22. https://doi.org/10.1186/1471-244X-9-22
1019. Phung B. Caring for resettled refugee children in the United States: guidelines, challenges and public health perspectives. Front Public Health. 2023 Sep 25;11:1046319. eCollection 2023. https://doi.org/10.3389/fpubh.2023.1046319
1020. Cropley L. The effect of health education interventions on child malaria treatment-seeking practices among mothers in rural refugee villages in Belize, Central America. Health Promot Int. 2004 Dec;19(4):445-52. Epub 2004 Nov 1. https://doi.org/10.1093/heapro/dah406
1021. Schwarzwald H. Illnesses among recently immigrated children. Semin Pediatr Infect Dis. 2005 Apr;16(2):78-83. https://doi.org/10.1053/j.spid.2005.12.003
1022. Pavlopoulou ID, Tanaka M, Dikalioti S, Samoli E, Nisianakis P, Boleti OD, Tsoumakas K. Clinical and laboratory evaluation of new immigrant and refugee children arriving in Greece. BMC Pediatr. 2017 May 26;17(1):132. https://doi.org/10.1186/s12887-017-0888-7
1023. Ali S, Domi S, Abbo B, Abbas R, Bushari T, Al Awad K, Elhassan A, Abdel-Rahman ME. Echocardiographic screening for rheumatic heart disease in 4 515 Sudanese school children: marked disparity between two communities. Cardiovasc J Afr. 2018 Sep/Oct 23;29(5):273-277. Epub 2018 Apr 16. https://doi.org/10.5830/CVJA-2018-022
1024. Chen SJ, Walker PJ, Mulholland K, Graham HR; ARI Review group. Childhood pneumonia in humanitarian emergencies in low- and middle-income countries: A systematic scoping review. J Glob Health. 2022 Apr 9;12:10001. eCollection 2022. https://doi.org/10.7189/jogh.12.10001

1025. Chawla J, Houbby N, Boutros S, Davies S, Farina E, Stewart CG, Munajjed O. Emergency paediatric medicine consultation-a practical guide to a consultation with refugee and asylum-seeking children within the paediatric emergency department. Eur J Pediatr. 2023 Oct;182(10):4379-4387. Epub 2023 Jul 21. https://doi.org/10.1007/s00431-023-05067-0
1026. El-Halabi S, Khader YS, Khdeir MA, Hanson C, Alfvén T, El-Khatib Z. Children Immunization App (CIMA): A Non-randomized Controlled Trial Among Syrian Refugees in Zaatari Camp, Jordan. J Prev (2022). 2023 Apr;44(2):239-252. Epub 2023 Jan 17. https://doi.org/10.1007/s10935-023-00721-7
1027. Logie CH, Loutet MG, Okumu M, MacKenzie F, Coelho M, Lukone SO, Kisubi N, Malhi A, Kyambadde P, Mbuagbaw L. Findings From the Todurujo na Kadurok (Empowering Youth) HIV Self-Testing and Edutainment Comic Randomized Controlled Trial With Refugee Youth in a Humanitarian Setting in Uganda. J Int Assoc Provid AIDS Care. 2024 Jan-Dec;23:23259582241307057. https://doi.org/10.1177/23259582241307057
1028. Panter-Brick C, Wiley K, Sancilio A, Dajani R, Hadfield K. C-reactive protein, Epstein-Barr virus, and cortisol trajectories in refugee and non-refugee youth: Links with stress, mental health, and cognitive function during a randomized controlled trial. Brain Behav Immun. 2020 Jul;87:207-217. Epub 2019 Feb 20. https://doi.org/10.1016/j.bbi.2019.02.015
1029. Baauw A, Kist-van Holthe J, Slattery B, Heymans M, Chinapaw M, van Goudoever H. Health needs of refugee children identified on arrival in reception countries: a systematic review and meta-analysis. BMJ Paediatr Open. 2019 Sep 11;3(1):e000516. eCollection 2019. https://doi.org/10.1136/bmjpo-2019-000516
1030. Svanemyr J, Amin A, Robles OJ, Greene ME. Creating an enabling environment for adolescent sexual and reproductive health: a framework and promising approaches. J Adolesc Health. 2015 Jan;56(1 Suppl):S7-14. https://doi.org/10.1016/j.jadohealth.2014.09.011
1031. Okumu M, Logie CH, Ansong D, Mwima S, Hakiza R, Newman PA. Support for Texting-Based Condom Negotiation Among Forcibly Displaced Adolescents in the Slums of Kampala, Uganda: Cross-sectional Validation of the Condom Use Negotiated Experiences Through Technology Scale. JMIR Public Health Surveill. 2022 Apr 6;8(4):e27792. https://doi.org/10.2196/27792
1032. Slodnjak V, Kos A, Yule W. Depression and parasuicide in refugee and Slovenian adolescents. Crisis. 2002;23(3):127-32. https://doi.org/10.1027//0227-5910.23.3.127
1033. Rousseau C, Benoit M, Gauthier MF, Lacroix L, Alain N, Rojas MV, Moran A, Bourassa D. Classroom drama therapy program for immigrant and refugee adolescents: a pilot study. Clin Child Psychol Psychiatry. 2007 Jul;12(3):451-65. https://doi.org/10.1177/1359104507078477
1034. Ruf M, Schauer M, Neuner F, Catani C, Schauer E, Elbert T. Narrative exposure therapy for 7- to 16-year-olds: a randomized controlled trial with traumatized refugee children. J Trauma Stress. 2010 Aug;23(4):437-45. https://doi.org/10.1002/jts.20548
1035. Björn GJ, Bodén C, Sydsjö G, Gustafsson PA. Psychological evaluation of refugee children: contrasting results from play diagnosis and parental interviews. Clin Child Psychol Psychiatry. 2011 Oct;16(4):517-34. Epub 2011 May 12. https://doi.org/10.1177/1359104510384550
1036. Montgomery E. Trauma, exile and mental health in young refugees. Acta Psychiatr Scand Suppl. 2011;(440):1-46. https://doi.org/10.1111/j.1600-0447.2011.01740.x
1037. Rousseau C, Beauregard C, Daignault K, Petrakos H, Thombs BD, Steele R, Vasiliadis HM, Hechtman L. A cluster randomized-controlled trial of a classroom-based drama

workshop program to improve mental health outcomes among immigrant and refugee youth in special classes. PLoS One. 2014 Aug 15;9(8):e104704. eCollection 2014. https://doi.org/10.1371/journal.pone.0104704
1038. Abraham ZK, Sher L. Adolescent suicide as a global public health issue. Int J Adolesc Med Health. 2017 Jul 7;31(4). https://doi.org/10.1515/ijamh-2017-0036
1039. Budde H, Akko DP, Ainamani HE, Murillo-Rodríguez E, Weierstall R. The impact of an exercise training intervention on cortisol levels and post-traumatic stress disorder in juveniles from an Ugandan refugee settlement: study protocol for a randomized control trial. Trials. 2018 Jul 9;19(1):364. https://doi.org/10.1186/s13063-018-2753-x
1040. Simenec TS, Reid BM. Refugee Children and Interventions for Depression: A Review of Current Interventions and Implications of the Ecological Context. Trauma Violence Abuse. 2022 Jul;23(3):877-890. Epub 2020 Dec 17. https://doi.org/10.1177/1524838020979844
1041. Smeeth D, McEwen FS, Popham CM, Karam EG, Fayyad J, Saab D, Rieder MJ, Elzagallaai AA, van Uum S, Pluess M. War exposure, post-traumatic stress symptoms and hair cortisol concentrations in Syrian refugee children. Mol Psychiatry. 2023 Feb;28(2):647-656. Epub 2022 Nov 16. https://doi.org/10.1038/s41380-022-01859-2
1042. Hawes DJ, Dadds MR, Tully LA, Northam JC; Growing Minds Australia Clinical Trials Network. Building a National Clinical Trials Network in child and youth mental health: Growing Minds Australia. Aust N Z J Psychiatry. 2023 Feb;57(2):164-168. Epub 2022 Mar 5. https://doi.org/10.1177/00048674221082525
1043. Jordans MJD, Brown FL, Kane J, Taha K, Steen F, Ali R, Elias J, Meksassi B, Aoun M, Greene CM, Malik A, Akhtar A, van Ommeren M, Sijbrandij M, Bryant R; STRENGTHS consortium. Evaluation of the Early Adolescent Skills for Emotions (EASE) intervention in Lebanon: A randomized controlled trial. Compr Psychiatry. 2023 Nov;127:152424. Epub 2023 Sep 16. https://doi.org/10.1016/j.comppsych.2023.152424
1044. May AK, Smeeth D, McEwen F, Moghames P, Karam E, Rieder MJ, Elzagallaai AA, van Uum S, Pluess M. Hair hormone data from Syrian refugee children: Perspectives from a two-year longitudinal study. Compr Psychoneuroendocrinol. 2024 Apr 8;18:100231. eCollection 2024 May. https://doi.org/10.1016/j.cpnec.2024.100231
1045. Lawton K, Spencer A. A Full Systematic Review on the Effects of Cognitive Behavioural Therapy for Mental Health Symptoms in Child Refugees. J Immigr Minor Health. 2021 Jun;23(3):624-639. Epub 2021 Feb 15. https://doi.org/10.1007/s10903-021-01151-5
1046. Alamgir A, Kyriakides C, Johnson A, Abeshu G, Bahri B, Abssy M. Resilience Mechanisms and Coping Strategies for Forcibly Displaced Youth: An Exploratory Rapid Review. Int J Environ Res Public Health. 2024 Oct 11;21(10):1347. https://doi.org/10.3390/ijerph21101347
1047. Crespo E. The Importance of Oral Health in Immigrant and Refugee Children. Children (Basel). 2019 Sep 9;6(9):102. https://doi.org/10.3390/children6090102
1048. Noaman BR, Khalid RF, Fattah LD. Maternal Dental Health Knowledge and Its Relation to the Dental Caries Experience of Their Children in Mamyzawa Camp of Refugees in Erbil, Iraq. Acta Med Acad. 2019 Dec;48(3):294-302. https://doi.org/10.5644/ama2006-124.270
1049. Alrashdi M, Cervantes Mendez MJ, Farokhi MR. A Randomized Clinical Trial Preventive Outreach Targeting Dental Caries and Oral-Health-Related Quality of Life for Refugee Children. Int J Environ Res Public Health. 2021 Feb 10;18(4):1686. https://doi.org/10.3390/ijerph18041686

1050. Skinner A, Tester-Jones MC, Carrieri D. Undernutrition among children living in refugee camps: a systematic review of prevalence. BMJ Open. 2023 Jun 15;13(6):e070246. https://doi.org/10.1136/bmjopen-2022-070246
1051. Folayan MO, Schroth RJ, Ayouni I, Nguweneza A, Arheiam A, Al-Batayneh OB, Virtanen JI, Gaffar B, Duangthip D, Sun IG, Mohebbi S, Feldens CA, Tantawi ME. A scoping review linking early childhood caries to violence, neglect, internally displaced, migrant and refugee status. BMC Oral Health. 2023 Oct 11;23(1):747. https://doi.org/10.1186/s12903-023-03459-0
1052. Teketelew BB, Chane E, Angelo AA, Tamir M, Cherie N, Nigus M, Mulatie Z, Berta DM. Global prevalence of anemia in displaced and refugee children: A comprehensive systematic review and meta-analysis. PLoS One. 2024 Nov 22;19(11):e0312905. eCollection 2024. https://doi.org/10.1371/journal.pone.0312905
1053. Neuman V, Vavra D, Drnkova L, Pruhova S, Plachy L, Kolouskova S, Obermannova B, Amaratunga SA, Konecna P, Vyzralkova J, Venhacova P, Pomahacova R, Paterova P, Stichova L, Skvor J, Kocourkova K, Romanova M, Vosahlo J, Strnadel J, Polockova K, Neumann D, Slavenko M, Sumnik Z. Introduction of continuous glucose monitoring (CGM) is a key factor in decreasing HbA1c in war refugee children with type 1 diabetes. Diabetes Res Clin Pract. 2024 Feb;208:111118. Epub 2024 Feb 1. https://doi.org/10.1016/j.diabres.2024.111118
1054. Balla SB, Angelakopoulos N, Tadakamadla J, Tadakamadla SK. A Systematic Review and Meta-Analysis of Interventions Targeted to Parents for Improving the Oral Health of Children from Culturally and Linguistically Diverse (CALD) Backgrounds. J Immigr Minor Health. 2025 Apr;27(2):313-328. Epub 2024 Nov 13. https://doi.org/10.1007/s10903-024-01650-1
1055. Freccero J, Biswas D, Whiting A, Alrabe K, Seelinger KT. Sexual exploitation of unaccompanied migrant and refugee boys in Greece: Approaches to prevention. PLoS Med. 2017 Nov 22;14(11):e1002438. eCollection 2017 Nov. https://doi.org/10.1371/journal.pmed.1002438
1056. Alipui N, Gerke N. The Refugee Crisis and the Rights of Children: Perspectives on Community-Based Resettlement Programs. New Dir Child Adolesc Dev. 2018 Mar;2018(159):91-98. https://doi.org/10.1002/cad.20228
1057. El Harake MD, Kharroubi S, Hamadeh SK, Jomaa L. Impact of a Pilot School-Based Nutrition Intervention on Dietary Knowledge, Attitudes, Behavior and Nutritional Status of Syrian Refugee Children in the Bekaa, Lebanon. Nutrients. 2018 Jul 17;10(7):913. https://doi.org/10.3390/nu10070913
1058. Chen S, Carver A, Sugiyama T, Knöll M. Built-environment attributes associated with refugee children's physical activity: a narrative review and research agenda. Confl Health. 2021 Jul 8;15(1):55. https://doi.org/10.1186/s13031-021-00393-2
1059. Fabbri C, Rodrigues K, Leurent B, Allen E, Qiu M, Zuakulu M, Nombo D, Kaemingk M, De Filippo A, Torrats-Espinosa G, Shayo E, Barongo V, Greco G, Tol W, Devries KM. The EmpaTeach intervention for reducing physical violence from teachers to students in Nyarugusu Refugee Camp: A cluster-randomised controlled trial. PLoS Med. 2021 Oct 4;18(10):e1003808. eCollection 2021 Oct. https://doi.org/10.1371/journal.pmed.1003808
1060. Smith EM, Minescu A. A test of the maintenance of the effects of imagined contact framed with supportive social norms as a teacher-led field intervention. J Sch Psychol. 2022 Jun;92:324-333. Epub 2022 May 6. https://doi.org/10.1016/j.jsp.2022.04.005
1061. Bodiang CK. Issues facing TB control (2.1). Tuberculosis control in refugee populations: a focus on developing countries. Scott Med J. 2000 Oct;45(5 Suppl):25-8; discussion 29. https://doi.org/10.1177/00369330000450S112

1062. Zoguéreh DD, Ndihokubwayo JB, Simboyinuma A. [Epidemic typhus in tropical Africa. A reemerging disease that is severe but curable]. Sante. 2000 Sep-Oct;10(5):339-44.
1063. Lauzardo M, Ashkin D. Phthisiology at the dawn of the new century. Chest. 2000 May;117(5):1455-73. https://doi.org/10.1378/chest.117.5.1455
1064. Koop DG, Jackson BM, Nestel G. Results of the expanded program on immunization in the Macedonian refugee camps. Am J Public Health. 2001 Oct;91(10):1656-9. https://doi.org/10.2105/ajph.91.10.1656
1065. Rowland M, Nosten F. Malaria epidemiology and control in refugee camps and complex emergencies. Ann Trop Med Parasitol. 2001 Dec;95(8):741-54. https://doi.org/10.1080/00034980120103405
1066. Charlwood JD, Qassim M, Elnsur EI, Donnelly M, Petrarca V, Billingsley PF, Pinto J, Smith T. The impact of indoor residual spraying with malathion on malaria in refugee camps in eastern Sudan. Acta Trop. 2001 Sep 1;80(1):1-8. https://doi.org/10.1016/s0001-706x(01)00152-8
1067. Fernández Sanfrancisco MT, Díaz Portillo J, Sánchez Romero JM, Pérez Fernández A, Vadillo Andrade J. [Prevalence of tuberculosis among the immigrant population in Ceuta, Spain]. Rev Esp Salud Publica. 2001 Nov-Dec;75(6):551-8.
1068. Brown V, Jacquier G, Bachy C, Bitar D, Legros D. [Management of cholera epidemics in a refugee camp]. Bull Soc Pathol Exot. 2002 Dec;95(5):351-4.
1069. Piarroux R. [Cholera: epidemiology and transmission. Experience from several humanitarian interventions in Africa, Indian Ocean and Central America]. Bull Soc Pathol Exot. 2002 Dec;95(5):345-50.
1070. Graham K, Mohammad N, Rehman H, Farhan M, Kamal M, Rowland M. Comparison of three pyrethroid treatments of top-sheets for malaria control in emergencies: entomological and user acceptance studies in an Afghan refugee camp in Pakistan. Med Vet Entomol. 2002 Jun;16(2):199-206. https://doi.org/10.1046/j.1365-2915.2002.00366.x
1071. Ivanoff B, Chaignat CL. [Anticholera vaccines and vaccination]. Bull Soc Pathol Exot. 2002 Dec;95(5):355-8.
1072. Langlet P, Mulkay JP, Holvoet J. Issues in managing patients with chronic hepatitis C in public hospitals. Acta Gastroenterol Belg. 2002 Apr-Jun;65(2):101-3.
1073. Crowcroft NS, Morgan D, Brown D. Viral haemorrhagic fevers in Europe--effective control requires a co-ordinated response. Euro Surveill. 2002 Mar;7(3):31-2. https://doi.org/10.2807/esm.07.03.00343-en
1074. Marras TK, Wilson J, Wang EE, Avendano M, Yang JW. Tuberculosis among Tibetan refugee claimants in Toronto: 1998 to 2000. Chest. 2003 Sep;124(3):915-21. https://doi.org/10.1378/chest.124.3.915
1075. Rowland M, Downey G, Rab A, Freeman T, Mohammad N, Rehman H, Durrani N, Reyburn H, Curtis C, Lines J, Fayaz M. DEET mosquito repellent provides personal protection against malaria: a household randomized trial in an Afghan refugee camp in Pakistan. Trop Med Int Health. 2004 Mar;9(3):335-42. https://doi.org/10.1111/j.1365-3156.2004.01198.x
1076. Christiansen D, Barnett ED. Comparison of varicella history with presence of varicella antibody in refugees. Vaccine. 2004 Oct 22;22(31-32):4233-7. https://doi.org/10.1016/j.vaccine.2004.04.024
1077. Depoortere E, Guthmann JP, Sipilanyambe N, Nkandu E, Fermon F, Balkan S, Legros D. Adherence to the combination of sulphadoxine-pyrimethamine and artesunate in the Maheba refugee settlement, Zambia. Trop Med Int Health. 2004 Jan;9(1):62-7. https://doi.org/10.1046/j.1365-3156.2003.01157.x

1078. Kolaczinski J, Mohammed N, Ali I, Ali M, Khan N, Ezard N, Rowland M. Comparison of the OptiMAL rapid antigen test with field microscopy for the detection of Plasmodium vivax and P. falciparum: considerations for the application of the rapid test in Afghanistan. Ann Trop Med Parasitol. 2004 Jan;98(1):15-20. https://doi.org/10.1179/000349804225003127
1079. Leslie T, Rab MA, Ahmadzai H, Durrani N, Fayaz M, Kolaczinski J, Rowland M. Compliance with 14-day primaquine therapy for radical cure of vivax malaria--a randomized placebo-controlled trial comparing unsupervised with supervised treatment. Trans R Soc Trop Med Hyg. 2004 Mar;98(3):168-73. https://doi.org/10.1016/s0035-9203(03)00041-5
1080. Bledsoe GH. The West Nile virus: a lesson in emerging infections. Wilderness Environ Med. 2004 Summer;15(2):113-8. [0113:twnval]2.0.co;2. https://doi.org/10.1580/1080-6032(2004)015
1081. Ndao M, Bandyayera E, Kokoskin E, Gyorkos TW, MacLean JD, Ward BJ. Comparison of blood smear, antigen detection, and nested-PCR methods for screening refugees from regions where malaria is endemic after a malaria outbreak in Quebec, Canada. J Clin Microbiol. 2004 Jun;42(6):2694-700. https://doi.org/10.1128/JCM.42.6.2694-2700.2004
1082. Hadzibegovic DS, Maloney SA, Cookson ST, Oladele A. Determining TB rates and TB case burden for refugees. Int J Tuberc Lung Dis. 2005 Apr;9(4):409-14.
1083. Depoortere E, Guthmann JP, Pressé J, Sipilanyambe N, Nkandu E, Balkan S, de Pécoulas PE, Legros D. Efficacy and effectiveness of the combination of sulfadoxine/pyrimethamine and a 3-day course of artesunate for the treatment of uncomplicated falciparum malaria in a refugee settlement in Zambia. Trop Med Int Health. 2005 Feb;10(2):139-45. https://doi.org/10.1111/j.1365-3156.2004.01363.x
1084. Nyindo M. Complementary factors contributing to the rapid spread of HIV-I in sub-Saharan Africa: a review. East Afr Med J. 2005 Jan;82(1):40-6. https://doi.org/10.4314/eamj.v82i1.9293
1085. Owen IL. Current status of Taenia solium and cysticercosis in Papua New Guinea. Parasitol Int. 2006;55 Suppl:S149-53. Epub 2005 Dec 9. https://doi.org/10.1016/j.parint.2005.11.023
1086. Kimani EW, Vulule JM, Kuria IW, Mugisha F. Use of insecticide-treated clothes for personal protection against malaria: a community trial. Malar J. 2006 Jul 27;5:63. https://doi.org/10.1186/1475-2875-5-63
1087. Hill DR, Ford L, Lalloo DG. Oral cholera vaccines: use in clinical practice. Lancet Infect Dis. 2006 Jun;6(6):361-73. https://doi.org/10.1016/S1473-3099(06)70494-7
1088. Sterling TR, Bethel J, Goldberg S, Weinfurter P, Yun L, Horsburgh CR; Tuberculosis Epidemiologic Studies Cosortium. The scope and impact of treatment of latent tuberculosis infection in the United States and Canada. Am J Respir Crit Care Med. 2006 Apr 15;173(8):927-31. Epub 2006 Jan 19. https://doi.org/10.1164/rccm.200510-1563OC
1089. Bradt DA, Drummond CM. Avian influenza pandemic threat and health systems response. Emerg Med Australas. 2006 Oct-Dec;18(5-6):430-43. https://doi.org/10.1111/j.1742-6723.2006.00906.x
1090. Clark RC, Mytton J. Estimating infectious disease in UK asylum seekers and refugees: a systematic review of prevalence studies. J Public Health (Oxf). 2007 Dec;29(4):420-8. Epub 2007 Oct 8. https://doi.org/10.1093/pubmed/fdm063
1091. Bonnet M, Roper C, Félix M, Coulibaly L, Kankolongo GM, Guthmann JP. Efficacy of antimalarial treatment in Guinea: in vivo study of two artemisinin combination therapies in Dabola and molecular markers of resistance to sulphadoxine-pyrimethamine in N'Zérékoré. Malar J. 2007 May 3;6:54. https://doi.org/10.1186/1475-2875-6-54

1092. Spiegel PB, Bennedsen AR, Claass J, Bruns L, Patterson N, Yiweza D, Schilperoord M. Prevalence of HIV infection in conflict-affected and displaced people in seven sub-Saharan African countries: a systematic review. Lancet. 2007 Jun 30;369(9580):2187-2195. https://doi.org/10.1016/S0140-6736(07)61015-0
1093. Rajabali A, Moin O, Ansari AS, Khanani MR, Ali SH. Communicable disease among displaced Afghans: refuge without shelter. Nat Rev Microbiol. 2009 Aug;7(8):609-14. https://doi.org/10.1038/nrmicro2176
1094. Borch M, Kiernan M, Rust K, Baron B, Simmons B, Hattala P, Davey A, Yovanovich J, Shayder D, Wasilewski A, LaFaro VE. Schistosomiasis: a case study. Urol Nurs. 2009 Jan-Feb;29(1):26-9.
1095. Mérens A, Guérin PJ, Guthmann JP, Nicand E. Outbreak of hepatitis E virus infection in Darfur, Sudan: effectiveness of real-time reverse transcription-PCR analysis of dried blood spots. J Clin Microbiol. 2009 Jun;47(6):1931-3. Epub 2009 Apr 1. https://doi.org/10.1128/JCM.02245-08
1096. Bellos A, Mulholland K, O'Brien KL, Qazi SA, Gayer M, Checchi F. The burden of acute respiratory infections in crisis-affected populations: a systematic review. Confl Health. 2010 Feb 11;4:3. https://doi.org/10.1186/1752-1505-4-3
1097. Roca MG, Charle P, Jimenez S, Nunez M. A new malaria protocol in a Congolese refugee camp in West Tanzania. Glob Public Health. 2011;6(4):398-406. https://doi.org/10.1080/17441692.2010.546805
1098. Howard N, Durrani N, Sanda S, Beshir K, Hallett R, Rowland M. Clinical trial of extended-dose chloroquine for treatment of resistant falciparum malaria among Afghan refugees in Pakistan. Malar J. 2011 Jun 23;10:171. https://doi.org/10.1186/1475-2875-10-171
1099. Jamali S. Role of pyrethroids in control of malaria amongst refugee population. J Pak Med Assoc. 2011 May;61(5):486-90.
1100. Jex AR, Lim YA, Bethony JM, Hotez PJ, Young ND, Gasser RB. Soil-transmitted helminths of humans in Southeast Asia--towards integrated control. Adv Parasitol. 2011;74:231-65. https://doi.org/10.1016/B978-0-12-385897-9.00004-5
1101. Nelson KE, Kmush B, Labrique AB. The epidemiology of hepatitis E virus infections in developed countries and among immunocompromised patients. Expert Rev Anti Infect Ther. 2011 Dec;9(12):1133-48. https://doi.org/10.1586/eri.11.138
1102. Greenaway C, Sandoe A, Vissandjee B, Kitai I, Gruner D, Wobeser W, Pottie K, Ueffing E, Menzies D, Schwartzman K; Canadian Collaboration for Immigrant and Refugee Health. Tuberculosis: evidence review for newly arriving immigrants and refugees. CMAJ. 2011 Sep 6;183(12):E939-51. Epub 2010 Jul 15. https://doi.org/10.1503/cmaj.090302
1103. Mittal PK, Sreehari U, Razdan RK, Dash AP. Evaluation of the impact of ZeroFly®, an insecticide incorporated plastic sheeting on malaria incidence in two temporary labour shelters in India. J Vector Borne Dis. 2011 Sep;48(3):138-43.
1104. Mendelsohn JB, Schilperoord M, Spiegel P, Ross DA. Adherence to antiretroviral therapy and treatment outcomes among conflict-affected and forcibly displaced populations: a systematic review. Confl Health. 2012 Oct 31;6(1):9. https://doi.org/10.1186/1752-1505-6-9
1105. Shantha GP, Kumar AA, Bhise V, Sivagnanam K, Subramanian KK, Kanade P, Khanna R. Screening for latent tuberculosis in refugees with renal failure. Saudi J Kidney Dis Transpl. 2012 Jan;23(1):8-14.
1106. Burns M, Rowland M, N'Guessan R, Carneiro I, Beeche A, Ruiz SS, Kamara S, Takken W, Carnevale P, Allan R. Insecticide-treated plastic sheeting for emergency malaria

prevention and shelter among displaced populations: an observational cohort study in a refugee setting in Sierra Leone. Am J Trop Med Hyg. 2012 Aug;87(2):242-250. https://doi.org/10.4269/ajtmh.2012.11-0744
1107. Badiaga S, Brouqui P. Human louse-transmitted infectious diseases. Clin Microbiol Infect. 2012 Apr;18(4):332-7. Epub 2012 Feb 23. https://doi.org/10.1111/j.1469-0691.2012.03778.x
1108. Verma R, Khanna P, Chawla S. Cholera vaccine: new preventive tool for endemic countries. Hum Vaccin Immunother. 2012 May;8(5):682-4. Epub 2012 May 1. https://doi.org/10.4161/hv.19083
1109. Rossi C, Shrier I, Marshall L, Cnossen S, Schwartzman K, Klein MB, Schwarzer G, Greenaway C. Seroprevalence of chronic hepatitis B virus infection and prior immunity in immigrants and refugees: a systematic review and meta-analysis. PLoS One. 2012;7(9):e44611. Epub 2012 Sep 5. https://doi.org/10.1371/journal.pone.0044611
1110. Pérez-Gracia MT, Mateos Lindemann ML, Caridad Montalvo Villalba M. Hepatitis E: current status. Rev Med Virol. 2013 Nov;23(6):384-98. Epub 2013 Aug 28. https://doi.org/10.1002/rmv.1759
1111. Mendelsohn JB, Schilperoord M, Spiegel P, Balasundaram S, Radhakrishnan A, Lee CK, Larke N, Grant AD, Sondorp E, Ross DA. Is forced migration a barrier to treatment success? Similar HIV treatment outcomes among refugees and a surrounding host community in Kuala Lumpur, Malaysia. AIDS Behav. 2014 Feb;18(2):323-34. https://doi.org/10.1007/s10461-013-0494-0
1112. Suphanchaimat R, Sommanustweechai A, Khitdee C, Thaichinda C, Kantamaturapoj K, Leelahavarong P, Jumriangrit P, Topothai T, Wisaijohn T, Putthasri W. HIV/AIDS health care challenges for cross-country migrants in low- and middle-income countries: a scoping review. HIV AIDS (Auckl). 2014 Feb 26;6:19-38. eCollection 2014. https://doi.org/10.2147/HIV.S56277
1113. Martin S, Lopez AL, Bellos A, Deen J, Ali M, Alberti K, Anh DD, Costa A, Grais RF, Legros D, Luquero FJ, Ghai MB, Perea W, Sack DA. Post-licensure deployment of oral cholera vaccines: a systematic review. Bull World Health Organ. 2014 Dec 1;92(12):881-93. Epub 2014 Sep 29. https://doi.org/10.2471/BLT.14.139949
1114. Kidenya BR, Webster LE, Behan S, Kabangila R, Peck RN, Mshana SE, Ocheretina O, Fitzgerald DW. Epidemiology and genetic diversity of multidrug-resistant tuberculosis in East Africa. Tuberculosis (Edinb). 2014 Jan;94(1):1-7. Epub 2013 Sep 7. https://doi.org/10.1016/j.tube.2013.08.009
1115. O'Connell EM, Nutman TB. Eosinophilia in Infectious Diseases. Immunol Allergy Clin North Am. 2015 Aug;35(3):493-522. https://doi.org/10.1016/j.iac.2015.05.003
1116. Subedi P, Drezner KA, Dogbey MC, Newbern EC, Yun K, Scott KC, Garland JM, Altshuler MJ, Johnson CC. Evaluation of latent tuberculous infection and treatment completion for refugees in Philadelphia, PA, 2010-2012. Int J Tuberc Lung Dis. 2015 May;19(5):565-9. https://doi.org/10.5588/ijtld.14.0729
1117. Richter J, Bode JG, Blondin D, Kircheis G, Kubitz R, Holtfreter MC, Müller-Stöver I, Breuer M, Hüttig F, Antoch G, Häussinger D. Severe liver fibrosis caused by Schistosoma mansoni: management and treatment with a transjugular intrahepatic portosystemic shunt. Lancet Infect Dis. 2015 Jun;15(6):731-7. Epub 2015 Mar 11. https://doi.org/10.1016/S1473-3099(15)70009-5
1118. Lam E, McCarthy A, Brennan M. Vaccine-preventable diseases in humanitarian emergencies among refugee and internally-displaced populations. Hum Vaccin Immunother. 2015;11(11):2627-36. https://doi.org/10.1080/21645515.2015.1096457

1119. Lönnroth K, Migliori GB, Abubakar I, D'Ambrosio L, de Vries G, Diel R, Douglas P, Falzon D, Gaudreau MA, Goletti D, González Ochoa ER, LoBue P, Matteelli A, Njoo H, Solovic I, Story A, Tayeb T, van der Werf MJ, Weil D, Zellweger JP, Abdel Aziz M, Al Lawati MR, Aliberti S, Arrazola de Oñate W, Barreira D, Bhatia V, Blasi F, Bloom A, Bruchfeld J, Castelli F, Centis R, Chemtob D, Cirillo DM, Colorado A, Dadu A, Dahle UR, De Paoli L, Dias HM, Duarte R, Fattorini L, Gaga M, Getahun H, Glaziou P, Goguadze L, Del Granado M, Haas W, Järvinen A, Kwon GY, Mosca D, Nahid P, Nishikiori N, Noguer I, O'Donnell J, Pace-Asciak A, Pompa MG, Popescu GG, Robalo Cordeiro C, Rønning K, Ruhwald M, Sculier JP, Simunović A, Smith-Palmer A, Sotgiu G, Sulis G, Torres-Duque CA, Umeki K, Uplekar M, van Weezenbeek C, Vasankari T, Vitillo RJ, Voniatis C, Wanlin M, Raviglione MC. Towards tuberculosis elimination: an action framework for low-incidence countries. Eur Respir J. 2015 Apr;45(4):928-52. https://doi.org/10.1183/09031936.00214014

1120. Kohl LJ, von Both U, Huebner J. [Vaccination]. MMW Fortschr Med. 2016 May 25;158(10):52-6. https://doi.org/10.1007/s15006-016-8281-2

1121. O'Laughlin KN, Rabideau DJ, Kasozi J, Parker RA, Bustamante ND, Faustin ZM, Greenwald KE, Walensky RP, Bassett IV. Predictors of HIV infection: a prospective HIV screening study in a Ugandan refugee settlement. BMC Infect Dis. 2016 Nov 23;16(1):695. https://doi.org/10.1186/s12879-016-2021-1

1122. Hytönen J, Khawaja T, Grönroos JO, Jalava A, Meri S, Oksi J. Relapsing fever. Duodecim. 2016;132(21):1952-6.

1123. Colomba C, Scarlata F, Di Carlo P, Giammanco A, Fasciana T, Trizzino M, Cascio A. Fourth case of louse-borne relapsing fever in Young Migrant, Sicily, Italy, December 2015. Mini Review Article. Public Health. 2016 Oct;139:22-26. Epub 2016 Jun 21. https://doi.org/10.1016/j.puhe.2016.05.019

1124. Piperaki ET, Daikos GL. Malaria in Europe: emerging threat or minor nuisance?. Clin Microbiol Infect. 2016 Jun;22(6):487-93. Epub 2016 May 10. https://doi.org/10.1016/j.cmi.2016.04.023

1125. Ozaras R, Leblebicioglu H, Sunbul M, Tabak F, Balkan II, Yemisen M, Sencan I, Ozturk R. The Syrian conflict and infectious diseases. Expert Rev Anti Infect Ther. 2016 Jun;14(6):547-55. Epub 2016 Apr 27. https://doi.org/10.1080/14787210.2016.1177457

1126. Rossi R, Assaad R, Rebeschini A, Hamadeh R. Vaccination Coverage Cluster Surveys in Middle Dreib - Akkar, Lebanon: Comparison of Vaccination Coverage in Children Aged 12-59 Months Pre- and Post-Vaccination Campaign. PLoS One. 2016 Dec 19;11(12):e0168145. eCollection 2016. https://doi.org/10.1371/journal.pone.0168145

1127. Al-Salem W, Herricks JR, Hotez PJ. A review of visceral leishmaniasis during the conflict in South Sudan and the consequences for East African countries. Parasit Vectors. 2016 Aug 22;9(1):460. https://doi.org/10.1186/s13071-016-1743-7

1128. Sunderkötter C, Feldmeier H, Fölster-Holst R, Geisel B, Klinke-Rehbein S, Nast A, Philipp S, Sachs B, Stingl J, Stoevesandt J, Hamm H. S1 guidelines on the diagnosis and treatment of scabies - short version. J Dtsch Dermatol Ges. 2016 Nov;14(11):1155-1167. https://doi.org/10.1111/ddg.13130

1129. de Smalen AW, Ghorab H, Abd El Ghany M, Hill-Cawthorne GA. Refugees and antimicrobial resistance: A systematic review. Travel Med Infect Dis. 2017 Jan-Feb;15:23-28. Epub 2016 Dec 3. https://doi.org/10.1016/j.tmaid.2016.12.001

1130. Eiset AH, Wejse C. Review of infectious diseases in refugees and asylum seekers-current status and going forward. Public Health Rev. 2017 Sep 8;38:22. eCollection 2017. https://doi.org/10.1186/s40985-017-0065-4

1131. Cianelli R, Villegas N, McCabe BE, de Tantillo L, Peragallo N. Self-efficacy for HIV Prevention Among Refugee Hispanic Women in South Florida. J Immigr Minor Health. 2017 Aug;19(4):905-912. https://doi.org/10.1007/s10903-016-0462-7

1132. Steverding D. The history of leishmaniasis. Parasit Vectors. 2017 Feb 15;10(1):82. https://doi.org/10.1186/s13071-017-2028-5

1133. Peterson JC, Gonzalez FJ, Schneider SP. Effects of disease salience and xenophobia on support for humanitarian aid. Politics Life Sci. 2017 Fall;36(2):17-36. https://doi.org/10.1017/pls.2017.24

1134. Maltezou HC, Theodoridou M, Daikos GL. Antimicrobial resistance and the current refugee crisis. J Glob Antimicrob Resist. 2017 Sep;10:75-79. Epub 2017 Jul 1. https://doi.org/10.1016/j.jgar.2017.03.013

1135. Tarashi S, Fateh A, Jamnani FR, Siadat SD, Vaziri F. Prevalence of Beijing and Haarlem genotypes among multidrug-resistant Mycobacterium tuberculosis in Iran: Systematic review and meta-analysis. Tuberculosis (Edinb). 2017 Dec;107:31-37. Epub 2017 Mar 24. https://doi.org/10.1016/j.tube.2017.03.005

1136. Bloch-Infanger C, Bättig V, Kremo J, Widmer AF, Egli A, Bingisser R, Battegay M, Erb S. Increasing prevalence of infectious diseases in asylum seekers at a tertiary care hospital in Switzerland. PLoS One. 2017 Jun 15;12(6):e0179537. eCollection 2017. https://doi.org/10.1371/journal.pone.0179537

1137. Ferguson A, Shannon K, Butler J, Goldenberg SM. A comprehensive review of HIV/STI prevention and sexual and reproductive health services among sex Workers in Conflict-Affected Settings: call for an evidence- and rights-based approach in the humanitarian response. Confl Health. 2017 Dec 4;11:25. eCollection 2017. https://doi.org/10.1186/s13031-017-0124-y

1138. Isenring E, Fehr J, Gültekin N, Schlagenhauf P. Infectious disease profiles of Syrian and Eritrean migrants presenting in Europe: A systematic review. Travel Med Infect Dis. 2018 Sep-Oct;25:65-76. Epub 2018 Apr 24. https://doi.org/10.1016/j.tmaid.2018.04.014

1139. Löscher T, Alberer M, Herbinger KH. [Fever in travellers and migrants from the tropics]. MMW Fortschr Med. 2018 Oct;160(18):44-54. https://doi.org/10.1007/s15006-018-0026-y

1140. Nimer NA. A Review on Emerging and Reemerging of Infectious Diseases in Jordan: The Aftermath of the Syrian Crises. Can J Infect Dis Med Microbiol. 2018 May 24;2018:8679174. eCollection 2018. https://doi.org/10.1155/2018/8679174

1141. Hargreaves S, Rustage K, Nellums LB, Powis J, Milburn J, Severoni S, Dara M, Puthoopparambil SJ, Friedland JS. What Constitutes an Effective and Efficient Package of Services for the Prevention, Diagnosis, Treatment and Care of Tuberculosis among Refugees and Migrants in the WHO European Region? Themed Issues on Migration and Health, VIII. Copenhagen: WHO Regional Office for Europe; 2018.

1142. Zadeh C, Tayara L. Tuberculosis at Raffic Hariri University Hospital (RHUH) during 10 years period 2005-2015, cross sectional, observational study. Indian J Tuberc. 2018 Jul;65(3):225-232. Epub 2017 Aug 18. https://doi.org/10.1016/j.ijtb.2017.08.013

1143. Joo H, Maskery B, Mitchell T, Leidner A, Klosovsky A, Weinberg M. A comparative cost analysis of the Vaccination Program for US-bound Refugees. Vaccine. 2018 May 11;36(20):2896-2901. Epub 2017 Sep 14. https://doi.org/10.1016/j.vaccine.2017.09.023

1144. Nellums LB, Thompson H, Holmes A, Castro-Sánchez E, Otter JA, Norredam M, Friedland JS, Hargreaves S. Antimicrobial resistance among migrants in Europe: a systematic review and meta-analysis. Lancet Infect Dis. 2018 Jul;18(7):796-811. Epub 2018 May 17. https://doi.org/10.1016/S1473-3099(18)30219-6

1145. Maia MF, Kliner M, Richardson M, Lengeler C, Moore SJ. Mosquito repellents for malaria prevention. Cochrane Database Syst Rev. 2018 Feb 6;2(2):CD011595. https://doi.org/10.1002/14651858.CD011595.pub2
1146. Boggild AK, Geduld J, Libman M, Yansouni CP, McCarthy AE, Hajek J, Ghesquiere W, Mirzanejad Y, Vincelette J, Kuhn S, Plourde PJ, Chakrabarti S, Greenaway C, Hamer DH, Kain KC. Spectrum of illness in migrants to Canada: sentinel surveillance through CanTravNet. J Travel Med. 2019 Feb 1;26(2):tay117. https://doi.org/10.1093/jtm/tay117
1147. Kibar Öztürk M. Skin diseases in rural Nyala, Sudan (in a rural hospital, in 12 orphanages, and in two refugee camps). Int J Dermatol. 2019 Nov;58(11):1341-1349. Epub 2019 Sep 9. https://doi.org/10.1111/ijd.14619
1148. Havumaki J, Meza R, Phares CR, Date K, Eisenberg MC. Comparing alternative cholera vaccination strategies in Maela refugee camp: using a transmission model in public health practice. BMC Infect Dis. 2019 Dec 21;19(1):1075. https://doi.org/10.1186/s12879-019-4688-6
1149. Shannon K, Hast M, Azman AS, Legros D, McKay H, Lessler J. Cholera prevention and control in refugee settings: Successes and continued challenges. PLoS Negl Trop Dis. 2019 Jun 20;13(6):e0007347. eCollection 2019 Jun. https://doi.org/10.1371/journal.pntd.0007347
1150. Nimer NA. A review on emerging and re-emerging of infectious diseases in Jordan the aftermath of the Syrian crises. J Pak Med Assoc. 2019 Mar;69(3):412-414.
1151. Muttalif AR, Presa JV, Haridy H, Gamil A, Serra LC, Cané A. Incidence and Prevention of Invasive Meningococcal Disease in Global Mass Gathering Events. Infect Dis Ther. 2019 Dec;8(4):569-579. Epub 2019 Aug 30. https://doi.org/10.1007/s40121-019-00262-9
1152. Seth-Smith HMB, Egli A. Whole Genome Sequencing for Surveillance of Diphtheria in Low Incidence Settings. Front Public Health. 2019 Aug 21;7:235. eCollection 2019. https://doi.org/10.3389/fpubh.2019.00235
1153. Chang E. Helminthiases in North Korea: a neglected public health challenge. Pathog Glob Health. 2019 Sep;113(6):256-262. Epub 2019 Oct 28. https://doi.org/10.1080/20477724.2019.1683326
1154. Mitchell T, Weinberg M, Posey DL, Cetron M. Immigrant and Refugee Health: A Centers for Disease Control and Prevention Perspective on Protecting the Health and Health Security of Individuals and Communities During Planned Migrations. Pediatr Clin North Am. 2019 Jun;66(3):549-560. https://doi.org/10.1016/j.pcl.2019.02.004
1155. Blake DP. Key Ways to Prevent Infection When There Is No "Building": Aspects for the Field. Surg Infect (Larchmt). 2019 Feb/Mar;20(2):115-118. Epub 2019 Jan 24. https://doi.org/10.1089/sur.2018.290
1156. Brechot C, Bryant J, Endtz H, Garry RF, Griffin DE, Lewin SR, Mercer N, Osterhaus A, Picot V, Vahlne A, Verjans GMGM, Weaver S. 2018 international meeting of the Global Virus Network. Antiviral Res. 2019 Mar;163:140-148. Epub 2019 Jan 25. https://doi.org/10.1016/j.antiviral.2019.01.013
1157. de Lima Junior MM, Rodrigues GA, Lima MR. Evaluation of emerging infectious disease and the importance of SINAN for epidemiological surveillance of Venezuelans immigrants in Brazil. Braz J Infect Dis. 2019 Sep-Oct;23(5):307-312. Epub 2019 Aug 28. https://doi.org/10.1016/j.bjid.2019.07.006
1158. Dandachi I, Azar E, Hamouch R, Maliha P, Abdallah S, Kanaan E, Badawi R, Khairallah T, Matar GM, Daoud Z. Acinetobacter spp in a Third World Country with Socio-economic and Immigrants Challenges. J Infect Dev Ctries. 2019 Nov 30;13(11):948-955. https://doi.org/10.3855/jidc.11341

1159. Hijawi KJF, Hijjawi NS, Ibbini JH. Detection, genotyping, and phylogenetic analysis of Leishmania isolates collected from infected Jordanian residents and Syrian refugees who suffered from cutaneous leishmaniasis. Parasitol Res. 2019 Mar;118(3):793-805. Epub 2019 Feb 7. https://doi.org/10.1007/s00436-019-06222-z

1160. Peragallo Montano N, Cianelli R, Villegas N, Gonzalez-Guarda R, Williams WO, de Tantillo L. Evaluating a Culturally Tailored HIV Risk Reduction Intervention Among Hispanic Women Delivered in a Real-World Setting by Community Agency Personnel. Am J Health Promot. 2019 May;33(4):566-575. Epub 2018 Oct 24. https://doi.org/10.1177/0890117118807716

1161. Schmit KM, Brostrom R, Largen A, Pyan A, Wang Z, Mase S, Morris S. Higher Rates of Tuberculosis Among Class B1 Filipino Immigrants to Hawaii Compared to Nationwide, 2010-2014. J Immigr Minor Health. 2019 Dec;21(6):1300-1305. https://doi.org/10.1007/s10903-019-00855-z

1162. Browne SH, Umlauf A, Tucker AJ, Low J, Moser K, Gonzalez Garcia J, Peloquin CA, Blaschke T, Vaida F, Benson CA. Wirelessly observed therapy compared to directly observed therapy to confirm and support tuberculosis treatment adherence: A randomized controlled trial. PLoS Med. 2019 Oct 4;16(10):e1002891. eCollection 2019 Oct. https://doi.org/10.1371/journal.pmed.1002891

1163. Michaleas SN, Sergentanis TN, Panourgia N, Psaltopoulou T, Stratigos A, Sipsas NV, Panayiotakopoulos G, Tsoucalas G, Karamanou M. Sexually Transmitted Infections in a Cohort of 15,921 Refugees (1926-1940) in the Region of Imathia, Northern Greece. Acta Dermatovenerol Croat. 2020 Dec;28(3):180-187.

1164. Ergönül Ö, Tülek N, Kayı I, Irmak H, Erdem O, Dara M. Profiling infectious diseases in Turkey after the influx of 3.5 million Syrian refugees. Clin Microbiol Infect. 2020 Mar;26(3):307-312. Epub 2019 Jul 5. https://doi.org/10.1016/j.cmi.2019.06.022

1165. Coates SJ, Thomas C, Chosidow O, Engelman D, Chang AY. Ectoparasites: Pediculosis and tungiasis. J Am Acad Dermatol. 2020 Mar;82(3):551-569. Epub 2019 Jul 13. https://doi.org/10.1016/j.jaad.2019.05.110

1166. Thomas C, Coates SJ, Engelman D, Chosidow O, Chang AY. Ectoparasites: Scabies. J Am Acad Dermatol. 2020 Mar;82(3):533-548. Epub 2019 Jul 13. https://doi.org/10.1016/j.jaad.2019.05.109

1167. Ghatee MA, Taylor WR, Karamian M. The Geographical Distribution of Cutaneous Leishmaniasis Causative Agents in Iran and Its Neighboring Countries, A Review. Front Public Health. 2020 Feb 18;8:11. eCollection 2020. https://doi.org/10.3389/fpubh.2020.00011

1168. Valeriani G, Vukovic IS, Mollica R. Unconventional Answers to Unprecedented Challenges: The Swedish Experience During the COVID-19 Outbreak. J Prev Med Public Health. 2020 Jul;53(4):233-235. Epub 2020 Jul 22. https://doi.org/10.3961/jpmph.20.235

1169. Hejaz HA. Palestinian strategies, guidelines, and challenges in the treatment and management of coronavirus disease-2019 (COVID-19). Avicenna J Med. 2020 Oct 13;10(4):135-162. eCollection 2020 Oct-Dec. https://doi.org/10.4103/ajm.ajm_171_20

1170. Charania NA, Gaze N, Kung JY, Brooks S. Interventions to reduce the burden of vaccine-preventable diseases among migrants and refugees worldwide: A scoping review of published literature, 2006-2018. Vaccine. 2020 Oct 27;38(46):7217-7225. Epub 2020 Sep 30. https://doi.org/10.1016/j.vaccine.2020.09.054

1171. Tankwanchi AS, Jaca A, Larson HJ, Wiysonge CS, Vermund SH. Taking stock of vaccine hesitancy among migrants: a scoping review protocol. BMJ Open. 2020 May 12;10(5):e035225. https://doi.org/10.1136/bmjopen-2019-035225

1172. Wondemagegn F, Berkessa T. High level risky sexual behavior among persons living with HIV in the urban setting of the highest HIV prevalent areas in Ethiopia: Implications for interventions. PLoS One. 2020 Nov 25;15(11):e0242701. eCollection 2020. https://doi.org/10.1371/journal.pone.0242701
1173. Pottie K, Girard V. Common Infectious Diseases. Prim Care. 2021 Mar;48(1):45-55. Epub 2020 Dec 19. https://doi.org/10.1016/j.pop.2020.11.002
1174. Padovese V, Knapp A. Challenges of Managing Skin Diseases in Refugees and Migrants. Dermatol Clin. 2021 Jan;39(1):101-115. Epub 2020 Oct 31. https://doi.org/10.1016/j.det.2020.08.010
1175. Saifee J, Franco-Paredes C, Lowenstein SR. Refugee Health During COVID-19 and Future Pandemics. Curr Trop Med Rep. 2021;8(3):1-4. Epub 2021 Jul 16. https://doi.org/10.1007/s40475-021-00245-2
1176. Hintermeier M, Gencer H, Kajikhina K, Rohleder S, Hövener C, Tallarek M, Spallek J, Bozorgmehr K. SARS-CoV-2 among migrants and forcibly displaced populations: A rapid systematic review. J Migr Health. 2021;4:100056. Epub 2021 Jun 16. https://doi.org/10.1016/j.jmh.2021.100056
1177. Garsow AV, Campbell E, Closs G, Kowalcyk BB. Food Safety Challenges in Refugee Camps: What Do We Know?. J Food Prot. 2021 May 1;84(5):876-884. https://doi.org/10.4315/JFP-20-316
1178. Logie CH, Okumu M, Berry I, Hakiza R, Kibuuka Musoke D, Kyambadde P, Mwima S, Lester RT, Perez-Brumer AG, Baral S, Mbuagbaw L. Kukaa Salama (Staying Safe): study protocol for a pre/post-trial of an interactive mHealth intervention for increasing COVID-19 prevention practices with urban refugee youth in Kampala, Uganda. BMJ Open. 2021 Nov 22;11(11):e055530. https://doi.org/10.1136/bmjopen-2021-055530
1179. Kobeissi E, Menassa M, Moussally K, Repetto E, Soboh I, Hajjar M, Saleh S, Abu-Sittah G. The socioeconomic burden of antibiotic resistance in conflict-affected settings and refugee hosting countries: a systematic scoping review. Confl Health. 2021 Apr 6;15(1):21. https://doi.org/10.1186/s13031-021-00357-6
1180. Jablonka A, Dopfer C, Happle C, Shalabi A, Wetzke M, Hummers E, Friede T, Heinemann S, Hillermann N, Simmenroth A, Müller F. Acute respiratory infections in an adult refugee population: an observational study. NPJ Prim Care Respir Med. 2021 Dec 21;31(1):50. https://doi.org/10.1038/s41533-021-00261-9
1181. Khan S, Akbar SMF, Kimitsuki K, Saito N, Yahiro T, Al Mahtab M, Nishizono A. Recent downhill course of COVID-19 at Rohingya refugee camps in Bangladesh: Urgent action solicited. J Glob Health. 2021 Sep 4;11:03097. eCollection 2021. https://doi.org/10.7189/jogh.11.03097
1182. Lambert JF, Stete K, Balmford J, Bockey A, Kern W, Rieg S, Boeker M, Lange B. Reducing burden from respiratory infections in refugees and immigrants: a systematic review of interventions in OECD, EU, EEA and EU-applicant countries. BMC Infect Dis. 2021 Aug 26;21(1):872. https://doi.org/10.1186/s12879-021-06474-0
1183. Addams J, Lainhart W. Closing the Brief Case: Salmonella enterica Serovar Typhi in a Central American Refugee. J Clin Microbiol. 2021 Apr 20;59(5):e01360-20. Print 2021 Apr 20. https://doi.org/10.1128/JCM.01360-20
1184. Kleinert E, Hillermann N, Jablonka A, Happle C, Müller F, Simmenroth A. Prescription of antibiotics in the medical care of newly arrived refugees and migrants. Pharmacoepidemiol Drug Saf. 2021 Aug;30(8):1074-1083. Epub 2021 May 4. https://doi.org/10.1002/pds.5254

1185. Addams J, Lainhart W. The Brief Case: Salmonella enterica Serovar Typhi in a Central American Refugee. J Clin Microbiol. 2021 Apr 20;59(5):e01359-20. Print 2021 Apr 20. https://doi.org/10.1128/JCM.01359-20
1186. Mishra D, O'Laughlin K, Spiegel P. A systematic review evaluating HIV prevalence among conflict-affected populations, 2005-2020. AIDS Rev. 2021 Jun 2;23(3):143-152. https://doi.org/10.24875/AIDSRev.200001311
1187. Mohareb AM, Rosenberg JM, Bhattacharyya RP, Kotton CN, Chu JT, Jilg N, Hysell KM, Albin JS, Sen P, Bloom SM, Schiff AE, Zachary KC, Letourneau AR, Kim AY, Hurtado RM. Preventing Infectious Complications of Immunomodulation in COVID-19 in Foreign-Born Patients. J Immigr Minor Health. 2021 Dec;23(6):1343-1347. Epub 2021 Jun 22. https://doi.org/10.1007/s10903-021-01225-4
1188. Deal A, Halliday R, Crawshaw AF, Hayward SE, Burnard A, Rustage K, Carter J, Mehrotra A, Knights F, Campos-Matos I, Majeed A, Friedland JS, Edelstein M, Mounier-Jack S, Hargreaves S; European Society of Clinical Microbiology and Infectious Diseases Study Group for Infections in Travellers and Migrants (ESGITM). Migration and outbreaks of vaccine-preventable disease in Europe: a systematic review. Lancet Infect Dis. 2021 Dec;21(12):e387-e398. Epub 2021 Oct 6. https://doi.org/10.1016/S1473-3099(21)00193-6
1189. Kousi T, Mitsi LC, Simos J. The Early Stage of COVID-19 Outbreak in Greece: A Review of the National Response and the Socioeconomic Impact. Int J Environ Res Public Health. 2021 Jan 4;18(1):322. https://doi.org/10.3390/ijerph18010322
1190. Zhang M, Gurung A, Anglewicz P, Yun K. COVID-19 and Immigrant Essential Workers: Bhutanese and Burmese Refugees in the United States. Public Health Rep. 2021 Jan/Feb;136(1):117-123. Epub 2020 Nov 18. https://doi.org/10.1177/0033354920971720
1191. Dembech M, Katz Z, Szilard I. Strengthening Country Readiness for Pandemic-Related Mass Movement: Policy Lessons Learned. Int J Environ Res Public Health. 2021 Jun 12;18(12):6377. https://doi.org/10.3390/ijerph18126377
1192. Dubey AP, Hazarika RD, Abitbol V, Kolhapure S, Agrawal S. Mass gatherings: a review of the scope for meningococcal vaccination in the Indian context. Hum Vaccin Immunother. 2021 Jul 3;17(7):2216-2224. Epub 2021 Feb 19. https://doi.org/10.1080/21645515.2020.1871572
1193. Michel W, Färber J, Dilas M, Heuft HG, Tammer I, Baar J, Kaasch AJ. A combined oro-nasopharyngeal swab is more sensitive than mouthwash in detecting SARS-CoV-2 by a high-throughput PCR assay. Infection. 2021 Jun;49(3):527-531. Epub 2021 Mar 18. https://doi.org/10.1007/s15010-021-01600-1
1194. Trollfors B, Sigurdsson V, Dahlgren-Aronsson A. Prevalence of Latent TB and Effectiveness of BCG Vaccination Against Latent Tuberculosis: An Observational Study. Int J Infect Dis. 2021 Aug;109:279-282. Epub 2021 Jun 24. https://doi.org/10.1016/j.ijid.2021.06.045
1195. Cinardo P, Farrant O, Gunn K, Ward A, Eisen S, Longley N. Screening for neglected tropical diseases and other infections in refugee and asylum-seeker populations in the United Kingdom. Ther Adv Infect Dis. 2022 Aug 6;9:20499361221116680. eCollection 2022 Jan-Dec. https://doi.org/10.1177/20499361221116680
1196. Desai AN, Mohareb AM, Hauser N, Abbara A. Antimicrobial Resistance and Human Mobility. Infect Drug Resist. 2022 Jan 13;15:127-133. eCollection 2022. https://doi.org/10.2147/IDR.S305078
1197. Nichol AA, Parcharidi Z, Al-Delaimy WK, Kondilis E. Rapid Review of COVID-19 Vaccination Access and Acceptance for Global Refugee, Asylum Seeker and Undocumented

Migrant Populations. Int J Public Health. 2022 Dec 22;67:1605508. eCollection 2022. https://doi.org/10.3389/ijph.2022.1605508
1198. Patel PK, Mehrotra P, Ladines-Lim JB. An opportunity for global antimicrobial stewardship research: Refugee populations. Antimicrob Steward Healthc Epidemiol. 2022 Feb 14;2(1):e23. eCollection 2022. https://doi.org/10.1017/ash.2022.8
1199. Crawshaw AF, Farah Y, Deal A, Rustage K, Hayward SE, Carter J, Knights F, Goldsmith LP, Campos-Matos I, Wurie F, Majeed A, Bedford H, Forster AS, Hargreaves S. Defining the determinants of vaccine uptake and undervaccination in migrant populations in Europe to improve routine and COVID-19 vaccine uptake: a systematic review. Lancet Infect Dis. 2022 Sep;22(9):e254-e266. Epub 2022 Apr 13. https://doi.org/10.1016/S1473-3099(22)00066-4
1200. Capturing the evidence on access to essential antibiotics in refugee and migrant populations. Geneva: World Health Organization; 2022.
1201. Logie CH, Okumu M, Loutet MG, Coelho M, Berry I, Gittings L, Odong Lukone S, Kisubi N, Atama M, Kyambadde P. Todurujo na Kadurok (empowering youth): study protocol of an HIV self-testing and edutainment comic cluster randomised trial among refugee youth in a humanitarian setting in Uganda. BMJ Open. 2022 Nov 23;12(11):e065452. https://doi.org/10.1136/bmjopen-2022-065452
1202. Jahn R, Hintermeier M, Bozorgmehr K. SARS-CoV-2 attack rate in reception and accommodation centres for asylum seekers during the first wave: Systematic review of outbreak media reports in Germany. J Migr Health. 2022;5:100084. Epub 2022 Feb 4. https://doi.org/10.1016/j.jmh.2022.100084
1203. Führer A, Özer Erdogdu I, Kompa P, Yilmaz-Aslan Y, Brzoska P. COVID-19 pandemic in shelters for asylum seekers: a scoping review of preventive measures. BMJ Open. 2022 Apr 27;12(4):e058076. https://doi.org/10.1136/bmjopen-2021-058076
1204. Aylett-Bullock J, Gilman RT, Hall I, Kennedy D, Evers ES, Katta A, Ahmed H, Fong K, Adib K, Al Ariqi L, Ardalan A, Nabeth P, von Harbou K, Hoffmann Pham K, Cuesta-Lazaro C, Quera-Bofarull A, Gidraf Kahindo Maina A, Valentijn T, Harlass S, Krauss F, Huang C, Moreno Jimenez R, Comes T, Gaanderse M, Milano L, Luengo-Oroz M. Epidemiological modelling in refugee and internally displaced people settlements: challenges and ways forward. BMJ Glob Health. 2022 Mar;7(3):e007822. https://doi.org/10.1136/bmjgh-2021-007822
1205. Chanda SK, Kabir MR, Roy T, Shohel TA, Howlader MH, Razu SR. Sociocultural Costs of the Long-term COVID-19 Outbreak in Bangladesh: A Systematic Review. Sage Open. 2022 Dec 20;12(4):21582440221143298. eCollection 2022 Oct-Dec. https://doi.org/10.1177/21582440221143298
1206. Al Janabi T, Petrillo G, Chung S, Pino M. Predictors of Vaccine Uptake among Migrants in the United States: A Rapid Systematic Review. Epidemiologia (Basel). 2022 Oct 20;3(4):465-481. https://doi.org/10.3390/epidemiologia3040035
1207. Michaleas SN, Protogerou AD, Sipsas NV, Panayiotakopoulos G, Angelakis AC, Michailidou C, Karamanou M. The Anti-tuberculosis Battle in Greece in the 1800s and 1900s. Cureus. 2022 Jun 16;14(6):e26023. eCollection 2022 Jun. https://doi.org/10.7759/cureus.26023
1208. Elharake JA, Omer SB, Schwartz JL. Country immunization policies for refugees across 20 low-middle income and 20 high-income countries. Vaccine. 2022 Oct 6;40(42):6017-6022. Epub 2022 Sep 16. https://doi.org/10.1016/j.vaccine.2022.09.032
1209. Chatziprodromidou IP, Dimitrakopoulou ME, Apostolou T, Katopodi T, Charalambous E, Vantarakis A. Hepatitis A and E in the Mediterranean: A systematic review. Travel Med Infect Dis. 2022 May-Jun;47:102283. Epub 2022 Feb 26. https://doi.org/10.1016/j.tmaid.2022.102283

1210. Ismail SA, Lam ST, Bell S, Fouad FM, Blanchet K, Borghi J. Strengthening vaccination delivery system resilience in the context of protracted humanitarian crisis: a realist-informed systematic review. BMC Health Serv Res. 2022 Oct 23;22(1):1277. https://doi.org/10.1186/s12913-022-08653-4
1211. Helou M, Van Berlaer G, Yammine K. Factors influencing the occurrence of infectious disease outbreaks in Lebanon since the Syrian crisis. Pathog Glob Health. 2022 Feb;116(1):13-21. Epub 2021 Jul 27. https://doi.org/10.1080/20477724.2021.1957192
1212. Saleh M, Farah Z, Howard N. Infectious disease surveillance for refugees at borders and in destination countries: a scoping review. BMC Public Health. 2022 Feb 4;22(1):227. https://doi.org/10.1186/s12889-022-12646-7
1213. Meaza A, Tola HH, Eshetu K, Mindaye T, Medhin G, Gumi B. Tuberculosis among refugees and migrant populations: Systematic review. PLoS One. 2022 Jun 9;17(6):e0268696. eCollection 2022. https://doi.org/10.1371/journal.pone.0268696
1214. Martins TLS, Silva GRDCE, Silva CA, Gomes DO, Diniz E Silva BV, Carneiro MADS, Pacheco LR, Araujo NM, Zanchetta MS, Teles SA, Caetano KAA. Hepatitis B and C in Immigrants and Refugees in Central Brazil: Prevalence, Associated Factors, and Immunization. Viruses. 2022 Jul 14;14(7):1534. https://doi.org/10.3390/v14071534
1215. Mengesha Z, Alloun E, Weber D, Smith M, Harris P. "Lived the Pandemic Twice": A Scoping Review of the Unequal Impact of the COVID-19 Pandemic on Asylum Seekers and Undocumented Migrants. Int J Environ Res Public Health. 2022 May 29;19(11):6624. https://doi.org/10.3390/ijerph19116624
1216. Yusuff SI, Tajudeen YA, Oladunjoye IO, Oladipo HJ, Bolarinwa OV, Popoola OT, Ahmed AF, Olana MD. The need to increase antimicrobial resistance surveillance among forcibly displaced persons (FDPs). Trop Dis Travel Med Vaccines. 2023 Sep 1;9(1):12. https://doi.org/10.1186/s40794-023-00198-6
1217. Taha H, Durham J, Reid S. Communicable Diseases Prevalence among Refugees and Asylum Seekers: Systematic Review and Meta-Analysis. Infect Dis Rep. 2023 Mar 31;15(2):188-203. https://doi.org/10.3390/idr15020020
1218. Badanta B, González-Cano-Caballero M, Fernández-García E, Lucchetti G, de Diego-Cordero R. The consequences of the COVID-19 pandemic on the refugee population: a rapid review. Perspect Public Health. 2023 Jul;143(4):225-241. Epub 2022 Jun 1. https://doi.org/10.1177/17579139221093159
1219. Shearn C, Krockow EM. Reasons for COVID-19 vaccine hesitancy in ethnic minority groups: A systematic review and thematic synthesis of initial attitudes in qualitative research. SSM Qual Res Health. 2023 Jun;3:100210. Epub 2022 Dec 22. https://doi.org/10.1016/j.ssmqr.2022.100210
1220. Alimoradi Z, Sallam M, Jafari E, Potenza MN, Pakpour AH. Prevalence of COVID-19 vaccine acceptance among migrant and refugee groups: A systematic review and meta-analysis. Vaccine X. 2023 Aug;14:100308. Epub 2023 May 6. https://doi.org/10.1016/j.jvacx.2023.100308
1221. Saseetharran A, Hiebert L, Gupta N, Nyirahabihirwe F, Kamali I, Ward JW. Prevention, testing, and treatment interventions for hepatitis B and C in refugee populations: results of a scoping review. BMC Infect Dis. 2023 Dec 9;23(1):866. https://doi.org/10.1186/s12879-023-08861-1
1222. Hossain MS, Noman AA, Mamun SMAA, Mosabbir AA. Twenty-two years of dengue outbreaks in Bangladesh: epidemiology, clinical spectrum, serotypes, and future disease risks. Trop Med Health. 2023 Jul 11;51(1):37. https://doi.org/10.1186/s41182-023-00528-6

1223. Tang C, Camp P. Supporting the Respiratory Health of Migrants and Refugees. Clin Chest Med. 2023 Sep;44(3):605-612. Epub 2023 May 25. https://doi.org/10.1016/j.ccm.2023.03.013
1224. Haldar K, Alam MS, Koepfli C, Lobo NF, Phru CS, Islam MN, Faiz A, Khan WA, Haque R. Bangladesh in the era of malaria elimination. Trends Parasitol. 2023 Sep;39(9):760-773. Epub 2023 Jul 25. https://doi.org/10.1016/j.pt.2023.06.009
1225. Logie CH, Okumu M, Berry I, Hakiza R, Baral SD, Musoke DK, Nakitende A, Mwima S, Kyambadde P, Loutet M, Batte S, Lester R, Neema S, Newby K, Mbuagbaw L. Findings from the Tushirikiane mobile health (mHealth) HIV self-testing pragmatic trial with refugee adolescents and youth living in informal settlements in Kampala, Uganda. J Int AIDS Soc. 2023 Oct;26(10):e26185. https://doi.org/10.1002/jia2.26185
1226. Al-Eitan L, Alnemri M, Alkhawaldeh M, Mihyar A. Rodent-borne viruses in the region of Middle East. Rev Med Virol. 2023 Jul;33(4):e2440. Epub 2023 Mar 16. https://doi.org/10.1002/rmv.2440
1227. Fadlallah M, Salman A, Salem-Sokhn E. Updates on the Status of Carbapenem-Resistant Enterobacterales in Lebanon. Int J Microbiol. 2023 May 29;2023:8831804. eCollection 2023. https://doi.org/10.1155/2023/8831804
1228. Feldmeier H. Travel- and migration-associated epidermal parasitic skin diseases. A review. Travel Med Infect Dis. 2023 Oct 28:102655. Online ahead of print. https://doi.org/10.1016/j.tmaid.2023.102655
1229. Tehranchinia Z, Robati RM, Moravvej H, Memariani M, Memariani H. Monkeypox Disease with a Focus on the 2022 Outbreak; a Narrative Review. Arch Acad Emerg Med. 2023 Jan 16;11(1):e19. eCollection 2023. https://doi.org/10.22037/aaem.v11i1.1856
1230. Deng YP, Fu YT, Yao C, Shao R, Zhang XL, Duan DY, Liu GH. Emerging bacterial infectious diseases/pathogens vectored by human lice. Travel Med Infect Dis. 2023 Sep-Oct;55:102630. Epub 2023 Aug 9. https://doi.org/10.1016/j.tmaid.2023.102630
1231. Khan AI, Islam MT, Khan ZH, Tanvir NA, Amin MA, Khan II, Bhuiyan ATMRH, Hasan ASMM, Islam MS, Bari TIA, Rahman A, Islam MN, Qadri F. Implementation and Delivery of Oral Cholera Vaccination Campaigns in Humanitarian Crisis Settings among Rohingya Myanmar nationals in Cox's Bazar, Bangladesh. Vaccines (Basel). 2023 Apr 14;11(4):843. https://doi.org/10.3390/vaccines11040843
1232. Gabaldón Figueira JC, Wagah MG, Adipo LB, Wanjiku C, Maia MF. Topical repellents for malaria prevention. Cochrane Database Syst Rev. 2023 Aug 21;8(8):CD015422. https://doi.org/10.1002/14651858.CD015422.pub2
1233. Melis T, Sahle T, Haile K, Timerga A, Zewdie A, Wegu Y, Zepire K, Bedewi J. Providing anti-retroviral treatment did not achieve the ambition of 'Joint united nations program on HIV/AIDS (UNAIDS) among HIV positive patient in Ethiopia': a systematic review and meta-analysis. J Pharm Policy Pract. 2023 Dec 27;17(1):2290672. eCollection 2024. https://doi.org/10.1080/20523211.2023.2290672
1234. Hitch L, Masoud D, Hobbs LA, Moujabber M, Cravero K. The vulnerability to COVID-19 of migrants in large urban areas: structural exacerbators and community-level mitigators. Eur J Public Health. 2023 Aug 1;33(4):704-716. https://doi.org/10.1093/eurpub/ckad076
1235. Oakley R, Hedrich N, Walker A, Dinkita HM, Tschopp R, Abongomera C, Paris DH. Status of zoonotic disease research in refugees, asylum seekers and internally displaced people, globally: A scoping review of forty clinically important zoonotic pathogens. PLoS Negl Trop Dis. 2024 May 20;18(5):e0012164. eCollection 2024 May. https://doi.org/10.1371/journal.pntd.0012164

1236. Bianchi FP, Fiacchini D, Frisicale EM, Gili R, Greco S, Guicciardi S, Riccò M, Zichichi S, Zotti N, Tafuri S. Infectious risk profile and strategies for prevention and control of outbreaks in refugee, asylum seekers and migrant populations in EU/EEA countries: a systematic narrative review of evidence. Ann Ig. 2024 Sep-Oct;36(5):549-568. Epub 2024 Mar 28. https://doi.org/10.7416/ai.2024.2610
1237. Thompson N, Kyaw KWY, Singh L, Cikomola JC, Singh NS, Roberts B. The effect of COVID-19 on the non-COVID health outcomes of crisis-affected peoples: a systematic review. Confl Health. 2024 Apr 25;18(1):37. https://doi.org/10.1186/s13031-024-00592-7
1238. Jaber T, Boelee E, Bleser J, Bartram JK. Outbreaks of faecal-orally transmitted diseases in displacement camps: A scoping review of pathogens, risk factors, exposure routes, and drivers of transmission. Glob Public Health. 2024 Jan;19(1):2380847. Epub 2024 Jul 25. https://doi.org/10.1080/17441692.2024.2380847
1239. Führer A, Pacolli-Tabaku L, Kompa P, Yılmaz-Aslan Y, Brzoska P. [Management Of Covid-19 Pandemic In Shelters For Asylum Seekers: Results From A Mixed Methods Study.]. Gesundheitswesen. 2024 Apr;86(4):315-321. Epub 2023 Oct 10. https://doi.org/10.1055/a-2144-5841
1240. Agaba B, Akunzirwe R, Baliruno LN, Naiga HN, Okello P, Kadobera D, Bulage L, Migisha R, Ario AR. Investigation of a bacterial meningitis cluster in a refugee settlement, Obongi District, Uganda, March 2023. Pan Afr Med J. 2024 Jan 10;47:11. eCollection 2024. https://doi.org/10.11604/pamj.2024.47.11.42377
1241. Ope M, Musyoka R, Kiogora J, Wambugu J, Hunsperger E, Emukule GO, Munyua P, Juma B, Simiyu E, Gagnidze L, Burton J, Eidex RB. Epidemiology of SARS-CoV-2 in Kakuma Refugee Camp Complex, Kenya, 2020-2021(1). Emerg Infect Dis. 2024 May;30(5):900-907. https://doi.org/10.3201/eid3005.231042
1242. Graci D, Piazza N, Ardagna S, Casuccio A, Drobov A, Geraci F, Immordino A, Pirrello A, Restivo V, Rumbo R, Stefano R, Virone R, Zarcone E, Immordino P. Barriers to and Facilitators for Accessing HPV Vaccination in Migrant and Refugee Populations: A Systematic Review. Vaccines (Basel). 2024 Feb 29;12(3):256. https://doi.org/10.3390/vaccines12030256
1243. Riccò M, Corrado S, Bottazzoli M, Marchesi F, Gili R, Bianchi FP, Frisicale EM, Guicciardi S, Fiacchini D, Tafuri S. RSV Infection in Refugees and Asylum Seekers: A Systematic Review and Meta-Analysis. Epidemiologia (Basel). 2024 May 27;5(2):221-249. https://doi.org/10.3390/epidemiologia5020016
1244. Winters M, Sochoń-Latuszek A, Nurzhynska A, Yoruk K, Kukuła K, Bahruddinov M, Kusek A, Kleszczewska D, Dzielska A, Maciejewski T, Mazur J, Melchinger H, Kinsman J, Kramarz P, Christie S, Omer SB. "Vaccinating your child during an emergency is more important than ever": a randomised controlled trial on message framing among Ukrainian refugees in Poland, 2023. Euro Surveill. 2024 Sep;29(39):2400159. https://doi.org/10.2807/1560-7917.ES.2024.29.39.2400159
1245. Anstead GM. The Maturation of the International Health Crisis Response: The Polish Typhus Epidemic of 1916-1923 Compared to the African Ebola Virus Disease Epidemic of 2013-2016: Part I, the Polish Epidemic. Epidemiologia (Basel). 2024 Dec 9;5(4):728-769. https://doi.org/10.3390/epidemiologia5040051
1246. Cernigliaro A, Giorgi Rossi P, Di Napoli A, Milli C, Petrelli A, Scondotto S, D'Amato S, Mondello S. [Reducing the impact of COVID-19 in immigrants: a systematic review of the efficacy of interventions]. Epidemiol Prev. 2024 Jul-Oct;48(4-5):75-84. https://doi.org/10.19191/EP24.4-5.S1.116

1247. Strahan AG, Elston DM. Dermatologic Care for Refugees: Effective Management of Scabies and Pediculosis. Cutis. 2024 Apr;113(4):E16-E21. https://doi.org/10.12788/cutis.0999
1248. Pradhan SK, Panda A, Debata I, Panda PS. Seroprevalence of Measles Antibodies Among Migrant Populations: A Systematic Review and Meta-Analysis. Cureus. 2024 Nov 22;16(11):e74243. eCollection 2024 Nov. https://doi.org/10.7759/cureus.74243
1249. Marinho TA, Okita MT, Guimarães RA, Zara ALSA, Caetano KAA, Teles SA, de Matos MAD, Carneiro MADS, Martins RMB. The Global Prevalence of HTLV-1 and HTLV-2 Infections among Immigrants and Refugees-A Systematic Review and Meta-Analysis. Viruses. 2024 Sep 27;16(10):1526. https://doi.org/10.3390/v16101526
1250. Li K, Thaweesee N, Kimmel A, Dorward E, Dam A. Barriers and facilitators to utilizing HIV prevention and treatment services among migrant youth globally: A scoping review. PLOS Glob Public Health. 2024 Feb 14;4(2):e0002851. eCollection 2024. https://doi.org/10.1371/journal.pgph.0002851
1251. Hinton SM. Preventing Vertical Transmission of Chagas Disease: An Emerging Public Health Issue in Perinatal Care. J Midwifery Womens Health. 2024 Nov-Dec;69(6):888-896. Epub 2024 Aug 26. https://doi.org/10.1111/jmwh.13682
1252. Msellemu D, Tanner M, Yadav R, Moore SJ. Occupational exposure to malaria, leishmaniasis and arbovirus vectors in endemic regions: A systematic review. Curr Res Parasitol Vector Borne Dis. 2024 Jun 1;6:100185. eCollection 2024. https://doi.org/10.1016/j.crpvbd.2024.100185
1253. Kebede HK, Gesesew HA, Gebremedhin AT, Ward P. The impact of armed conflicts on HIV treatment outcomes in Sub-Saharan Africa: a systematic review and meta-analysis. Confl Health. 2024 May 17;18(1):40. https://doi.org/10.1186/s13031-024-00591-8
1254. Chukwudile B, Pan D, Silva L, Gogoi M, Al-Oraibi A, Bird P, George N, Thompson HA, Baggaley RF, Hargreaves S, Pareek M, Nellums LB. Antimicrobial resistance among migrants in Europe: a systematic review and meta-analysis - update from 2017 to 2023. EClinicalMedicine. 2024 Sep 5;75:102801. eCollection 2024 Sep. https://doi.org/10.1016/j.eclinm.2024.102801
1255. Saif-Ur-Rahman KM, Mamun R, Hasan M, Meiring JE, Khan MA. Oral killed cholera vaccines for preventing cholera. Cochrane Database Syst Rev. 2024 Jan 10;1(1):CD014573. https://doi.org/10.1002/14651858.CD014573
1256. Volkman T, Muruganandah V, Graham H, Tosif S, Stokes S, Ranganathan S. QuantiFERON Gold-In-Tube for the diagnosis of mycobacterial tuberculosis infection in children under 5 years of age: A systematic review and meta-analysis. PLoS One. 2024 Jan 2;19(1):e0295913. eCollection 2024. https://doi.org/10.1371/journal.pone.0295913
1257. Hintermeier M, Gottlieb N, Rohleder S, Oppenberg J, Baroudi M, Pernitez-Agan S, Lopez J, Flores S, Mohsenpour A, Wickramage K, Bozorgmehr K. COVID-19 among migrants, refugees, and internally displaced persons: systematic review, meta-analysis and qualitative synthesis of the global empirical literature. EClinicalMedicine. 2024 Jul 10;74:102698. eCollection 2024 Aug. https://doi.org/10.1016/j.eclinm.2024.102698
1258. Garfein RS, Liu L, Cepeda J, Graves S, San Miguel S, Antonio A, Cuevas-Mota J, Mercer V, Miller M, Catanzaro DG, Rios P, Raab F, Benson CA. Asynchronous Video Directly Observed Therapy to Monitor Short-Course Latent Tuberculosis Infection Treatment: Results of a Randomized Controlled Trial. Open Forum Infect Dis. 2024 Mar 26;11(4):ofae180. eCollection 2024 Apr. https://doi.org/10.1093/ofid/ofae180

1259. Hassan IN, Abuassa N, Ibrahim M. The Sudan conflict: A catalyst for the spread of infectious diseases in displaced populations. Int J Infect Dis. 2025 Feb;151:107326. Epub 2024 Dec 6. https://doi.org/10.1016/j.ijid.2024.107326
1260. Igbokwe V, Otto-Knapp R, Breuer C, Priwitzer M, Bauer T, Häcker B. [Tuberculosis among Ukrainian Refugees in Germany - A Comparison of Screening and Reporting Data]. Gesundheitswesen. 2025 Feb;87(2):99-102. Epub 2024 Jun 28. https://doi.org/10.1055/a-2312-6270
1261. Beiras CG, Malembi E, Escrig-Sarreta R, Ahuka S, Mbala P, Mavoko HM, Subissi L, Abecasis AB, Marks M, Mitjà O. Concurrent outbreaks of mpox in Africa-an update. Lancet. 2025 Jan 4;405(10472):86-96. Epub 2024 Dec 12. https://doi.org/10.1016/S0140-6736(24)02353-5
1262. Müller M, Khamis D, Srivastava D, Exadaktylos AK, Pfortmueller CA. Understanding Refugees' Health. Semin Neurol. 2018 Apr;38(2):152-162. Epub 2018 May 23. https://doi.org/10.1055/s-0038-1649337
1263. Tourapi C, Tsioutis C. Circular Policy: A New Approach to Vector and Vector-Borne Diseases' Management in Line with the Global Vector Control Response (2017-2030). Trop Med Infect Dis. 2022 Jul 4;7(7):125. https://doi.org/10.3390/tropicalmed7070125
1264. Salih A, Mohamed M. A case analysis of a mass treatment approach to control GI and water-related conditions in Sudan. BMC Public Health. 2021 Nov 17;21(1):2111. https://doi.org/10.1186/s12889-021-12154-0
1265. Oelmeier de Murcia K, Glatz B, Willems S, Kossow A, Strobel M, Stühmer B, Schaumburg F, Mellmann A, Kipp F, Schmitz R, Möllers M. Prevalence of Multidrug Resistant Bacteria in Refugees: A Prospective Case Control Study in an Obstetric Cohort. Z Geburtshilfe Neonatol. 2017 Jun;221(3):132-136. Epub 2017 Jun 30. https://doi.org/10.1055/s-0043-102579
1266. Singer K, Schulze-Sturm U, Alba-Alejandre I, Hollwitz B, Nguyen TTT, Sollinger F, Eberle J, Hübner J, Kobbe R, Genzel-Boroviczény O, von Both U. Impact of refugee influx on the epidemiology of late-presenting HIV-infected pregnant women and mother-to-child transmission: comparing a southern and northern medical centre in Germany. Infection. 2019 Oct;47(5):847-852. Epub 2019 Jun 12. https://doi.org/10.1007/s15010-019-01332-3
1267. Rowland M, Durrani N, Kenward M, Mohammed N, Urahman H, Hewitt S. Control of malaria in Pakistan by applying deltamethrin insecticide to cattle: a community-randomised trial. Lancet. 2001 Jun 9;357(9271):1837-41. https://doi.org/10.1016/S0140-6736(00)04955-2
1268. Bizri NA, Alam W, Khoury M, Musharrafieh U, Ghosn N, Berri A, Bizri AR. The Association Between the Syrian Crisis and Cutaneous Leishmaniasis in Lebanon. Acta Parasitol. 2021 Dec;66(4):1240-1245. Epub 2021 Apr 22. https://doi.org/10.1007/s11686-021-00395-3
1269. El Tatary G, Gill N. The impact of the COVID-19 pandemic on the mental health and wellbeing of refugees and asylum seekers-A Narrative Review of the Literature. Australas Psychiatry. 2022 Dec;30(6):728-731. Epub 2022 Oct 12. https://doi.org/10.1177/10398562221131154
1270. Salameh G, Marais D, Khoury R. Impact of COVID-19 Pandemic on Mental Health among the Population in Jordan. Int J Environ Res Public Health. 2023 Jul 17;20(14):6382. https://doi.org/10.3390/ijerph20146382
1271. Bal S, Duckles A, Buttenheim A. Visual Health and Visual Healthcare Access in Refugees and Displaced Persons: A Systematic Review. J Immigr Minor Health. 2019 Feb;21(1):161-174. https://doi.org/10.1007/s10903-018-0766-x

1272. Eiset AH, Aoun MP, Haddad RS, Naja WJ, Fuursted K, Nielsen HV, Stensvold CR, Nielsen MS, Gottlieb A, Frydenberg M, Wejse C. Asylum seekers' and Refugees' Changing Health (ARCH) study protocol: an observational study in Lebanon and Denmark to assess health implications of long-distance migration on communicable and non-communicable diseases and mental health. BMJ Open. 2020 May 26;10(5):e034412. https://doi.org/10.1136/bmjopen-2019-034412
1273. Jazwa A, Coleman MS, Gazmararian J, Wingate LT, Maskery B, Mitchell T, Weinberg M. Cost-benefit comparison of two proposed overseas programs for reducing chronic Hepatitis B infection among refugees: is screening essential?. Vaccine. 2015 Mar 10;33(11):1393-9. Epub 2015 Jan 14. https://doi.org/10.1016/j.vaccine.2015.01.010
1274. Hvass AMF, Wejse C. Systematic health screening of refugees after resettlement in recipient countries: a scoping review. Ann Hum Biol. 2017 Aug;44(5):475-483. Epub 2017 May 31. https://doi.org/10.1080/03014460.2017.1330897
1275. Hermans MPJ, Kooistra J, Cannegieter SC, Rosendaal FR, Mook-Kanamori DO, Nemeth B. Healthcare and disease burden among refugees in long-stay refugee camps at Lesbos, Greece. Eur J Epidemiol. 2017 Sep;32(9):851-854. Epub 2017 Jun 8. https://doi.org/10.1007/s10654-017-0269-4
1276. Sidhu A, Kakkar R, Alenezi O. The Management of Newly Diagnosed HIV in a Sudanese Refugee in Canada: Commentary and Review of Literature. Rev Recent Clin Trials. 2019;14(1):61-65. https://doi.org/10.2174/1574887113666180903145323
1277. Nazareth J, Baggaley RF, Divall P, Pan D, Martin CA, Volik M, Nellums LB, Pareek M. What is the evidence on existing national policies and guidelines for delivering effective tuberculosis, HIV and viral hepatitis services for refugees and migrants among Member States of the WHO European Region?. Copenhagen: WHO Regional Office for Europe; 2021.
1278. Nightingale S, Stormon MO, Day AS, Webber MT, Ward KA, O'Loughlin EV. Chronic hepatitis B and C infection in children in New South Wales. Med J Aust. 2009 Jun 15;190(12):670-3. https://doi.org/10.5694/j.1326-5377.2009.tb02633.x
1279. Lucas M, Nicol P, McKinnon E, Whidborne R, Lucas A, Thambiran A, Burgner D, Waring J, French M. A prospective large-scale study of methods for the detection of latent Mycobacterium tuberculosis infection in refugee children. Thorax. 2010 May;65(5):442-8. https://doi.org/10.1136/thx.2009.127555
1280. Goldwater PN. Iatrogenic blood-borne viral infections in refugee children from war and transition zones. Emerg Infect Dis. 2013 Jun;19(6):892-8. https://doi.org/10.3201/eid1906.120806
1281. Dang K, Tribble AC. Strategies in infectious disease prevention and management among US-bound refugee children. Curr Probl Pediatr Adolesc Health Care. 2014 Aug;44(7):196-207. Epub 2014 Jun 25. https://doi.org/10.1016/j.cppeds.2014.03.004
1282. Marais BJ. Tuberculosis in children. J Paediatr Child Health. 2014 Oct;50(10):759-67. Epub 2014 Feb 19. https://doi.org/10.1111/jpc.12503
1283. Maltezou HC, Elhadad D, Glikman D. Monitoring and managing antibiotic resistance in refugee children. Expert Rev Anti Infect Ther. 2017 Nov;15(11):1015-1025. Epub 2017 Oct 23. https://doi.org/10.1080/14787210.2017.1392853
1284. Elliot C, Marais B, Williams P, Joshua P, Towle S, Hart G, Zwi K. Tuberculin skin test versus interferon-gamma release assay in refugee children: A retrospective cohort study. J Paediatr Child Health. 2018 Aug;54(8):834-839. Epub 2018 Feb 14. https://doi.org/10.1111/jpc.13865
1285. Shetty AK. Infectious Diseases among Refugee Children. Children (Basel). 2019 Nov 27;6(12):129. https://doi.org/10.3390/children6120129

1286. Condemi F, Rossi G, Lupiz M, Pagano A, Zamatto F, Marini S, Romeo F, De Maio G. Screening of asymptomatic rheumatic heart disease among refugee/migrant children and youths in Italy. Pediatr Rheumatol Online J. 2019 Apr 2;17(1):12. https://doi.org/10.1186/s12969-019-0314-9

1287. Ghosh S, Dronavalli M, Raman S. Tuberculosis infection in under-2-year-old refugees: Should we be screening? A systematic review and meta-regression analysis. J Paediatr Child Health. 2020 Apr;56(4):622-629. Epub 2019 Dec 28. https://doi.org/10.1111/jpc.14701

1288. Charania NA, Paynter J, Lee AC, Watson DG, Turner NM. Vaccine-Preventable Disease-Associated Hospitalisations Among Migrant and Non-migrant Children in New Zealand. J Immigr Minor Health. 2020 Apr;22(2):223-231. https://doi.org/10.1007/s10903-019-00888-4

1289. Sack DA, Debes AK, Ateudjieu J, Bwire G, Ali M, Ngwa MC, Mwaba J, Chilengi R, Orach CC, Boru W, Mohamed AA, Ram M, George CM, Stine OC. Contrasting Epidemiology of Cholera in Bangladesh and Africa. J Infect Dis. 2021 Dec 20;224(12 Suppl 2):S701-S709. https://doi.org/10.1093/infdis/jiab440

1290. Graber KM, Byrne EM, Goodacre EJ, Kirby N, Kulkarni K, O'Farrelly C, Ramchandani PG. A rapid review of the impact of quarantine and restricted environments on children's play and the role of play in children's health. Child Care Health Dev. 2021 Mar;47(2):143-153. Epub 2020 Dec 7. https://doi.org/10.1111/cch.12832

1291. Pfeil J; DGPI; Assaad K; BVÖGD; von Both U; DAKJ/Bündnis Kinder- und Jugendgesundheit; Janda A, Kitz C, Kobbe R; GTP; Kunze M; DGGG; Lindert J; DGKCH; Ritz N; PIGS; Trapp S; BVKJ; Hufnagel M; DGKJ. [Updated recommendations on the treatment of infectious diseases in refugees in childhood and adolescence in Germany (situation as of 30 March 2022), registered as S1 guidelines (AWMF-Register Nr. 048-017)]. Monatsschr Kinderheilkd. 2022;170(7):632-647. Epub 2022 May 25. https://doi.org/10.1007/s00112-022-01499-4

1292. Volkman T, Clarke R, Anstey J, Cherian S. COVID-19 vaccination of children with refugee backgrounds in Western Australia: a retrospective observational study. Med J Aust. 2023 Dec 11;219(11):549-550. Epub 2023 Oct 24. https://doi.org/10.5694/mja2.52131

1293. Sana S, Fabbro E, Zovi A, Vitiello A, Ola-Ajayi T, Zahoui Z, Salami B, Sabbatucci M. Scoping Review on Barriers and Challenges to Pediatric Immunization Uptake among Migrants: Health Inequalities in Italy, 2003 to Mid-2023. Vaccines (Basel). 2023 Aug 25;11(9):1417. https://doi.org/10.3390/vaccines11091417

1294. Alhassan A, Ajala LS, Ode B, Alanjiro M, Rehman S, Onesime J, Kihanduka E, Tague C, Farhan K, Banga S, Rugendabanga E, Manga A, Mbwambo GJ, Hangi S, Rhugendabanga F, Mufungizi I, Furqan M, Rusho MA, Budair MM, Akilimali A; Medical Research Circle Collaborators. Call for elimination program of Malaria among children under 5 years old living in refugee camps in eastern Democratic Republic of Congo. New Microbes New Infect. 2024 Oct 11;62:101508. eCollection 2024 Dec. https://doi.org/10.1016/j.nmni.2024.101508

1295. Pach S, Ritz N, Eisen S. Optimizing Early Diagnosis of Infectious Diseases in Migrant and Refugee Children: An Overview of Best Practices and Strategies. Pediatr Infect Dis J. 2024 Aug 1;43(8):e278-e281. Epub 2024 May 31. https://doi.org/10.1097/INF.0000000000004406

1296. Krüger C, Schuler-Lüttmann S, Haug T, Gantert M, Hermsen M. Multidrug-Resistant Bacteria in Refugee Children and Pregnant Women Admitted to a General Hospital in North

Rhine-Westphalia, Germany. Klin Padiatr. 2016 Jul;228(4):227-9. Epub 2016 Jun 30. https://doi.org/10.1055/s-0042-109711
1297. Shanks L, Schull MJ. Rape in war: the humanitarian response. CMAJ. 2000 Oct 31;163(9):1152-6.
1298. Short RV. The future fertility of mankind: effects on world population growth and migration. Reprod Fertil Dev. 2001;13(5-6):405-10. https://doi.org/10.1071/rd01107
1299. Taylor VM, Jackson JC, Yasui Y, Kuniyuki A, Acorda E, Marchand A, Schwartz SM, Tu SP, Thompson B. Evaluation of an outreach intervention to promote cervical cancer screening among Cambodian American women. Cancer Detect Prev. 2002;26(4):320-7. https://doi.org/10.1016/s0361-090x(02)00055-7
1300. Abdulsalam AA, Bashour HN, Monem FS, Hamadeh FM. Pregnancy outcomes among Palestinian refugee women with sickle cell trait in Damascus, Syria. Saudi Med J. 2003 Sep;24(9):986-90.
1301. O'Heir J. Pregnancy and childbirth care following conflict and displacement: care for refugee women in low-resource settings. J Midwifery Womens Health. 2004 Jul-Aug;49(4 Suppl 1):14-8. https://doi.org/10.1016/j.jmwh.2004.04.031
1302. Momoh C. Female genital mutilation. Curr Opin Obstet Gynecol. 2004 Dec;16(6):477-80. https://doi.org/10.1097/00001703-200412000-00007
1303. Kaddour A, Hafez R, Zurayk H. Women's perceptions of reproductive health in three communities around Beirut, Lebanon. Reprod Health Matters. 2005 May;13(25):34-42. https://doi.org/10.1016/s0968-8080(05)25170-4
1304. Norredam M, Crosby S, Munarriz R, Piwowarczyk L, Grodin M. Urologic complications of sexual trauma among male survivors of torture. Urology. 2005 Jan;65(1):28-32. https://doi.org/10.1016/j.urology.2004.08.006
1305. Tober DM, Taghdisi MH, Jalali M. "Fewer children, better life" or "as many as God wants"? Family planning among low-income Iranian and Afghan refugee families in Isfahan, Iran. Med Anthropol Q. 2006 Mar;20(1):50-71. https://doi.org/10.1525/maq.2006.20.1.50
1306. O'Mahony J, Donnelly T. Immigrant and refugee women's post-partum depression help-seeking experiences and access to care: a review and analysis of the literature. J Psychiatr Ment Health Nurs. 2010 Dec;17(10):917-28. Epub 2010 Sep 2. https://doi.org/10.1111/j.1365-2850.2010.01625.x
1307. Carolan M. Pregnancy health status of sub-Saharan refugee women who have resettled in developed countries: a review of the literature. Midwifery. 2010 Aug;26(4):407-14. Epub 2009 Jan 1. https://doi.org/10.1016/j.midw.2008.11.002
1308. DeStephano CC, Flynn PM, Brost BC. Somali prenatal education video use in a United States obstetric clinic: a formative evaluation of acceptability. Patient Educ Couns. 2010 Oct;81(1):137-41. Epub 2010 Jan 13. https://doi.org/10.1016/j.pec.2009.12.003
1309. Badshah S, Mason L, Mckelvie K, Payne R, Lisboa PJ. Maternal risk factors in Afghan-refugees compared to Pakistani mothers in Peshawar, NWFP Pakistan. J Pak Med Assoc. 2011 Feb;61(2):161-4.
1310. Schmied V, Olley H, Burns E, Duff M, Dennis CL, Dahlen HG. Contradictions and conflict: a meta-ethnographic study of migrant women's experiences of breastfeeding in a new country. BMC Pregnancy Childbirth. 2012 Dec 27;12:163. https://doi.org/10.1186/1471-2393-12-163
1311. Asgary R, Emery E, Wong M. Systematic review of prevention and management strategies for the consequences of gender-based violence in refugee settings. Int Health. 2013 Jun;5(2):85-91. https://doi.org/10.1093/inthealth/iht009

1312. Gibson-Helm M, Teede H, Block A, Knight M, East C, Wallace EM, Boyle J. Maternal health and pregnancy outcomes among women of refugee background from African countries: a retrospective, observational study in Australia. BMC Pregnancy Childbirth. 2014 Nov 27;14:392. https://doi.org/10.1186/s12884-014-0392-0
1313. Balić D, Rizvanović M, Cizek-Sajko M, Balić A. Age at natural menopause in refugee and domicile women who lived in Tuzla Canton in Bosnia and Herzegovina during and after the war. Menopause. 2014 Jul;21(7):721-5. https://doi.org/10.1097/GME.0000000000000173
1314. Dennis CL, Gagnon A, Van Hulst A, Dougherty G. Predictors of breastfeeding exclusivity among migrant and Canadian-born women: results from a multi-centre study. Matern Child Nutr. 2014 Oct;10(4):527-44. Epub 2012 Sep 13. https://doi.org/10.1111/j.1740-8709.2012.00442.x
1315. Bouchghoul H, Hornez E, Duval-Arnould X, Philippe HJ, Nizard J. Humanitarian obstetric care for refugees of the Syrian war. The first 6 months of experience of Gynécologie Sans Frontières in Zaatari Refugee Camp (Jordan). Acta Obstet Gynecol Scand. 2015 Jul;94(7):755-759. Epub 2015 Apr 28. https://doi.org/10.1111/aogs.12638
1316. Gibson-Helm ME, Teede HJ, Cheng IH, Block AA, Knight M, East CE, Wallace EM, Boyle JA. Maternal health and pregnancy outcomes comparing migrant women born in humanitarian and nonhumanitarian source countries: a retrospective, observational study. Birth. 2015 Jun;42(2):116-24. Epub 2015 Apr 11. https://doi.org/10.1111/birt.12159
1317. Warren E, Post N, Hossain M, Blanchet K, Roberts B. Systematic review of the evidence on the effectiveness of sexual and reproductive health interventions in humanitarian crises. BMJ Open. 2015 Dec 18;5(12):e008226. https://doi.org/10.1136/bmjopen-2015-008226
1318. Kentoffio K, Berkowitz SA, Atlas SJ, Oo SA, Percac-Lima S. Use of maternal health services: comparing refugee, immigrant and US-born populations. Matern Child Health J. 2016 Dec;20(12):2494-2501. https://doi.org/10.1007/s10995-016-2072-3
1319. Nybo Andersen AM, Gundlund A, Villadsen SF. Stillbirth and congenital anomalies in migrants in Europe. Best Pract Res Clin Obstet Gynaecol. 2016 Apr;32:50-9. Epub 2015 Oct 19. https://doi.org/10.1016/j.bpobgyn.2015.09.004
1320. Guideline: Use of Multiple Micronutrient Powders for Point-of-Use Fortification of Foods Consumed by Pregnant Women. Geneva: World Health Organization; 2016.
1321. Merry L, Vangen S, Small R. Caesarean births among migrant women in high-income countries. Best Pract Res Clin Obstet Gynaecol. 2016 Apr;32:88-99. Epub 2015 Sep 14. https://doi.org/10.1016/j.bpobgyn.2015.09.002
1322. Ndlovu E, Bhala E. Menstrual hygiene - A salient hazard in rural schools: A case of Masvingo district of Zimbabwe. Jamba. 2016 Jan 13;8(2):204. eCollection 2016. https://doi.org/10.4102/jamba.v8i2.204
1323. Patel P, Dahab M, Tanabe M, Murphy A, Ettema L, Guy S, Roberts B. Tracking official development assistance for reproductive health in conflict-affected countries: 2002-2011. BJOG. 2016 Sep;123(10):1693-704. Epub 2016 Jan 28. https://doi.org/10.1111/1471-0528.13851
1324. Alnuaimi K, Kassab M, Ali R, Mohammad K, Shattnawi K. Pregnancy outcomes among Syrian refugee and Jordanian women: a comparative study. Int Nurs Rev. 2017 Dec;64(4):584-592. Epub 2017 May 25. https://doi.org/10.1111/inr.12382
1325. Anaman-Torgbor JA, King J, Correa-Velez I. Barriers and facilitators of cervical cancer screening practices among African immigrant women living in Brisbane, Australia.

Eur J Oncol Nurs. 2017 Dec;31:22-29. Epub 2017 Oct 13. https://doi.org/10.1016/j.ejon.2017.09.005
1326. Erenel H, Aydogan Mathyk B, Sal V, Ayhan I, Karatas S, Koc Bebek A. Clinical characteristics and pregnancy outcomes of Syrian refugees: a case-control study in a tertiary care hospital in Istanbul, Turkey. Arch Gynecol Obstet. 2017 Jan;295(1):45-50. Epub 2016 Sep 2. https://doi.org/10.1007/s00404-016-4188-5
1327. Khanlou N, Haque N, Skinner A, Mantini A, Kurtz Landy C. Scoping Review on Maternal Health among Immigrant and Refugee Women in Canada: Prenatal, Intrapartum, and Postnatal Care. J Pregnancy. 2017;2017:8783294. Epub 2017 Jan 22. https://doi.org/10.1155/2017/8783294
1328. Ackerson K, Zielinski R. Factors influencing use of family planning in women living in crisis affected areas of Sub-Saharan Africa: A review of the literature. Midwifery. 2017 Nov;54:35-60. Epub 2017 Aug 1. https://doi.org/10.1016/j.midw.2017.07.021
1329. Vigod SN, Bagadia AJ, Hussain-Shamsy N, Fung K, Sultana A, Dennis CE. Postpartum mental health of immigrant mothers by region of origin, time since immigration, and refugee status: a population-based study. Arch Womens Ment Health. 2017 Jun;20(3):439-447. Epub 2017 Apr 4. https://doi.org/10.1007/s00737-017-0721-1
1330. Marsh N, Scheele D, Feinstein JS, Gerhardt H, Strang S, Maier W, Hurlemann R. Oxytocin-enforced norm compliance reduces xenophobic outgroup rejection. Proc Natl Acad Sci U S A. 2017 Aug 29;114(35):9314-9319. Epub 2017 Aug 14. https://doi.org/10.1073/pnas.1705853114
1331. Winn A, Hetherington E, Tough S. Systematic Review of Immigrant Women's Experiences With Perinatal Care in North America. J Obstet Gynecol Neonatal Nurs. 2017 Sep-Oct;46(5):764-775. Epub 2017 Jun 28. https://doi.org/10.1016/j.jogn.2017.05.002
1332. Fellmeth G, Fazel M, Plugge E. Migration and perinatal mental health in women from low- and middle-income countries: a systematic review and meta-analysis. BJOG. 2017 Apr;124(5):742-752. Epub 2016 Jun 20. https://doi.org/10.1111/1471-0528.14184
1333. Aubrey C, Chari R, Mitchell BFP, Mumtaz Z. Gender of Provider-Barrier to Immigrant Women's Obstetrical Care: A Narrative Review. J Obstet Gynaecol Can. 2017 Jul;39(7):567-577. https://doi.org/10.1016/j.jogc.2017.01.013
1334. Demirci H, Yildirim Topak N, Ocakoglu G, Karakulak Gomleksiz M, Ustunyurt E, Ulku Turker A. Birth characteristics of Syrian refugees and Turkish citizens in Turkey in 2015. Int J Gynaecol Obstet. 2017 Apr;137(1):63-66. Epub 2017 Jan 10. https://doi.org/10.1002/ijgo.12088
1335. Robbers GML, Morgan A. Programme potential for the prevention of and response to sexual violence among female refugees: a literature review. Reprod Health Matters. 2017 Nov;25(51):69-89. Epub 2017 Dec 7. https://doi.org/10.1080/09688080.2017.1401893
1336. Varol N, Hall JJ, Black K, Turkmani S, Dawson A. Evidence-based policy responses to strengthen health, community and legislative systems that care for women in Australia with female genital mutilation / cutting. Reprod Health. 2017 May 18;14(1):63. https://doi.org/10.1186/s12978-017-0324-3
1337. Hutchinson A, Waterhouse P, March-McDonald J, Neal S, Ingham R. Understanding processes of risk and protection that shape the sexual and reproductive health of young women affected by conflict: the price of protection. Confl Health. 2017 Aug 17;11:15. eCollection 2017. https://doi.org/10.1186/s13031-017-0117-x
1338. Sami S, Kerber K, Kenyi S, Amsalu R, Tomczyk B, Jackson D, Dimiti A, Scudder E, Meyers J, Umurungi JPC, Kenneth K, Mullany LC. State of newborn care in South Sudan's

displacement camps: a descriptive study of facility-based deliveries. Reprod Health. 2017 Nov 29;14(1):161. https://doi.org/10.1186/s12978-017-0417-z
1339. Steenkamp M, Boyle J, Kildea S, Moore V, Davies M, Rumbold A. Perinatal outcomes among young Indigenous Australian mothers: A cross-sectional study and comparison with adult Indigenous mothers. Birth. 2017 Sep;44(3):262-271. Epub 2017 Apr 22. https://doi.org/10.1111/birt.12283
1340. Ozel S, Yaman S, Kansu-Celik H, Hancerliogullari N, Balci N, Engin-Ustun Y. Obstetric Outcomes among Syrian Refugees: A Comparative Study at a Tertiary Care Maternity Hospital in Turkey. Rev Bras Ginecol Obstet. 2018 Nov;40(11):673-679. Epub 2018 Oct 11. https://doi.org/10.1055/s-0038-1673427
1341. Ivanova O, Rai M, Kemigisha E. A Systematic Review of Sexual and Reproductive Health Knowledge, Experiences and Access to Services among Refugee, Migrant and Displaced Girls and Young Women in Africa. Int J Environ Res Public Health. 2018 Jul 26;15(8):1583. https://doi.org/10.3390/ijerph15081583
1342. Dehghan R. The health impact of (sexual) torture amongst Afghan, Iranian and Kurdish refugees: A literature review. Torture. 2018;28(3):77-91. https://doi.org/10.7146/torture.v28i3.111194
1343. Baillot H, Murray N, Connelly E, Howard N. Addressing female genital mutilation in Europe: a scoping review of approaches to participation, prevention, protection, and provision of services. Int J Equity Health. 2018 Feb 8;17(1):21. https://doi.org/10.1186/s12939-017-0713-9
1344. Parmar PK, Jin RO, Walsh M, Scott J. Mortality in Rohingya refugee camps in Bangladesh: historical, social, and political context. Sex Reprod Health Matters. 2019 May;27(2):1610275. https://doi.org/10.1080/26410397.2019.1610275
1345. Agbemenu K, Auerbach S, Murshid NS, Shelton J, Amutah-Onukagha N. Reproductive Health Outcomes in African Refugee Women: A Comparative Study. J Womens Health (Larchmt). 2019 Jun;28(6):785-793. Epub 2019 Feb 15. https://doi.org/10.1089/jwh.2018.7314
1346. Yasmine R, Sukkar B. Restrained motherhood: the Lebanese state in times of changing demographics and moral values. Sex Reprod Health Matters. 2019 May;27(2):1-12. https://doi.org/10.1080/26410397.2019.1643192
1347. Kanmaz AG, İnan AH, Beyan E, Özgür S, Budak A. Obstetric Outcomes of Syrian Refugees and Turkish Citizens. Arch Iran Med. 2019 Sep 1;22(9):482-488.
1348. Hirani SAA, Richter S, Salami BO, Vallianatos H. Breastfeeding in Disaster Relief Camps: An Integrative Review of Literature. ANS Adv Nurs Sci. 2019 Apr/Jun;42(2):E1-E12. https://doi.org/10.1097/ANS.0000000000000231
1349. Tantet C, Delaporte C, Cordel H. [Violence against migrant and refugee women: how to identify them]. Rev Prat. 2019 Jun;69(6):676-678.
1350. Siddiq H, Alemi Q, Mentes J, Pavlish C, Lee E. Preventive Cancer Screening Among Resettled Refugee Women from Muslim-Majority Countries: A Systematic Review. J Immigr Minor Health. 2020 Oct;22(5):1067-1093. https://doi.org/10.1007/s10903-019-00967-6
1351. Leppälä S, Lamminpää R, Gissler M, Vehviläinen-Julkunen K. Humanitarian migrant women's experiences of maternity care in Nordic countries: A systematic integrative review of qualitative research. Midwifery. 2020 Jan;80:102572. Epub 2019 Oct 31. https://doi.org/10.1016/j.midw.2019.102572
1352. Bayram Değer V, Ertem M, Çifçi S. Comparison of the Breastfeeding Practices of Refugee Syrian Mothers and Native Turkish Mothers. Breastfeed Med. 2020 Mar;15(3):170-175. Epub 2020 Jan 24. https://doi.org/10.1089/bfm.2019.0233

1353. Turkay Ü, Aydın Ü, Salıcı M, Çalışkan E, Terzi H, Astepe BS, Varlıklı O. Comparison of pregnant Turkish women and Syrian refugees: Does living as a refugee have an unfavorable effect on pregnancy outcomes?. Int J Gynaecol Obstet. 2020 May;149(2):160-165. Epub 2020 Mar 6. https://doi.org/10.1002/ijgo.13117
1354. Hawkey AJ, Ussher JM, Perz J. "I Treat My Daughters Not Like My Mother Treated Me": Migrant and Refugee Women's Constructions and Experiences of Menarche and Menstruation. 2020 Jul 25. In: Bobel C, Winkler IT, Fahs B, Hasson KA, Kissling EA, Roberts TA, editors. The Palgrave Handbook of Critical Menstruation Studies [Internet]. Singapore: Palgrave Macmillan; 2020. Chapter 10.
1355. Çöl M, Bilgili Aykut N, Usturalı Mut AN, Koçak C, Uzun SU, Akın A, Say L, Kobeissi L. Sexual and reproductive health of Syrian refugee women in Turkey: a scoping review within the framework of the MISP objectives. Reprod Health. 2020 Jun 22;17(1):99. https://doi.org/10.1186/s12978-020-00948-1
1356. Tirado V, Chu J, Hanson C, Ekström AM, Kågesten A. Barriers and facilitators for the sexual and reproductive health and rights of young people in refugee contexts globally: A scoping review. PLoS One. 2020 Jul 20;15(7):e0236316. eCollection 2020. https://doi.org/10.1371/journal.pone.0236316
1357. Amiri M, El-Mowafi IM, Chahien T, Yousef H, Kobeissi LH. An overview of the sexual and reproductive health status and service delivery among Syrian refugees in Jordan, nine years since the crisis: a systematic literature review. Reprod Health. 2020 Oct 28;17(1):166. https://doi.org/10.1186/s12978-020-01005-7
1358. Kiyak H, Gezer S, Ozdemir C, Gunkaya S, Karacan T, Gedikbasi A. Comparison of delivery characteristics and early obstetric outcomes between Turkish women and Syrian refugee pregnancies. Niger J Clin Pract. 2020 Jan;23(1):12-17. https://doi.org/10.4103/njcp.njcp_10_18
1359. Tellier M, Farley A, Jahangir A, Nakalema S, Nalunga D, Tellier S. Practice Note: Menstrual Health Management in Humanitarian Settings. 2020 Jul 25. In: Bobel C, Winkler IT, Fahs B, Hasson KA, Kissling EA, Roberts TA, editors. The Palgrave Handbook of Critical Menstruation Studies [Internet]. Singapore: Palgrave Macmillan; 2020. Chapter 45.
1360. Gausman J, Othman A, Dababneh A, Hamad I, Dabobe M, Daas I, Langer A. Landscape analysis of family planning research, programmes and policies targeting young people in Jordan: stakeholder assessment and systematic review. East Mediterr Health J. 2020 Sep 24;26(9):1115-1134. https://doi.org/10.26719/emhj.20.018
1361. Dhair A, Abed Y. The association of types, intensities and frequencies of physical activity with primary infertility among females in Gaza Strip, Palestine: A case-control study. PLoS One. 2020 Oct 23;15(10):e0241043. eCollection 2020. https://doi.org/10.1371/journal.pone.0241043
1362. Sharif Mohamed F, Wild V, Earp BD, Johnson-Agbakwu C, Abdulcadir J. Clitoral Reconstruction After Female Genital Mutilation/Cutting: A Review of Surgical Techniques and Ethical Debate. J Sex Med. 2020 Mar;17(3):531-542. Epub 2020 Jan 10. https://doi.org/10.1016/j.jsxm.2019.12.004
1363. Nabulsi D, Abou Saad M, Ismail H, Doumit MAA, El-Jamil F, Kobeissi L, Fouad FM. Minimum initial service package (MISP) for sexual and reproductive health for women in a displacement setting: a narrative review on the Syrian refugee crisis in Lebanon. Reprod Health. 2021 Mar 8;18(1):58. https://doi.org/10.1186/s12978-021-01108-9
1364. Luft H, Perzan M, Mitchell R, Schmidt A. An integrative literature review of barriers and facilitators to cervical cancer screening among refugee women in the United States.

Health Care Women Int. 2021 Sep;42(7-9):992-1012. Epub 2020 Aug 19. https://doi.org/10.1080/07399332.2020.1803872
1365. Scott HM, Wallis N. Maternity care for refugees living in Greek refugee camps: What are the challenges to provision?. Birth. 2021 Mar;48(1):114-121. Epub 2020 Dec 22. https://doi.org/10.1111/birt.12522
1366. Maheen H, Chalmers K, Khaw S, McMichael C. Sexual and reproductive health service utilisation of adolescents and young people from migrant and refugee backgrounds in high-income settings: a qualitative evidence synthesis (QES). Sex Health. 2021 Sep;18(4):283-293. https://doi.org/10.1071/SH20112
1367. van Eijk AM, Jayasinghe N, Zulaika G, Mason L, Sivakami M, Unger HW, Phillips-Howard PA. Exploring menstrual products: A systematic review and meta-analysis of reusable menstrual pads for public health internationally. PLoS One. 2021 Sep 24;16(9):e0257610. eCollection 2021. https://doi.org/10.1371/journal.pone.0257610
1368. Huffstetler AN, Ramirez SI, Dalrymple SN, Mendez Miller MH. Women's Health and Gender-Specific Considerations. Prim Care. 2021 Mar;48(1):117-129. Epub 2020 Nov 26. https://doi.org/10.1016/j.pop.2020.09.008
1369. Egli-Gany D, Aftab W, Hawkes S, Abu-Raddad L, Buse K, Rabbani F, Low N, Onarheim K. The social and structural determinants of sexual and reproductive health and rights in migrants and refugees: a systematic review of reviews. East Mediterr Health J. 2021 Dec 28;27(12):1203-1213. https://doi.org/10.26719/emhj.20.101
1370. Tchuenkam LW, Mbonda AN, Tochie JN, Mbem-Ngos PP, Noah-Ndzie HG, Bang GA. Transvaginal strangulated bowel evisceration through uterine perforation due to unsafe abortion: a case report and literature review. BMC Womens Health. 2021 Mar 5;21(1):98. https://doi.org/10.1186/s12905-021-01247-y
1371. Rayment-Jones H, Dalrymple K, Harris J, Harden A, Parslow E, Georgi T, Sandall J. Project20: Does continuity of care and community-based antenatal care improve maternal and neonatal birth outcomes for women with social risk factors? A prospective, observational study. PLoS One. 2021 May 4;16(5):e0250947. eCollection 2021. https://doi.org/10.1371/journal.pone.0250947
1372. Billett H, Vazquez Corona M, Bohren MA. Women from migrant and refugee backgrounds' perceptions and experiences of the continuum of maternity care in Australia: A qualitative evidence synthesis. Women Birth. 2022 Jul;35(4):327-339. Epub 2021 Aug 21. https://doi.org/10.1016/j.wombi.2021.08.005
1373. Hossain MA, Dawson A. A Systematic review of sexual and reproductive health needs, experiences, access to services, and interventions among the rohingya and the afghan refugee women of reproductive age in Asia. WHO South East Asia J Public Health. 2022 Jan-Jun;11(1):42-53. https://doi.org/10.4103/WHO-SEAJPH.WHO-SEAJPH_144_21
1374. Khaw SM, Zahroh RI, O'Rourke K, Dearnley R, Homer C, Bohren MA. Community-based doulas for migrant and refugee women: a mixed-method systematic review and narrative synthesis. BMJ Glob Health. 2022 Jul;7(7):e009098. https://doi.org/10.1136/bmjgh-2022-009098
1375. Ozkaya M, Korukcu O, Aune I. Breastfeeding attitudes of refugee women from Syria and influencing factors: a study based on the transition theory. Perspect Public Health. 2022 Jan;142(1):46-55. Epub 2020 Nov 23. https://doi.org/10.1177/1757913920964520
1376. Agbemenu K, Mencia JJ, de Rosa C, Aidoo-Frimpong G, Ely G. Family Planning Research in African Immigrant and Refugee Women: A Scoping Review. J Transcult Nurs. 2022 May;33(3):416-426. Epub 2022 Feb 9. https://doi.org/10.1177/10436596211072891

1377. Jamaluddine Z, Paolucci G, Ballout G, Al-Fudoli H, Day LT, Seita A, Campbell OMR. Classifying caesarean section to understand rising rates among Palestinian refugees: results from 290,047 electronic medical records across five settings. BMC Pregnancy Childbirth. 2022 Dec 13;22(1):935. https://doi.org/10.1186/s12884-022-05264-z
1378. Nowshin N, Kapiriri L, Davison CM, Harms S, Kwagala B, Mutabazi MG, Niec A. Sexual and reproductive health and rights of "last mile" adolescents: a scoping review. Sex Reprod Health Matters. 2022 Dec;30(1):2077283. https://doi.org/10.1080/26410397.2022.2077283
1379. Kassam S, Butcher D, Marcellus L. Experiences of nurses caring for involuntary migrant maternal women: a qualitative systematic review. JBI Evid Synth. 2022 Nov 1;20(11):2609-2655. https://doi.org/10.11124/JBIES-21-00181
1380. Racine L, Isik Andsoy I. Barriers and Facilitators Influencing Arab Muslim Immigrant and Refugee Women's Breast Cancer Screening: A Narrative Review. J Transcult Nurs. 2022 Jul;33(4):542-549. Epub 2022 Apr 26. https://doi.org/10.1177/10436596221085301
1381. Agunwamba AA, Finney Rutten LJ, St Sauver JL, Agunwamba AO, Jacobson DJ, McGree ME, Njeru JW. Higher Rates of Cesarean Sections Found in Somali Immigrant Women in Minnesota. J Racial Ethn Health Disparities. 2022 Oct;9(5):1765-1774. Epub 2021 Jul 26. https://doi.org/10.1007/s40615-021-01113-5
1382. Sidze EM, Wekesah FM, Kisia L, Abajobir A. Inequalities in Access and Utilization of Maternal, Newborn and Child Health Services in sub-Saharan Africa: A Special Focus on Urban Settings. Matern Child Health J. 2022 Feb;26(2):250-279. Epub 2021 Oct 15. https://doi.org/10.1007/s10995-021-03250-z
1383. Elnakib S, Metzler J. A scoping review of FGM in humanitarian settings: an overlooked phenomenon with lifelong consequences. Confl Health. 2022 Sep 15;16(1):49. https://doi.org/10.1186/s13031-022-00479-5
1384. Salameh TN, Nyakeriga DB, Hall LA. Telehealth Care for Perinatal Depression in Immigrant and Refugee Women: A Scoping Review. Issues Ment Health Nurs. 2023 Dec;44(12):1216-1225. Epub 2024 Jan 5. https://doi.org/10.1080/01612840.2023.2258222
1385. Fink G, Abdulcadir J, Johnson-Agbakwu CE. Rates of Cervical Cancer Screening and Dysplasia Among Refugees in a Health Care Safety Net System. J Immigr Minor Health. 2023 Dec;25(6):1315-1322. Epub 2023 May 30. https://doi.org/10.1007/s10903-023-01491-4
1386. Sawadogo PM, Sia D, Onadja Y, Beogo I, Sangli G, Sawadogo N, Gnambani A, Bassinga G, Robins S, Tchouaket Nguemeleu E. Barriers and facilitators of access to sexual and reproductive health services among migrant, internally displaced, asylum seeking and refugee women: A scoping review. PLoS One. 2023 Sep 14;18(9):e0291486. eCollection 2023. https://doi.org/10.1371/journal.pone.0291486
1387. Pokharel B, Yelland J, Hooker L, Taft A. A Systematic Review of Culturally Competent Family Violence Responses to Women in Primary Care. Trauma Violence Abuse. 2023 Apr;24(2):928-945. Epub 2021 Oct 10. https://doi.org/10.1177/15248380211046968
1388. Ramos AA. Considerations in designing trauma-focused interventions for displaced Afghan women. Front Glob Womens Health. 2023 Feb 23;3:893957. eCollection 2022. https://doi.org/10.3389/fgwh.2022.893957
1389. Varshney K, Chu MG, Shet P, Hopkins J, Braga F, Ghosh P. Health and social consequences for survivors of genocidal rape: A systematic scoping review. J Trauma Stress. 2023 Aug;36(4):691-699. Epub 2023 May 28. https://doi.org/10.1002/jts.22936

1390. Cadman V, Soltani H, Spencer R, Marvin-Dowle K, Harrop D. Cesarean birth rates among migrants in Europe: A systematic review. Birth. 2023 Dec;50(4):657-671. Epub 2023 Mar 20. https://doi.org/10.1111/birt.12718
1391. Terkawi AS, Bakri B, Bakour C. Pregnancy and delivery characteristics and outcomes in Northwestern Syria: A prospective cohort study. Int J Gynaecol Obstet. 2023 Oct;163(1):256-264. Epub 2023 May 1. https://doi.org/10.1002/ijgo.14826
1392. Mprah A, Haith-Cooper M, Duda-Mikulin E, Meddings F. A systematic review and narrative synthesis of fathers' (including migrant fathers') experiences of pregnancy and childbirth. BMC Pregnancy Childbirth. 2023 Apr 11;23(1):238. https://doi.org/10.1186/s12884-023-05568-8
1393. Alam Z, Cairns JM, Scott M, Dean JA, Janda M. Interventions to increase cervical screening uptake among immigrant women: A systematic review and meta-analysis. PLoS One. 2023 Jun 2;18(6):e0281976. eCollection 2023. https://doi.org/10.1371/journal.pone.0281976
1394. Erhardt-Ohren B, McCoy SI, Feehan DM, Haar RJ, Prata N. Maternal mortality estimation methodologies: a scoping review and evaluation of suitability for use in humanitarian settings. Confl Health. 2024 Dec 19;18(1):75. https://doi.org/10.1186/s13031-024-00636-y
1395. Heer K, Mahmoud L, Abdelmeguid H, Selvan K, Malvankar-Mehta MS. Prevalence, Risk Factors, and Interventions of Postpartum Depression in Refugees and Asylum-Seeking Women: A Systematic Review and Meta-Analysis. Gynecol Obstet Invest. 2024;89(1):11-21. Epub 2024 Jan 12. https://doi.org/10.1159/000535719
1396. Bukkfalvi-Cadotte A, Naha G, Khanom A, Brown A, Snooks H. Are rates of clinical interventions during pregnancy and childbirth different for refugees and asylum seekers in high-income countries? A scoping review. BMC Pregnancy Childbirth. 2024 Nov 12;24(1):742. https://doi.org/10.1186/s12884-024-06893-2
1397. Mathis CM, Steiner JJ, Kappas Mazzio A, Bagwell-Gray M, Wachter K, Johnson-Agbakwu C, Messing J, Nizigiyimana J. Sexual and Reproductive Healthcare Needs of Refugee Women Exposed to Gender-Based Violence: The Case for Trauma-Informed Care in Resettlement Contexts. Int J Environ Res Public Health. 2024 Aug 8;21(8):1046. https://doi.org/10.3390/ijerph21081046
1398. Ether ST, Afrin S, Habib NN, Akter F, Chowdhury AT, Sayeed A, Raza S, Ahmed A, Saif-Ur-Rahman KM. Managing pre and postpartum mental health issues of refugee women from fragile and conflict-affected countries: A systematic review. Public Health Pract (Oxf). 2024 Dec 24;9:100573. eCollection 2025 Jun. https://doi.org/10.1016/j.puhip.2024.100573
1399. Schlothauer D, Teschemacher L, Breckenkamp J, Borde T, David M, Abou-Dakn M, Henrich W, Seidel V. Perinatal outcome of immigrant women with and without refugee status compared to non-immigrant women: results of the pregnancy and obstetric care for refugees (PROREF) cross-sectional study. Arch Gynecol Obstet. 2024 Nov;310(5):2413-2424. Epub 2024 Aug 16. https://doi.org/10.1007/s00404-024-07639-3
1400. Yeshitila YG, Gold L, Abimanyi-Ochom J, Riggs E, Tolossa T, Le HND. Effectiveness and cost-effectiveness of models of maternity care for women from migrant and refugee backgrounds in high-income countries: A systematic review. Soc Sci Med. 2024 Oct;358:117250. Epub 2024 Aug 23. https://doi.org/10.1016/j.socscimed.2024.117250
1401. Mirzaei Damabi N, Begum M, Avery JC, Padhani ZA, Lassi ZS. Unveiling silenced narratives: a scoping review on sexual function challenges in migrant and refugee women. Sex Med Rev. 2024 Jun 26;12(3):288-298. https://doi.org/10.1093/sxmrev/qeae005

1402. Napier-Raman S, Hossain SZ, Mpofu E, Lee MJ, Liamputtong P, Dune T. Abortion Experiences and Perspectives Amongst Migrants and Refugees: A Systematic Review. Int J Environ Res Public Health. 2024 Mar 8;21(3):312. https://doi.org/10.3390/ijerph21030312
1403. Doğan M, Özdemir F, Dağlituncezdi Çam Ş. Effect of migration on fertility and family planning: The case of Kayseri in Türkiye. Medicine (Baltimore). 2024 Dec 13;103(50):e40716. https://doi.org/10.1097/MD.0000000000040716
1404. Niroomand S, Gholizadeh L, Baird K. Iranian Immigrant Women's Experiences of Intimate Partner Violence: A Literature Review. J Immigr Minor Health. 2024 Oct;26(5):905-924. Epub 2024 Jul 2. https://doi.org/10.1007/s10903-024-01610-9
1405. Kalra N, Habumugisha L, Shankar A. Impacts of an abbreviated personal agency training with refugee women and their male partners on economic empowerment, gender-based violence, and mental health: a randomized controlled trial in Rwanda. BMC Public Health. 2024 May 14;24(1):1306. https://doi.org/10.1186/s12889-024-18780-8
1406. Seidel V, Teschemacher L, Breckenkamp J, Henrich W, Borde T, David M, Abou-Dakn M. [Obstetric Care for Gestational Diabetes in Refugee and Immigrant Women in Comparison to Non-Immigrant Women in Berlin: an Analysis of Quantitative Data of the Pregnancy and Obstetric Care for Refugees (PROREF)-study]. Z Geburtshilfe Neonatol. 2024 Jun;228(3):260-269. Epub 2024 Feb 19. https://doi.org/10.1055/a-2238-3364
1407. Vizheh M, Zurynski Y, Braithwaite J, Rapport F. Determinants of women's agency in accessing and utilising reproductive healthcare services; a systematic review. Cult Health Sex. 2024 Feb;26(2):248-264. Epub 2023 Apr 20. https://doi.org/10.1080/13691058.2023.2200814
1408. İşgüder ÇK, Arslan O, Gunkaya OS, Kanat-Pektas M, Tuğ N. Adolescent pregnancies in Turkey: a single center experience. Ann Saudi Med. 2024 Jan-Feb;44(1):11-17. Epub 2024 Feb 1. https://doi.org/10.5144/0256-4947.2024.11
1409. Adekola PO, Adedini SA. Sexual Violence, Disclosure Pattern, and Abortion and Post-Abortion Care Services in Displaced People's Camps in Africa: A Scoping Review. Int J Environ Res Public Health. 2024 Jul 30;21(8):1001. https://doi.org/10.3390/ijerph21081001
1410. Ngwibete A, Ogunbode O, Oluwasola T, Omigbodun A. Provision of sexual and reproductive health services to internally displaced women and refugees in Africa: a systematic review. Malawi Med J. 2024 Oct 16;36(3):238-249. eCollection 2024 Oct. https://doi.org/10.4314/mmj.v36i3.11
1411. Darebo TD, Spigt M, Teklewold B, Badacho AS, Mayer N, Teklewold M. The sexual and reproductive healthcare challenges when dealing with female migrants and refugees in low and middle-income countries (a qualitative evidence synthesis). BMC Public Health. 2024 Feb 19;24(1):520. https://doi.org/10.1186/s12889-024-17916-0
1412. Meinhart M, Seff I, Falb K, Deitch J, Roth D, Poulton C, Stark L. Humanitarian-specific recommendations for gender-transformative parenting programming: lessons from the field to address gender-based violence. EClinicalMedicine. 2024 Nov 27;78:102954. eCollection 2024 Dec. https://doi.org/10.1016/j.eclinm.2024.102954
1413. Gambir K, Hutchison C, Alexander L, Alessi EJ. Addressing the needs of cisgender, heterosexual men and LGBTIQ+ survivors of sexual violence: a scoping review of service delivery and funding priorities among humanitarian organisations. Glob Public Health. 2024 Jan;19(1):2371389. Epub 2024 Jul 14. https://doi.org/10.1080/17441692.2024.2371389
1414. Shiman LJ, Pickering S, Romero D, Jones HE. Abortion care access and experience among U.S. immigrants: A systematic review. J Migr Health. 2024 Jul 16;10:100248. eCollection 2024. https://doi.org/10.1016/j.jmh.2024.100248

1415. Yirsaw AN, Nigusie A, Andualem F, Getachew E, Getachew D, Tareke AA, Mihret MS, Lakew G. Cervical cancer screening utilization and associated factors among women living with HIV in Ethiopia, 2024: systematic review and meta-analysis. BMC Womens Health. 2024 Sep 19;24(1):521. https://doi.org/10.1186/s12905-024-03362-y
1416. Yirsaw AN, Tefera M, Bogale EK, Anagaw TF, Tiruneh MG, Fenta ET, Endeshaw D, Adal O, Tareke AA, Jemberu L, Getachew E, Belayneh AG, Andarge GA, Seid K, Lakew G. Applying the Health Belief Model to cervical cancer screening uptake among women in Ethiopia: a systematic review and meta-analysis. BMC Cancer. 2024 Oct 19;24(1):1294. https://doi.org/10.1186/s12885-024-13055-2
1417. Babiszewska-Aksamit M, Bzikowska-Jura A, Kotlińska A, Aduła A, Chrobak A, Domosud J, Drążkowska I, Gaweł P, Jakimiuk A, Kołodziej J, Królak-Olejnik B, Lisak-Gurba K, Mozdyniewicz K, Mołas A, Piątkowska A, Sinkiewicz-Darol E, Wesołowska A. Good practice in lactation counseling for Ukrainian refugee mothers to ensure the health and mental benefits of breastfeeding - an observational study. Arch Womens Ment Health. 2025 Apr;28(2):257-269. Epub 2024 Dec 6. https://doi.org/10.1007/s00737-024-01538-x
1418. Wachter K, Baccam Z, Burgess T, Alemi Q. A Scoping Review of the Intimate Partner Violence Literature Among Afghans Across Contexts. Trauma Violence Abuse. 2025 Jan;26(1):3-19. Epub 2024 Sep 5. https://doi.org/10.1177/15248380241271419
1419. Pangas J, Ogunsiji O, Elmir R, Raman S, Liamputtong P, Burns E, Dahlen HG, Schmied V. Refugee women's experiences negotiating motherhood and maternity care in a new country: A meta-ethnographic review. Int J Nurs Stud. 2019 Feb;90:31-45. Epub 2018 Dec 4. https://doi.org/10.1016/j.ijnurstu.2018.10.005
1420. Abdi HI, Hoover E, Fagan SE, Adsul P. Cervical Cancer Screening Among Immigrant and Refugee Women: Scoping-Review and Directions for Future Research. J Immigr Minor Health. 2020 Dec;22(6):1304-1319. https://doi.org/10.1007/s10903-020-01014-5
1421. Haque S, Malebranche M. Impact of culture on refugee women's conceptualization and experience of postpartum depression in high-income countries of resettlement: A scoping review. PLoS One. 2020 Sep 1;15(9):e0238109. eCollection 2020. https://doi.org/10.1371/journal.pone.0238109
1422. Rogers HJ, Hogan L, Coates D, Homer CSE, Henry A. Responding to the health needs of women from migrant and refugee backgrounds-Models of maternity and postpartum care in high-income countries: A systematic scoping review. Health Soc Care Community. 2020 Sep;28(5):1343-1365. Epub 2020 Jan 30. https://doi.org/10.1111/hsc.12950
1423. Harakow HI, Hvidman L, Wejse C, Eiset AH. Pregnancy complications among refugee women: A systematic review. Acta Obstet Gynecol Scand. 2021 Apr;100(4):649-657. Epub 2021 Jan 23. https://doi.org/10.1111/aogs.14070
1424. Abi Zeid Daou KR. Refugee Mothers Mental Health and Social Support Needs: A Systematic Review of Interventions for Refugee Mothers. Eur J Psychol. 2022 Aug 31;18(3):337-349. eCollection 2022 Aug. https://doi.org/10.5964/ejop.4665
1425. Larrea-Schiavon S, Vázquez-Quesada LM, Bartlett LR, Lam-Cervantes N, Sripad P, Vieitez I, Coutiño-Escamilla L. Interventions to Improve the Reproductive Health of Undocumented Female Migrants and Refugees in Protracted Situations: A Systematic Review. Glob Health Sci Pract. 2022 Dec 21;10(6):1-15. Print 2022 Dec 21. https://doi.org/10.9745/GHSP-D-21-00418
1426. Njue C, Sharmin S, Dawson A. Models of Maternal Healthcare for African refugee women in High-Income Countries: A Systematic Review. Midwifery. 2022 Jan;104:103187. Epub 2021 Oct 28. https://doi.org/10.1016/j.midw.2021.103187

1427. Soeiro RE, de Siqueira Guida JP, da-Costa-Santos J, Costa ML. Sexual and reproductive health (SRH) needs for forcibly displaced adolescent girls and young women (10-24 years old) in humanitarian settings: a mixed-methods systematic review. Reprod Health. 2023 Nov 24;20(1):174. https://doi.org/10.1186/s12978-023-01715-8
1428. Yeo S, Park Y, McClelland DJ, Ehiri J, Ernst K, Magrath P, Alaofè H. A scoping review of maternal health among resettled refugee women in the United States. Front Public Health. 2023 May 9;11:1157098. eCollection 2023. https://doi.org/10.3389/fpubh.2023.1157098
1429. Mengesha Z, Hawkey AJ, Baroudi M, Ussher JM, Perz J. Men of refugee and migrant backgrounds in Australia: a scoping review of sexual and reproductive health research. Sex Health. 2023 Feb;20(1):20-34. https://doi.org/10.1071/SH22073
1430. Stirling-Cameron E, Almukhaini S, Dol J, DuPlessis BJ, Stone K, Aston M, Goldenberg SM. Access and use of sexual and reproductive health services among asylum-seeking and refugee women in high-income countries: A scoping review. PLoS One. 2024 Nov 7;19(11):e0312746. eCollection 2024. https://doi.org/10.1371/journal.pone.0312746
1431. Inthavong AB, Pourmarzi D. Characteristics of Sexual Health Programs for Migrants, Refugees, and Asylum Seekers: A Scoping Review. Int J Environ Res Public Health. 2024 Jul 23;21(8):961. https://doi.org/10.3390/ijerph21080961
1432. Collins CH, Zimmerman C, Howard LM. Refugee, asylum seeker, immigrant women and postnatal depression: rates and risk factors. Arch Womens Ment Health. 2011 Feb;14(1):3-11. Epub 2010 Dec 14. https://doi.org/10.1007/s00737-010-0198-7
1433. Heslehurst N, Brown H, Pemu A, Coleman H, Rankin J. Perinatal health outcomes and care among asylum seekers and refugees: a systematic review of systematic reviews. BMC Med. 2018 Jun 12;16(1):89. https://doi.org/10.1186/s12916-018-1064-0
1434. McKnight P, Goodwin L, Kenyon S. A systematic review of asylum-seeking women's views and experiences of UK maternity care. Midwifery. 2019 Oct;77:16-23. Epub 2019 Jun 15. https://doi.org/10.1016/j.midw.2019.06.007
1435. Sturrock S, Williams E, Greenough A. Antenatal and perinatal outcomes of refugees in high income countries. J Perinat Med. 2020 Sep 1;49(1):80-93. https://doi.org/10.1515/jpm-2020-0389
1436. Frank GD, Fox D, Njue C, Dawson A. The maternity experiences of women seeking asylum in high-income countries: a meta-ethnography. Women Birth. 2021 Nov;34(6):531-539. Epub 2021 Jan 5. https://doi.org/10.1016/j.wombi.2020.12.012
1437. Kasper A, Mohwinkel LM, Nowak AC, Kolip P. Maternal health care for refugee women - A qualitative review. Midwifery. 2022 Jan;104:103157. Epub 2021 Sep 30. https://doi.org/10.1016/j.midw.2021.103157
1438. Balaam MC, Kingdon C, Haith-Cooper M. A Systematic Review of Perinatal Social Support Interventions for Asylum-seeking and Refugee Women Residing in Europe. J Immigr Minor Health. 2022 Jun;24(3):741-758. Epub 2021 Jul 17. https://doi.org/10.1007/s10903-021-01242-3
1439. Davidson N, Hammarberg K, Romero L, Fisher J. Access to preventive sexual and reproductive health care for women from refugee-like backgrounds: a systematic review. BMC Public Health. 2022 Feb 27;22(1):403. https://doi.org/10.1186/s12889-022-12576-4
1440. Chalmiers MA, Karaki F, Muriki M, Mody SK, Chen A, Thiel de Bocanegra H. Refugee women's experiences with contraceptive care after resettlement in high-income countries: A critical interpretive synthesis. Contraception. 2022 Apr;108:7-18. Epub 2021 Dec 29. https://doi.org/10.1016/j.contraception.2021.11.004
1441. Jahangirifar M, Fooladi E, Davis SR, Islam RM. Menopause symptoms, sexual dysfunctions and pelvic floor disorders in refugee and asylum seeker women: a scoping

review. Climacteric. 2023 Aug;26(4):373-380. Epub 2023 Feb 13. https://doi.org/10.1080/13697137.2023.2173572
1442. Donnelly A, Haintz GL, McKenzie H, Graham M. Influences on reproductive decision-making among forcibly displaced women resettling in high-income countries: a scoping review and thematic analysis. Int J Equity Health. 2023 Sep 5;22(1):179. https://doi.org/10.1186/s12939-023-01993-5
1443. Ramadan M, Rukh-E-Qamar H, Yang S, Vang ZM. Fifty years of evidence on perinatal experience among refugee and asylum-seeking women in Organization for Economic Co-operation and Development (OECD) countries: A scoping review. PLoS One. 2023 Oct 26;18(10):e0287617. eCollection 2023. https://doi.org/10.1371/journal.pone.0287617
1444. Saunders SL, Sutcliffe KL, McOrist NS, Levett KM. The associations between women who are immigrants, refugees, or asylum seekers, access to universal healthcare, and the timely uptake of antenatal care: A systematic review. Aust N Z J Obstet Gynaecol. 2023 Apr;63(2):134-145. Epub 2022 Dec 8. https://doi.org/10.1111/ajo.13632
1445. Logie CH, MacKenzie F, Malama K, Lorimer N, Lad A, Zhao M, Narasimhan M, Fahme S, Turan B, Kagunda J, Konda K, Hasham A, Perez-Brumer A. Sexual and reproductive health among forcibly displaced persons in urban environments in low and middle-income countries: scoping review findings. Reprod Health. 2024 Apr 12;21(1):51. https://doi.org/10.1186/s12978-024-01780-7
1446. Firth A, Haith-Cooper M, Dickerson J, Hart A. Perinatal depression: Factors affecting help-seeking behaviours in asylum seeking and refugee women. A systematic review. J Migr Health. 2022 Sep 2;6:100128. eCollection 2022. https://doi.org/10.1016/j.jmh.2022.100128
1447. Rezzoug D, Baubet T, Broder G, Taïeb O, Moro MR. Addressing the mother-infant relationship in displaced communities. Child Adolesc Psychiatr Clin N Am. 2008 Jul;17(3):551-68, viii. https://doi.org/10.1016/j.chc.2008.02.008
1448. Abuhaloob L, MacGillivray S, Mossey P, Freeman R. Maternal and child oral health interventions in Middle East and North Africa regions: a rapid review. Int Dent J. 2019 Dec;69(6):409-418. Epub 2019 Jul 23. https://doi.org/10.1111/idj.12506
1449. Ponguta LA, Issa G, Aoudeh L, Maalouf C, Hein SD, Zonderman AL, Katsovich L, Khoshnood K, Bick J, Awar A, Nourallah S, Householder S, Moore CC, Salah R, Britto PR, Leckman JF. Effects of the Mother-Child Education Program on Parenting Stress and Disciplinary Practices Among Refugee and Other Marginalized Communities in Lebanon: A Pilot Randomized Controlled Trial. J Am Acad Child Adolesc Psychiatry. 2020 Jun;59(6):727-738. Epub 2020 Jan 31. https://doi.org/10.1016/j.jaac.2019.12.010
1450. Kwankye SO, Richter S, Okeke-Ihejirika P, Gomma H, Obegu P, Salami B. A review of the literature on sexual and reproductive health of African migrant and refugee children. Reprod Health. 2021 Apr 17;18(1):81. https://doi.org/10.1186/s12978-021-01138-3
1451. Bakesiima R, Beyeza-Kashesya J, Tumwine JK, Chalo RN, Gemzell-Danielsson K, Cleeve A, Larsson EC. Effect of peer counselling on acceptance of modern contraceptives among female refugee adolescents in northern Uganda: A randomised controlled trial. PLoS One. 2021 Sep 2;16(9):e0256479. eCollection 2021. https://doi.org/10.1371/journal.pone.0256479
1452. Aibangbee M, Micheal S, Mapedzahama V, Liamputtong P, Pithavadian R, Hossain Z, Mpofu E, Dune T. Migrant and Refugee Youth's Sexual and Reproductive Health and Rights: A Scoping Review to Inform Policies and Programs. Int J Public Health. 2023 Jun 5;68:1605801. eCollection 2023. https://doi.org/10.3389/ijph.2023.1605801

1453. Napier-Raman S, Hossain SZ, Lee MJ, Mpofu E, Liamputtong P, Dune T. Migrant and refugee youth perspectives on sexual and reproductive health and rights in Australia: a systematic review. Sex Health. 2023 Feb;20(1):35-48. https://doi.org/10.1071/SH22081
1454. Al Karmi J, Alshrouf MA, Haddad TA, Alhanbali AE, Raiq NA, Ghanem H, Ibrahim OB, Khamis T, Muhaidat N. Urinary and reproductive tract infection symptoms and menstrual hygiene practices in refugee camps in Jordan: A cross-sectional study. Womens Health (Lond). 2024 Jan-Dec;20:17455057241240920. https://doi.org/10.1177/17455057241240920
1455. Tol WA, Greene MC, Likindikoki S, Misinzo L, Ventevogel P, Bonz AG, Bass JK, Mbwambo JKK. An integrated intervention to reduce intimate partner violence and psychological distress with refugees in low-resource settings: study protocol for the Nguvu cluster randomized trial. BMC Psychiatry. 2017 May 18;17(1):186. https://doi.org/10.1186/s12888-017-1338-7
1456. Anderson FM, Hatch SL, Comacchio C, Howard LM. Prevalence and risk of mental disorders in the perinatal period among migrant women: a systematic review and meta-analysis. Arch Womens Ment Health. 2017 Jun;20(3):449-462. Epub 2017 Apr 8. https://doi.org/10.1007/s00737-017-0723-z
1457. Shovers SM, Bachman SS, Popek L, Turchi RM. Maternal postpartum depression: risk factors, impacts, and interventions for the NICU and beyond. Curr Opin Pediatr. 2021 Jun 1;33(3):331-341. https://doi.org/10.1097/MOP.0000000000001011
1458. Markey K, Moloney M, O'Donnell CA, Noonan M, O'Donnell C, Tuohy T, MacFarlane A, Huschke S, Mohamed AH, Doody O. Enablers of and Barriers to Perinatal Mental Healthcare Access and Healthcare Provision for Refugee and Asylum-Seeking Women in the WHO European Region: A Scoping Review. Healthcare (Basel). 2024 Sep 1;12(17):1742. https://doi.org/10.3390/healthcare12171742
1459. Winter AK, Due C, Ziersch A. Wellbeing Outcomes and Risk and Protective Factors for Parents with Migrant and Refugee Backgrounds from the Middle East in the First 1000 Days: A Systematic Review. J Immigr Minor Health. 2024 Apr;26(2):395-408. Epub 2023 Jul 6. https://doi.org/10.1007/s10903-023-01510-4
1460. Ponguta LA, Issa G, Aoudeh L, Maalouf C, Nourallah S, Khoshnood K, Zonderman AL, Katsovich L, Moore C, Salah R, Al-Soleiti M, Britto PR, Leckman JF. Implementation Evaluation of the Mother-Child Education Program Among Refugee and Other Vulnerable Communities in Lebanon. New Dir Child Adolesc Dev. 2019 Sep;2019(167):91-116. Epub 2019 Sep 11. https://doi.org/10.1002/cad.20314
1461. Erenoğlu R, Yaman Sözbir Ş. The Effect of Health Education Given to Syrian Refugee Women in Their Own Language on Awareness of Breast and Cervical Cancer, in Turkey: a Randomized Controlled Trial. J Cancer Educ. 2020 Apr;35(2):241-247. https://doi.org/10.1007/s13187-019-01604-4
1462. Kingsford Smith D, Szuster F. Aspects of tooth decay in recently arrived refugees. Aust N Z J Public Health. 2000 Dec;24(6):623-6. https://doi.org/10.1111/j.1467-842x.2000.tb00529.x
1463. Tomashek KM, Woodruff BA, Gotway CA, Bloland P, Mbaruku G. Randomized intervention study comparing several regimens for the treatment of moderate anemia among refugee children in Kigoma Region, Tanzania. Am J Trop Med Hyg. 2001 Mar-Apr;64(3-4):164-71. https://doi.org/10.4269/ajtmh.2001.64.164
1464. Hollifield M, Warner TD, Lian N, Krakow B, Jenkins JH, Kesler J, Stevenson J, Westermeyer J. Measuring trauma and health status in refugees: a critical review. JAMA. 2002 Aug 7;288(5):611-21. https://doi.org/10.1001/jama.288.5.611

1465. Weise Prinzo Z, de Benoist B. Meeting the challenges of micronutrient deficiencies in emergency-affected populations. Proc Nutr Soc. 2002 May;61(2):251-7. https://doi.org/10.1079/PNS2002151
1466. Robertson E, Iglesias E, Johansson SE, Sundquist J. Migration status and limiting long-standing illness: a longitudinal study of women of childbearing age in Sweden. Eur J Public Health. 2003 Jun;13(2):99-104. https://doi.org/10.1093/eurpub/13.2.99
1467. Nasir K, Hyder AA, Shahbaz CM. Injuries among Afghan refugees: review of evidence. Prehosp Disaster Med. 2004 Apr-Jun;19(2):169-73. https://doi.org/10.1017/s1049023x00001692
1468. Fernández E, Riestra S, Rodrigo L, Blanco C, López-Vázquez A, Fuentes D, Moreno M, López-Larrea C. Comparison of six human anti-transglutaminase ELISA-tests in the diagnosis of celiac disease in the Saharawi population. World J Gastroenterol. 2005 Jun 28;11(24):3762-6. https://doi.org/10.3748/wjg.v11.i24.3762
1469. Benson J, Skull S. Hiding from the sun - vitamin D deficiency in refugees. Aust Fam Physician. 2007 May;36(5):355-7.
1470. Kinzie JD, Riley C, McFarland B, Hayes M, Boehnlein J, Leung P, Adams G. High prevalence rates of diabetes and hypertension among refugee psychiatric patients. J Nerv Ment Dis. 2008 Feb;196(2):108-12. https://doi.org/10.1097/NMD.0b013e318162aa51
1471. Weaver H, Roberts B. Drinking and displacement: a systematic review of the influence of forced displacement on harmful alcohol use. Subst Use Misuse. 2010 Nov;45(13):2340-55. Epub 2010 May 14. https://doi.org/10.3109/10826081003793920
1472. Fox SH, Willis MS. Dental restorations for dinka and nuer refugees: a confluence of culture and healing. Transcult Psychiatry. 2010 Jul;47(3):452-72. https://doi.org/10.1177/1363461510374559
1473. MacDuff S, Grodin MA, Gardiner P. The use of complementary and alternative medicine among refugees: a systematic review. J Immigr Minor Health. 2011 Jun;13(3):585-99. https://doi.org/10.1007/s10903-010-9318-8
1474. Hollander AC, Bruce D, Ekberg J, Burström B, Borrell C, Ekblad S. Longitudinal study of mortality among refugees in Sweden. Int J Epidemiol. 2012 Aug;41(4):1153-61. Epub 2012 May 2. https://doi.org/10.1093/ije/dys072
1475. Renzaho AM, Polonsky MJ. Examining demographic and socio-economic correlates of accurate knowledge about blood donation among African migrants in Australia. Transfus Med. 2012 Oct;22(5):321-31. Epub 2012 Jul 25. https://doi.org/10.1111/j.1365-3148.2012.01175.x
1476. Crosby SS. Primary care management of non-English-speaking refugees who have experienced trauma: a clinical review. JAMA. 2013 Aug 7;310(5):519-28. https://doi.org/10.1001/jama.2013.8788
1477. Tappuni AR, Tbakhi A, Sharquie KE, Hayani RK, Al-Kaisi A, Lafi A, Al-Araji A. A comparative study of the genetics of Behcet's disease in Iraq: international collaboration to transfer clinical and laboratory skills to Baghdad medical school and hospitals. Med Confl Surviv. 2013 Jan-Mar;29(1):57-68. https://doi.org/10.1080/13623699.2013.765201
1478. van Wyk S, Schweitzer RD. A systematic review of naturalistic interventions in refugee populations. J Immigr Minor Health. 2014 Oct;16(5):968-77. https://doi.org/10.1007/s10903-013-9835-3
1479. Amara AH, Aljunid SM. Noncommunicable diseases among urban refugees and asylum-seekers in developing countries: a neglected health care need. Global Health. 2014 Apr 3;10:24. https://doi.org/10.1186/1744-8603-10-24

1480. Cheng HM, Kumarasinghe SP. Dermatological problems of asylum seekers arriving on boats: a case report from Australia and a brief review. Australas J Dermatol. 2014 Nov;55(4):270-4. Epub 2014 Sep 1. https://doi.org/10.1111/ajd.12183
1481. Spiegel P, Khalifa A, Mateen FJ. Cancer in refugees in Jordan and Syria between 2009 and 2012: challenges and the way forward in humanitarian emergencies. Lancet Oncol. 2014 Jun;15(7):e290-7. https://doi.org/10.1016/S1470-2045(14)70067-1
1482. Terasaki G, Ahrenholz NC, Haider MZ. Care of Adult Refugees with Chronic Conditions. Med Clin North Am. 2015 Sep;99(5):1039-58. Epub 2015 Jul 7. https://doi.org/10.1016/j.mcna.2015.05.006
1483. Wagner J, Berthold SM, Buckley T, Kong S, Kuoch T, Scully M. Diabetes among refugee populations: what newly arriving refugees can learn from resettled Cambodians. Curr Diab Rep. 2015 Aug;15(8):56. https://doi.org/10.1007/s11892-015-0618-1
1484. Bo A, Zinckernagel L, Krasnik A, Petersen JH, Norredam M. Coronary heart disease incidence among non-Western immigrants compared to Danish-born people: effect of country of birth, migrant status, and income. Eur J Prev Cardiol. 2015 Oct;22(10):1281-9. Epub 2014 Sep 26. https://doi.org/10.1177/2047487314551538
1485. Shahin Y, Kapur A, Seita A. Diabetes care in refugee camps: the experience of UNRWA. Diabetes Res Clin Pract. 2015 Apr;108(1):1-6. Epub 2015 Jan 31. https://doi.org/10.1016/j.diabres.2015.01.035
1486. Otoukesh S, Mojtahedzadeh M, Figlin RA, Rosenfelt FP, Behazin A, Sherzai D, Cooper CJ, Nahleh ZA. Literature Review and Profile of Cancer Diseases Among Afghan Refugees in Iran: Referrals in Six Years of Displacement. Med Sci Monit. 2015 Nov 23;21:3622-8. https://doi.org/10.12659/msm.895173
1487. Lo J, Patel P, Roberts B. A systematic review on tobacco use among civilian populations affected by armed conflict. Tob Control. 2016 Mar;25(2):129-40. Epub 2015 Mar 13. https://doi.org/10.1136/tobaccocontrol-2014-052054
1488. Wang Y, Min J, Harris K, Khuri J, Anderson LM. A Systematic Examination of Food Intake and Adaptation to the Food Environment by Refugees Settled in the United States. Adv Nutr. 2016 Nov 15;7(6):1066-1079. Print 2016 Nov. https://doi.org/10.3945/an.115.011452
1489. Keboa MT, Hiles N, Macdonald ME. The oral health of refugees and asylum seekers: a scoping review. Global Health. 2016 Oct 7;12(1):59. https://doi.org/10.1186/s12992-016-0200-x
1490. Isreb MA, Rifai AO, Murad LB, Al-Makki A, Al-Saghir F, Sekkarie MA. Care and outcomes of end-stage kidney disease patients in times of armed conflict: recommendations for action. Clin Nephrol. 2016 May;85(5):281-8. https://doi.org/10.5414/CN108795
1491. Kim YJ, Lee YH, Lee YJ, Kim KJ, An JH, Kim NH, Kim HY, Choi DS, Kim SG. Prevalence of metabolic syndrome and its related factors among North Korean refugees in South Korea: a cross-sectional study. BMJ Open. 2016 Jun 1;6(6):e010849. https://doi.org/10.1136/bmjopen-2015-010849
1492. Doocy S, Lyles E, Akhu-Zaheya L, Oweis A, Al Ward N, Burton A. Health Service Utilization among Syrian Refugees with Chronic Health Conditions in Jordan. PLoS One. 2016 Apr 13;11(4):e0150088. eCollection 2016. https://doi.org/10.1371/journal.pone.0150088
1493. Stewart BT, Lafta R, Esa Al Shatari SA, Cherewick M, Flaxman A, Hagopian A, Burnham G, Kushner AL. Fall injuries in Baghdad from 2003 to 2014: Results of a randomised household cluster survey. Injury. 2016 Jan;47(1):244-9. Epub 2015 Nov 14. https://doi.org/10.1016/j.injury.2015.11.006

1494. Stewart BT, Lafta R, Cherewick M, Esa Al Shatari SA, Flaxman AD, Hagopian A, Galway LP, Takaro TK, Burnham G, Kushner AL, Mock C. Road traffic injuries in Baghdad from 2003 to 2014: results of a randomised household cluster survey. Inj Prev. 2016 Oct;22(5):321-7. Epub 2016 Feb 5. https://doi.org/10.1136/injuryprev-2015-041707
1495. Alduraidi H, Waters CM. Health-related quality of life of Palestinian refugees inside and outside camps in Jordan. Nurs Outlook. 2017 Jul-Aug;65(4):436-443. Epub 2017 May 26. https://doi.org/10.1016/j.outlook.2017.05.007
1496. Yankam Lemdjo FM. Unveiling the Menace of Hidden Hunger in Refugee Camps: Nutritional Status among Refugees, States Responsibility, and Key African Strategies on Nutrition. World Rev Nutr Diet. 2017;118:152-160. Epub 2018 Apr 13. https://doi.org/10.1159/000484642
1497. Williams ACC, Amris K. Treatment of persistent pain from torture: review and commentary. Med Confl Surviv. 2017 Mar;33(1):60-81. Epub 2016 Oct 13. https://doi.org/10.1080/13623699.2016.1242050
1498. Sekkarie MA, Abdel-Rahman EM. Cultural Challenges in the Care of Refugees with End-Stage Renal Disease: What Western Nephrologists Should Know. Nephron. 2017;137(2):85-90. Epub 2017 Jun 8. https://doi.org/10.1159/000477362
1499. Pavli A, Maltezou H. Health problems of newly arrived migrants and refugees in Europe. J Travel Med. 2017 Jul 1;24(4). https://doi.org/10.1093/jtm/tax016
1500. Holguin F, Moughrabieh MA, Ojeda V, Patel SR, Peyrani P, Pinedo M, Celedón JC, Douglas IS, Upson DJ, Roman J. Respiratory Health in Migrant Populations: A Crisis Overlooked. Ann Am Thorac Soc. 2017 Feb;14(2):153-159. https://doi.org/10.1513/AnnalsATS.201608-592PS
1501. Melo M, Ata-Ali J. Accuracy of the estimation of dental age in comparison with chronological age in a Spanish sample of 2641 living subjects using the Demirjian and Nolla methods. Forensic Sci Int. 2017 Jan;270:276.e1-276.e7. Epub 2016 Oct 12. https://doi.org/10.1016/j.forsciint.2016.10.001
1502. Odolini S, Gobbi F, Zammarchi L, Migliore S, Mencarini P, Vecchia M, di Lauria N, Schivazappa S, Sabatini T, Chianura L, Vanino E, Piacentini D, Zanotti P, Bussi A, Bartoloni A, Bisoffi Z, Castelli F. Febrile rhabdomyolysis of unknown origin in refugees coming from West Africa through the Mediterranean. Int J Infect Dis. 2017 Sep;62:77-80. Epub 2017 Jul 26. https://doi.org/10.1016/j.ijid.2017.07.018
1503. Rosenthal T. Immigration and Acculturation: Impact on Health and Well-Being of Immigrants. Curr Hypertens Rep. 2018 Jul 3;20(8):70. https://doi.org/10.1007/s11906-018-0872-0
1504. Leone A, Bertoli S, Di Lello S, Bassoli A, Ravasenghi S, Borgonovo G, Forlani F, Battezzati A. Effect of Moringa oleifera Leaf Powder on Postprandial Blood Glucose Response: In Vivo Study on Saharawi People Living in Refugee Camps. Nutrients. 2018 Oct 12;10(10):1494. https://doi.org/10.3390/nu10101494
1505. Van Biesen W, Vanholder R, Ernandez T, Drewniak D, Luyckx V. Caring for Migrants and Refugees With End-Stage Kidney Disease in Europe. Am J Kidney Dis. 2018 May;71(5):701-709. Epub 2017 Dec 21. https://doi.org/10.1053/j.ajkd.2017.10.015
1506. Banerjee AT, Shah BR. Differences in prevalence of diabetes among immigrants to Canada from South Asian countries. Diabet Med. 2018 Jul;35(7):937-943. Epub 2018 May 3. https://doi.org/10.1111/dme.13647
1507. Kim YJ, Kim SG, Lee YH. Prevalence of General and Central Obesity and Associated Factors among North Korean Refugees in South Korea by Duration after Defection from

North Korea: A Cross-Sectional Study. Int J Environ Res Public Health. 2018 Apr 20;15(4):811. https://doi.org/10.3390/ijerph15040811
1508. Saleh S, El Harakeh A, Baroud M, Zeineddine N, Farah A, Sibai AM. Costs associated with management of non-communicable diseases in the Arab Region: a scoping review. J Glob Health. 2018 Dec;8(2):020410. https://doi.org/10.7189/jogh.08.020410
1509. Moreau AM, Hennous F, Dabbagh B, Ferraz Dos Santos B. Oral Health Status of Refugee Children in Montreal. J Immigr Minor Health. 2019 Aug;21(4):693-698. https://doi.org/10.1007/s10903-018-0835-1
1510. Jurković M, Tomašković I, Tomašković M, Smital Zore B, Pavić I, Roić AC. Refugee Status as a Possible Risk Factor for Childhood Enuresis. Int J Environ Res Public Health. 2019 Apr 11;16(7):1293. https://doi.org/10.3390/ijerph16071293
1511. Akik C, Ghattas H, Mesmar S, Rabkin M, El-Sadr WM, Fouad FM. Host country responses to non-communicable diseases amongst Syrian refugees: a review. Confl Health. 2019 Mar 22;13:8. eCollection 2019. https://doi.org/10.1186/s13031-019-0192-2
1512. Clarysse K, Grosber M, Ring J, Gutermuth J, Kivlahan C. Skin lesions, differential diagnosis and practical approach to potential survivors of torture. J Eur Acad Dermatol Venereol. 2019 Jul;33(7):1232-1240. Epub 2019 Apr 15. https://doi.org/10.1111/jdv.15439
1513. Richter K, Baumgärtner L, Niklewski G, Peter L, Köck M, Kellner S, Hillemacher T, Büttner-Teleaga A. Sleep disorders in migrants and refugees: a systematic review with implications for personalized medical approach. EPMA J. 2020 May 13;11(2):251-260. eCollection 2020 Jun. https://doi.org/10.1007/s13167-020-00205-2
1514. Alsubhi M, Goldthorpe J, Epton T, Khanom S, Peters S. What factors are associated with obesity-related health behaviours among child refugees following resettlement in developed countries? A systematic review and synthesis of qualitative and quantitative evidence. Obes Rev. 2020 Nov;21(11):e13058. Epub 2020 Jun 30. https://doi.org/10.1111/obr.13058
1515. Paisi M, Baines R, Burns L, Plessas A, Radford P, Shawe J, Witton R. Barriers and facilitators to dental care access among asylum seekers and refugees in highly developed countries: a systematic review. BMC Oral Health. 2020 Nov 25;20(1):337. https://doi.org/10.1186/s12903-020-01321-1
1516. Karadağ ŞG, Sönmez HE, Demir F, Çakan M, Öztürk K, Tanatar A, Çakmak F, Sözeri B, Aktay Ayaz N. Rheumatic diseases in Syrian refugee children: a retrospective multicentric study in Turkey. Rheumatol Int. 2020 Apr;40(4):583-589. Epub 2020 Feb 22. https://doi.org/10.1007/s00296-020-04534-3
1517. Hamad L, Kreidieh K, Hamdan MB, Nakouzi G, Yazbek S. Mapping the Diverse Genetic Disorders and Rare Diseases Among the Syrian Population: Implications on Refugee Health and Health Services in Host Countries. J Immigr Minor Health. 2020 Dec;22(6):1347-1367. https://doi.org/10.1007/s10903-020-00987-7
1518. Joarder T, Sutradhar I, Hasan MI, Bulbul MMI. A Record Review on the Health Status of Rohingya Refugees in Bangladesh. Cureus. 2020 Aug 15;12(8):e9753. https://doi.org/10.7759/cureus.9753
1519. McNatt ZZ. Addressing noncommunicable diseases among urban refugees in the Middle East and North Africa - a scoping review. Confl Health. 2020 Feb 18;14:9. eCollection 2020. https://doi.org/10.1186/s13031-020-0255-4
1520. Denli Yalvac ES. Cardiovascular diseases and their risk factors among Syrian refugees in Turkey. Rev Epidemiol Sante Publique. 2020 Apr;68(2):137-144. Epub 2020 Mar 2. https://doi.org/10.1016/j.respe.2019.11.020

1521. Kim KJ, An JH, Kim KJ, Yu JH, Kim NH, Yoo HJ, Kim HY, Seo JA, Kim NH, Choi KM, Baik SH, Kim SG. Prevalence of osteoporosis among North Korean women refugees living in South Korea: a comparative cross-sectional study. BMJ Open. 2020 Jun 11;10(6):e036230. https://doi.org/10.1136/bmjopen-2019-036230
1522. DiVito B, Talavlikar R, Seifu S. Common Hematologic, Nutritional, Asthma/Allergic Conditions and Lead Screening/Management. Prim Care. 2021 Mar;48(1):67-81. Epub 2020 Nov 27. https://doi.org/10.1016/j.pop.2020.10.002
1523. Kumar GS, Beeler JA, Seagle EE, Jentes ES. Long-Term Physical Health Outcomes of Resettled Refugee Populations in the United States: A Scoping Review. J Immigr Minor Health. 2021 Aug;23(4):813-823. Epub 2021 Jan 30. https://doi.org/10.1007/s10903-021-01146-2
1524. Alrashdi M, Hameed A, Cervantes Mendez MJ, Farokhi M. Education intervention with respect to the oral health knowledge, attitude, and behaviors of refugee families: A randomized clinical trial of effectiveness. J Public Health Dent. 2021 Jun;81(2):90-99. Epub 2020 Oct 20. https://doi.org/10.1111/jphd.12415
1525. Shahin W, Stupans I, Kennedy G. Health beliefs and chronic illnesses of refugees: a systematic review. Ethn Health. 2021 Jul;26(5):756-768. Epub 2018 Dec 11. https://doi.org/10.1080/13557858.2018.1557118
1526. Santana J, Lemma A, O'Connor MH, Kelli H. Understanding risk factors and preventive measures of cardiovascular disease in refugee communities. Heart. 2021 Sep;107(18):1518-1519. Epub 2021 Jul 21. https://doi.org/10.1136/heartjnl-2021-319435
1527. Zibara V, Costanian C, Al Haddad N, Kilani H, Tohme F, Aoun Bahous S. Epidemiology and management of hypertension among refugees in the Middle East: a review of the literature. J Hum Hypertens. 2021 Aug;35(8):657-666. Epub 2021 Mar 2. https://doi.org/10.1038/s41371-021-00508-5
1528. Tan ST, Low PTA, Howard N, Yi H. Social capital in the prevention and management of non-communicable diseases among migrants and refugees: a systematic review and meta-ethnography. BMJ Glob Health. 2021 Dec;6(12):e006828. https://doi.org/10.1136/bmjgh-2021-006828
1529. Payton C, Kimball S, Ahrenholz NC, Wieland ML. Preventive Care and Management of Chronic Diseases in Immigrant Adults. Prim Care. 2021 Mar;48(1):83-97. Epub 2020 Dec 7. https://doi.org/10.1016/j.pop.2020.09.006
1530. Waheed A, McCloskey A, Kennedy F, Seraj SM, Khan J, Nama N, Johnson O, Lo P, Magee H, Akbar W, Ullah A, Cason FD. Colorectal Cancer Screening Challenges in the Recent Afghan Refugee Population: A Comprehensive Review Article. Cureus. 2022 Feb 20;14(2):e22400. eCollection 2022 Feb. https://doi.org/10.7759/cureus.22400
1531. Gingell T, Murray K, Correa-Velez I, Gallegos D. Determinants of food security among people from refugee backgrounds resettled in high-income countries: A systematic review and thematic synthesis. PLoS One. 2022 Jun 2;17(6):e0268830. eCollection 2022. https://doi.org/10.1371/journal.pone.0268830
1532. Vickery KD, Novotny PJ, Ford BR, Lantz K, Kavistan S, Singh D, Hernandez V, Sia IG, Wieland ML. Experiences of Hispanic Safety Net Clinic Patients With Diabetes During the COVID-19 Pandemic. Sci Diabetes Self Manag Care. 2022 Apr;48(2):87-97. Epub 2022 Feb 4. https://doi.org/10.1177/26350106221076037
1533. Alalawneh M, Berardi A, Nuaimi N, Basheti IA. Improving Syrian refugees' knowledge of medications and adherence following a randomized control trial assessing the effect of a medication management review service. PLoS One. 2022 Oct 14;17(10):e0276304. eCollection 2022. https://doi.org/10.1371/journal.pone.0276304

1534. Ngaruiya C, Bernstein R, Leff R, Wallace L, Agrawal P, Selvam A, Hersey D, Hayward A. Systematic review on chronic non-communicable disease in disaster settings. BMC Public Health. 2022 Jun 21;22(1):1234. https://doi.org/10.1186/s12889-022-13399-z
1535. Hamrah MS, Bartlett L, Jang S, Roccati E, Vickers JC. Modifiable Risk Factors for Dementia Among Migrants, Refugees and Asylum Seekers in Australia: A Systematic Review. J Immigr Minor Health. 2023 Jun;25(3):692-711. Epub 2023 Jan 18. https://doi.org/10.1007/s10903-022-01445-2
1536. Almoussa M, Mattei J. Cardiovascular health and risk factors in African refugees and immigrants in the United States: a narrative review. Ethn Health. 2023 Apr;28(3):399-412. Epub 2022 Mar 21. https://doi.org/10.1080/13557858.2022.2052712
1537. Racine L, D'Souza MS, Tinampay C. Effectiveness of breast cancer screening interventions in improving screening rates and preventive activities in Muslim refugee and immigrant women: A systematic review and meta-analysis. J Nurs Scholarsh. 2023 Jan;55(1):329-344. Epub 2022 Oct 5. https://doi.org/10.1111/jnu.12818
1538. Hokland P, Daar S, Khair W, Sheth S, Taher AT, Torti L, Hantaweepant C, Rund D. Thalassaemia-A global view. Br J Haematol. 2023 Apr;201(2):199-214. Epub 2023 Feb 17. https://doi.org/10.1111/bjh.18671
1539. Banihashem Rad SA, Esteves Oliveira M, Maklennan A, Castiglia P, Campus G. Higher prevalence of dental caries and periodontal problems among refugees: A scoping review. J Glob Health. 2023 Sep 15;13:04111. https://doi.org/10.7189/jogh.13.04111
1540. Shortland T, McGranahan M, Stewart D, Oyebode O, Shantikumar S, Proto W, Malik B, Yau R, Cobbin M, Sabouni A, Rudge G, Kidy F. A systematic review of the burden of, access to services for and perceptions of patients with overweight and obesity, in humanitarian crisis settings. PLoS One. 2023 Apr 24;18(4):e0282823. eCollection 2023. https://doi.org/10.1371/journal.pone.0282823
1541. Baskaran A, Marogi E, Bitar R, Attarian H, Saadi A. Improving Sleep Health Among Refugees: A Systematic Review. Neurol Clin Pract. 2023 Apr;13(2):e200139. Epub 2023 Mar 14. https://doi.org/10.1212/CPJ.0000000000200139
1542. de-Graft Aikins A, Sanuade O, Baatiema L, Adjaye-Gbewonyo K, Addo J, Agyemang C. How chronic conditions are understood, experienced and managed within African communities in Europe, North America and Australia: A synthesis of qualitative studies. PLoS One. 2023 Feb 15;18(2):e0277325. eCollection 2023. https://doi.org/10.1371/journal.pone.0277325
1543. Saadi A, Williams J, Parvez A, Alegría M, Vranceanu AM. Head Trauma in Refugees and Asylum Seekers: A Systematic Review. Neurology. 2023 May 23;100(21):e2155-e2169. Epub 2023 Apr 5. https://doi.org/10.1212/WNL.0000000000207261
1544. Bousquet J, Samolinski B, Kaidashev I, Maurer M, Roche N, Sousa-Pinto B, Kurchenko A, Stepanenko R, Tsaryk V, Klimek L, Ventura MT, Bedbrook A, Czarlewski W, Lysanets Y, Kupczyk M, Skolimowski Ł, Kulus M, Del Giacco S, Ollert M, Garcia-Aymerich J, Robalo Cordeiro C, Yorgancioglu A, Schlapbach C, Amaral R, Bonaglia C, Bossé I, Buquicchio R, Christou D, Fedoruk G, Fontanesi P, Gemicioglu B, Giuliano AFM, Lepore P, Nakonechna A, Neisinger S, Pereira AM, Ramanauskaite A, Raciborski F, Sitkauskiene B, Sokhatska O, Stepanenko V, Stevanovic K, Syzon O, Kvedariene V, de Vries G, van Eerd M, Valiulis A, Fonseca JA, Anto JM, Haahtela T, Schünemann H, Zuberbier T. UCRAID (Ukrainian Citizen and refugee electronic support in Respiratory diseases, Allergy, Immunology and Dermatology) action plan. Allergy. 2023 Oct;78(10):2581-2595. Epub 2023 Aug 28. https://doi.org/10.1111/all.15855

1545. Jankovic-Rankovic J, Panter-Brick C. Physiological and genomic signatures of war and displacement: A comprehensive literature review and future directions. Psychoneuroendocrinology. 2024 Aug;166:107084. Epub 2024 May 18. https://doi.org/10.1016/j.psyneuen.2024.107084
1546. Al-Khalil Z, Attarian H, Dunietz GL, Gavidia Romero R, Knutson K, Johnson DA. Sleep health inequities in vulnerable populations: Beyond sleep deserts. Sleep Med X. 2024 Mar 30;7:100110. eCollection 2024 Dec. https://doi.org/10.1016/j.sleepx.2024.100110
1547. Kibibi NI, Dena I, Cummings PD, Hicks CD, Bao W, Schweizer ML. Obesity in Refugees post-resettlement in a high-income country: a meta-analysis. J Racial Ethn Health Disparities. 2024 Aug;11(4):2197-2209. Epub 2023 Jul 19. https://doi.org/10.1007/s40615-023-01688-1
1548. Mohamed AA, Shah V, Njeru JW, Wieland ML, Rutten LJF, Prokop LJ, Murad MH. Interventions to Increase Cancer Screening Adherence Among Somali Immigrants in the US and Europe: A Systematic Review. J Immigr Minor Health. 2024 Apr;26(2):385-394. Epub 2023 Aug 23. https://doi.org/10.1007/s10903-023-01532-y
1549. Bakirtzis M, Michaleakou E, Martidou ME, Lahana E, Kostagiolas P, Niakas D, Labiris G. Visual Acuity Screening of Refugees and Immigrants with a Web-Based Digital Test: A Pilot Study. Acta Medica (Hradec Kralove). 2024;67(3):79-86. https://doi.org/10.14712/18059694.2025.2
1550. Booth J, Erwin J, Burns L, Axford N, Horrell J, Wheat H, Witton R, Shawe J, Doughty J, Kaddour S, Boswell S, Devalia U, Nelder A, Paisi M. A Scoping Review of the Oral Health Status, Oral Health Behaviours and Interventions to Improve the Oral Health of Children and Young People in Care and Care Leavers. Dent J (Basel). 2024 Feb 9;12(2):38. https://doi.org/10.3390/dj12020038
1551. Vyas MV, Redditt V, Mohamed S, Abraha M, Sheth J, Shah BR, Ko DT, Ke C. Determinants, Prevention, and Incidence of Cardiovascular Disease Among Immigrant and Refugee Populations. Can J Cardiol. 2024 Jun;40(6):1077-1087. Epub 2024 Feb 21. https://doi.org/10.1016/j.cjca.2024.02.011
1552. Ngo CC, Maidment C, Atkins L, Eagar S, Smith MM. Blood screen findings in a 2-year cohort of newly arrived refugees to Sydney, Australia. Public Health Res Pract. 2018 Mar 15;28(1):2811804. https://doi.org/10.17061/phrp2811804
1553. Khuri J, Wang Y, Holden K, Fly AD, Mbogori T, Mueller S, Kandiah J, Zhang M. Dietary Intake and Nutritional Status among Refugees in Host Countries: A Systematic Review. Adv Nutr. 2022 Oct 2;13(5):1846-1865. https://doi.org/10.1093/advances/nmac051
1554. McPherson JI. Traumatic brain injury among refugees and asylum seekers. Disabil Rehabil. 2019 May;41(10):1238-1242. Epub 2017 Dec 28. https://doi.org/10.1080/09638288.2017.1422038
1555. Mateen FJ. Treating epilepsy in forcibly displaced persons: timely, necessary, affordable. Nat Rev Neurol. 2021 Oct;17(10):593-594. https://doi.org/10.1038/s41582-021-00548-y
1556. Hallab A, Sen A. Epilepsy and psychogenic non-epileptic seizures in forcibly displaced people: A scoping review. Seizure. 2021 Nov;92:128-148. Epub 2021 Aug 13. https://doi.org/10.1016/j.seizure.2021.08.004
1557. Kortas GT, Abrahão ABB, Malbergier A, Fidalgo TM, Moura H, de Andrade AG, Neto FL, Torales J, Ventriglio A, Castaldelli-Maia JM. Immigrants, refugees and cannabis use. Int Rev Psychiatry. 2022 Feb;34(1):59-77. Epub 2022 May 3. https://doi.org/10.1080/09540261.2022.2039595

1558. Altun A, Brown H, Sturgiss L, Russell G. Evaluating chronic pain interventions in recent refugees and immigrant populations: A systematic review. Patient Educ Couns. 2022 May;105(5):1152-1169. Epub 2021 Aug 27. https://doi.org/10.1016/j.pec.2021.08.021
1559. Gerber M, Filippou K, Knappe F, Morres ID, Tzormpatzakis E, Havas E, Seelig H, Colledge F, Ludyga S, Meier M, Theodorakis Y, von Känel R, Pühse U, Hatzigeorgiadis A. Associations between grip strength, cardiorespiratory fitness, cardiovascular risk and mental health in forcibly displaced people from a Greek refugee camp. Sci Rep. 2023 Nov 28;13(1):20970. https://doi.org/10.1038/s41598-023-48032-5
1560. Nieto-Martínez R, De Oliveira-Gomes D, Gonzalez-Rivas JP, Al-Rousan T, Mechanick JI, Danaei G; Migrants Health Lown Scholar Program Team. Telehealth and cardiometabolic-based chronic disease: optimizing preventive care in forcibly displaced migrant populations. J Health Popul Nutr. 2023 Sep 4;42(1):93. https://doi.org/10.1186/s41043-023-00418-x
1561. Wainman NE, Phillips OR, Morling JR. Facilitators and barriers to asylum seeker and refugee oral health care access: a qualitative systematic review. Br Dent J. 2024 Apr 11. Online ahead of print. https://doi.org/10.1038/s41415-024-7235-9
1562. Knappe F, Filippou K, Hatzigeorgiadis A, Morres ID, Ludyga S, Seelig H, Tzormpatzakis E, Havas E, Theodorakis Y, von Känel R, Pühse U, Gerber M. The impact of an exercise and sport intervention on cognitive function and pain among forcibly displaced individuals at risk for PTSD: a secondary analysis of the SALEEM randomized controlled trial. BMC Med. 2024 Sep 12;22(1):387. https://doi.org/10.1186/s12916-024-03601-x
1563. Riggs E, Rajan S, Casey S, Kilpatrick N. Refugee child oral health. Oral Dis. 2017 Apr;23(3):292-299. Epub 2016 Aug 1. https://doi.org/10.1111/odi.12530
1564. Kroening ALH, Dawson-Hahn E. Health Considerations for Immigrant and Refugee Children. Adv Pediatr. 2019 Aug;66:87-110. Epub 2019 May 18. https://doi.org/10.1016/j.yapd.2019.04.003
1565. Guerin PB, Elmi FH, Corrigan C. Body composition and cardiorespiratory fitness among refugee Somali women living in New Zealand. J Immigr Minor Health. 2007 Jul;9(3):191-6. https://doi.org/10.1007/s10903-006-9030-x
1566. Sundquist J, Hagströmer M, Johansson SE, Sundquist K. Effect of a primary health-care-based controlled trial for cardiorespiratory fitness in refugee women. BMC Fam Pract. 2010 Aug 2;11:55. https://doi.org/10.1186/1471-2296-11-55
1567. Keatley E, d'Alfonso A, Abeare C, Keller A, Bertelsen NS. Health Outcomes of Traumatic Brain Injury Among Refugee Survivors of Torture. J Head Trauma Rehabil. 2015 Nov-Dec;30(6):E1-8. https://doi.org/10.1097/HTR.0000000000000103
1568. Bhusari S, Ilechukwu C, Elwishahy A, Horstick O, Winkler V, Antia K. Dental Caries among Refugees in Europe: A Systematic Literature Review. Int J Environ Res Public Health. 2020 Dec 18;17(24):9510. https://doi.org/10.3390/ijerph17249510
1569. Weinmann A, Fontaine P, Loth K, Luepker R. Lipid Levels in Refugees from Burma. J Community Health. 2021 Dec;46(6):1083-1089. Epub 2021 Apr 30. https://doi.org/10.1007/s10900-021-00990-5
1570. Al-Rousan T, AlHeresh R, Saadi A, El-Sabrout H, Young M, Benmarhnia T, Han BH, Alshawabkeh L. Epidemiology of cardiovascular disease and its risk factors among refugees and asylum seekers: Systematic review and meta-analysis. Int J Cardiol Cardiovasc Risk Prev. 2022 Feb 10;12:200126. eCollection 2022 Mar. https://doi.org/10.1016/j.ijcrp.2022.200126
1571. Buford A, Ashworth HC, Ezzeddine FL, Dada S, Nguyen E, Ebrahim S, Zhang A, Lebovic J, Hamvas L, Prokop LJ, Midani S, Chilazi M, Alahdab F. Systematic review of

electronic health records to manage chronic conditions among displaced populations. BMJ Open. 2022 Sep 6;12(9):e056987. https://doi.org/10.1136/bmjopen-2021-056987
1572. Rahimitabar P, Kraemer A, Bozorgmehr K, Ebrahimi F, Takian A. Health condition of Afghan refugees residing in Iran in comparison to Germany: a systematic review of empirical studies. Int J Equity Health. 2023 Jan 21;22(1):16. https://doi.org/10.1186/s12939-023-01832-7
1573. Cote S, Geltman P, Nunn M, Lituri K, Henshaw M, Garcia RI. Dental caries of refugee children compared with US children. Pediatrics. 2004 Dec;114(6):e733-40. https://doi.org/10.1542/peds.2004-0496
1574. Lopriore C, Guidoum Y, Briend A, Branca F. Spread fortified with vitamins and minerals induces catch-up growth and eradicates severe anemia in stunted refugee children aged 3-6 y. Am J Clin Nutr. 2004 Oct;80(4):973-81. https://doi.org/10.1093/ajcn/80.4.973
1575. Brown RM, Canham D, Cureton VY. An oral health education program for Latino immigrant parents. J Sch Nurs. 2005 Oct;21(5):266-71. https://doi.org/10.1177/10598405050210050401
1576. Talley L, Woodruff BA, Seal A, Tripp K, Mselle LS, Abdalla F, Bhatia R, Mirghani Z. Evaluation of the effectiveness of stainless steel cooking pots in reducing iron-deficiency anaemia in food aid-dependent populations. Public Health Nutr. 2010 Jan;13(1):107-15. Epub 2009 Apr 1. https://doi.org/10.1017/S1368980009005254
1577. Thacher TD, Pludowski P, Shaw NJ, Mughal MZ, Munns CF, Högler W. Nutritional rickets in immigrant and refugee children. Public Health Rev. 2016 Jul 22;37:3. eCollection 2016. https://doi.org/10.1186/s40985-016-0018-3
1578. Reza M, Amin MS, Sgro A, Abdelaziz A, Ito D, Main P, Azarpazhooh A. ORAL HEALTH STATUS OF IMMIGRANT AND REFUGEE CHILDREN IN NORTH AMERICA: A SCOPING REVIEW. J Can Dent Assoc. 2016 Feb;82:g3.
1579. Thielemans L, Trip-Hoving M, Bancone G, Turner C, Simpson JA, Hanboonkunupakarn B, van Hensbroek MB, van Rheenen P, Paw MK, Nosten F, McGready R, Carrara VI. Neonatal Hyperbilirubinemia in a Marginalized Population on the Thai-Myanmar Border: a study protocol. BMC Pediatr. 2017 Jan 21;17(1):32. https://doi.org/10.1186/s12887-017-0798-8
1580. Dajani R, Hadfield K, van Uum S, Greff M, Panter-Brick C. Hair cortisol concentrations in war-affected adolescents: A prospective intervention trial. Psychoneuroendocrinology. 2018 Mar;89:138-146. Epub 2017 Dec 26. https://doi.org/10.1016/j.psyneuen.2017.12.012
1581. Gooding C, Musa S, Lavin T, Sibeko L, Ndikom CM, Iwuagwu S, Ani-Amponsah M, Maduforo AN, Salami B. Nutritional Challenges among African Refugee and Internally Displaced Children: A Comprehensive Scoping Review. Children (Basel). 2024 Mar 7;11(3):318. https://doi.org/10.3390/children11030318
1582. Erwin J, Horrell J, Wheat H, Axford N, Burns L, Booth J, Witton R, Shawe J, Doughty J, Kaddour S, Boswell S, Devalia U, Nelder A, Paisi M. Access to Dental Care for Children and Young People in Care and Care Leavers: A Global Scoping Review. Dent J (Basel). 2024 Feb 8;12(2):37. https://doi.org/10.3390/dj12020037
1583. Basu S, Yudkin JS, Berkowitz SA, Jawad M, Millett C. Reducing chronic disease through changes in food aid: A microsimulation of nutrition and cardiometabolic disease among Palestinian refugees in the Middle East. PLoS Med. 2018 Nov 20;15(11):e1002700. eCollection 2018 Nov. https://doi.org/10.1371/journal.pmed.1002700

1584. Silva GS, Rosenbach M. Climate change and dermatology: An introduction to a special topic, for this special issue. Int J Womens Dermatol. 2021 Jan;7(1):3-7. Epub 2020 Aug 19. https://doi.org/10.1016/j.ijwd.2020.08.002
1585. Mohammadi M, Jafari H, Etemadi M, Dalugoda Y, Mohtady Ali H, Phung H, Ahmadvand A, Dwirahmadi F, Barnes P, Chu C. Health Problems of Increasing Man-Made and Climate-Related Disasters on Forcibly Displaced populations: A Scoping Review on Global Evidence. Disaster Med Public Health Prep. 2023 Nov 23;17:e537. https://doi.org/10.1017/dmp.2023.159
1586. Veenema TG, Rains AB, Casey-Lockyer M, Springer J, Kowal M. Quality of healthcare services provided in disaster shelters: An integrative literature review. Int Emerg Nurs. 2015 Jul;23(3):225-31. Epub 2015 Jan 28. https://doi.org/10.1016/j.ienj.2015.01.004
1587. Burkle FM Jr. Mass casualty management of a large-scale bioterrorist event: an epidemiological approach that shapes triage decisions. Emerg Med Clin North Am. 2002 May;20(2):409-36. https://doi.org/10.1016/s0733-8627(01)00008-6
1588. Patz JA, Olson SH. Climate change and health: global to local influences on disease risk. Ann Trop Med Parasitol. 2006 Jul-Sep;100(5-6):535-49. https://doi.org/10.1179/136485906X97426
1589. Kirsch T, Sauer L, Guha Sapir D. Analysis of the international and US response to the Haiti earthquake: recommendations for change. Disaster Med Public Health Prep. 2012 Oct;6(3):200-8. https://doi.org/10.1001/dmp.2012.48
1590. McMichael AJ. Climate Change and Children: Health Risks of Abatement Inaction, Health Gains from Action. Children (Basel). 2014 Aug 14;1(2):99-106. https://doi.org/10.3390/children1020099
1591. De Buck E, Borra V, De Weerdt E, Vande Veegaete A, Vandekerckhove P. A systematic review of the amount of water per person per day needed to prevent morbidity and mortality in (post-)disaster settings. PLoS One. 2015 May 11;10(5):e0126395. eCollection 2015. https://doi.org/10.1371/journal.pone.0126395
1592. Veenema TG, Thornton CP. Guidance in Managing Patients Following Radiation Events. Adv Emerg Nurs J. 2015 Jul-Sep;37(3):197-208. https://doi.org/10.1097/TME.0000000000000058
1593. Carlson LC, Rogers TT, Kamara TB, Rybarczyk MM, Leow JJ, Kirsch TD, Kushner AL. Petroleum pipeline explosions in sub-Saharan Africa: a comprehensive systematic review of the academic and lay literature. Burns. 2015 May;41(3):497-501. Epub 2014 Sep 17. https://doi.org/10.1016/j.burns.2014.08.013
1594. Kwak R, Kamal K, Charrow A, Khalifian S. Mass migration and climate change: Dermatologic manifestations. Int J Womens Dermatol. 2020 Aug 20;7(1):98-106. eCollection 2021 Jan. https://doi.org/10.1016/j.ijwd.2020.07.014
1595. Cooper B, Behnke NL, Cronk R, Anthonj C, Shackelford BB, Tu R, Bartram J. Environmental health conditions in the transitional stage of forcible displacement: A systematic scoping review. Sci Total Environ. 2021 Mar 25;762:143136. Epub 2020 Oct 16. https://doi.org/10.1016/j.scitotenv.2020.143136
1596. Jay O, Capon A, Berry P, Broderick C, de Dear R, Havenith G, Honda Y, Kovats RS, Ma W, Malik A, Morris NB, Nybo L, Seneviratne SI, Vanos J, Ebi KL. Reducing the health effects of hot weather and heat extremes: from personal cooling strategies to green cities. Lancet. 2021 Aug 21;398(10301):709-724. https://doi.org/10.1016/S0140-6736(21)01209-5
1597. Sindall R, Mecrow T, Queiroga AC, Boyer C, Koon W, Peden AE. Drowning risk and climate change: a state-of-the-art review. Inj Prev. 2022 Apr;28(2):185-191. Epub 2022 Feb 23. https://doi.org/10.1136/injuryprev-2021-044486

1598. Choi SH, Beer J, Charrow A. Climate change and the displaced person: how vectors and climate are changing the landscape of infectious diseases among displaced and migrant populations. Int J Dermatol. 2023 May;62(5):681-684. Epub 2023 Mar 13. https://doi.org/10.1111/ijd.16636
1599. Bansal A, Cherbuin N, Davis DL, Peek MJ, Wingett A, Christensen BK, Carlisle H, Broom M, Schoenaker DAJM, Dahlstrom JE, Phillips CB, Vardoulakis S, Nanan R, Nolan CJ. Heatwaves and wildfires suffocate our healthy start to life: time to assess impact and take action. Lancet Planet Health. 2023 Aug;7(8):e718-e725. https://doi.org/10.1016/S2542-5196(23)00134-1
1600. Jacquet GA, Hansoti B, Vu A, Bayram JD. Earthquake-related injuries in the pediatric population: a systematic review. PLoS Curr. 2013 Nov 27;5:ecurrents.dis.6d3efba2712560727c0a551f4febac16. https://doi.org/10.1371/currents.dis.6d3efba2712560727c0a551f4febac16
1601. Delbiso TD, Altare C, Rodriguez-Llanes JM, Doocy S, Guha-Sapir D. Drought and child mortality: a meta-analysis of small-scale surveys from Ethiopia. Sci Rep. 2017 May 19;7(1):2212. https://doi.org/10.1038/s41598-017-02271-5
1602. Rebaudet S, Sudre B, Faucher B, Piarroux R. Environmental determinants of cholera outbreaks in inland Africa: a systematic review of main transmission foci and propagation routes. J Infect Dis. 2013 Nov 1;208 Suppl 1:S46-54. https://doi.org/10.1093/infdis/jit195
